# Functional analysis of the AUG initiator codon context reveals novel conserved sequences that disfavor mRNA translation in eukaryotes

**DOI:** 10.1093/nar/gkad1152

**Published:** 2023-12-01

**Authors:** Greco Hernández, Alejandra García, Shira Weingarten-Gabbay, Rishi Kumar Mishra, Tanweer Hussain, Mehdi Amiri, Gabriel Moreno-Hagelsieb, Angélica Montiel-Dávalos, Paul Lasko, Nahum Sonenberg

**Affiliations:** mRNA and Cancer Laboratory, Unit of Biomedical Research on Cancer, National Institute of Cancer (INCan), Mexico City 14080, Mexico; mRNA and Cancer Laboratory, Unit of Biomedical Research on Cancer, National Institute of Cancer (INCan), Mexico City 14080, Mexico; Broad Institute of MIT and Harvard, Cambridge, MA, USA; Department of Organismic and Evolutionary Biology, Harvard University, Cambridge, MA, USA; Laboratory of Virology and Infectious Disease, The Rockefeller University, New York, NY, USA; Department of Developmental Biology and Genetics, Indian Institute of Science, Bengaluru-560012, India; Department of Developmental Biology and Genetics, Indian Institute of Science, Bengaluru-560012, India; Department of Biochemistry and Goodman Cancer Institute. McGill University., Montreal, QC H3A 1A3, Canada; Department of Biology, Wilfrid Laurier University. 75 University Ave. W, Waterloo, ON N2L 3C5, Canada; mRNA and Cancer Laboratory, Unit of Biomedical Research on Cancer, National Institute of Cancer (INCan), Mexico City 14080, Mexico; Department of Biology, McGill University. Montreal, QC H3G 0B1, Canada; Department of Biochemistry and Goodman Cancer Institute. McGill University., Montreal, QC H3A 1A3, Canada

## Abstract

mRNA translation is a fundamental process for life. Selection of the translation initiation site (TIS) is crucial, as it establishes the correct open reading frame for mRNA decoding. Studies in vertebrate mRNAs discovered that a purine at −3 and a G at +4 (where A of the AUG initiator codon is numbered + 1), promote TIS recognition. However, the TIS context in other eukaryotes has been poorly experimentally analyzed. We analyzed *in vitro* the influence of the −3, −2, −1 and + 4 positions of the TIS context in rabbit, *Drosophila*, wheat, and yeast. We observed that −3A conferred the best translational efficiency across these species. However, we found variability at the + 4 position for optimal translation. In addition, the Kozak motif that was defined from mammalian cells was only weakly predictive for wheat and essentially non-predictive for yeast. We discovered eight conserved sequences that significantly disfavored translation. Due to the big differences in translational efficiency observed among weak TIS context sequences, we define a novel category that we termed ‘barren AUG context sequences (BACS)’, which represent sequences disfavoring translation. Analysis of mRNA-ribosomal complexes structures provided insights into the function of BACS. The gene ontology of the BACS-containing mRNAs is presented.

## Graphical Abstract

**Graphical Abstract:**
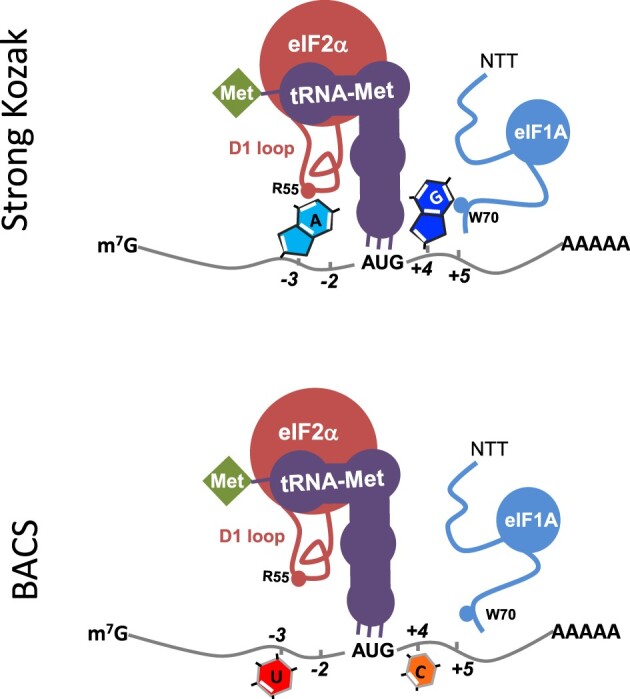

## Introduction

Recognition of the mRNA translation initiation site (TIS) by the 40S ribosomal subunit establishes the correct open reading frame for triplet decoding (1). This is achieved during the initiation step of translation, which in eukaryotes involves interaction of a free 40S ribosomal subunit with eukaryotic initiation factor (eIF) eIF1, eIF1A, eIF3, eIF5 and a ternary complex (TC), consisting of eIF2•initiator Met-tRNA_i_^Met^•GTP) to form a 43S preinitiation complex (PIC). The 43S PIC is recruited to the 5′-end of an mRNA to form the 48S PIC that scans the 5′-untranslated region (UTR) in a 5′ 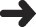 3′ direction to reach the TIS, an AUG codon or a near cognate AUG (1). Due to codon–anticodon mismatches of Met-tRNA_i_^Met^ with mRNA triplets other than AUG, the eIF1A C-terminal tail along with eIF1 prevent tight engagement of TC in the ribosomal peptidyl (P) site, which is termed P_OUT_ state. When an authentic AUG start codon is encountered by the 48S PIC, precise base-pairing with the Met-tRNA_i_^Met^ anticodon triggers eIF1 dissociation from the 40S subunit and the formation of a stable, closed PIC conformation called P_IN_ that arrests scanning. During this step, eIF1A stabilizes codon/anticodon duplex formation. AUG codon recognition triggers eIF2 release and subsequent 60S ribosomal subunit joining to assemble an active 80S initiation complex (2,3).

Not all AUG codons function equally well as translation start sites. In the 1980s, a consensus sequence flanking the TIS called the ‘Kozak motif’ was identified as being required for optimal translation of vertebrate mRNAs (4,5). This motif consists of the sequence GCCRCC*AUG*G, where A of the AUG start codon (underlined) is numbered + 1 and R at −3 is A or G (6–9). The −3R and + 4G are functionally the most important nucleotides of this motif, with −3R being the most critical nucleotide for optimal TIS recognition (5,10). When the −3 is an unfavorable pyrimidine, the +4G position becomes more critical and also significantly contributes to TIS recognition and promotion of translation. Nucleotides in positions −1C, −2C, −4C, −5C and −6C contribute in a minor way to promoting translation only in the absence of −3R and + 4G. Positions −7 to −10 and +5 and +6 show essentially no influence on translation (9,11,12). Genetic, biochemical, and structural studies in yeast and rabbit have shown interactions of −3R, −2, +4 and + 5 positions with the translation machinery during TIS recognition (13–18).

*In silico* analyses of invertebrate eukaryotes noted variability in the consensus (i.e. the most frequent) sequence surrounding the TIS (19–27). Recently, Hernández *et al.* (28) performed a comprehensive analysis of all TIS consensus contexts published from vertebrates, unicellular fungi, insects, flowering land plants, and some protists, observing that the −3R position is universally conserved among all eukaryotes and that there is significant variation in the + 4 position, particularly in unicellular fungi and some protists. Surprisingly, the −2 (A/C) position was found to be universally conserved as well (28). Depending on the presence of the two critical nucleotides (i.e. −3R and + 4G), TIS contexts have been classified as Kozak motifs that are ‘optimal’, GCC**R**CCAUG**G** (only for vertebrates); ‘strong’, **R**NNAUG**G** (the two key bases are present; N, any base); ‘moderate’, **R**NNAUGH or YNNAUG**G** (only one of the key nucleotides is present; Y, C/U; H, A/C/U); and ‘weak’, YNNAUGH (none of the key nucleotides for vertebrates is present) (5,28).

While *in silico* studies define consensus sequences (which is a statistical measure), they do not inform about the ability of a specific base to promote translation. Systematic studies to experimentally assess the influence of TIS contexts on the translational efficiency in different eukaryotes are scarce (28). Here, using cell-free *in vitro* translation extracts, we exhaustively analyzed the functional importance of the −3, −2, −1 and + 4 positions of the TIS context in four well-studied model organisms. We found that −3A conferred the best translational efficiency whereas there was variability at the +4 position for optimal translation. Importantly, we discovered eight conserved YNNAUGY sequences that significantly disfavor mRNA translation, representing a new category of TIS contexts that we termed ‘Barren AUG context sequences (BACS)’. Analyses of 48S PIC structures and gene ontology of this motif are also presented.

## Materials and methods

### Plasmid construction

Plasmids were derived from the pLUC-cassette, which contains the firefly luciferase (FLuc) cistron as a reporter and a poly(A)_71_ tail (29). All constructs were PCR-amplified using the pLUC-cassette as a template and 5′-end forward specific primers with mutations at the −3, −2, −1 and +4 positions of the AUG start codon (Supplementary Figure S1). In total, forward primers with 152 different sequences were used (Table 1). PCR fragments were gel-extracted and further cloned onto the pTZ57 R/T vector of the InsTAclone PCR Cloning kit (ThermoFisher Scientific). Clones were verified for the correct orientation by EcoRI digestion, in which the reporter cistron was placed under the vector T7 RNA polymerase promoter. All constructs were further verified by sequencing. All reagents are listed in Supplementary Table S1.

**Table 1.** AUG start codon context sequences analyzed in this study

ID^a^	Sequence^b^					K^c^	ID	Sequence					K	ID	Sequence					K	ID	Sequence					K
	−3	−2	−1		+4			−3	−2	−1		+4			−3	−2	−1		+4			−3	−2	−1		+4	
1	A	A	A	–	A	♦	33	A	U	A	–	A	♦	65	A	A	C	–	A	♦	97	A	U	C	–	A	♦
2	A	A	A	–	C	♦	34	A	U	A	–	C	♦	66	A	A	C	–	C	♦	98	A	U	C	–	C	♦
3	A	A	A	–	U	♦	35	A	U	A	–	U	♦	67	A	A	C	–	U	♦	n.t.	A	U	C	–	U	♦
4	A	A	A	–	G	**★**	36	A	U	A	–	G	★	68	A	A	C	–	G	★	100	A	U	C	–	G	★
5	C	A	A	–	A	•	37	C	U	A	–	A	•	69	C	A	C	–	A	•	101	C	U	C	–	A	•
6	C	A	A	–	C	•	38	C	U	A	–	C	•	n.t.	C	A	C	–	C	•	102	C	U	C	–	C	•
7	C	A	A	–	U	•	39	C	U	A	–	U	•	71	C	A	C	–	U	•	103	C	U	C	–	U	•
8	C	A	A	–	G	♦	40	C	U	A	–	G	♦	72	C	A	C	–	G	♦	104	C	U	C	–	G	♦
9	U	A	A	–	A	•	41	U	U	A	–	A	•	73	U	A	C	–	A	•	105	U	U	C	–	A	•
10	U	A	A	–	C	•	42	U	U	A	–	C	***B***	74	U	A	C	–	C	***B***	106	U	U	C	–	C	•
11	U	A	A	–	U	•	43	U	U	A	–	U	•	75	U	A	C	–	U	•	107	U	U	C	–	U	***B***
12	U	A	A	–	G	♦	44	U	U	A	–	G	♦	76	U	A	C	–	G	♦	108	U	U	C	–	G	♦
13	G	A	A	–	A	♦	45	G	U	A	–	A	♦	77	G	A	C	–	A	♦	109	G	U	C	–	A	♦
14	G	A	A	–	C	♦	46	G	U	A	–	C	♦	78	G	A	C	–	C	♦	110	G	U	C	–	C	♦
15	G	A	A	–	U	♦	47	G	U	A	–	U	♦	79	G	A	C	–	U	♦	111	G	U	C	–	U	♦
16	G	A	A	–	G	★	48	G	U	A	–	G	★	80	G	A	C	–	G	★	n.t.	G	U	C	–	G	★
17	A	C	A	–	A	♦	49	A	G	A	–	A	♦	81	A	C	C	–	A	♦	113	A	G	C	–	A	♦
18	A	C	A	–	C	♦	50	A	G	A	–	C	♦	82	A	C	C	–	C	♦	114	A	G	C	–	C	♦
19	A	C	A	–	U	♦	51	A	G	A	–	U	♦	83	A	C	C	–	U	♦	115	A	G	C	–	U	♦
20	A	C	A	–	G	★	52	A	G	A	–	G	★	n.t.	A	C	C	–	G	★	116	A	G	C	–	G	★
21	C	C	A	–	A	•	n.t.	C	G	A	–	A	•	85	C	C	C	–	A	•	117	C	G	C	–	A	•
22	C	C	A	–	C	•	54	C	G	A	–	C	•	86	C	C	C	–	C	•	118	C	G	C	–	C	•
23	C	C	A	–	U	***B***	55	C	G	A	–	U	***B***	87	C	C	C	–	U	***B***	119	C	G	C	–	U	•
**n.t**.	C	C	A	–	G	♦	56	C	G	A	–	G	♦	n.t.	C	C	C	–	G	♦	120	C	G	C	–	G	♦
25	U	C	A	—	A	•	57	U	G	A	–	A	•	89	U	C	C	–	A	•	121	U	G	C	–	A	•
26	U	C	A	–	C	•	58	U	G	A	–	C	•	90	U	C	C	–	C	•	122	U	G	C	–	C	•
27	U	C	A	–	U	•	59	U	G	A	–	U	•	91	U	C	C	–	U	***B***	123	U	G	C	–	U	•
28	U	C	A	–	G	♦	60	U	G	A	–	G	♦	92	U	C	C	–	G	♦	124	U	G	C	–	G	♦
29	G	C	A	–	A	♦	61	G	G	A	–	A	♦	93	G	C	C	–	A	♦	125	G	G	C	–	A	♦
30	G	C	A	–	C	♦	62	G	G	A	–	C	♦	94	G	C	C	–	C	♦	126	G	G	C	–	C	♦
31	G	C	A	–	U	♦	63	G	G	A	–	U	♦	n.t.	G	C	C	–	U	♦	127	G	G	C	–	U	♦
32	G	C	A	–	G	★	64	G	G	A	–	G	★	n.t.	G	C	C	–	G	★	128	G	G	C	–	G	★
129	C	C	G	–	C	•	137	C	A	G	–	C	•	145	C	U	G	–	C	•	153	C	G	G	–	C	•
130	C	C	G	–	U	•	138	C	A	G	–	U	•	146	C	U	G	–	U	•	154	C	G	G	–	U	•
131	C	C	U	–	C	•	139	C	A	U	–	C	•	147	C	U	U	–	C	•	155	C	G	U	–	C	•
132	C	C	U	–	U	•	140	C	A	U	–	U	•	148	C	U	U	–	U	•	156	C	G	U	–	U	•
133	U	C	G	–	C	•	141	U	A	G	–	C	•	n.t.	U	U	G	–	C	•	157	U	G	G	–	C	•
134	U	C	G	–	U	•	142	U	A	G	–	U	•	150	U	U	G	–	U	•	158	U	G	G	–	U	•
135	U	C	U	–	C	•	143	U	A	U	–	C	•	151	U	U	U	–	C	•	159	U	G	U	–	C	•
136	U	C	U	–	U	•	144	U	A	U	–	U	•	152	U	U	U	–	U	•	160	U	G	U	–	U	***B***

^a^ *n.t*., non-tested.

^b^(**—**), AUG codon. The A of the AUG is nucleotide + 1.

^c^ *K*, Kozak motifs (5,28): ★, strong: –3**(A/G)** and + 4**G**; ♦, moderate −3**(A/G)** and + 4(A/C/U) or −3(C/U) and + 4**G**; •, weak: −3(C/U) and + 4(A/C/U). ***B***, Kozak barren: barren AUG context sequences.

### *In vitro* transcription of capped and polyadenylated mRNAs

Constructs in the pTZ57 R/T vector were linearized with XhoI downstream the poly(A) tail, gel-extracted and further transcribed at 37°C for 2 h using the mMessage Machine T7 polymerase *in vitro* transcription kit (Thermo Fisher Scientific) that incorporates a cap (m^7^GpppG) at the 5′-end of mRNAs. The Dual-*reaper* plasmid that expresses the bicistronic FLuc/*reaper* IRES/RLuc reporter mRNA (30) was linearized with XhoI downstream the poly(A) tail, gel-extracted and further *in vitro* transcribed at 37°C for 2 h using the Maxiscript T3 polymerase *in vitro* transcription kit (Invitrogen) and the non-functional cap analog G(5′)ppp(5′)A (New England Biolabs). Reactions were treated with DNAse Turbo for 15 min and the mRNAs were further purified using the RNeasy minielute RNA clean up kit (Qiagen) and quantified using an Epoch spectrophotometer (BioTek). The integrity of purified mRNA was assessed by agarose gel electrophoresis. mRNAs were aliquoted and stored at −70ºC until use.

### mRNA *in vitro* translation in cell-free lysates

Each mRNA was assayed in triplicate. *In vitro* translations were performed using 96-well plates in a PCR thermocycler with an initial step of 5 min at 4ºC followed by the temperature and time indicated below for each species and a final step of 15 minutes at 4ºC. Reactions were stopped with 10 μl (RRL, wheat and yeast) or 20 μl (*Drosophila*) of cold 1X Pasive lysis buffer (Promega), placed on ice for additional 10 min and stored at −20ºC until further analysis. In every 96-well plate the Dual-*reaper* bicistronic reporter mRNA was included as a control, that contains the non-functional ApppG cap, the FLuc as a first cistron, and the Renilla luciferase (RLuc) as a second cistron under the translational control of the *Drosophila reaper* IRES (30).

*In vitro* translation in rabbit reticulocyte lysates (RRL, Promega) was performed using 15 ng mRNA per reaction in a total volume of 14 μl for 90 min at 30ºC, according to the manufacturer′s instructions. Each reaction contained 9.8 μl of rabbit lysate (70%); 11 units RNasin; 20 μM of each amino acid; 10 mM creatine phosphate; 0.05 μg/μl creatine phosphokinase; 2 mM DTT; 0.05 μg/μl calf liver tRNA; 79 mM KCH_3_COO; 0.5 mM Mg(CH_3_COO)_2_; and 0.02 mM hemin*. In vitro* translation in *Drosophila* was performed using 0–12-hr-old embryo lysates and 30 ng mRNA per reaction, in a total volume of 17 μl for 60 min at 25ºC according to Hernández *et al.* and Gebauer *et al.* (29,30). Each reaction contained 5 μl of embryo lysate (30% vol/vol) and 12 μl of a cocktail containing 1.25 units RNasin; 60 μM of each amino acid; 17 mM creatine phosphate; 80 ng/μl creatine phosphokinase; 24 mM KOH–HEPES pH 7.4; 0.4 mM Mg(CH_3_COO)_2_; 30 mM KCH_3_COO; 100 ng/μl tRNA from calf liver; 30 ng mRNA; and 0.1 mM spermidine. *In vitro* translation in wheat was performed using wheat germ lysates (Promega) using 30 ng of mRNA for 90 min at 25°C in a final volume of 12.5 μl, according to the manufacturer. Each reaction contained 6.4 μl of wheat lysate (51%); 4 units RNasin; 80 μM of each amino acid; 10 mM creatine phosphate; 0.05 μg/μl creatine phosphokinase; 5 mM DTT; 72 mM KCH_3_COO; 2.1 mM Mg(CH_3_COO)_2_; 0.5 mM spermidine; 1.2 mM ATP; and 0.1 mM GTP. *In vitro* translation in *S. cerevisiae* lysates was performed according to Altmann and Trachsel (31), using 11 ng mRNA per reaction in a total volume of 3 μl for 60 min at 25ºC. Each translation reaction contained 1 μl of yeast lysate (33% vol/vol) and 1 μl of a cocktail containing 40 μM of each amino acid; 25 mM creatine phosphate; 0.5 μg/μl creatine phosphokinase; 22 mM KOH–HEPES pH 7.4; 1.5 mM Mg(OAc)_2_; 120 mM KOAc; 1.7 mM DTT; and 0.75 mM ATP; 0.1 mM GTP.

### Quantitation of synthesized active luciferase

10 μl aliquots of each reaction were assayed with 50 μl FLuc substrate of the Luciferase reporter assay substrate (Promega). Relative light units were detected in an automatic luminometer FLUOstar Omega BMG (Labtech), programmed to take 10 readings spaced 1 second apart. To correct for possible variability during the PCR, FLuc activities of each mRNA was standardized to the average of RLuc activity driven by the *Drosophila reaper* IRES included in every 96-wells plate.

### Motif enrichment analysis of TIS context sequences

To visualize the TIS context, sequence logos were plotted (32). In each system sequences were divided into high and low expression based on luciferase measurement. Enrichment scores were computed for each nucleotide in each position using the following formula as previously described (33): *E*_set[*i,k*]_=*P*_set[*i,k*]_*log_2_(*P*_set[*i,k*]_/*P*_back[*i,k*]_), where *P*_set[*i,k*]_ denotes the probability of nucleotide *i* at position *k* in a subset of sequences (20% of sequences with highest luciferase expression) and *P*_back[*i,k*]_ is the probability of the same nucleotide at the same position in the background set (the remining 80% of the sequences). The relative height of individual symbols (A, C, G or U) equals to *E*_set[*i,k*]_, whereas enrichment and depletion are indicated by positive and negative *E*_set[*i,k*]_ values, respectively. Sequence logos were plotted in Python using the Logomaker package (32). Probabilities were computed using the logomaker.alignment_to_matrix function of Logomaker and the pseudocount parameter was set to 0.01.

### Ribosome profiling of BACS-containing mRNAs

We utilized publicly available ribosome profiling datasets in GWIPS-Viz browsers (34) (http://gwips.ucc.ie/) for the visualization of ribosome footprints (RFPs) across *S. cerevisiae* genome. We then used the built-in visualization tools to view the ribosome footprint coverage for mRNAs of interest.

### 48S structural analysis

We analysed all 48S PIC in the P_IN_ state published to date. We searched all PDBs for 48S complexed with mRNA and tRNA in the P_IN_ state. In the P_OUT_ state, no 48S PDB has mRNA coordinates for Kozak positions. We found out that PDB 7Q7P of human 48S and PDB 6GSN of yeast 48S have coordinates for mRNA with Kozak positions. Comparison and analysis of the two provided a mechanism of how these positions moderate the translation initiation rate. Analysis of atomic coordinates of the 48S structures was performed in *Coot* (35) and the figures were prepared in ChimeraX (36).

### Gene ontology (GO) analysis

Genome sequences and annotations were downloaded from NCBI’s RefSeq database (37). The genomes were *Drosophila melanogaster* (fruit fly: GCF_000001215.4); *Oryctolagus cuniculus* (rabbit: GCF_000003625.3); and *Triticum aestivum* (wheat: GCF_018294505.1). We wrote *ad hoc* programs in PERL to find and extract the start codon for each annotated gene, plus three bases upstream and one downstream. These were tested to match the YCMAUGY pattern using a regular expression ([CT]C[AC]ATG[CT]). For more specific sequences (Table 2), we found them by pattern matching. Gene Ontology annotations were obtained by matching the encoded proteins to the UniProt resource (38). Proteins had to be more than 99% identical to be deemed a proper match for transference of GO annotations. Comparisons were run using DIAMOND (39), and GO annotations were obtained from the full UniProt protein sequence annotations file (uniprot_trembl.dat), also using an *ad hoc* program written in PERL.

**Table 2.** Twenty percent less efficient AUG context sequences^a^

Rabbit		Fruit fly		Wheat		Common to all three species	
*mRNA ID*	*Sequence* ^b^	*mRNA ID*	*Sequence*	*mRNA ID*	*Sequence*	*mRNA ID*	*Sequence*
#130	CCG — U	#157	UGG — C	#86	CCC — C	#160	UGU — U
#122	UGC — C	#131	CCU — C	#160	UGU — U	#42	UUA — C
#159	UGU — C	#160	UGU — U	#137	CAG — C	#107	UUC — U
#160	UGU — U	#107	UUC — U	#22	CCA — C	#23	CCA — U
#157	UGG — C	#122	UGC — C	#130	CCG — U	#74	UAC — C
#42	UUA — C	#42	UUA — C	#107	UUC — U	#55	CGA — U
#107	UUC — U	#159	UGU — C	#42	UUA — C	#91	UCC — U
#23	CCA — U	#23	CCA — U	#74	UAC — C	#87	CCC — U
#74	UAC — C	#55	CGA — U	#23	CCA — U		
#55	CGA — U	#74	UAC — C	#91	UCC — U		
#91	UCC — U	#91	UCC — U	#55	CGA — U		
#87	CCC — U	#87	CCC — U	#87	CCC — U		

^a^From the best to the less efficient sequence.

^b^—, AUG initiator codon.

## Results

We experimentally analyzed the impact of the −3, −2, −1 and + 4 positions surrounding the TIS (Table 1) on the translation efficiency in the fruit fly *Drosophila melanogaster*, the wheat *Triticum aestivum*, rabbit (*Oryctolagus cuniculus*), and the budding yeast *Saccharomyces cerevisiae*. We used a cell-free *in vitro* translation approach with mRNAs expressing the firefly luciferase (FLuc) coding sequence as a reporter. Overall, 147 (rabbit, fruit fly, and wheat) or 120 (yeast) TIS context sequences were tested. Because the mRNA at the −1 position does not likely contribute to promoting translation in vertebrates (9,11,12), and no contact between this position with the translation machinery has been reported during TIS recognition (13–18), we first analyzed only A or C at −1 in combination with all possible combinations at −3, −2 and + 4. In this way, we tested 90–94% of all 128 possible combinations of the functional positions −3, −2 and + 4 (Table 1).

**Figure 1.**
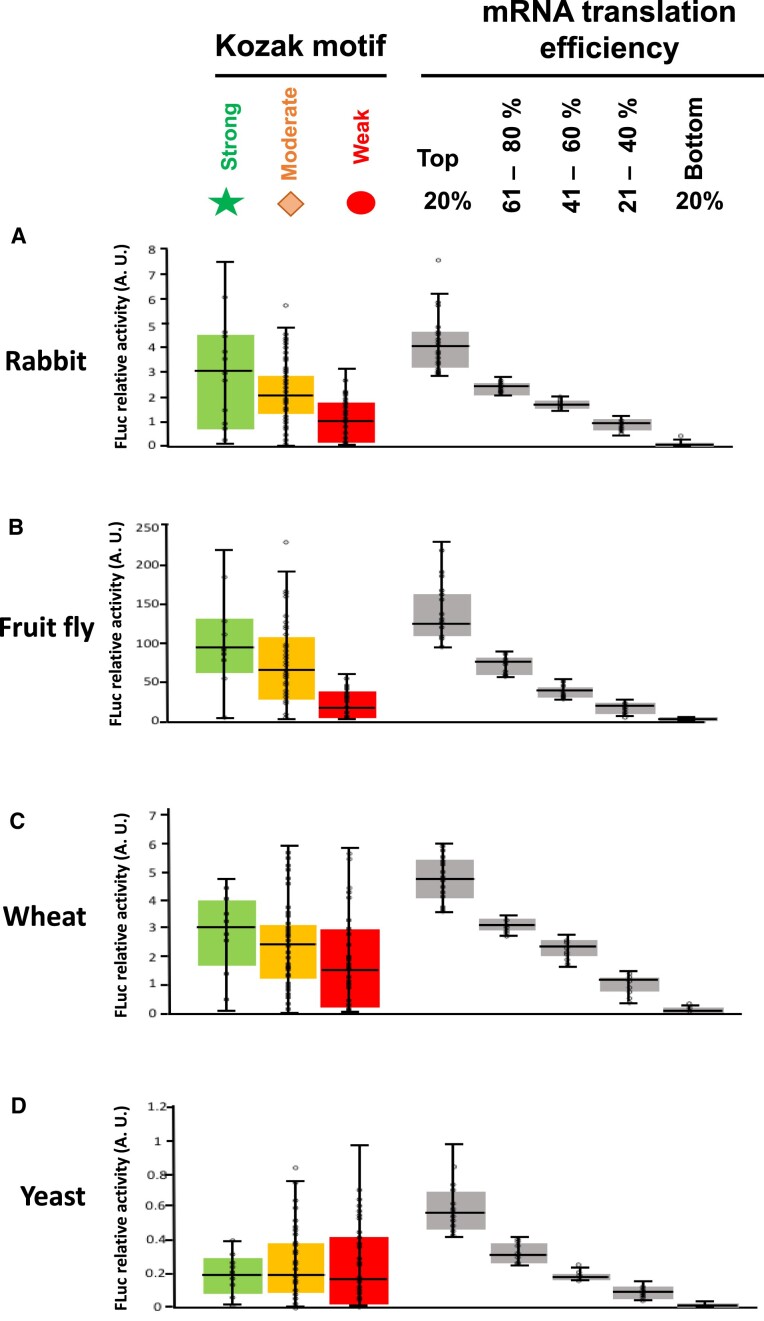
TIS context sequences confer different translational activity across species. Expression levels of 115 (rabbit (**A**), fruit fly (**B**) and wheat (**C**)) or 120 (yeast (**D**)) mRNAs is shown by box and whisker plots. Each dot represents a TIS sequence. *Left*) Translational efficiency of mRNAs possessing *Strong*, *Moderate* or *Weak* TIS contexts. *Right*) mRNAs expression according to translational efficiency. Horizontal lines represent the median. Note that the bottom mRNAs are tightly grouped and show the least variability in the translational efficiency.

We ranked the translation efficiency of mRNAs according to the type of Kozak motif, namely strong, moderate, and weak. In rabbit (Supplementary Table S2), *Drosophila* (Supplementary Table S3), and wheat (Supplementary Table S4), the median of the translation values was higher for the group of strong mRNAs, smaller in the moderate transcripts, and had the lowest values in the weak mRNAs (Figure 1, left). In contrast, yeast (Supplementary Table S5) showed very similar median values among the three groups of transcripts. The distribution of the values was widely dispersed for strong mRNAs in rabbit, *Drosophila*, and wheat (Figure 1A-C), but not in yeast (Figure 1D). On the other hand, the weak mRNAs showed the broadest value dispersion in wheat and yeast. We conclude that, for wheat and yeast the differences between strong and weak motifs with respect to promoting translation initiation are smaller than for rabbit and the fruit fly. We next plotted the TIS context sequences according to their translation efficiency. The 20% top mRNAs that promoted translation best in the four organisms showed the highest sequence variability. In contrast, the variability among the less efficient sequences was significantly narrowed (Figure 1 right, A–D). Remarkably, the 20% least efficient sequences were tightly grouped. It is noteworthy that the correlation between the classification of Kozak motifs as strong, moderate, and weak, and the experimentally determined translation efficiency, was good in rabbit and fly, but only modest in wheat and essentially nonexistent in yeast (Figure 1D). Thus, the Kozak motif that was derived from experiments in mammalian cells is not applicable to wheat and yeast. The expression level of the 20% most efficiently translated mRNAs with respect of that of the 20% least efficiently translated was 2381.2 in rabbit, 2935.1 in *Drosophila*, 919.7 in wheat, and 554 in yeast.

**Figure 2.**
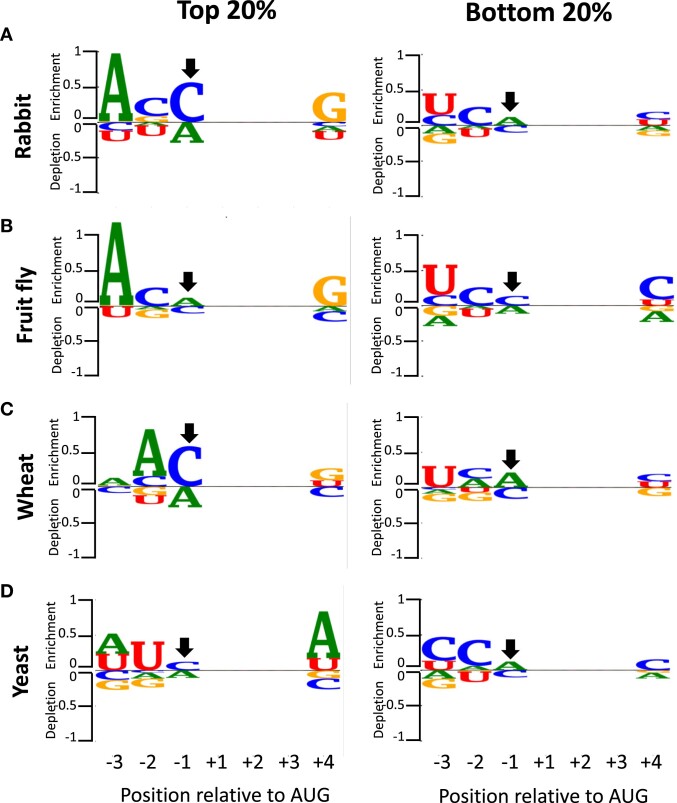
Consensus of the TIS contexts with the 20% highest and 20% lowest expression. Sequence logos of enrichment and depletion for the top (*left*) or bottom (*right*) expressed sequences. (**A**) Rabbit; (**B**) fruit fly; (**C**) wheat); (**D**) yeast. Note that *YCMAUGY* is the most represented sequence for the translationally least efficient mRNAs across the analyzed species. *M* represents A or C, and Y represents U or C. In all cases, an arrow indicates that only A and C were tested at the −2 position.

Because of the poor correlation in yeast and wheat of experimentally determined translational activity with similarity to the consensus Kozak motif, we assessed the nucleotide frequency of the sequences with the highest and lowest translational efficiencies. For each position, enrichment (over-representation) and depletion (under-representation) of nucleotides was calculated according to Dvir *et al.* (33). Consistent with published results (12,40), logo-like representations show that the 20% most active sequences in rabbit (Figure 2A) and *Drosophila* (Figure 2B) had a strong Kozak consensus motif; in particular, enrichment of the −3A and + 4G bases. However, the top 20% sequences with the highest luciferase measurements in wheat (Figure 2C) and yeast (Figure 2D) did not share a canonical strong Kozak consensus motif (Figure 2, left). For instance, in the most highly translated RNAs from yeast, +4A was more predominant than +4G (Figure 2 left). In contrast, we found a high similarity in the consensus sequences of the 20% least active RNAs in all four organisms (Figure 2, right). These poorly translated sequences preferentially contained −3U/C, −2C and + 4C. Thus, we conclude that the consensus sequence YCMAUGY (Y represents C or U) correlates with weak translational activity. We also analyzed the nucleotide frequency of the 20% top and 20% bottom sequences according to the Cavener consensus rules for TIS context analysis (19), and obtained similar results (data not shown).

**Figure 3.**
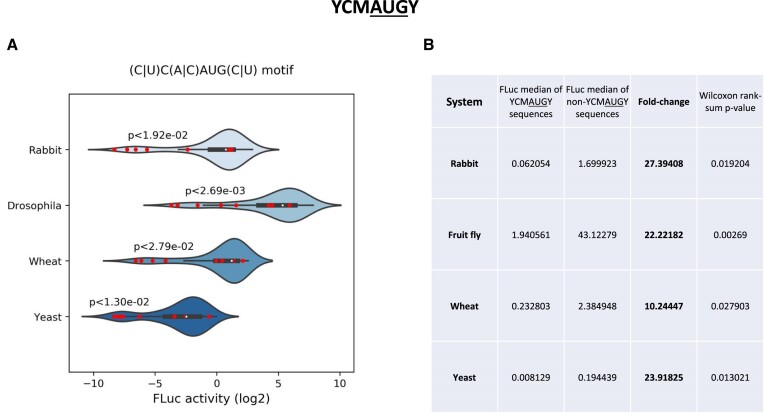
The YCMAUGY motif correlates with poor translation initiation. (**A**) Violin plots showing the distribution of FLuc expression measurements of all tested sequences in each of the four systems. Sequences that harbor the YCMAUGY [(C/U)C(A/C)AUG(C/U)] motif are highlighted in red circles on each distribution. To determine if the reduction in expression is statistically significant, Wilcoxon rank-sum test (**B**) was performed, and the computed *P*-values are denoted on top of each violin plot.

**Figure 4.**
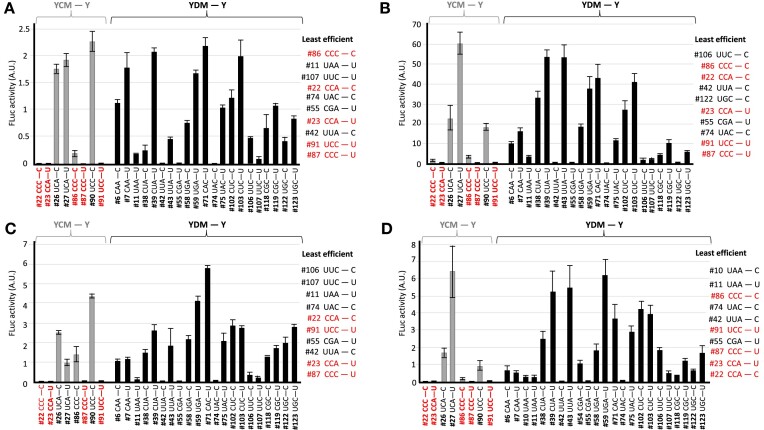
The −2C position of the YCMAUGY motif confers differential translational efficience. *YCMAUGY* sequences (*grey bars*) were compared with all *YDMAUGY* sequences (*black bars*) in rabbit (**A**), fruit fly (**B**), wheat (**C**) and yeast (**D**). For each sequence evaluated, the respective mRNA ID *#* (Table 1) and the TIS context sequence is indicated. *Y* represents C or U; *D* represents G, A, or U; —, AUG initiator codon; *A.U*., arbitrary units. For each species, the least efficient sequences are shown to the right. YCMAUGY sequences are indicated in *red*.

To corroborate that the YCMAUGY motif negatively correlates with translation efficiency, we compared luciferase activity measurements of YCMAUGY sequences to all other sequences (Figure 3A). In all four translation systems, we observed significant reduction in expression with fold-changes of 27.39, 22.22, 10.24, and 23.9 for rabbit, *Drosophila*, wheat, and yeast, respectively, with *P*< 0.02 for rabbit, *P*< 0.003 for *Drosophila*, *P*< 0.03 for wheat, and *P*< 0.01 for yeast (Wilcoxon rank-sum test) (Figure 3B). We next evaluated the influence of the −2C position of YCMAUGY sequences on translation efficiency relative to the other 3 nucleotides (YDMAUGY; D represents G, A, or U) in the four species (Figure 4). No significant enrichment of −2C was observed. Indeed, all four nucleotides conferred different translational efficiency that depended on the bases in the other positions. Interestingly, we observed that some sequences significantly disfavored translation. The ten least efficient TIS context sequences for rabbit, fruit fly, wheat, and yeast are shown in Figure 4. Sequences CCCAUGU (mRNA #87), UCCAUGU (mRNA #91), UUAAUGC (mRNA #42), CGAAUGU (mRNA #55), CCAAUGU (mRNA #23), UACAUGC (mRNA #74) and CCAAUGC (mRNA #22) were among the least efficient sequences in the four species.

**Figure 5.**
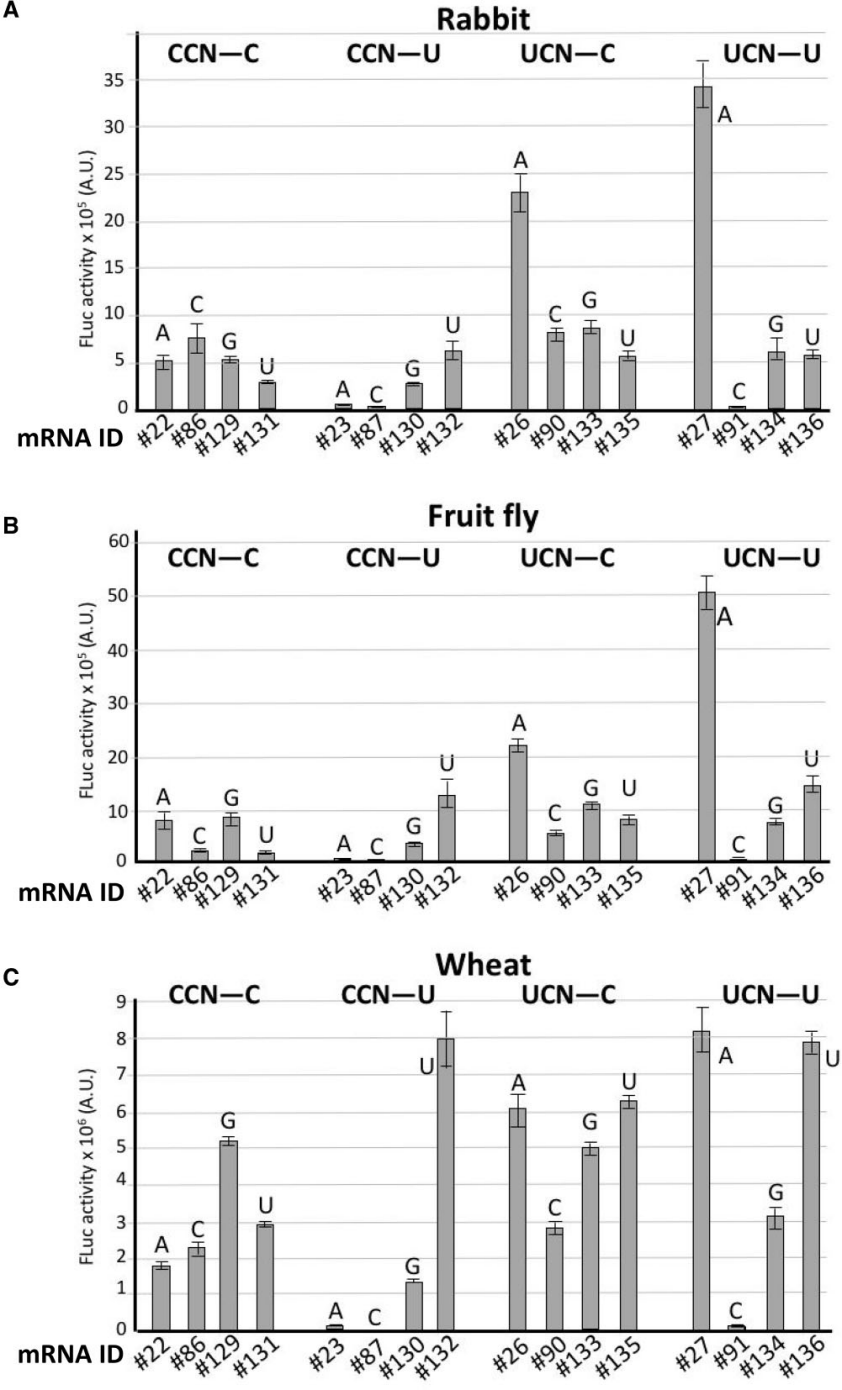
The −1C position of the YCNAUGY motif confers differential translational efficiency. All sequences *YCNAUGY* were compared in rabbit (**A**), fruit fly (**B**) and wheat (**C**). For each sequence evaluated, the respective mRNA ID *#* (Table 1) is indicated. *N* represents any of the nucleotides showed in bars. The evaluated TIS context sequences are indicated on top. *D* represents G, A, or U; —, AUG initiator codon; *A.U*., arbitrary units.

In the YCMAUGY motif, the M at the −1 position was deduced because we only tested nucleotides A and C. To expand this analysis, we examined G and U. Translational efficiency of mRNAs containing A and C at the −1 position (mRNAs #22, #23, #26, #27, #86, #87, #90 and #91) were compared with those containing G and U (mRNAs #129 – #136) in all possible combinations of YCNAUGY in rabbit, fruit fly and wheat (Figure 5). All four nucleotides at −1 also differently influenced translation depending on the other positions. Moreover, some combinations also disfavored it. This was the case of CC(A/C)AUGU (mRNAs #23 and #87) and UCCAUGU (mRNA #91) in rabbit; CC(C/U)AUGC (mRNAs #86 and #131), CC(A/C)AUGU (mRNAs #23 and #87), and UCCAUGU (mRNA #91) in the fruit fly; and CC(A/C)AUGU (mRNAs #23 and #87) and UCCAUGU (mRNA #91) in wheat. Remarkably, we noticed that −1A/C in CC(A/C)AUGU (mRNAs #23 and #87) and −1C in UCCAUGU (mRNA #91) strongly disfavored translation across the three species, an effect also observed in yeast (Supplementary Table S5). In the fruit fly C/U at −1 in the sequences CC(C/U)AUGC (mRNAs #86 and #131) had a strong disfavoring effect as well (Figure 5).

**Figure 6.**
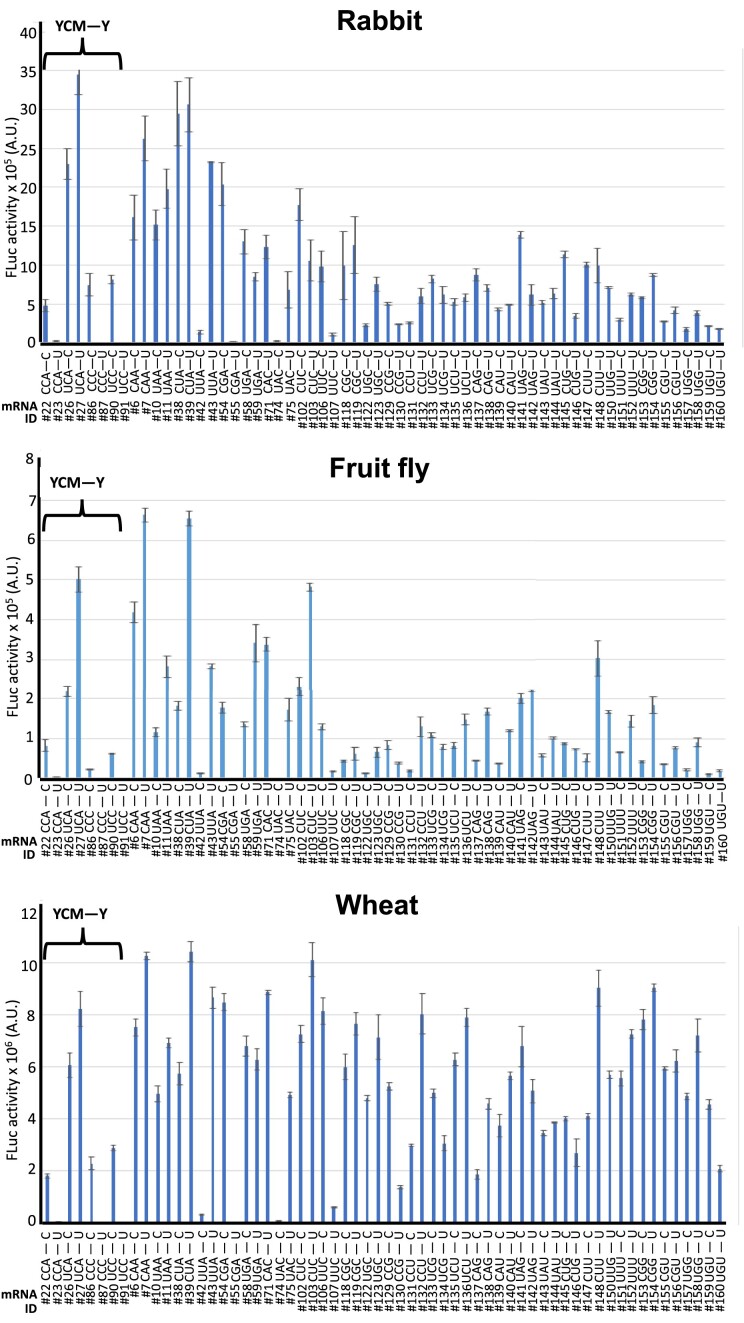
YCMAUGY and YNNAUGY sequences confer variable translational efficiency. *YCMAUGY* sequences were compared to all possible *YRDAUGY* sequences in rabbit (top), fruit fly (middle), and wheat (bottom). For each sequence evaluated, the respective mRNA ID *#* (Table 1) and the TIS context sequence is indicated. *N* represents any nucleotide; R, represents A/G; *D* represents G, A, or U; —, AUG initiator codon; *A.U*., arbitrary units.

The translational efficience of YCMAUGY sequences was also compared to that of all possible sequences containing −3C/U and + 4C/U, i.e. YNNAUGY, in rabbit, fruit fly, and wheat (TIS context sequences mRNAs #129–#160 in Table 1). As it is shown in Figure 6, we observed variable influence on FLuc expression that depended on the sequence, but no enrichment of any specific nucleotide was observed at the −2 and −1 positions. Interestingly, we observed again some of them strongly disfavoring translation. The 20% less efficient AUG context sequences in all three species is shown in Table 2. Among them, we found that eight sequences are conserved in rabbit, *Drosophila*, and wheat (Table 2) and that six are also conserved in the 20% least efficient sequences in yeast (Supplementary Table S5), namely CCAAUGU (mRNA #23), UUAAUGC (mRNA #42), CGAAUGU (mRNA #55), CCCAUGU (mRNA #87), UACAUGC (mRNA #74) and UCCAUGU (mRNA #91). We conclude that these weak Kozak sequences significantly disfavor translation and are conserved across the species analyzed. We termed these barren sequences ‘BACS’, and conclude that they represent a new category of weak TIS context sequences.

**Figure 7.**
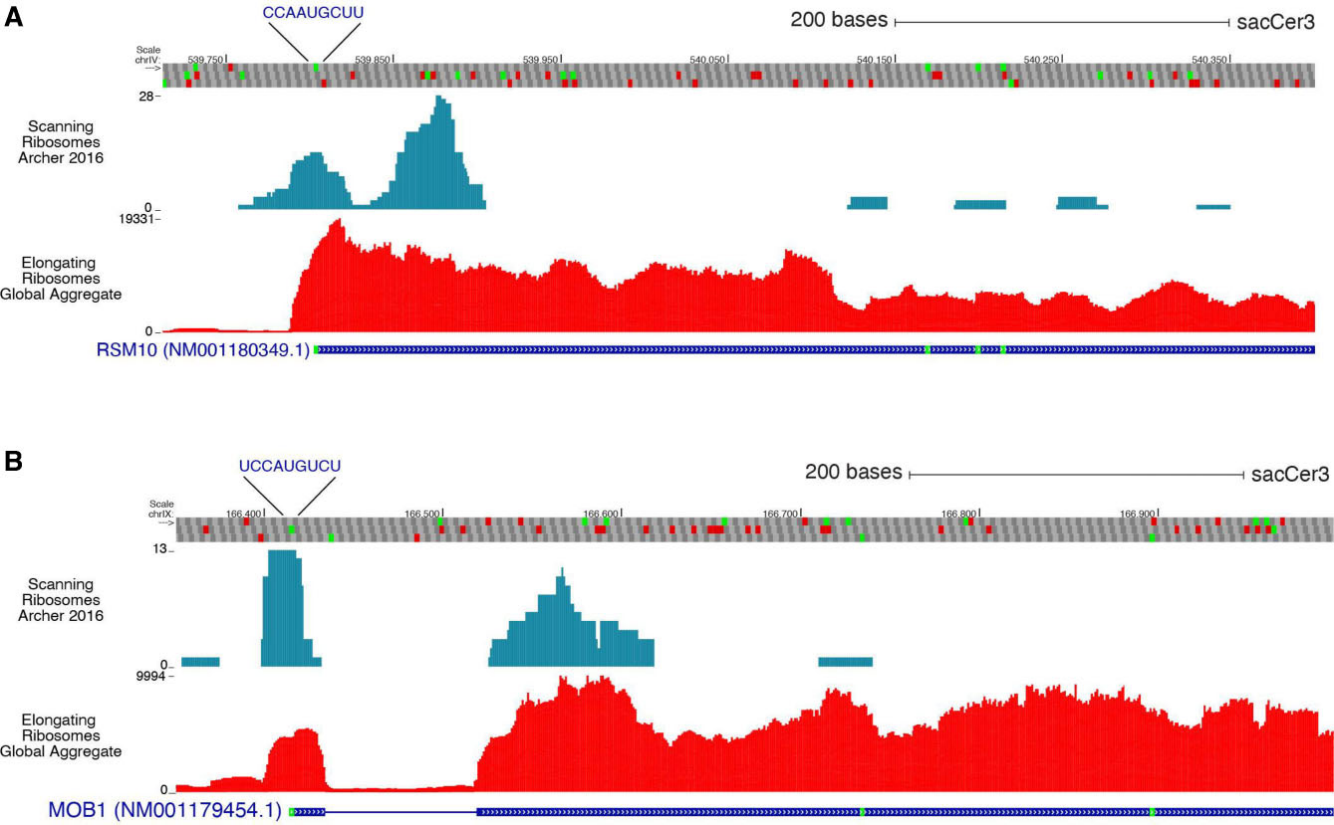
Leaky scanning in YCMAUGY-containing mRNAs from yeast. Ribosome profiling traces of scanning ribosomes from Archer *et al.* (67) (top) and elongating ribosomes (aggregate footprints coverage, bottom) on yeast *RMS10* (**A**) and *MOB1* (**B**) mRNAs visualized using GWIPS-Viz web browser. Both mRNAs show poor translation initiation from the BACS motif and show initiation events downstream.

We next analyzed the translational activity of actual YNNAUGY-containing mRNAs in ribosomal profiles from the publicly available datasets. Using GWIPS-Viz (34), we observed that some yeast mRNAs had evidence of leaky scanning, as seen by the presence of scanning ribosome footprints downstream of their start sites. The specific mRNAs examined were RSM10 (Figure 7, top) and MOB1 (Figure 7, bottom), both of which contain the TIS context CCAAUGCUU and UCCAUGUCU, respectively.

### Possible structural basis of consensus sequences disfavoring translation initiation

**Figure 8.**
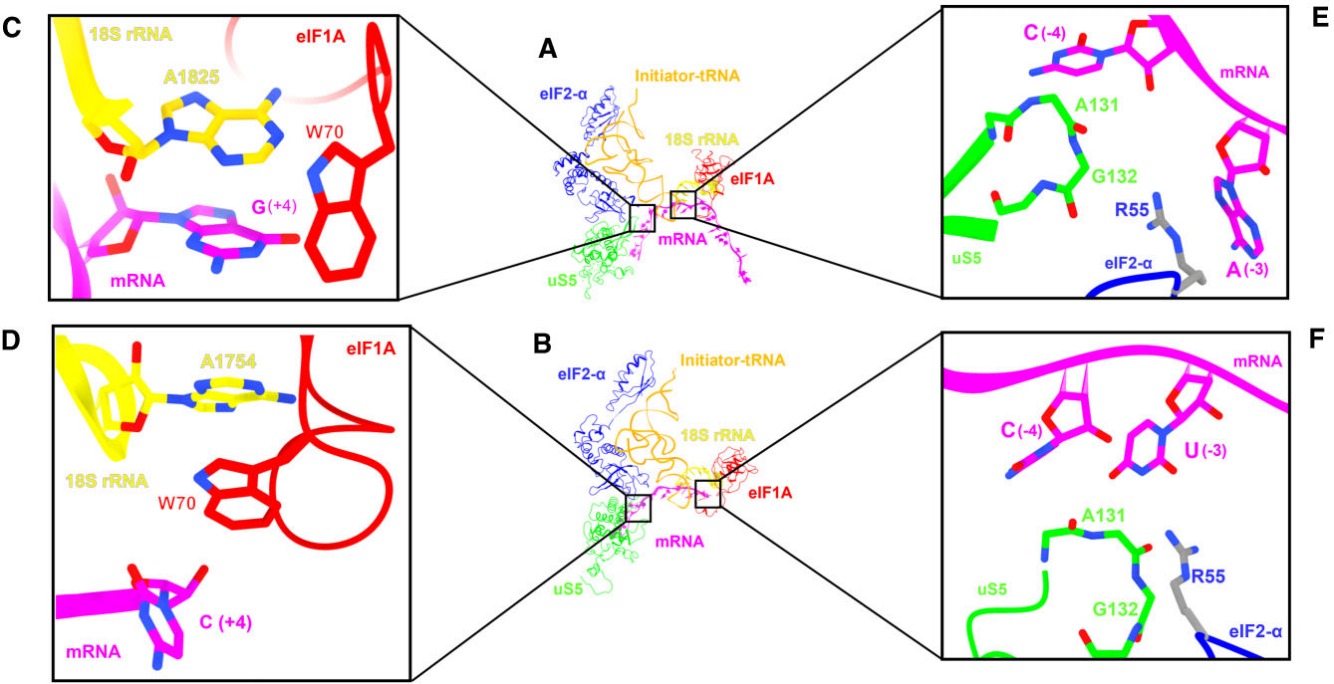
Depiction of mRNA with interacting partners on the 48S PIC in closed state. (**A**) Mammalian complex. (**B**) Fungal complex. (**C**) Stacking interaction between purine (guanine) at +4 with A1825 and absence of interaction in case of (**D**) pyrimidine (cytosine) at +4 position. (**E**) Interaction between R55 (eIF2α) and (**E**) purine (adenine) and (**F**) pyrimidine (uridine) at −3 position of mRNA where bulky purine makes extensive interactions with R55 while the smaller pyrimidine makes a weak contact with the same. The hairpin loop of uS5 ribosomal protein is also depicted in (E) and (F).

To explore a possible structural basis through which the BACS disfavor translation, we analyzed several experimentally resolved structures of YNNAUGY from rabbit (Figure 8A) and yeast (Figure 8B) 48S translation initiation complexes in the P_IN_ state. The nitrogenous base of purine at position + 4 in the human 48S structure (PDB: 7QP7) (41) stacks with the base ring of the conserved A1825 at the top of h44 of the 18S ribosomal RNA and interacts with the conserved W70 of eIF1A (Figure 8C). Guanine-adenine (G:A) stacking is significantly stronger than adenine-adenine (A:A) stacking which is consistent with the higher rate of mRNA translation for G at +4 position compared to that of A (42). However, in the case of pyrimidine at position +4 a similar interaction is not observed in the yeast 48S (PDB: 6GSN) (43) (Figure 8D).

Similar analyses of nucleotide at position −3 showed that the double ringed base of purine allows formation of extensive stacking interactions with eIF2α R55 (PDB: 7QP7) (Figure 8E and F). However, with pyrimidine at −3 position, the single ring of nitrogenous base C/U allows only weak interaction with an R55 in stretched side chain conformation (PDB: 6GSN). Accordingly, the −3U was crosslinked less efficiently with eIF2α than −3G in rabbit reticulocyte lysates (RRL) (16). Further, a recent computational study of the P_OUT_ form of yeast 48S reports that −3 purine base of mRNA interacts with eIF2α R54 during scanning (44). Thus, stable and extensive interactions between mRNA bases at −3 and +4 positions with 40S and eIFs in the case of strong Kozak context might allow stable codon–anticodon interaction engendering optimal translation initiation. In contrast for BACS, weak interactions between mRNA bases at −3 and + 4 positions with 40S (in human and yeast) and eIFs engender leaky scanning and hence lower initiation rates.

### Functionality of the BACS-containing genes

**Figure 9.**
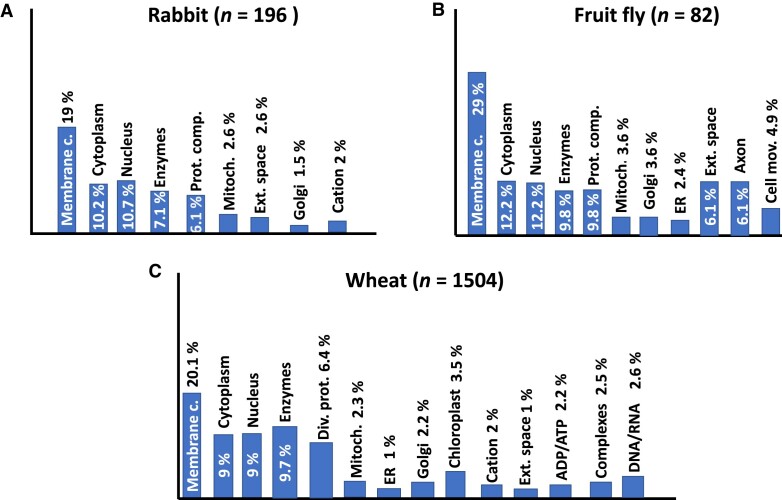
Gene ontology (GO) of enriched BACS-containing genes. Complete genomes were analyzed, and genes containing BACS as TIS context sequence were grouped according to the annotated GO. The total number of genes analyzed per species is indicated. *Membrane c*., membrane component; *Complexes*, any macromolecular complex; *Enzymes*, any catalytic enzyme; *Mitoch*., mitochondrion; *ER*, endoplasmic reticulum; *Cation*, proteins binding metal divalent cations (zinc, cobalt, copper or magnesium); *Golgi*, Golgi apparatus; *Cell mov*., cell movement; *Div. prot*., diverse proteins; *ADP/ATP*, ADP/ATP-binding proteins; *DNA/RNA*, DNA/RNA-binding proteins.

Next, we performed genome-wide analyses to determine the function of the BACS-containing genes described in Table 2 in rabbit, fruit fly, and wheat. We found subsets of 196, 82, and 1504 genes in rabbit, fruit fly, and wheat respectively (Supplementary Tables S6–S8). According to the annotated gene ontologies (GO), genes encoding membrane proteins were the most enriched (19% in rabbit, 29% in the fruit fly and 20.1% in wheat). Overall, the most represented GO was 16021, i.e. ‘Integral component of membrane’ genes. The distribution of all genes for each species studied is depicted in Figure 9.

## Discussion

Few eukaryotic species have been studied to experimentally determine the functional contribution of the mRNA TIS context that include the −3 to +4 positions. Historically, all the studies have focused mainly to find sequences for optimal translation. For mammals, Kozak studied 66 sequences by plasmid transfection into monkey cells (9,11) and 12 mRNAs in cell-free *in vitro* translation using RRL (12) to assess nucleotides in positions −1 to −10 and the G at +4. Also, by plasmid transfection into mouse cells, two studies found that −3A/G conferred the highest translational efficiency, and that the + 4 position shows variability (i.e. +4G was not necessarily the optimum nucleotide) (45,46). In human transfected cells, Ivanov *et al.* systematically analyzed the −3 and +4 positions (47), demonstrating that −3A followed by −3G conferred the best translation efficiency. In *Drosophila*, a cell plasmid transfection approach (40) employed 680 sequences, concluding that AAAAUGG is the most efficient sequence to promote translation.

In land plants, the following TIS contexts have been experimentally assessed: *Nicotiana tabacum* (tobacco), 16 sequences (48); *Picea abies* (Norway spruce), 16 sequences (48); *Zea mays* (corn), 16 sequences (48); *Arabidopsis thaliana* (thale cress), 64 sequences (49); *Oryza sativa* (rice), 64 sequences (49); and *Triticum aestivum* (wheat), 24 sequences (6,50). In *O. sativa* and *A. thaliana* the sequences (A/G)(U/C)(G/C)AUGU and (A/G)(A/C)(A/G)AUGU sequences, respectively, showed the optimal translational efficiency, whereas in the other plants −3(A/C) and + 4G (i.e. Kozak strong) were the optimal sequences.

In the fungi *S. cerevisiae*, *Cryptococcus neoformans*, *Neurospora crassa*, *Candida albicans*, and *Schizosaccharomyces pombe*, RNA-Seq and ribosome profiling studies reported that for the 5% most translated mRNAs the TIS consensus context contains −3A, −2A (−2N only for *C. albicans*), and + 4N (51). In *S. cerevisiae*, several studies (51–57) showed that −3A confers the highest efficiency in translation. In contrast, other studies in *S. cerevisiae* showed that the absence of a −3 purine only moderately affects mRNA translation (33,52,56,58–60). Recently, Li *et al.* (61) found that during the AUG start codon selection in yeast, the ribosome performs approximately ten small-amplitude back and forth oscillations per triplet with a net 5′-3′movement. In this process, changes of the −3A position favored leaky scanning of the authentic AUG initiator to a downstream AUG.

We have used a cell-free *in vitro* translation approach to analyze the influence of TIS context sequence in four systems. Translation experiments in cell extracts circumvent confounding effects of differential RNA transcription, mRNA turnover or storage, and mRNA export that might mislead conclusions in cells. However, in a cell-free *in vitro* approach different buffer conditions may importantly alter the fidelity of TIS recognition. For example, RRL require optimized buffer conditions to improve the fidelity of TIS recognition so that it resembles that found in live cells (62). Moreover, changes in the concentration of specific initiation factors, including eIF1 and eIF5, also may alter TIS recognition fidelity to certain extent (63). Thus, in the cell-free *in vitro* approach, TIS recognition fidelity might be affected due to changes in the dilution of eIF1 and eIF5 as well. Nevertheless, cell-free *in vitro* translation in all systems so far published have been optimized to increase the efficience of translation and therefore that of TIS recognition (64,65). Our results and those of other *in vitro* studies complement *in vivo* approaches.

We have tested 152 different sequences flanking the TIS, comprising most of the possible combinations of the positions at −3, −2, −1, and + 4 positions. An A at the −3 position conferred the best translational efficiency in rabbit, fruit fly and wheat, and −3(A/U) in yeast. Moreover, we found functional variability in the + 4 position. Our results agree with the consensus TIS context reported for all eukaryotes studied so far (28), in which −3(A/G) is the most conserved position and the + 4 shows variability. It is noteworthy that yeast showed the most divergent TIS consensus context to efficiently promote translation, i.e. uridine-rich at the −2 and −3 and adenine at + 4 position. Indeed, in yeast the degree of a match to the strong Kozak consensus did not have much effect on translational efficiency, in agreement with Niederer *et al.* (2022) (53). Thus, in yeast the requirement for specific sequences flanking the AUG start codon is much less stringent than in multicellular eukaryotes.

### Structural differences in the P_IN_ state might explain TIS differential recognition in rabbit and yeast

Structural differences in the ribosome and initiation factors in the P_IN_ conformation between rabbit and yeast potentially explain the differences in the optimal mRNA sequences for translation. The most striking difference between TIS contexts is +4G in rabbit and +4A in yeast. While in rabbit RPS15 (16,17,66) and the 18S nucleotides 1697 and 1820 (15) interact with this position, in yeast only a contact with eIF1A Trp70 was reported (13,14). Importantly, in the P_IN_ conformation the Met-tRNA_i_^Met^ is inserted deeper into the P-site in the yeast ribosome than in rabbit (14). This pushes the eIF2α-Domain 1 (D1) of the ternary complex about 7 Å deeper into the ribosomal E-site compared to the rabbit molecule, allowing interaction between Arg55 and Arg57 of eIF2α-D1 and the −3 and −2 nucleotides of mRNA, respectively (14). In addition, Tyr79 contacting the D stem loop of Met-tRNA_i_^Met^ in rabbit eIF1 (15) is substituted by a methionine in yeast eIF1 (28). Moreover, in rabbit, eIF2α and RPS26e Val83 contact the mRNA −3 position (15); RPS5 contacts the −3 and −4 positions (16,17); and RPS15 carboxy-terminus interacts with the + 4 and + 5 positions (16,17,66). In contrast, in yeast no ribosomal proteins contact the TIS context sequence. Finally, yeast eIF1 Asp71, Glu73, and Glu76 contact the D stem backbone of Met-tRNA_i_^Met^, an interaction not detected in rabbit (14).

### BACS context

We discovered eight conserved sequences in rabbit, fruit fly, and wheat that strongly disfavor translation (Table 2). Six out of them are conserved in yeast. These sequences are also included among the 20% least efficient TIS contexts in the fission yeast *Schizossacharomyces pombe* (data not shown), and might be among the most potent negative regulators across eukaryotes at the level of TIS recognition. These findings are consistent with studies in mouse cells describing that −3C and + 4C are the least efficient bases in the TIS context from −5 to + 4 positions (45), and with the observation that U at position −3 is abundant in poorly expressed genes in yeast (33). Moreover, eIF1 genes, which show a low level of expression, possess −3C/U and U + 4 across eukaryotes (47). Indeed, ribosomal profiles provided evidence that YCMAUGY-containing mRNAs undergo leaky scanning during translation initiation. According with these experimental observations, *in silico* studies showed that −3U/C and −2U/G are universally absent in the consensus TIS context (28).

Among the TIS context sequences that significantly promote translation in vertebrates, Kozak defined two types, namely strong and optimal, based on differences between them (4,5,28). Kozak also defined weak sequences generally as YNNNAUGH (H represents A, C or U). Based on the substantive differences among the weak sequences we observed, we defined a novel category among them that we termed BACS. Thus, although YNNAUGY sequences have been termed ‘Kozak weak’, here we redefine eight specific sequences on a functional basis. Kozak and others analyzed the TIS context to find the sequences that better promote translation. Our analysis presents the first study defining a specific TIS context that disfavors translation. In rabbit, fruit fly, and wheat the BACS-containing genes are enriched in membrane components. Future investigations will address the biological relevance of this observation under different physiological conditions.

## Supplementary Material

gkad1152_supplemental_files

## Data Availability

The data underlying this article are available in the article and in its online supplementary material.
