# Supplementary material for: Functional analysis of the AUG initiator codon context reveals novel conserved sequences that disfavor mRNA translation in eukaryotes
Source: Nucleic Acids Res. 2023 Dec 1;52(3):1064–79. doi: 10.1093/nar/gkad1152 (PMC10853783; doi:10.1093/nar/gkad1152)
Supplement: gkad1152_supplemental_files [file gkad1152_supplemental_files.zip › Suppl. Table 1.docx]

**Suppl. Table 1.** Reagents used in this study.

| **Reagent** | **Company** | **Location** | **Cat. Num.** |
| --- | --- | --- | --- |
| Vector pTZ57R/T, from the insTAclone PCR cloning kit | Fermentas Thermo Scientific | Massachusetts, USA | K1213 |
| Primers | Integrated DNA Technologies (IDT), Inc. | Iowa,  USA | ----- |
| Taq plus precision DNA polymerase | Stratagene | La Jolla,  USA | 600211-51 |
| In vitro transcription mMessage mMachine T7 Kit | Thermo Fisher Scientific | Massachusetts, USA | AM1344 |
| Maxiscript T3 in vitro transcription kit | Invitrogen | California, USA | 8150G |
| RNeasy minielute RNA clean up kit | Qiagen | Hilden, Germany | 74204 |
| QIAquick gel extraction kit | Qiagen | Hilden, Germany | 28704 |
| QIAprep spin plasmid miniprep kit | Qiagen | Hilden, Germany | 27106 |
| EcoRI restriction enzyme | New England Biolabs | Massachusetts, USA | R0101 |
| XhoI restriction enzyme | New England Biolabs | Massachusetts, USA | R0146 |
| Passive lysis buffer 5X | Promega | Wisconsin,  USA | E1910 |
| Non-functional cap analogue G(5´)ppp(5´)A | New England Biolabs | Massachusetts, USA | S1406 |
| Renilla luciferase (RLuc) assay system | Promega | Wisconsin,  USA | E2820 |
| Rabbit reticulocyte lysate (RRL) | Promega | Wisconsin,  USA | L4960 |
| Wheat germ extract | Promega | Wisconsin,  USA | L4380 |
| Luciferase assay system | Promega | Wisconsin,  USA | E1501 |
| RNasin ribonuclease inhibitor | Promega | Wisconsin,  USA | N2111A |
| Amino acid mixture, complete | Promega | Wisconsin,  USA | L4461 |
| m^7^G(5')ppp(5')G RNA cap structure analog | New England Biolabs | Massachusetts, USA | S1404S |
| Creatine phosphoric acid, disodium salt | Merck | Darmstadt, Germany | 112 OA47950 |
| Creatine phosphokinase from rabbit muscle | Merck/Sigma Aldrich | Darmstadt, Germany | C-3755 |
| Ribonucleic acid, transfer from bovine liver | Merck/Sigma Aldrich | Darmstadt, Germany | [9014-25-9](https://www.sigmaaldrich.com/MX/es/search/9014-25-9?focus=products&page=1&perpage=30&sort=relevance&term=9014-25-9&type=cas_number) |
| cOmplete, EDTA-free protease inhibitor cocktail | Roche | Basel, Switzerland | 11836170001 |
| Spermidine | Merck | Darmstadt, Germany | 85558 |
| tRNA (ribonucleic acid, transfer) from bovine liver type XI | Merck/Sigma Aldrich | Darmstadt, Germany | R4752 |
