# Supplementary material for: Functional analysis of the AUG initiator codon context reveals novel conserved sequences that disfavor mRNA translation in eukaryotes
Source: Nucleic Acids Res. 2023 Dec 1;52(3):1064–79. doi: 10.1093/nar/gkad1152 (PMC10853783; doi:10.1093/nar/gkad1152)
Supplement: gkad1152_supplemental_files [file gkad1152_supplemental_files.zip › Suppl. Table 6.docx]

**Supplemental Table 6**

Annotated Gene Ontology (GO) of BACS-containing genes in **rabbit.**

***n* = 196**.

Kozak: TCCATGT ID=cds-XP_008262590.1;Parent=rna-XM_008264368.2;Dbxref=GeneID:100346635,Genbank:XP_008262590.1;Name=XP_008262590.1;gbkey=CDS;gene=KCNN3;product=

Small conductance calcium-activated potassium channel protein 3 isoform.

Kozak: TTAATGC ID=cds-XP_002715325.1;Parent=rna-XM_002715279.1;Dbxref=GeneID:100349334,Genbank:XP_002715325.1;Name=XP_002715325.1;gbkey=CDS;gene=LOC100349334;product=olfactory receptor 10J3-like;protein_id=XP_002715325.1

GO:0016021: Integral component of membrane.

Kozak: TTCATGT ID=cds-XP_017201856.1;Parent=rna-XM_017346367.1;Dbxref=GeneID:100351167,Genbank:XP_017201856.1;Name=XP_017201856.1;gbkey=CDS;gene=SLC6A9;product=sodium- and chloride-dependent glycine transporter 1 isoform X3;protein_id=XP_017201856.1

GO:0016324: Apical plasma membrane.

Kozak: CCAATGT ID=cds-XP_017201368.1;Parent=rna-XM_017345879.1;Dbxref=GeneID:100353789,Genbank:XP_017201368.1;Name=XP_017201368.1;gbkey=CDS;gene=LINGO4;product=leucine-rich repeat and immunoglobulin-like domain-containing nogo receptor-interacting protein 4;protein_id=XP_017201368.

GO:0016021: Integral component of membrane.

Kozak: TGCATGC ID=cds-XP_008264210.1;Parent=rna-XM_008265988.1;Dbxref=GeneID:100353939,Genbank:XP_008264210.1;Name=XP_008264210.1;gbkey=CDS;gene=NOL9;product=

Polynucleotide 5'-hydroxyl-kinase NOL9 isoform X2.

Kozak: TGCATGC ID=cds-XP_008263834.1;Parent=rna-XM_008265612.2;Dbxref=GeneID:100355386,Genbank:XP_008263834.1;Name=XP_008263834.1;gbkey=CDS;gene=COL24A1;product=collagen alpha-1(XXIV) chain;protein_id=XP_008263834.1

GO:0005201: Extracellular matrix structural constituent.

Kozak: TGGATGC ID=cds-XP_008263737.1;Parent=rna-XM_008265515.2;Dbxref=GeneID:103350213,Genbank:XP_008263737.1;Name=XP_008263737.1;gbkey=CDS;gene=LOC103350213;product=bone morphogenetic protein 8A;protein_id=XP_008263737.1

GO:0005576: Extracellular region.

Kozak: TGTATGC ID=cds-XP_002715160.1;Parent=rna-XM_002715114.3;Dbxref=GeneID:100339994,Genbank:XP_002715160.1;Name=XP_002715160.1;gbkey=CDS;gene=CMPK1;product=UMP-CMP kinase;protein_id=XP_002715160.1

GO:0005737; Cytoplasm.

Kozak: CCCATGT ID=cds-XP_017201978.1;Parent=rna-XM_017346489.1;Dbxref=GeneID:100341109,Genbank:XP_017201978.1;Name=XP_017201978.1;Note=The sequence of the model RefSeq protein was modified relative to this genomic sequence to represent the inferred CDS: added 133 bases not found in genome assembly;exception=annotated by transcript or proteomic data;gbkey=CDS;gene=ALPL;inference=similar to RNA sequence (same species):INSD:GBCD01053290.1;partial=true;product=

Alkaline phosphatase 2C tissue-nonspecific isozyme.

Kozak: TGTATGT ID=cds-XP_002716028.1;Parent=rna-XM_002715982.3;Dbxref=GeneID:100345346,Genbank:XP_002716028.1;Name=XP_002716028.1;gbkey=CDS;gene=LYPLA2;product=acyl-protein thioesterase 2;protein_id=XP_002716028.1

GO:0016787: Hydrolase activity.

Kozak: TGGATGC ID=cds-XP_008264128.2;Parent=rna-XM_008265906.2;Dbxref=GeneID:100345347,Genbank:XP_008264128.2;Name=XP_008264128.2;Note=The sequence of the model RefSeq protein was modified relative to this genomic sequence to represent the inferred CDS: inserted 1 base in 1 codon;exception=unclassified translation discrepancy;gbkey=CDS;gene=SH3BGRL3;product=LOW QUALITY PROTEIN: SH3 domain-binding glutamic acid-rich-like protein 3;protein_id=XP_008264128.2

GO:0016604: Nuclear body.

Kozak: TGCATGC ID=cds-XP_008263573.1;Parent=rna-XM_008265351.2;Dbxref=GeneID:100347910,Genbank:XP_008263573.1;Name=XP_008263573.1;gbkey=CDS;gene=UROD;product=uroporphyrinogen decarboxylase isoform X2;protein_id=XP_008263573.1

GO:0005829; Cytosol.

Kozak: TGCATGC ID=cds-XP_008263458.1;Parent=rna-XM_008265236.2;Dbxref=GeneID:100348157,Genbank:XP_008263458.1;Name=XP_008263458.1;gbkey=CDS;gene=ZYG11B;product=protein zyg-11 homolog B;protein_id=XP_008263458.1

GO:0031462; Cul2-RING ubiquitin ligase complex.

Kozak: TGCATGC ID=cds-XP_002715887.1;Parent=rna-XM_002715841.3;Dbxref=GeneID:100349006,Genbank:XP_002715887.1;Name=XP_002715887.1;gbkey=CDS;gene=PLPPR4;product=phospholipid phosphatase-related protein type 4 isoform X1;protein_id=XP_002715887.1

GO:0098978; Glutamatergic synapse.

Kozak: CCCATGT ID=cds-XP_017201875.1;Parent=rna-XM_017346386.1;Dbxref=GeneID:100349416,Genbank:XP_017201875.1;Name=XP_017201875.1;gbkey=CDS;gene=SZT2;product=protein SZT2 isoform X2;protein_id=XP_017201875.1

GO:1990130; GATOR1 complex.

Kozak: CCCATGT ID=cds-XP_008264200.1;Parent=rna-XM_008265978.2;Dbxref=GeneID:100349420,Genbank:XP_008264200.1;Name=XP_008264200.1;gbkey=CDS;gene=CAMTA1;product=calmodulin-binding transcription activator 1 isoform X2;protein_id=XP_008264200.1

GO:0003677; DNA binding.

Kozak: TTCATGT ID=cds-XP_008263881.1;Parent=rna-XM_008265659.2;Dbxref=GeneID:100355635,Genbank:XP_008263881.1;Name=XP_008263881.1;gbkey=CDS;gene=CLIC4;product=chloride intracellular channel protein 4;protein_id=XP_008263881.1

GO:0034707; Chloride channel complex.

Kozak: TCCATGT ID=cds-NP_001164920.1;Parent=rna-NM_001171449.1;Dbxref=GeneID:100328851,Genbank:NP_001164920.1;Name=NP_001164920.1;gbkey=CDS;gene=OLR108;product=

Olfactory receptor Olr108.

Kozak: CCCATGT ID=cds-XP_002708069.1;Parent=rna-XM_002708023.3;Dbxref=GeneID:100342932,Genbank:XP_002708069.1;Name=XP_002708069.1;gbkey=CDS;gene=KIAA1161;product=uncharacterized family 31 glucosidase KIAA1161 homolog;protein_id=XP_002708069.1

GO:0016021; Integral component of membrane.

Kozak: TGGATGC ID=cds-XP_002708307.1;Parent=rna-XM_002708261.3;Dbxref=GeneID:100345297,Genbank:XP_002708307.1;Name=XP_002708307.1;gbkey=CDS;gene=NFIL3;product=nuclear factor interleukin-3-regulated protein;protein_id=XP_002708307.1

GO:0090575; RNA polymerase II transcription regulator complex.

Kozak: CCAATGT ID=cds-XP_017203432.1;Parent=rna-XM_017347943.1;Dbxref=GeneID:100352054,Genbank:XP_017203432.1;Name=XP_017203432.1;gbkey=CDS;gene=THAP12;product=52 kDa repressor of the inhibitor of the protein kinase;protein_id=XP_017203432.1

GO:0003677; DNA binding.

Kozak: CCGATGT ID=cds-XP_002709060.1;Parent=rna-XM_002709014.3;Dbxref=GeneID:100353901,Genbank:XP_002709060.1;Name=XP_002709060.1;gbkey=CDS;gene=MS4A15;product=membrane-spanning 4-domains subfamily A member 15 isoform X1;protein_id=XP_002709060.1

GO:0016021; Integral component of membrane.

Kozak: TGCATGC ID=cds-XP_017197643.1;Parent=rna-XM_017342154.1;Dbxref=GeneID:100358576,Genbank:XP_017197643.1;Name=XP_017197643.1;gbkey=CDS;gene=NAA35;product=N-alpha-acetyltransferase 35%2C NatC auxiliary subunit isoform X1;protein_id=XP_017197643.1

GO:0005829; Cytosol.

Kozak: CCGATGT ID=cds-XP_002708004.1;Parent=rna-XM_002707958.3;Dbxref=GeneID:100359004,Genbank:XP_002708004.1;Name=XP_002708004.1;gbkey=CDS;gene=TUSC1;product=

Tumor suppressor candidate gene 1 protein.

Kozak: TACATGC ID=cds-XP_017203050.1;Parent=rna-XM_017347561.1;Dbxref=GeneID:103349066,Genbank:XP_017203050.1;Name=XP_017203050.1;gbkey=CDS;gene=LOC103349066;product=

Rho GTPase-activating protein 20.

Kozak: TGCATGC ID=cds-XP_017201545.1;Parent=rna-XM_017346056.1;Dbxref=GeneID:103351337,Genbank:XP_017201545.1;Name=XP_017201545.1;gbkey=CDS;gene=LOC103351337;product=protein FAM170A;protein_id=XP_017201545.1

GO:0005634; Nucleus.

Kozak: CCAATGT ID=cds-XP_002708588.1;Parent=rna-XM_002708542.2;Dbxref=GeneID:100345050,Genbank:XP_002708588.1;Name=XP_002708588.1;gbkey=CDS;gene=LOC100345050;product=olfactory receptor 52K1-like;protein_id=XP_002708588.1

GO:0016021; Integral component of membrane.

Kozak: CCAATGT ID=cds-XP_008267829.1;Parent=rna-XM_008269607.2;Dbxref=GeneID:100349057,Genbank:XP_008267829.1;Name=XP_008267829.1;gbkey=CDS;gene=LMO2;product=rhombotin-2;protein_id=XP_008267829.1

GO:0005667; Transcription regulator complex.

Kozak: TGGATGC ID=cds-XP_017195053.1;Parent=rna-XM_017339564.1;Dbxref=GeneID:100343380,Genbank:XP_017195053.1;Name=XP_017195053.1;gbkey=CDS;gene=CHST10;product=carbohydrate sulfotransferase 10 isoform X1;protein_id=XP_017195053.1

GO:0000139; Golgi membrane.

Kozak: CCCATGT ID=cds-XP_008251514.2;Parent=rna-XM_008253292.2;Dbxref=GeneID:100345061,Genbank:XP_008251514.2;Name=XP_008251514.2;gbkey=CDS;gene=RNF149;product=E3 ubiquitin-protein ligase RNF149;protein_id=XP_008251514.2

GO:0016021; Integral component of membrane.

Kozak: CCCATGT ID=cds-XP_008272966.1;Parent=rna-XM_008274744.2;Dbxref=GeneID:100350229,Genbank:XP_008272966.1;Name=XP_008272966.1;gbkey=CDS;gene=ANAPC4;product=anaphase-promoting complex subunit 4 isoform X1;protein_id=XP_008272966.1

GO:0005680; Anaphase-promoting complex.

Kozak: TGGATGC ID=cds-XP_017196384.1;Parent=rna-XM_017340895.1;Dbxref=GeneID:100355023,Genbank:XP_017196384.1;Name=XP_017196384.1;gbkey=CDS;gene=MYCN;product=

N-myc proto-oncogene protein.

Kozak: TCCATGT ID=cds-XP_017195018.1;Parent=rna-XM_017339529.1;Dbxref=GeneID:100358766,Genbank:XP_017195018.1;Name=XP_017195018.1;gbkey=CDS;gene=TBC1D8;product=TBC1 domain family member 8 isoform X3;protein_id=XP_017195018.1

GO:0005096; GTPase activator activity.

Kozak: CCCATGT ID=cds-XP_008252538.1;Parent=rna-XM_008254316.2;Dbxref=GeneID:100343033,Genbank:XP_008252538.1;Name=XP_008252538.1;gbkey=CDS;gene=PLEK;product=pleckstrin isoform X2;protein_id=XP_008252538.1

GO:0035556; Intracellular signal transduction.

Kozak: TGGATGC ID=cds-XP_008272817.1;Parent=rna-XM_008274595.2;Dbxref=GeneID:100344208,Genbank:XP_008272817.1;Name=XP_008272817.1;Note=The sequence of the model RefSeq protein was modified relative to this genomic sequence to represent the inferred CDS: inserted 1 base in 1 codon;exception=unclassified translation discrepancy;gbkey=CDS;gene=ADGRA3;product=LOW QUALITY PROTEIN: adhesion G protein-coupled receptor A3;protein_id=XP_008272817.1

GO:0016021; Integral component of membrane.

Kozak: CCCATGT ID=cds-XP_002709632.1;Parent=rna-XM_002709586.3;Dbxref=GeneID:100348299,Genbank:XP_002709632.1;Name=XP_002709632.1;gbkey=CDS;gene=ANAPC1;product=anaphase-promoting complex subunit 1 isoform X1;protein_id=XP_002709632.1

GO:0005680; Anaphase-promoting complex.

Kozak: CCCATGT ID=cds-XP_008272379.1;Parent=rna-XM_008274157.2;Dbxref=GeneID:100348808,Genbank:XP_008272379.1;Name=XP_008272379.1;gbkey=CDS;gene=SLC2A9;product=solute carrier family 2%2C facilitated glucose transporter member 9 isoform X2;protein_id=XP_008272379.1

GO:0016324; Apical plasma membrane.

Kozak: TCCATGT ID=cds-XP_008252023.1;Parent=rna-XM_008253801.2;Dbxref=GeneID:100351730,Genbank:XP_008252023.1;Name=XP_008252023.1;gbkey=CDS;gene=NCAPH;product=condensin complex subunit 2 isoform X3;protein_id=XP_008252023.1

GO:0000796; Condensin complex.

Kozak: TACATGC ID=cds-XP_017194960.1;Parent=rna-XM_017339471.1;Dbxref=GeneID:100355438,Genbank:XP_017194960.1;Name=XP_017194960.1;gbkey=CDS;gene=MAP4K4;product=

Mitogen-activated protein kinase kinase kinase kinase 4 isoform X2.

Kozak: CCCATGT ID=cds-XP_008252624.1;Parent=rna-XM_008254402.2;Dbxref=GeneID:103347681,Genbank:XP_008252624.1;Name=XP_008252624.1;gbkey=CDS;gene=ACYP2;product=acylphosphatase-2 isoform X2;protein_id=XP_008252624.1

GO:0003998; Acylphosphatase activity.

Kozak: TGTATGT ID=cds-XP_002712489.1;Parent=rna-XM_002712443.3;Dbxref=GeneID:100340828,Genbank:XP_002712489.1;Name=XP_002712489.1;gbkey=CDS;gene=NIF3L1;product=NIF3-like protein 1 isoform X1;protein_id=XP_002712489.1

GO:0005737; Cytoplasm.

Kozak: CCCATGT ID=cds-XP_002712420.1;Parent=rna-XM_002712374.3;Dbxref=GeneID:100351659,Genbank:XP_002712420.1;Name=XP_002712420.1;gbkey=CDS;gene=C7H2orf69;product=UPF0565 protein C2orf69 homolog;protein_id=XP_002712420.1

GO:0005739; Mitochondrion.

Kozak: CCCATGT ID=cds-NP_001164878.1;Parent=rna-NM_001171407.1;Dbxref=GeneID:100328794,Genbank:NP_001164878.1;Name=NP_001164878.1;gbkey=CDS;gene=MDFIC;product=myoD family inhibitor domain-containing protein;protein_id=NP_001164878.1

GO:0030332; Cyclin binding.

Kozak: CCAATGT ID=cds-XP_017198671.1;Parent=rna-XM_017343182.1;Dbxref=GeneID:100341427,Genbank:XP_017198671.1;Name=XP_017198671.1;gbkey=CDS;gene=TRIP12;product=E3 ubiquitin-protein ligase TRIP12;protein_id=XP_017198671.1

GO:0005654; Nucleoplasm.

Kozak: TTCATGT ID=cds-XP_002712167.3;Parent=rna-XM_002712121.3;Dbxref=GeneID:100343646,Genbank:XP_002712167.3;Name=XP_002712167.3;gbkey=CDS;gene=LRP1B;product=low-density lipoprotein receptor-related protein 1B;protein_id=XP_002712167.3

GO:0016021; Integral component of membrane.

Kozak: TTAATGC ID=cds-XP_017198456.1;Parent=rna-XM_017342967.1;Dbxref=GeneID:100354780,Genbank:XP_017198456.1;Name=XP_017198456.1;gbkey=CDS;gene=PRKRA;product=interferon-inducible double-stranded RNA-dependent protein kinase activator A isoform X2;protein_id=XP_017198456.1 GO:0005829; Cytosol.

Kozak: TCCATGT ID=cds-XP_002716694.3;Parent=rna-XM_002716648.3;Dbxref=GeneID:100344748,Genbank:XP_002716694.3;Name=XP_002716694.3;gbkey=CDS;gene=RABL3;product=rab-like protein 3;protein_id=XP_002716694.3

GO:0004411; Homogentisate 1,2-dioxygenase activity.

Kozak: CCCATGT ID=cds-XP_017202427.1;Parent=rna-XM_017346938.1;Dbxref=GeneID:100353793,Genbank:XP_017202427.1;Name=XP_017202427.1;gbkey=CDS;gene=TRA2B;product=transformer-2 protein homolog beta isoform X2;protein_id=XP_017202427.1

GO:0003723; RNA binding.

Kozak: TGGATGC ID=cds-XP_017202315.1;Parent=rna-XM_017346826.1;Dbxref=GeneID:100341366,Genbank:XP_017202315.1;Name=XP_017202315.1;gbkey=CDS;gene=LOC100341366;product=phospholipid scramblase 1 isoform X3;protein_id=XP_017202315.1

GO:0017128; Phospholipid scramblase activity.

Kozak: TGTATGT ID=cds-XP_002716653.1;Parent=rna-XM_002716607.3;Dbxref=GeneID:100351020,Genbank:XP_002716653.1;Name=XP_002716653.1;gbkey=CDS;gene=TRMT10C;product=mitochondrial ribonuclease P protein 1;protein_id=XP_002716653.1

GO:0042645; Mitochondrial nucleoid.

Kozak: CCCATGT ID=cds-XP_017199554.1;Parent=rna-XM_017344065.1;Dbxref=GeneID:100351663,Genbank:XP_017199554.1;Name=XP_017199554.1;gbkey=CDS;gene=GREB1L;product=GREB1-like protein;protein_id=XP_017199554.1

GO:0016021; Integral component of membrane.

Kozak: TTCATGT ID=cds-XP_002713308.1;Parent=rna-XM_002713262.3;Dbxref=GeneID:100353603,Genbank:XP_002713308.1;Name=XP_002713308.1;gbkey=CDS;gene=PDE12;product=2'%2C5'-phosphodiesterase 12;protein_id=XP_002713308.1

GO:0005759; Mitochondrial matrix.

Kozak: TACATGC ID=cds-XP_008259657.1;Parent=rna-XM_008261435.2;Dbxref=GeneID:100358526,Genbank:XP_008259657.1;Name=XP_008259657.1;gbkey=CDS;gene=ZCCHC2;product=zinc finger CCHC domain-containing protein 2 isoform X1;protein_id=XP_008259657.1

GO:0005737; Cytoplasm.

Kozak: TTCATGT ID=cds-XP_017199500.1;Parent=rna-XM_017344011.1;Dbxref=GeneID:103349294,Genbank:XP_017199500.1;Name=XP_017199500.1;gbkey=CDS;gene=LOC103349294;product=ankyrin repeat domain-containing protein 12-like;protein_id=XP_017199500.1

GO:0005829; Cytosol.

Kozak: TCCATGT ID=cds-XP_017199212.1;Parent=rna-XM_017343723.1;Dbxref=GeneID:100341689,Genbank:XP_017199212.1;Name=XP_017199212.1;Note=The sequence of the model RefSeq protein was modified relative to this genomic sequence to represent the inferred CDS: inserted 1 base in 1 codon;exception=unclassified translation discrepancy;gbkey=CDS;gene=ZNF197;product=LOW QUALITY PROTEIN: zinc finger protein 197;protein_id=XP_017199212.1

GO:0005634; Nucleus.

Kozak: TTAATGC ID=cds-XP_008259556.1;Parent=rna-XM_008261334.2;Dbxref=GeneID:100346796,Genbank:XP_008259556.1;Name=XP_008259556.1;gbkey=CDS;gene=CCDC68;product=coiled-coil domain-containing protein 68;protein_id=XP_008259556.1

GO:0120103; Centriolar subdistal appendage.

Kozak: CCGATGT ID=cds-XP_008259620.1;Parent=rna-XM_008261398.2;Dbxref=GeneID:100349405,Genbank:XP_008259620.1;Name=XP_008259620.1;Note=The sequence of the model RefSeq protein was modified relative to this genomic sequence to represent the inferred CDS: substituted 1 base at 1 genomic stop codon%3B added 87 bases not found in genome assembly;exception=annotated by transcript or proteomic data;gbkey=CDS;gene=NARS;inference=similar to RNA sequence (same species):INSD:GBAX01029137.1;partial=true;product=LOW QUALITY PROTEIN: Asparagine--tRNA ligase2C.

Kozak: TGCATGC ID=cds-XP_017199458.1;Parent=rna-XM_017343969.1;Dbxref=GeneID:100353349,Genbank:XP_017199458.1;Name=XP_017199458.1;gbkey=CDS;gene=LOC100353349;product=E3 ubiquitin-protein ligase PDZRN3;protein_id=XP_017199458.1

GO:0008270; Zinc ion binding.

Kozak: TCCATGT ID=cds-XP_008259077.1;Parent=rna-XM_008260855.2;Dbxref=GeneID:100354864,Genbank:XP_008259077.1;Name=XP_008259077.1;gbkey=CDS;gene=FAM107A;product=protein FAM107A isoform X3;protein_id=XP_008259077.1

GO:0005737; Cytoplasm.

Kozak: TGGATGC ID=cds-NP_001075638.1;Parent=rna-NM_001082169.1;Dbxref=GeneID:100008931,Genbank:NP_001075638.1;Name=NP_001075638.1;Note=The RefSeq protein has 1 substitution compared to this genomic sequence;exception=annotated by transcript or proteomic data;gbkey=CDS;gene=SLC22A3;inference=similar to AA sequence (same species):RefSeq:NP_001075638.1;product=solute carrier family 22 member 3;protein_id=NP_001075638.1

GO:0016021; Integral component of membrane.

Kozak: CCGATGT ID=cds-NP_001164809.1;Parent=rna-NM_001171338.1;Dbxref=GeneID:100328713,Genbank:NP_001164809.1;Name=NP_001164809.1;gbkey=CDS;gene=MDFI;product=

MyoD family inhibitor.

Kozak: CCGATGT ID=cds-XP_008261759.1;Parent=rna-XM_008263537.2;Dbxref=GeneID:100357328,Genbank:XP_008261759.1;Name=XP_008261759.1;Note=The sequence of the model RefSeq protein was modified relative to this genomic sequence to represent the inferred CDS: deleted 1 base in 1 codon;exception=unclassified translation discrepancy;gbkey=CDS;gene=EYA4;product=LOW QUALITY PROTEIN: eyes absent homolog 4;protein_id=XP_008261759.1

GO:0005737; Cytoplasm.

Kozak: TACATGC ID=cds-XP_017200967.1;Parent=rna-XM_017345478.1;Dbxref=GeneID:108177497,Genbank:XP_017200967.1;Name=XP_017200967.1;Note=The sequence of the model RefSeq protein was modified relative to this genomic sequence to represent the inferred CDS: added 188 bases not found in genome assembly;end_range=152550943,.;exception=annotated by transcript or proteomic data;gbkey=CDS;gene=LOC108177497;inference=similar to RNA sequence (same species):INSD:GBCI01111389.1;partial=true;product=

Collagen alpha-1(I) chain.

Kozak: CGAATGT ID=cds-XP_008261128.2;Parent=rna-XM_008262906.2;Dbxref=GeneID:100348748,Genbank:XP_008261128.2;Name=XP_008261128.2;gbkey=CDS;gene=LOC100348748;product=

Triggering receptor expressed on myeloid cells 2.

Kozak: TGGATGC ID=cds-XP_002714381.2;Parent=rna-XM_002714335.3;Dbxref=GeneID:100352008,Genbank:XP_002714381.2;Name=XP_002714381.2;gbkey=CDS;gene=UBD;product=ubiquitin D;protein_id=XP_002714381.2 GO; GO:0016235; Aggresome.

Kozak: TCCATGT ID=cds-XP_008261593.1;Parent=rna-XM_008263371.2;Dbxref=GeneID:100355706,Genbank:XP_008261593.1;Name=XP_008261593.1;gbkey=CDS;gene=CDK19;product=cyclin-dependent kinase 19 isoform X2;protein_id=XP_008261593.1

GO:0005829; Cytosol.

Kozak: TCCATGT ID=cds-XP_008261777.2;Parent=rna-XM_008263555.2;Dbxref=GeneID:103349891,Genbank:XP_008261777.2;Name=XP_008261777.2;gbkey=CDS;gene=MTFR2;product=mitochondrial fission regulator 2 isoform X2;protein_id=XP_008261777.2

GO:0005739; Mitochondrion.

Kozak: TCCATGT ID=cds-XP_008269144.2;Parent=rna-XM_008270922.2;Dbxref=GeneID:100341205,Genbank:XP_008269144.2;Name=XP_008269144.2;gbkey=CDS;gene=RTN4RL1;product=reticulon-4 receptor-like 1;protein_id=XP_008269144.2

GO:0046658; Anchored component of plasma membrane.

Kozak: TGGATGC ID=cds-XP_008269717.1;Parent=rna-XM_008271495.2;Dbxref=GeneID:100343163,Genbank:XP_008269717.1;Name=XP_008269717.1;gbkey=CDS;gene=HSPB9;product=heat shock protein beta-9;protein_id=XP_008269717.1

GO:0005829; Cytosol.

Kozak: TTCATGT ID=cds-XP_008269370.1;Parent=rna-XM_008271148.2;Dbxref=GeneID:100355725,Genbank:XP_008269370.1;Name=XP_008269370.1;gbkey=CDS;gene=SYNRG;product=

Synergin gamma isoform X1.

Kozak: TACATGC ID=cds-XP_008270031.1;Parent=rna-XM_008271809.2;Dbxref=GeneID:100356834,Genbank:XP_008270031.1;Name=XP_008270031.1;gbkey=CDS;gene=LOC100356834;product=

Leucine-rich repeat-containing protein 37A3.

Kozak: TGGATGC ID=cds-XP_017204317.1;Parent=rna-XM_017348828.1;Dbxref=GeneID:100343083,Genbank:XP_017204317.1;Name=XP_017204317.1;Note=The sequence of the model RefSeq protein was modified relative to this genomic sequence to represent the inferred CDS: inserted 1 base in 1 codon%3B deleted 1 base in 1 codon;exception=unclassified translation discrepancy;gbkey=CDS;gene=KDM6B;product=LOW QUALITY PROTEIN: lysine-specific demethylase 6B;protein_id=XP_017204317.1

GO:0005634; Nucleus.

Kozak: TGGATGC ID=cds-XP_002718189.1;Parent=rna-XM_002718143.4;Dbxref=GeneID:100009021,Genbank:XP_002718189.1;Name=XP_002718189.1;Note=The sequence of the model RefSeq protein was modified relative to this genomic sequence to represent the inferred CDS: inserted 1 base in 1 codon%3B deleted 1 base in 1 codon;exception=unclassified translation discrepancy;gbkey=CDS;gene=PPIB;product=LOW QUALITY PROTEIN:

Peptidyl-prolyl cis-trans isomerase B.

Kozak: CGAATGT ID=cds-XP_008267980.1;Parent=rna-XM_008269758.2;Dbxref=GeneID:100342825,Genbank:XP_008267980.1;Name=XP_008267980.1;gbkey=CDS;gene=UBE3A;product=ubiquitin-protein ligase E3A isoform X2;protein_id=XP_008267980.1

GO:0005829; Cytosol.

Kozak: CCAATGT ID=cds-XP_002718067.1;Parent=rna-XM_002718021.2;Dbxref=GeneID:100345870,Genbank:XP_002718067.1;Name=XP_002718067.1;gbkey=CDS;gene=LOC100345870;product=olfactory receptor 4F15-like;protein_id=XP_002718067.1

GO:0016021; Integral component of membrane.

Kozak: TGCATGC ID=cds-XP_017203506.1;Parent=rna-XM_017348017.1;Dbxref=GeneID:100348259,Genbank:XP_017203506.1;Name=XP_017203506.1;gbkey=CDS;gene=SPPL2A;

Signal peptide peptidase-like 2A.

Kozak: TCCATGT ID=cds-XP_017203741.1;Parent=rna-XM_017348252.1;Dbxref=GeneID:100353954,Genbank:XP_017203741.1;Name=XP_017203741.1;gbkey=CDS;gene=AKAP6;product=A-kinase anchor protein 6 isoform X1;protein_id=XP_017203741.1

GO:0034704; Calcium channel complex.

Kozak: CCAATGT ID=cds-XP_017203857.1;Parent=rna-XM_017348368.1;Dbxref=GeneID:108178332,Genbank:XP_017203857.1;Name=XP_017203857.1;Note=The sequence of the model RefSeq protein was modified relative to this genomic sequence to represent the inferred CDS: inserted 1 base in 1 codon;exception=unclassified translation discrepancy;gbkey=CDS;gene=LOC108178332;product=LOW QUALITY PROTEIN: Translation initiation factor IF-2-like.

Kozak: TGCATGC ID=cds-XP_002717831.1;Parent=rna-XM_002717785.3;Dbxref=GeneID:100344591,Genbank:XP_002717831.1;Name=XP_002717831.1;gbkey=CDS;gene=CHAC1;product=glutathione-specific gamma-glutamylcyclotransferase 1;protein_id=XP_002717831.1

GO:0003839; Gamma-glutamylcyclotransferase activity.

Kozak: TGCATGC ID=cds-XP_008267393.1;Parent=rna-XM_008269171.2;Dbxref=GeneID:100347265,Genbank:XP_008267393.1;Name=XP_008267393.1;gbkey=CDS;gene=RTF1;product=RNA polymerase-associated protein RTF1 homolog isoform X2;protein_id=XP_008267393.1

GO:0016593; Cdc73/Paf1 complex.

Kozak: TGTATGC ID=cds-XP_008267452.1;Parent=rna-XM_008269230.2;Dbxref=GeneID:100351685,Genbank:XP_008267452.1;Name=XP_008267452.1;gbkey=CDS;gene=SPRED1;product=sprouty-related%2C EVH1 domain-containing protein 1 isoform X2;protein_id=XP_008267452.1

GO:0016020; Membrane.

Kozak: CCAATGT ID=cds-XP_017203464.1;Parent=rna-XM_017347975.1;Dbxref=GeneID:100355644,Genbank:XP_017203464.1;Name=XP_017203464.1;Note=The sequence of the model RefSeq protein was modified relative to this genomic sequence to represent the inferred CDS: added 521 bases not found in genome assembly;end_range=10199885,.;exception=annotated by transcript or proteomic data;gbkey=CDS;gene=LOC100355644;inference=similar to RNA sequence (same species):INSD:GBCA01136047.1;partial=true;product=

C2 calcium-dependent domain-containing protein 4A.

Kozak: CCCATGT ID=cds-XP_008254240.1;Parent=rna-XM_008256018.2;Dbxref=GeneID:100352911,Genbank:XP_008254240.1;Name=XP_008254240.1;Note=The sequence of the model RefSeq protein was modified relative to this genomic sequence to represent the inferred CDS: added 146 bases not found in genome assembly;exception=annotated by transcript or proteomic data;gbkey=CDS;gene=MROH8;inference=similar to RNA sequence (same species):INSD:GBCK01099120.1;partial=true;product=

Protein MROH8.

Kozak: TGGATGC ID=cds-XP_008254456.2;Parent=rna-XM_008256234.2;Dbxref=GeneID:100353007,Genbank:XP_008254456.2;Name=XP_008254456.2;Note=The sequence of the model RefSeq protein was modified relative to this genomic sequence to represent the inferred CDS: added 74 bases not found in genome assembly;exception=annotated by transcript or proteomic data;gbkey=CDS;gene=EBF4;inference=similar to RNA sequence (same species):INSD:GBCT01063057.1;partial=true;product=

Transcription factor COE4.

Kozak: TGGATGC ID=cds-XP_008255033.1;Parent=rna-XM_008256811.2;Dbxref=GeneID:100355031,Genbank:XP_008255033.1;Name=XP_008255033.1;gbkey=CDS;gene=GLIPR1L1;product=

GLIPR1-like protein 1 isoform X2.

Kozak: TCCATGT ID=cds-XP_008255034.1;Parent=rna-XM_008256812.2;Dbxref=GeneID:100355280,Genbank:XP_008255034.1;Name=XP_008255034.1;gbkey=CDS;gene=LOC100355280;product=GLIPR1-like protein 1 isoform X1;protein_id=XP_008255034.1

GO:0005576; Extracellular region.

Kozak: TCCATGT ID=cds-XP_002711306.2;Parent=rna-XM_002711260.3;Dbxref=GeneID:100355530,Genbank:XP_002711306.2;Name=XP_002711306.2;gbkey=CDS;gene=LOC100355530;product=

GLIPR1-like protein 1.

Kozak: TGCATGC ID=cds-XP_017197414.1;Parent=rna-XM_017341925.1;Dbxref=GeneID:100342436,Genbank:XP_017197414.1;Name=XP_017197414.1;gbkey=CDS;gene=PYM1;product=partner of Y14 and mago isoform X1;protein_id=XP_017197414.1

GO:0005737; Cytoplasm.

Kozak: TCCATGT ID=cds-XP_017197191.1;Parent=rna-XM_017341702.1;Dbxref=GeneID:103348206,Genbank:XP_017197191.1;Name=XP_017197191.1;gbkey=CDS;gene=LOC103348206;product=

Translation initiation factor IF-2.

Kozak: TTCATGT ID=cds-XP_008253404.2;Parent=rna-XM_008255182.2;Dbxref=GeneID:100338557,Genbank:XP_008253404.2;Name=XP_008253404.2;gbkey=CDS;gene=TCERG1;product=transcription elongation regulator 1 isoform X1;protein_id=XP_008253404.2

GO:0070063; RNA polymerase binding.

Kozak: CCAATGT ID=cds-XP_008253791.1;Parent=rna-XM_008255569.2;Dbxref=GeneID:100350319,Genbank:XP_008253791.1;Name=XP_008253791.1;gbkey=CDS;gene=YTHDF3;product=YTH domain-containing family protein 3 isoform X5;protein_id=XP_008253791.1

GO:0003723; RNA binding.

Kozak: TGTATGT ID=cds-XP_008253754.1;Parent=rna-XM_008255532.2;Dbxref=GeneID:100125992,Genbank:XP_008253754.1;Name=XP_008253754.1;gbkey=CDS;gene=LYPLA1;product=acyl-protein thioesterase 1;protein_id=XP_008253754.1

GO:0005634; Nucleus.

Kozak: TCCATGT ID=cds-XP_017196869.1;Parent=rna-XM_017341380.1;Dbxref=GeneID:100345151,Genbank:XP_017196869.1;Name=XP_017196869.1;Note=The sequence of the model RefSeq protein was modified relative to this genomic sequence to represent the inferred CDS: deleted 2 bases in 2 codons%3B added 32 bases not found in genome assembly;exception=annotated by transcript or proteomic data;gbkey=CDS;gene=ARFGEF1;inference=similar to RNA sequence (same species):INSD:GBAX01068613.1;partial=true;product=LOW QUALITY PROTEIN:

Brefeldin A-inhibited guanine nucleotide-exchange protein 1.

Kozak: CCCATGT ID=cds-XP_008253542.1;Parent=rna-XM_008255320.2;Dbxref=GeneID:100350151,Genbank:XP_008253542.1;Name=XP_008253542.1;gbkey=CDS;gene=HAVCR2;product=hepatitis A virus cellular receptor 2;protein_id=XP_008253542.1

GO:0016021; Integral component of membrane.

Kozak: TCCATGT ID=cds-XP_008253936.1;Parent=rna-XM_008255714.2;Dbxref=GeneID:100352398,Genbank:XP_008253936.1;Name=XP_008253936.1;gbkey=CDS;gene=NBN;product=nibrin isoform X1;protein_id=XP_008253936.1

GO:0000781; Chromosome, telomeric region.

Kozak: TTCATGT ID=cds-XP_002710389.1;Parent=rna-XM_002710343.3;Dbxref=GeneID:100354013,Genbank:XP_002710389.1;Name=XP_002710389.1;gbkey=CDS;gene=EBF1;product=transcription factor COE1 isoform X3;protein_id=XP_002710389.1

GO:0005634; Nucleus.

Kozak: CCAATGT ID=cds-XP_017197009.1;Parent=rna-XM_017341520.1;Dbxref=GeneID:100357469,Genbank:XP_017197009.1;Name=XP_017197009.1;gbkey=CDS;gene=TATDN1;product=putative deoxyribonuclease TATDN1 isoform X3;protein_id=XP_017197009.1

GO:0005654; Nucleoplasm.

Kozak: CCAATGT ID=cds-XP_008268078.1;Parent=rna-XM_008269856.2;Dbxref=GeneID:100341201,Genbank:XP_008268078.1;Name=XP_008268078.1;gbkey=CDS;gene=ZFAND4;product=AN1-type zinc finger protein 4 isoform X1;protein_id=XP_008268078.1

GO:0008270; Zinc ion binding.

Kozak: CCGATGT ID=cds-XP_017203909.1;Parent=rna-XM_017348420.1;Dbxref=GeneID:100342397,Genbank:XP_017203909.1;Name=XP_017203909.1;gbkey=CDS;gene=EXOC6;product=

Exocyst complex component 6 isoform X1

Kozak: TGCATGC ID=cds-XP_008268231.1;Parent=rna-XM_008270009.2;Dbxref=GeneID:100343161,Genbank:XP_008268231.1;Name=XP_008268231.1;gbkey=CDS;gene=LRRC20;product=

Leucine-rich repeat-containing protein 20 isoform X1.

Kozak: CCCATGT ID=cds-XP_002718546.1;Parent=rna-XM_002718500.3;Dbxref=GeneID:100339088,Genbank:XP_002718546.1;Name=XP_002718546.1;gbkey=CDS;gene=PANK1;product=pantothenate kinase 1 isoform X1;protein_id=XP_002718546.1

GO:0005737; Cytoplasm.

Kozak: TGGATGC ID=cds-XP_008268695.1;Parent=rna-XM_008270473.2;Dbxref=GeneID:100344851,Genbank:XP_008268695.1;Name=XP_008268695.1;gbkey=CDS;gene=OBFC1;product=CST complex subunit STN1 isoform X1;protein_id=XP_008268695.1

GO:1990879; CST complex.

Kozak: CCCATGT ID=cds-XP_017204143.1;Parent=rna-XM_017348654.1;Dbxref=GeneID:100350841,Genbank:XP_017204143.1;Name=XP_017204143.1;Note=The sequence of the model RefSeq protein was modified relative to this genomic sequence to represent the inferred CDS: substituted 1 base at 1 genomic stop codon;gbkey=CDS;gene=LOC100350841;product=LOW QUALITY PROTEIN:

Cytochrome P450 2C5 like;protein_id=XP_017204143.1;

GO:0020037; Heme binding.

Kozak: TCCATGT ID=cds-XP_017203933.1;Parent=rna-XM_017348444.1;Dbxref=GeneID:108175409,Genbank:XP_017203933.1;Name=XP_017203933.1;gbkey=CDS;gene=LOC108175409;product=cytochrome P450 2C15-like;protein_id=XP_017203933.1

GO:0020037; Heme binding.

Kozak: TGGATGC ID=cds-XP_008257618.1;Parent=rna-XM_008259396.2;Dbxref=GeneID:100341344,Genbank:XP_008257618.1;Name=XP_008257618.1;gbkey=CDS;gene=GXYLT1;product=glucoside xylosyltransferase 1;protein_id=XP_008257618.1

GO:0035252; UDP-xylosyltransferase activity.

Kozak: TCCATGT ID=cds-XP_017198883.1;Parent=rna-XM_017343394.1;Dbxref=GeneID:100345073,Genbank:XP_017198883.1;Name=XP_017198883.1;gbkey=CDS;gene=NTF3;product=neurotrophin-3 isoform X1;protein_id=XP_017198883.1

GO:0005576; Extracellular region.

Kozak: TTCATGT ID=cds-XP_002713026.1;Parent=rna-XM_002712980.3;Dbxref=GeneID:100345587,Genbank:XP_002713026.1;Name=XP_002713026.1;gbkey=CDS;gene=LOC100345587;product=

Heterogeneous nuclear ribonucleoprotein A1-like.

Kozak: TCCATGT ID=cds-XP_008258419.1;Parent=rna-XM_008260197.1;Dbxref=GeneID:103349135,Genbank:XP_008258419.1;Name=XP_008258419.1;gbkey=CDS;gene=LOC103349135;product=

Mini-chromosome maintenance complex-binding protein-like.

Kozak: TCCATGT ID=cds-XP_002712861.1;Parent=rna-XM_002712815.3;Dbxref=GeneID:100339728,Genbank:XP_002712861.1;Name=XP_002712861.1;gbkey=CDS;gene=CLEC12A;product=C-type lectin domain family 12 member A;protein_id=XP_002712861.1

GO:0016021; Integral component of membrane.

Kozak: TACATGC ID=cds-XP_017205156.1;Parent=rna-XM_017349667.1;Dbxref=GeneID:100346146,Genbank:XP_017205156.1;Name=XP_017205156.1;gbkey=CDS;gene=ARR3;product=

Arrestin-C.

Kozak. TCCATGT ID=cds-XP_002720146.1;Parent=rna-XM_002720100.3;Dbxref=GeneID:100353208,Genbank:XP_002720146.1;Name=XP_002720146.1;gbkey=CDS;gene=ITGB1BP2;product=integrin beta-1-binding protein 2 isoform X2;protein_id=XP_002720146.1

GO:0016021; Integral component of membrane.

Kozak: TCCATGT ID=cds-XP_008270820.1;Parent=rna-XM_008272598.2;Dbxref=GeneID:100356230,Genbank:XP_008270820.1;Name=XP_008270820.1;gbkey=CDS;gene=PIM2;product=serine/threonine-protein kinase pim-2;protein_id=XP_008270820.1

GO:0005737; Cytoplasm.

Kozak: TGTATGC ID=cds-XP_017205434.1;Parent=rna-XM_017349945.1;Dbxref=GeneID:103351977,Genbank:XP_017205434.1;Name=XP_017205434.1;gbkey=CDS;gene=LOC103351977;product=C

Cancer/testis antigen 55.

Kozak: TACATGC ID=cds-XP_002717445.1;Parent=rna-XM_002717399.3;Dbxref=GeneID:100341708,Genbank:XP_002717445.1;Name=XP_002717445.1;gbkey=CDS;gene=RRP15;product=

RRP15-like protein.

Kozak: CCGATGT ID=cds-XP_017203094.1;Parent=rna-XM_017347605.1;Dbxref=GeneID:100353949,Genbank:XP_017203094.1;Name=XP_017203094.1;gbkey=CDS;gene=LIN9;product=

Protein lin-9 homolog.

Kozak: CCAATGT ID=cds-XP_008266404.1;Parent=rna-XM_008268182.2;Dbxref=GeneID:100353951,Genbank:XP_008266404.1;Name=XP_008266404.1;gbkey=CDS;gene=SDCCAG8;product=serologically defined colon cancer antigen 8 isoform X1;protein_id=XP_008266404.1

GO:0005911; Cell-cell junction.

Kozak: CCGATGT ID=cds-XP_017202901.1;Parent=rna-XM_017347412.1;Dbxref=GeneID:100008640,Genbank:XP_017202901.1;Name=XP_017202901.1;Note=The sequence of the model RefSeq protein was modified relative to this genomic sequence to represent the inferred CDS: added 125 bases not found in genome assembly;exception=annotated by transcript or proteomic data;gbkey=CDS;gene=LOC100008640;inference=similar to RNA sequence%2C mRNA (same species):INSD:KT852940.1;partial=true;product=

ATP-binding cassette sub-family G member 2 isoform X1.

Kozak: CCGATGT ID=cds-XP_017202906.1;Parent=rna-XM_017347417.1;Dbxref=GeneID:100346814,Genbank:XP_017202906.1;Name=XP_017202906.1;Note=The sequence of the model RefSeq protein was modified relative to this genomic sequence to represent the inferred CDS: substituted 1 base at 1 genomic stop codon;gbkey=CDS;gene=LOC100346814;product=LOW QUALITY PROTEIN:

ATP-binding cassette sub-family G member 2.

Kozak: CCAATGT ID=cds-XP_008266099.1;Parent=rna-XM_008267877.1;Dbxref=GeneID:100350768,Genbank:XP_008266099.1;Name=XP_008266099.1;Note=The sequence of the model RefSeq protein was modified relative to this genomic sequence to represent the inferred CDS: inserted 5 bases in 3 codons%3B deleted 1 base in 1 codon%3B substituted 1 base at 1 genomic stop codon;exception=unclassified translation discrepancy;gbkey=CDS;gene=LOC100350768;product=LOW QUALITY PROTEIN:

Splicing factor 3A subunit 2.

Kozak: TTCATGT ID=cds-XP_002717223.1;Parent=rna-XM_002717177.3;Dbxref=GeneID:100009367,Genbank:XP_002717223.1;Name=XP_002717223.1;gbkey=CDS;gene=AIMP1;product=aminoacyl tRNA synthase complex-interacting multifunctional protein 1;protein_id=XP_002717223.1

GO:0017101; Aminoacyl-tRNA synthetase multienzyme complex.

Kozak: CCGATGT ID=cds-XP_002714160.1;Parent=rna-XM_002714114.3;Dbxref=GeneID:100356114,Genbank:XP_002714160.1;Name=XP_002714160.1;gbkey=CDS;gene=TARS;product=threonine--tRNA ligase%2C cytoplasmic;protein_id=XP_002714160.1

GO:0005737; Cytoplasm.

Kozak: TGGATGC ID=cds-XP_017197889.1;Parent=rna-XM_017342400.1;Dbxref=GeneID:100348817,Genbank:XP_017197889.1;Name=XP_017197889.1;gbkey=CDS;gene=LOC100348817;product=

WD repeat-containing protein 87.

Kozak: CCGATGT ID=cds-XP_002711898.2;Parent=rna-XM_002711852.3;Dbxref=GeneID:100008960,Genbank:XP_002711898.2;Name=XP_002711898.2;gbkey=CDS;gene=AQP8;product=aquaporin-8;protein_id=XP_002711898.2

GO:0045177; Apical part of cell.

Kozak: TGCATGC ID=cds-XP_002711960.1;Parent=rna-XM_002711914.3;Dbxref=GeneID:100357573,Genbank:XP_002711960.1;Name=XP_002711960.1;gbkey=CDS;gene=MOSPD3;product=motile sperm domain-containing protein 3;protein_id=XP_002711960.1

GO:0005789; Endoplasmic reticulum membrane.

Kozak: TGCATGC ID=cds-XP_017199826.1;Parent=rna-XM_017344337.1;Dbxref=GeneID:100340919,Genbank:XP_017199826.1;Name=XP_017199826.1;Note=The sequence of the model RefSeq protein was modified relative to this genomic sequence to represent the inferred CDS: inserted 1 base in 1 codon%3B deleted 1 base in 1 codon;exception=unclassified translation discrepancy;gbkey=CDS;gene=LOC100340919;product=LOW QUALITY PROTEIN:

Up-regulator of cell proliferation.

Kozak: TGTATGT ID=cds-XP_008259957.1;Parent=rna-XM_008261735.2;Dbxref=GeneID:100343469,Genbank:XP_008259957.1;Name=XP_008259957.1;gbkey=CDS;gene=LOC100343469;product=bax inhibitor 1-like;protein_id=XP_008259957.1

GO:0016021; Integral component of membrane.

Kozak: CCAATGT ID=cds-XP_017199869.1;Parent=rna-XM_017344380.1;Dbxref=GeneID:100351329,Genbank:XP_017199869.1;Name=XP_017199869.1;gbkey=CDS;gene=TRA2A;product=transformer-2 protein homolog alpha isoform X3;protein_id=XP_017199869.1

GO:0005730; Nucleolus.

Kozak: TGGATGC ID=cds-XP_002713892.1;Parent=rna-XM_002713846.3;Dbxref=GeneID:100358003,Genbank:XP_002713892.1;Name=XP_002713892.1;gbkey=CDS;gene=POLM;product=DNA-directed DNA/RNA polymerase mu isoform X1;protein_id=XP_002713892.1

GO:0005634; Nucleus.

Kozak: TTCATGT ID=cds-NP_001075516.1;Parent=rna-NM_001082047.1;Dbxref=GeneID:100008711,Genbank:NP_001075516.1;Name=NP_001075516.1;Note=The RefSeq protein has 4 substitutions and aligns at 24%25 coverage compared to this genomic sequence;end_range=15537461; exception=annotated by transcript or proteomic data;gbkey=CDS;gene=BSP1;inference=similar to AA sequence (same species):RefSeq:NP_001075516.1;partial=true;product=

Binder of sperm 1 precursor

Kozak: TCCATGT ID=cds-XP_017204997.1;Parent=rna-XM_017349508.1;Dbxref=GeneID:100346142,Genbank:XP_017204997.1;Name=XP_017204997.1;gbkey=CDS;gene=RNFT2;product=

RING finger and transmembrane domain-containing protein 2.

Kozak: CCCATGT ID=cds-XP_008270539.1;Parent=rna-XM_008272317.2;Dbxref=GeneID:100354315,Genbank:XP_008270539.1;Name=XP_008270539.1;gbkey=CDS;gene=TMEM233;product=transmembrane protein 233 isoform X1;protein_id=XP_008270539.1

GO:0016021; Integral component of membrane.

Kozak: CCGATGT ID=cds-XP_002720482.2;Parent=rna-XM_002720436.3;Dbxref=GeneID:100339427,Genbank:XP_002720482.2;Name=XP_002720482.2;gbkey=CDS;gene=WDR38;product=WD repeat-containing protein 38;protein_id=XP_002720482.2

GO:0002244; Hematopoietic progenitor cell differentiation.

Kozak: CCCATGT ID=cds-XP_002720829.1;Parent=rna-XM_002720783.3;Dbxref=GeneID:100353212,Genbank:XP_002720829.1;Name=XP_002720829.1;gbkey=CDS;gene=BAG4;product=BAG family molecular chaperone regulator 4;protein_id=XP_002720829.1

GO:0005829; Cytosol.

Kozak: TTCATGT ID=cds-XP_008272474.2;Parent=rna-XM_008274252.2;Dbxref=GeneID:103352259,Genbank:XP_008272474.2;Name=XP_008272474.2;gbkey=CDS;gene=LOC103352259;product=retinol dehydrogenase 16-like;protein_id=XP_008272474.2

GO:0016491; Oxidoreductase activity.

Kozak: TGTATGC ID=cds-XP_017206244.1;Parent=rna-XM_017350755.1;Dbxref=GeneID:100357266,Genbank:XP_017206244.1;Name=XP_017206244.1;Note=The sequence of the model RefSeq protein was modified relative to this genomic sequence to represent the inferred CDS: added 240 bases not found in genome assembly;exception=annotated by transcript or proteomic data;gbkey=CDS;gene=PREX1;inference=similar to RNA sequence (same species):INSD:GBCK01019375.1;partial=true;product=

Phosphatidylinositol trisphosphate-dependent Rac exchanger 1.

Kozak: TGTATGT ID=cds-XP_017194454.1;Parent=rna-XM_017338965.1;Dbxref=GeneID:100351460,Genbank:XP_017194454.1;Name=XP_017194454.1;gbkey=CDS;gene=GLE1;product=nucleoporin GLE1 isoform X2;protein_id=XP_017194454.1

GO:0005814; Centriole.

Kozak: TGCATGC ID=cds-XP_017192997.1;Parent=rna-XM_017337508.1;Dbxref=GeneID:108175476,Genbank:XP_017192997.1;Name=XP_017192997.1;Note=The sequence of the model RefSeq protein was modified relative to this genomic sequence to represent the inferred CDS: inserted 2 bases in 1 codon;exception=unclassified translation discrepancy;gbkey=CDS;gene=LOC108175476;product=LOW QUALITY PROTEIN: Bromodomain-containing protein DDB_G0271118-like.

Kozak: CCAATGT ID=cds-XP_017193012.1;Parent=rna-XM_017337523.1;Dbxref=GeneID:108175478,Genbank:XP_017193012.1;Name=XP_017193012.1;gbkey=CDS;gene=LOC108175478;product=

UDP-glucuronosyltransferase 1-8-like.

Kozak: CCCATGT ID=cds-XP_017193717.1;Parent=rna-XM_017338228.1;Dbxref=GeneID:103345451,Genbank:XP_017193717.1;Name=XP_017193717.1;gbkey=CDS;gene=LOC103345451;product=transforming growth factor beta-1;protein_id=XP_017193717.1

GO:0005615; Extracellular space.

Kozak: CCCATGT ID=cds-XP_017193718.1;Parent=rna-XM_017338229.1;Dbxref=GeneID:108175468,Genbank:XP_017193718.1;Name=XP_017193718.1;gbkey=CDS;gene=LOC108175468;product=

Transforming growth factor beta-1-like.

Kozak: CCCATGT ID=cds-XP_008249173.1;Parent=rna-XM_008250951.1;Dbxref=GeneID:103345789,Genbank:XP_008249173.1;Name=XP_008249173.1;Note=The sequence of the model RefSeq protein was modified relative to this genomic sequence to represent the inferred CDS: added 219 bases not found in genome assembly;exception=annotated by transcript or proteomic data;gbkey=CDS;gene=LOC103345789;inference=similar to RNA sequence (same species):INSD:GBCI01197233.1;partial=true;product=

Tyrosine-protein phosphatase non-receptor type 23.

Kozak: CGAATGT ID=cds-XP_002721117.1;Parent=rna-XM_002721071.3;Dbxref=GeneID:100348692,Genbank:XP_002721117.1;Name=XP_002721117.1;gbkey=CDS;gene=TTPAL;product=

Alpha-tocopherol transfer protein-like isoform X1.

Kozak: TTCATGT ID=cds-XP_017193326.1;Parent=rna-XM_017337837.1;Dbxref=GeneID:108175650,Genbank:XP_017193326.1;Name=XP_017193326.1;Note=The sequence of the model RefSeq protein was modified relative to this genomic sequence to represent the inferred CDS: added 125 bases not found in genome assembly;end_range=1711985,.;exception=annotated by transcript or proteomic data;gbkey=CDS;gene=LOC108175650;inference=similar to RNA sequence (same species):INSD:GBCJ01092956.1;partial=true;product=

Translation initiation factor IF-2-like.

Kozak: TTCATGT ID=cds-XP_008248577.1;Parent=rna-XM_008250355.2;Dbxref=GeneID:103345600,Genbank:XP_008248577.1;Name=XP_008248577.1;gbkey=CDS;gene=FAM83G;product=protein FAM83G isoform X1;protein_id=XP_008248577.1

GO:0005829; Cytosol.

Kozak: TGCATGC ID=cds-XP_002722600.2;Parent=rna-XM_002722554.3;Dbxref=GeneID:100358051,Genbank:XP_002722600.2;Name=XP_002722600.2;gbkey=CDS;gene=GGT5;product=gamma-glutamyltransferase 5 isoform X1;protein_id=XP_002722600.2

GO:0016021; Integral component of membrane.

Kozak: TACATGC ID=cds-XP_008247816.1;Parent=rna-XM_008249594.2;Dbxref=GeneID:100337937,Genbank:XP_008247816.1;Name=XP_008247816.1;Note=The sequence of the model RefSeq protein was modified relative to this genomic sequence to represent the inferred CDS: deleted 2 bases in 2 codons;exception=unclassified translation discrepancy;gbkey=CDS;gene=TFAP4;product=LOW QUALITY PROTEIN:

Transcription factor AP-4.

Kozak: TGTATGC ID=cds-XP_008272521.1;Parent=rna-XM_008274299.2;Dbxref=GeneID:103352280,Genbank:XP_008272521.1;Name=XP_008272521.1;gbkey=CDS;gene=LOC103352280;product=

Translation initiation factor IF-2.

Kozak: CCCATGT ID=cds-XP_008247306.2;Parent=rna-XM_008249084.2;Dbxref=GeneID:100343763,Genbank:XP_008247306.2;Name=XP_008247306.2;gbkey=CDS;gene=EIF4H;product=eukaryotic translation initiation factor 4H;protein_id=XP_008247306.2

GO:0048471; Perinuclear region of cytoplasm.

Kozak: TGGATGC ID=cds-XP_017194263.1;Parent=rna-XM_017338774.1;Dbxref=GeneID:100343359,Genbank:XP_017194263.1;Name=XP_017194263.1;gbkey=CDS;gene=LOC100343359;product=olfactory receptor 18-like;protein_id=XP_017194263.1

GO:0016021; Integral component of membrane.

Kozak: TGCATGC ID=cds-NP_001160740.1;Parent=rna-NM_001167268.1;Dbxref=GeneID:100311048,Genbank:NP_001160740.1;Name=NP_001160740.1;gbkey=CDS;gene=ORYCUNV1R1581;product=vomeronasal 1 receptor oryCunV1R1581;protein_id=NP_001160740.1

GO:0016021; Integral component of membrane.

Kozak: TACATGC ID=cds-NP_001160667.1;Parent=rna-NM_001167195.1;Dbxref=GeneID:100310973,Genbank:NP_001160667.1;Name=NP_001160667.1;Note=The RefSeq protein has 2 substitutions compared to this genomic sequence;exception=annotated by transcript or proteomic data;gbkey=CDS;gene=ORYCUNV1R1506;inference=similar to AA sequence (same species):RefSeq:NP_001160667.1;product=vomeronasal 1 receptor oryCunV1R1506;protein_id=NP_001160667.1

GO:0016021; Integral component of membrane.

Kozak: TGCATGC ID=cds-NP_001160767.1;Parent=rna-NM_001167295.1;Dbxref=GeneID:100311075,Genbank:NP_001160767.1;Name=NP_001160767.1;Note=The RefSeq protein has 2 substitutions compared to this genomic sequence;exception=annotated by transcript or proteomic data;gbkey=CDS;gene=ORYCUNV1R1608;inference=similar to AA sequence (same species):RefSeq:NP_001160767.1;product=vomeronasal 1 receptor oryCunV1R1608;protein_id=NP_001160767.1

GO:0016021; Integral component of membrane.

Kozak: TGCATGC ID=cds-NP_001160805.1;Parent=rna-NM_001167333.1;Dbxref=GeneID:100311113,Genbank:NP_001160805.1;Name=NP_001160805.1;Note=The RefSeq protein has 1 substitution compared to this genomic sequence;exception=annotated by transcript or proteomic data;gbkey=CDS;gene=ORYCUNV1R1652;inference=similar to AA sequence (same species):RefSeq:NP_001160805.1;product=vomeronasal 1 receptor oryCunV1R1652;protein_id=NP_001160805.1

GO:0016021; Integral component of membrane.

Kozak: CCCATGT ID=cds-XP_002721668.1;Parent=rna-XM_002721622.3;Dbxref=GeneID:100347019,Genbank:XP_002721668.1;Name=XP_002721668.1;gbkey=CDS;gene=LOC100347019;product=histone H2A type 1-H;protein_id=XP_002721668.1

GO:0000786; Nucleosome.

Kozak: TTCATGT ID=cds-XP_017193191.1;Parent=rna-XM_017337702.1;Dbxref=GeneID:100340705,Genbank:XP_017193191.1;Name=XP_017193191.1;gbkey=CDS;gene=SLC39A9;product=zinc transporter ZIP9 isoform X2;protein_id=XP_017193191.

GO:0016021; Integral component of membrane.

Kozak: CCGATGT ID=cds-XP_008249863.1;Parent=rna-XM_008251641.1;Dbxref=GeneID:103346108,Genbank:XP_008249863.1;Name=XP_008249863.1;gbkey=CDS;gene=LOC103346108;product=

Dynein heavy chain 10%2C axonemal.

Kozak: CGAATGT ID=cds-XP_017206347.1;Parent=rna-XM_017350858.1;Dbxref=GeneID:100351546,Genbank:XP_017206347.1;Name=XP_017206347.1;gbkey=CDS;gene=LOC100351546;product=

Melanoma-associated antigen 8.

Kozak: TCCATGT ID=cds-XP_017194249.1;Parent=rna-XM_017338760.1;Dbxref=GeneID:100341579,Genbank:XP_017194249.1;Name=XP_017194249.1;gbkey=CDS;gene=MYL10;product=myosin regulatory light chain 10;protein_id=XP_017194249.1

GO:0005739; Mitochondrion.

Kozak: TTAATGC ID=cds-XP_008273625.1;Parent=rna-XM_008275403.2;Dbxref=GeneID:103352517,Genbank:XP_008273625.1;Name=XP_008273625.1;Note=The sequence of the model RefSeq protein was modified relative to this genomic sequence to represent the inferred CDS: added 461 bases not found in genome assembly;exception=annotated by transcript or proteomic data;gbkey=CDS;gene=CRLF2;inference=similar to RNA sequence (same species):INSD:GBCH01012338.1;partial=true;product=

Cytokine receptor-like factor 2.

Kozak: TTCATGT ID=cds-XP_002723401.3;Parent=rna-XM_002723355.3;Dbxref=GeneID:100356681,Genbank:XP_002723401.3;Name=XP_002723401.3;gbkey=CDS;gene=CDPF1;product=

Cysteine-rich DPF motif domain-containing protein 1 isoform X1.

Kozak: TCCATGT ID=cds-XP_017194611.1;Parent=rna-XM_017339122.1;Dbxref=GeneID:100353638,Genbank:XP_017194611.1;Name=XP_017194611.1;gbkey=CDS;gene=ZNF180;product=

Zinc finger protein 180.

Kozak: TCCATGT ID=cds-XP_008249360.1;Parent=rna-XM_008251138.2;Dbxref=GeneID:100357279,Genbank:XP_008249360.1;Name=XP_008249360.1;gbkey=CDS;gene=COL4A3BP;product=collagen type IV alpha-3-binding protein isoform X1;protein_id=XP_008249360.1

GO:0005829; Cytosol.

Kozak: TTCATGT ID=cds-XP_017194529.1;Parent=rna-XM_017339040.1;Dbxref=GeneID:100358832,Genbank:XP_017194529.1;Name=XP_017194529.1;Note=The sequence of the model RefSeq protein was modified relative to this genomic sequence to represent the inferred CDS: inserted 1 base in 1 codon;exception=unclassified translation discrepancy;gbkey=CDS;gene=LOC100358832;product=LOW QUALITY PROTEIN: histone-lysine N-methyltransferase PRDM9; protein_id=XP_017194529.1

GO:0005634; Nucleus.

Kozak: TTCATGT ID=cds-XP_002721862.1;Parent=rna-XM_002721816.3;Dbxref=GeneID:100350123,Genbank:XP_002721862.1;Name=XP_002721862.1;gbkey=CDS;gene=PAPD7;product=non-canonical poly(A) RNA polymerase PAPD7;protein_id=XP_002721862.1

GO:0005794; Golgi apparatus.

Kozak: TCCATGT ID=cds-XP_002723774.2;Parent=rna-XM_002723728.3;Dbxref=GeneID:100344034,Genbank:XP_002723774.2;Name=XP_002723774.2;gbkey=CDS;gene=RTP5;product=receptor-transporting protein 5;protein_id=XP_002723774.2

GO:0016021; Integral component of membrane.

Kozak: TTCATGT ID=cds-XP_017195308.1;Parent=rna-XM_017339819.1;Dbxref=GeneID:108176063,Genbank:XP_017195308.1;Name=XP_017195308.1;Note=The sequence of the model RefSeq protein was modified relative to this genomic sequence to represent the inferred CDS: added 617 bases not found in genome assembly;end_range=57190,.;exception=annotated by transcript or proteomic data;gbkey=CDS;gene=LOC108176063;inference=similar to RNA sequence (same species):INSD:GBCK01141007.1;partial=true;product=

Formin-like protein 5.

Kozak: TGGATGC ID=cds-XP_017194374.1;Parent=rna-XM_017338885.1;Dbxref=GeneID:100345129,Genbank:XP_017194374.1;Name=XP_017194374.1;gbkey=CDS;gene=LOC100345129;product=putative gustatory receptor clone PTE01;protein_id=XP_017194374.1

GO:0016021; Integral component of membrane.

Kozak: TCCATGT ID=cds-XP_017194375.1;Parent=rna-XM_017338886.1;Dbxref=GeneID:100345385,Genbank:XP_017194375.1;Name=XP_017194375.1;gbkey=CDS;gene=LOC100345385;product=putative gustatory receptor clone PTE01;protein_id=XP_017194375.1

GO:0016021; Integral component of membrane.

Kozak: TCCATGT ID=cds-XP_017194383.1;Parent=rna-XM_017338894.1;Dbxref=GeneID:100346931,Genbank:XP_017194383.1;Name=XP_017194383.1;gbkey=CDS;gene=LOC100346931;product=

Olfactory receptor 7E24-like.

Kozak: TCCATGT ID=cds-XP_002722953.2;Parent=rna-XM_002722907.2;Dbxref=GeneID:100347449,Genbank:XP_002722953.2;Name=XP_002722953.2;gbkey=CDS;gene=LOC100347449;product=olfactory receptor 7E24-like;protein_id=XP_002722953.2 GO;

GO:0016021; Integral component of membrane.

Kozak: TCCATGT ID=cds-XP_017194385.1;Parent=rna-XM_017338896.1;Dbxref=GeneID:100346416,Genbank:XP_017194385.1;Name=XP_017194385.1;gbkey=CDS;gene=LOC100346416;product=olfactory receptor 7E24-like;protein_id=XP_017194385.1

GO:0016021; Integral component of membrane.

Kozak: TCCATGT ID=cds-XP_017194380.1;Parent=rna-XM_017338891.1;Dbxref=GeneID:100348701,Genbank:XP_017194380.1;Name=XP_017194380.1;gbkey=CDS;gene=LOC100348701;product=olfactory receptor 7E24-like;protein_id=XP_017194380.1

GO:0016021; Integral component of membrane.

Kozak: CCCATGT ID=cds-XP_017194729.1;Parent=rna-XM_017339240.1;Dbxref=GeneID:100343267,Genbank:XP_017194729.1;Name=XP_017194729.1;gbkey=CDS;gene=LOC100343267;product=

Olfactory receptor 7A10-like.

Kozak: TGGATGC ID=cds-XP_017195333.1;Parent=rna-XM_017339844.1;Dbxref=GeneID:103346397,Genbank:XP_017195333.1;Name=XP_017195333.1;Note=The sequence of the model RefSeq protein was modified relative to this genomic sequence to represent the inferred CDS: inserted 2 bases in 2 codons%3B deleted 2 bases in 2 codons%3B added 853 bases not found in genome assembly;exception=annotated by transcript or proteomic data;gbkey=CDS;gene=HMCN2;inference=similar to RNA sequence (same species):INSD:GBCD01019924.1;partial=true;product=LOW QUALITY PROTEIN: Hemicentin-2.

Kozak: CCCATGT ID=cds-XP_017195598.1;Parent=rna-XM_017340109.1;Dbxref=GeneID:108176143,Genbank:XP_017195598.1;Name=XP_017195598.1;gbkey=CDS;gene=LOC108176143;product=

V-set and immunoglobulin domain-containing protein 10-like.

Kozak: CCGATGT ID=cds-XP_002723426.1;Parent=rna-XM_002723380.3;Dbxref=GeneID:100345904,Genbank:XP_002723426.1;Name=XP_002723426.1;gbkey=CDS;gene=CHMP7;product=

Charged multivesicular body protein 7 isoform X1.

Kozak: TCCATGT ID=cds-NP_001164528.1;Parent=rna-NM_001171057.1;Dbxref=GeneID:100328600,Genbank:NP_001164528.1;Name=NP_001164528.1;Note=isoform 1 is encoded by transcript variant 1%3B The RefSeq protein has 9 substitutions%2C 2 frameshifts and aligns at 87%25 coverage compared to this genomic sequence;end_range=10866,.;exception=annotated by transcript or proteomic data;gbkey=CDS;gene=KCNAB2;inference=similar to AA sequence (same species):RefSeq:NP_001164528.1;partial=true;product=

Voltage-gated potassium channel subunit beta-2 isoform 1

Kozak: TGCATGC ID=cds-XP_002724056.2;Parent=rna-XM_002724010.3;Dbxref=GeneID:100349896,Genbank:XP_002724056.2;Name=XP_002724056.2;gbkey=CDS;gene=VPREB3;product=

Pre-B lymphocyte protein 3.

Kozak: CCGATGT ID=cds-XP_008250685.1;Parent=rna-XM_008252463.2;Dbxref=GeneID:103346401,Genbank:XP_008250685.1;Name=XP_008250685.1;gbkey=CDS;gene=MUC5B;product=mucin-5B;protein_id=XP_008250685.1 NA

103346559:cds-XP_017195509.1 TGCATGC ID=cds-XP_017195509.1;Parent=rna-XM_017340020.1;Dbxref=GeneID:103346559,Genbank:XP_017195509.1;Name=XP_017195509.1;gbkey=CDS;gene=LOC103346559;product=k

Keratin-associated protein 10-7.

Kozak: TGCATGC ID=cds-XP_017195467.1;Parent=rna-XM_017339978.1;Dbxref=GeneID:103346520,Genbank:XP_017195467.1;Name=XP_017195467.1;gbkey=CDS;gene=LOC103346520;product=

Golgin subfamily A member 2.

Kozak: TGCATGC ID=cds-XP_008252131.1;Parent=rna-XM_008253909.2;Dbxref=GeneID:103347399,Genbank:XP_008252131.1;Name=XP_008252131.1;Note=The sequence of the model RefSeq protein was modified relative to this genomic sequence to represent the inferred CDS: deleted 1 base in 1 codon%3B added 827 bases not found in genome assembly;end_range=12880,.;exception=annotated by transcript or proteomic data;gbkey=CDS;gene=ESRRA;inference=similar to RNA sequence (same species):INSD:GBCA01026897.1;partial=true;product=LOW QUALITY PROTEIN:

Steroid hormone receptor ERR1.

Kozak: TCCATGT ID=cds-XP_002723717.2;Parent=rna-XM_002723671.2;Dbxref=GeneID:100345818,Genbank:XP_002723717.2;Name=XP_002723717.2;gbkey=CDS;gene=LOC100345818;product=olfactory receptor 7E24-like;protein_id=XP_002723717.2

GO:0016021; Integral component of membrane.

Kozak: TCCATGT ID=cds-XP_017195273.1;Parent=rna-XM_017339784.1;Dbxref=GeneID:100346593,Genbank:XP_017195273.1;Name=XP_017195273.1;gbkey=CDS;gene=LOC100346593;product=olfactory receptor 18-like;protein_id=XP_017195273.1

GO:0016021; Integral component of membrane.

Kozak: TTCATGT ID=cds-XP_017195274.1;Parent=rna-XM_017339785.1;Dbxref=GeneID:100348370,Genbank:XP_017195274.1;Name=XP_017195274.1;gbkey=CDS;gene=LOC100348370;product=olfactory receptor 18-like;protein_id=XP_017195274.1

GO:0016021; Integral component of membrane.

Kozak: CCCATGT ID=cds-XP_002723724.1;Parent=rna-XM_002723678.1;Dbxref=GeneID:100348875,Genbank:XP_002723724.1;Name=XP_002723724.1;gbkey=CDS;gene=LOC100348875;product=putative gustatory receptor clone PTE01;protein_id=XP_002723724.1

GO:0016021; Integral component of membrane.

Kozak: TGCATGC ID=cds-XP_008250407.2;Parent=rna-XM_008252185.2;Dbxref=GeneID:103346293,Genbank:XP_008250407.2;Name=XP_008250407.2;gbkey=CDS;gene=LOC103346293;product=

Zinc finger protein 260-like.

Kozak: CCAATGT ID=cds-XP_008250862.2;Parent=rna-XM_008252640.2;Dbxref=GeneID:103346500,Genbank:XP_008250862.2;Name=XP_008250862.2;Note=The sequence of the model RefSeq protein was modified relative to this genomic sequence to represent the inferred CDS: added 124 bases not found in genome assembly;end_range=178740,.;exception=annotated by transcript or proteomic data;gbkey=CDS;gene=LOC103346500;inference=similar to RNA sequence (same species):INSD:GBCM01059343.1;partial=true;product=

Afadin.

Kozak: TGTATGC ID=cds-XP_008251532.1;Parent=rna-XM_008253310.1;Dbxref=GeneID:100338299,Genbank:XP_008251532.1;Name=XP_008251532.1;Note=The sequence of the model RefSeq protein was modified relative to this genomic sequence to represent the inferred CDS: inserted 4 bases in 3 codons%3B deleted 4 bases in 3 codons%3B substituted 2 bases at 2 genomic stop codons;exception=unclassified translation discrepancy;gbkey=CDS;gene=LOC100338299;product=LOW QUALITY PROTEIN: Dystrophia myotonica WD repeat-containing protein.

Kozak: CCGATGT ID=cds-XP_008251915.1;Parent=rna-XM_008253693.2;Dbxref=GeneID:100357978,Genbank:XP_008251915.1;Name=XP_008251915.1;Note=The sequence of the model RefSeq protein was modified relative to this genomic sequence to represent the inferred CDS: deleted 1 base in 1 codon%3B substituted 1 base at 1 genomic stop codon;exception=unclassified translation discrepancy;gbkey=CDS;gene=LOC100357978;product=LOW QUALITY PROTEIN:

EH domain-containing protein.

Kozak: TGCATGC ID=cds-XP_017195859.1;Parent=rna-XM_017340370.1;Dbxref=GeneID:108175449,Genbank:XP_017195859.1;Name=XP_017195859.1;Note=The sequence of the model RefSeq protein was modified relative to this genomic sequence to represent the inferred CDS: added 134 bases not found in genome assembly;end_range=46693,.;exception=annotated by transcript or proteomic data;gbkey=CDS;gene=LOC108175449;inference=similar to RNA sequence (same species):INSD:GBAX01006219.1;partial=true;product=Zinc finger protein 39.

GO:0046872; Metal ion binding.

Kozak: TGGATGC ID=cds-XP_008251868.1;Parent=rna-XM_008253646.1;Dbxref=GeneID:103347180,Genbank:XP_008251868.1;Name=XP_008251868.1;Note=The sequence of the model RefSeq protein was modified relative to this genomic sequence to represent the inferred CDS: added 367 bases not found in genome assembly;exception=annotated by transcript or proteomic data;gbkey=CDS;gene=MGAT5B;inference=similar to RNA sequence (same species):INSD:GBCT01117367.1;partial=true;product=alpha-1%2C6-mannosylglycoprotein 6-beta-N-acetylglucosaminyltransferase B;protein_id=XP_008251868.1;start_range=.,5730

GO:0000139; Golgi membrane.

Kozak: TGCATGC ID=cds-XP_017195562.1;Parent=rna-XM_017340073.1;Dbxref=GeneID:108176135,Genbank:XP_017195562.1;Name=XP_017195562.1;gbkey=CDS;gene=LOC108176135;product=

Zinc finger protein 260-like.

Kozak: TGCATGC ID=cds-XP_017195723.1;Parent=rna-XM_017340234.1;Dbxref=GeneID:103346829,Genbank:XP_017195723.1;Name=XP_017195723.1;gbkey=CDS;gene=LOC103346829;product=

Integrin alpha-9-like.

Kozak: CCGATGT ID=cds-XP_002724136.1;Parent=rna-XM_002724090.3;Dbxref=GeneID:100349813,Genbank:XP_002724136.1;Name=XP_002724136.1;gbkey=CDS;gene=SOX1;product=transcription factor SOX-1;protein_id=XP_002724136.1

GO:0005634; Nucleus

Kozak: CCCATGT ID=cds-XP_017196108.1;Parent=rna-XM_017340619.1;Dbxref=GeneID:108176323,Genbank:XP_017196108.1;Name=XP_017196108.1;end_range=8655,.;gbkey=CDS;gene=LOC108176323;partial=true;product=

Translation initiation factor IF-2-like.
