# Supplementary material for: Functional analysis of the AUG initiator codon context reveals novel conserved sequences that disfavor mRNA translation in eukaryotes
Source: Nucleic Acids Res. 2023 Dec 1;52(3):1064–79. doi: 10.1093/nar/gkad1152 (PMC10853783; doi:10.1093/nar/gkad1152)
Supplement: gkad1152_supplemental_files [file gkad1152_supplemental_files.zip › Suppl. Table 7.docx]

**Supplemental Table 7**

Annotated Gene Ontology (GO) of BACS-containing genes in ***Drosophila* *melanogaster.***

***n* = 82**

**GO annotation**

Kozak: TTAATGC ID=cds-NP_788618.1;Parent=rna-NM_176441.2;Dbxref=FLYBASE:FBpp0081563,GeneID:318260,Genbank:NP_788618.1,FLYBASE:FBgn0052939;Name=NP_788618.1;gbkey=CDS;gene=CG32939;locus_tag=Dmel_CG32939;orig_transcript_id=gnl|FlyBase|CG32939-RA;product=uncharacterized protein;protein_id=NP_788618.1

GO:0016021: Integral component of membrane.

Kozak: TGGATGC ID=cds-NP_730934.1;Parent=rna-NM_169054.2;Dbxref=FLYBASE:FBpp0078330,GeneID:40655,Genbank:NP_730934.1,FLYBASE:FBgn0037329;Name=NP_730934.1;gbkey=CDS;gene=POLDIP2;locus_tag=Dmel_CG12162;orig_transcript_id=gnl|FlyBase|CG12162-RB;product=polymerase (DNA-directed)%2C delta interacting protein 2%2C isoform B;protein_id=NP_730934.1

GO:0042645; Mitochondrial nucleoid.

Kozak: TTAATGC ID=cds-NP_649928.1;Parent=rna-NM_141671.4;Dbxref=FLYBASE:FBpp0081562,GeneID:41176,Genbank:NP_649928.1,FLYBASE:FBgn0037731;Name=NP_649928.1;gbkey=CDS;gene=CG18542;locus_tag=Dmel_CG18542;orig_transcript_id=gnl|FlyBase|CG18542-RA;product=uncharacterized protein;protein_id=NP_649928.1

GO; GO:0016021: Integral component of membrane.

Kozak: CCAATGT ID=cds-NP_001138040.1;Parent=rna-NM_001144568.2;Dbxref=FLYBASE:FBpp0113057,GeneID:41512,Genbank:NP_001138040.1,FLYBASE:FBgn0038029;Name=NP_001138040.1;gbkey=CDS;gene=GstD11;locus_tag=Dmel_CG17639;orig_transcript_id=gnl|FlyBase|CG17639-RB;product=glutathione S transferase D11%2C isoform B;protein_id=NP_001138040.1

GO:0005737: Cytoplasm.

Kozak: CGAATGT ID=cds-NP_650946.3;Parent=rna-NM_142689.4;Dbxref=FLYBASE:FBpp0083418,GeneID:42507,Genbank:NP_650946.3,FLYBASE:FBgn0038860;Name=NP_650946.3;gbkey=CDS;gene=Ice2;locus_tag=Dmel_CG10825;orig_transcript_id=gnl|FlyBase|CG10825-RA;product=interacts with the C terminus of ELL 2%2C isoform A;protein_id=NP_650946.3

GO:0032783: Super elongation complex.

Kozak: CCCATGT ID=cds-NP_651229.2;Parent=rna-NM_142972.5;Dbxref=FLYBASE:FBpp0083940,GeneID:42877,Genbank:NP_651229.2,FLYBASE:FBgn0039165;Name=NP_651229.2;gbkey=CDS;gene=CG6204;locus_tag=Dmel_CG6204;orig_transcript_id=gnl|FlyBase|CG6204-RA;product=uncharacterized protein;protein_id=NP_651229.2

GO:0031380: Nuclear RNA-directed RNA polymerase complex.

Kozak: CGAATGT ID=cds-NP_651316.1;Parent=rna-NM_143059.1;Dbxref=FLYBASE:FBpp0084126,GeneID:42988,Genbank:NP_651316.1,FLYBASE:FBgn0285912;Name=NP_651316.1;gbkey=CDS;gene=mah;locus_tag=Dmel_CG13646;orig_transcript_id=gnl|FlyBase|CG13646-RA;product=mahogany;protein_id=NP_651316.1

GO:0016021: Integral component of membrane.

Kozak: TCCATGT ID=cds-NP_001138013.2;Parent=rna-NM_001144541.2;Dbxref=FLYBASE:FBpp0306608,GeneID:44039,Genbank:NP_001138013.2,FLYBASE:FBgn0267698;Name=NP_001138013.2;gbkey=CDS;gene=Pak;locus_tag=Dmel_CG10295;orig_transcript_id=gnl|FlyBase|CG10295-RG;product=p21-activated kinase%2C isoform G;protein_id=NP_001138013.2

GO:0031252: Cell leading edge.

Kozak: CGAATGT ID=cds-NP_731685.1;Parent=rna-NM_169460.3;Dbxref=FLYBASE:FBpp0082084,GeneID:41495,Genbank:NP_731685.1,FLYBASE:FBgn0038013;Name=NP_731685.1;gbkey=CDS;gene=CG10038;locus_tag=Dmel_CG10038;orig_transcript_id=gnl|FlyBase|CG10038-RB;product=uncharacterized protein%2C isoform B;protein_id=NP_731685.1

GO:0005634: Nucleus.

Kozak: CCCATGT ID=cds-NP_001097785.1;Parent=rna-NM_001104315.2;Dbxref=FLYBASE:FBpp0111809,GeneID:41718,Genbank:NP_001097785.1,FLYBASE:FBgn0020510;Name=NP_001097785.1;gbkey=CDS;gene=Abi;locus_tag=Dmel_CG9749;orig_transcript_id=gnl|FlyBase|CG9749-RB;product=abelson interacting protein%2C isoform B;protein_id=NP_001097785.1

GO:0098858: Actin-based cell projection.

Kozak: TGCATGC ID=cds-NP_001247155.1;Parent=rna-NM_001260226.2;Dbxref=FLYBASE:FBpp0293546,GeneID:42137,Genbank:NP_001247155.1,FLYBASE:FBgn0262562;Name=NP_001247155.1;gbkey=CDS;gene=CG43102;locus_tag=Dmel_CG43102;orig_transcript_id=gnl|FlyBase|CG43102-RA;product=uncharacterized protein%2C isoform A;protein_id=NP_001247.

GO:0005085: Guanyl-nucleotide exchange factor activity.

Kozak: CCCATGT ID=cds-NP_650994.1;Parent=rna-NM_142737.2;Dbxref=FLYBASE:FBpp0083550,GeneID:42579,Genbank:NP_650994.1,FLYBASE:FBgn0038915;Name=NP_650994.1;gbkey=CDS;gene=CG17819;locus_tag=Dmel_CG17819;orig_transcript_id=gnl|FlyBase|CG17819-RA;product=uncharacterized protein;protein_id=NP_650994.1

GO:0005794: Golgi apparatus.

Kozak: TGTATGC ID=cds-NP_732925.1;Parent=rna-NM_170103.2;Dbxref=FLYBASE:FBpp0083899,GeneID:42834,Genbank:NP_732925.1,FLYBASE:FBgn0005674;Name=NP_732925.1;gbkey=CDS;gene=GluProRS;locus_tag=Dmel_CG5394;orig_transcript_id=gnl|FlyBase|CG5394-RB;product=Glutamyl-prolyl-tRNA synthetase%2C isoform B;protein_id=NP_732925.1

GO:0017101: Aminoacyl-tRNA synthetase multienzyme complex.

Kozak: CCTATGC ID=cds-NP_001262969.1;Parent=rna-NM_001276040.1;Dbxref=FLYBASE:FBpp0303777,GeneID:43104,Genbank:NP_001262969.1,FLYBASE:FBgn0039360;Name=NP_001262969.1;gbkey=CDS;gene=CLS;locus_tag=Dmel_CG4774;orig_transcript_id=gnl|FlyBase|CG4774-RD;product=cardiolipin synthase%2C isoform D;protein_id=NP_001262969.1

GO:0016021: Integral component of membrane.

Kozak: TCCATGT ID=cds-NP_001247327.1;Parent=rna-NM_001260398.1;Dbxref=FLYBASE:FBpp0297113,GeneID:43291,Genbank:NP_001247327.1,FLYBASE:FBgn0039507;Name=NP_001247327.1;gbkey=CDS;gene=mrt;locus_tag=Dmel_CG3361;orig_transcript_id=gnl|FlyBase|CG3361-RB;product=martik%2C isoform B;protein_id=NP_001247327.1

GO:0005634: nucleus.

Kozak: CCAATGT ID=cds-NP_651726.1;Parent=rna-NM_143469.2;Dbxref=FLYBASE:FBpp0084903,GeneID:43513,Genbank:NP_651726.1,FLYBASE:FBgn0039696;Name=NP_651726.1;gbkey=CDS;gene=Rnb;locus_tag=Dmel_CG7837;orig_transcript_id=gnl|FlyBase|CG7837-RA;product=R and B;protein_id=NP_651726.1

GO:0005829: Cytosol.

Kozak: TACATGC ID=cds-NP_725504.1;Parent=rna-NM_166124.4;Dbxref=FLYBASE:FBpp0086341,GeneID:246451,Genbank:NP_725504.1,FLYBASE:FBgn0050093;Name=NP_725504.1;gbkey=CDS;gene=COX6AL;locus_tag=Dmel_CG30093;orig_transcript_id=gnl|FlyBase|CG30093-RA;product=cytochrome c oxidase subunit 6A-like;protein_id=NP_725504.1

GO:0005751: Mitochondrial respiratory chain complex IV.

Kozak: TTAATGC ID=cds-NP_001015349.2;Parent=rna-NM_001015349.4;Dbxref=FLYBASE:FBpp0112507,GeneID:3355064,Genbank:NP_001015349.2,FLYBASE:FBgn0046706;Name=NP_001015349.2;gbkey=CDS;gene=Haspin;locus_tag=Dmel_CG40080;orig_transcript_id=gnl|FlyBase|CG40080-RA;product=haspin;protein_id=NP_001015349.2

GO:0000785: Chromatin.

Kozak: TTCATGT ID=cds-NP_001036470.1;Parent=rna-NM_001043005.2;Dbxref=FLYBASE:FBpp0110537,GeneID:35475,Genbank:NP_001036470.1,FLYBASE:FBgn0033005;Name=NP_001036470.1;gbkey=CDS;gene=CG3107;locus_tag=Dmel_CG3107;orig_transcript_id=gnl|FlyBase|CG3107-RC;product=uncharacterized protein%2C isoform C;protein_id=NP_001036470.1

GO:0005759: Mitochondrial matrix.

Kozak: TTAATGC ID=cds-NP_523620.1;Parent=rna-NM_078896.3;Dbxref=FLYBASE:FBpp0085420,GeneID:35486,Genbank:NP_523620.1,FLYBASE:FBgn0261403;Name=NP_523620.1;gbkey=CDS;gene=sxc;locus_tag=Dmel_CG10392;orig_transcript_id=gnl|FlyBase|CG10392-RB;product=super sex combs%2C isoform B;protein_id=NP_523620.1

GO:0016262: Protein N-acetylglucosaminyltransferase activity.

Kozak: CCAATGT ID=cds-NP_001246144.1;Parent=rna-NM_001259215.2;Dbxref=FLYBASE:FBpp0293272,GeneID:35541,Genbank:NP_001246144.1,FLYBASE:FBgn0033063;Name=NP_001246144.1;gbkey=CDS;gene=CG14589;locus_tag=Dmel_CG14589;orig_transcript_id=gnl|FlyBase|CG14589-RB;product=uncharacterized protein%2C isoform B;protein_id=NP_001246144.1

GO:0016021: Component of membrane.

Kozak: CCCATGT ID=cds-NP_001246156.2;Parent=rna-NM_001259227.2;Dbxref=FLYBASE:FBpp0307719,GeneID:35604,Genbank:NP_001246156.2,FLYBASE:FBgn0033117;Name=NP_001246156.2;gbkey=CDS;gene=CG3358;locus_tag=Dmel_CG3358;orig_transcript_id=gnl|FlyBase|CG3358-RF;product=uncharacterized protein%2C isoform F;protein_id=NP_001246156.2

GO:0008296; F:3'-5'-exodeoxyribonuclease activity.

Kozak: TGGATGC ID=cds-NP_001246184.1;Parent=rna-NM_001259255.1;Dbxref=FLYBASE:FBpp0297794,GeneID:35698,Genbank:NP_001246184.1,FLYBASE:FBgn0263077;Name=NP_001246184.1;gbkey=CDS;gene=CG43340;locus_tag=Dmel_CG43340;orig_transcript_id=gnl|FlyBase|CG43340-RM;product=uncharacterized protein%2C isoform M;protein_id=NP_001246184.1

GO:0005829: Cytosol.

Kozak: TACATGC ID=cds-NP_001260787.1;Parent=rna-NM_001273858.1;Dbxref=FLYBASE:FBpp0307930,GeneID:35707,Genbank:NP_001260787.1,FLYBASE:FBgn0033204;Name=NP_001260787.1;gbkey=CDS;gene=CG2065;locus_tag=Dmel_CG2065;orig_transcript_id=gnl|FlyBase|CG2065-RB;product=uncharacterized protein%2C isoform B;protein_id=NP_001260787.1

GO:0005789: Endoplasmic reticulum membrane.

Kozak: TCCATGT ID=cds-NP_001286305.1;Parent=rna-NM_001299376.1;Dbxref=FLYBASE:FBpp0308569,GeneID:36167,Genbank:NP_001286305.1,FLYBASE:FBgn0033578;Name=NP_001286305.1;gbkey=CDS;gene=BBS4;locus_tag=Dmel_CG13232;orig_transcript_id=gnl|FlyBase|CG13232-RB;product=Bardet-Biedl syndrome 4%2C isoform B;protein_id=NP_001286305.1

GO:0034464: BBSome.

Kozak: TTAATGC ID=cds-NP_610670.2;Parent=rna-NM_136826.3;Dbxref=FLYBASE:FBpp0087219,GeneID:36208,Genbank:NP_610670.2,FLYBASE:FBgn0033615;Name=NP_610670.2;gbkey=CDS;gene=CG7741;locus_tag=Dmel_CG7741;orig_transcript_id=gnl|FlyBase|CG7741-RA;product=uncharacterized protein%2C isoform A;protein_id=NP_610670.2

GO:0071014: Post-mRNA release spliceosomal complex.

Kozak: CCCATGT ID=cds-NP_611008.2;Parent=rna-NM_137164.4;Dbxref=FLYBASE:FBpp0086539,GeneID:36671,Genbank:NP_611008.2,FLYBASE:FBgn0033985;Name=NP_611008.2;gbkey=CDS;gene=CG10257;locus_tag=Dmel_CG10257;orig_transcript_id=gnl|FlyBase|CG10257-RA;product=uncharacterized protein;protein_id=NP_611008.2

GO:0007249: I-kappaB kinase/NF-kappaB signaling.

Kozak: CCCATGT ID=cds-NP_001097358.1;Parent=rna-NM_001103888.3;Dbxref=FLYBASE:FBpp0111302,GeneID:5740534,Genbank:NP_001097358.1,FLYBASE:FBgn0085223;Name=NP_001097358.1;gbkey=CDS;gene=CG34194;locus_tag=Dmel_CG34194;orig_transcript_id=gnl|FlyBase|CG34194-RA;product=uncharacterized protein%2C isoform A;protein_id=NP_001097358.1

GO:0016021: Integral component of membrane.

Kozak: CGAATGT ID=cds-NP_001163062.1;Parent=rna-NM_001169591.2;Dbxref=FLYBASE:FBpp0291400,GeneID:35569,Genbank:NP_001163062.1,FLYBASE:FBgn0033087;Name=NP_001163062.1;gbkey=CDS;gene=Hsepi;locus_tag=Dmel_CG3194;orig_transcript_id=gnl|FlyBase|CG3194-RB;product=heparan sulfate C5-epimerase%2C isoform B;protein_id=NP_001163062.1

GO:0005794: Golgi apparatus.

Kozak: TGGATGC ID=cds-NP_610429.2;Parent=rna-NM_136585.3;Dbxref=FLYBASE:FBpp0087756,GeneID:35895,Genbank:NP_610429.2,FLYBASE:FBgn0033354;Name=NP_610429.2;gbkey=CDS;gene=FANCI;locus_tag=Dmel_CG13745;orig_transcript_id=gnl|FlyBase|CG13745-RA;product=fanconi anemia complementation group I;protein_id=NP_610429.2

GO:0070182: DNA polymerase binding.

Kozak: CGAATGT ID=cds-NP_724916.1;Parent=rna-NM_165763.3;Dbxref=FLYBASE:FBpp0087449,GeneID:36086,Genbank:NP_724916.1,FLYBASE:FBgn0028426;Name=NP_724916.1;gbkey=CDS;gene=RNaseZ;locus_tag=Dmel_CG3298;orig_transcript_id=gnl|FlyBase|CG3298-RB;product=ribonuclease Z;protein_id=NP_724916.1

GO:0005829: Cytosol.

Kozak: CCAATGT ID=cds-NP_001188916.1;Parent=rna-NM_001201987.2;Dbxref=FLYBASE:FBpp0291643,GeneID:36405,Genbank:NP_001188916.1,FLYBASE:FBgn0026619;Name=NP_001188916.1;gbkey=CDS;gene=Taz;locus_tag=Dmel_CG8766;orig_transcript_id=gnl|FlyBase|CG8766-RD;product=tafazzin%2C isoform D;protein_id=NP_001188916.1

GO:0005783: Endoplasmic reticulum.

Kozak: CCAATGT ID=cds-NP_001286473.1;Parent=rna-NM_001299544.1;Dbxref=FLYBASE:FBpp0308803,GeneID:36769,Genbank:NP_001286473.1,FLYBASE:FBgn0034068;Name=NP_001286473.1;gbkey=CDS;gene=casp;locus_tag=Dmel_CG8400;orig_transcript_id=gnl|FlyBase|CG8400-RC;product=caspar%2C isoform C;protein_id=NP_001286473.1

GO:0005737: Cytoplasm.

Kozak: TGTATGC ID=cds-NP_477116.1;Parent=rna-NM_057768.3;Dbxref=FLYBASE:FBpp0292050,GeneID:36806,Genbank:NP_477116.1,FLYBASE:FBgn0261612;Name=NP_477116.1;gbkey=CDS;gene=CngA;locus_tag=Dmel_CG42701;orig_transcript_id=gnl|FlyBase|CG42701-RA;product=cyclic nucleotide-gated ion channel subunit A%2C isoform A;protein_id=NP_477116.1

GO:0017071: Intracellular cyclic nucleotide activated cation channel complex.

Kozak: CCCATGT ID=cds-NP_001356967.1;Parent=rna-NM_001369962.1;Dbxref=FLYBASE:FBpp0423125,GeneID:37443,Genbank:NP_001356967.1,FLYBASE:FBgn0034626;Name=NP_001356967.1;gbkey=CDS;gene=CG10795;locus_tag=Dmel_CG10795;orig_transcript_id=gnl|FlyBase|CG10795-RB;product=uncharacterized protein%2C isoform B;protein_id=NP_001356967.1;transl_except=(pos:complement(21383561..21383563)%2Caa:Other)

GO:0016021: Integral component of membrane.

Kozak: CGAATGT ID=cds-NP_001137681.1;Parent=rna-NM_001144209.2;Dbxref=FLYBASE:FBpp0289320,GeneID:7354430,Genbank:NP_001137681.1,FLYBASE:FBgn0259738;Name=NP_001137681.1;gbkey=CDS;gene=CG42392;locus_tag=Dmel_CG42392;orig_transcript_id=gnl|FlyBase|CG42392-RA;product=uncharacterized protein;protein_id=NP_001137681.1

GO:0016021: Integral component of membrane.

Kozak: CCAATGT ID=cds-NP_612023.1;Parent=rna-NM_138179.3;Dbxref=FLYBASE:FBpp0072407,GeneID:38049,Genbank:NP_612023.1,FLYBASE:FBgn0035124;Name=NP_612023.1;gbkey=CDS;gene=ttm2;locus_tag=Dmel_CG12313;orig_transcript_id=gnl|FlyBase|CG12313-RA;product=tiny tim 2;protein_id=NP_612023.1

GO:0016021: Integral component of membrane.

Kozak: TTAATGC ID=cds-NP_729124.1;Parent=rna-NM_168153.2;Dbxref=FLYBASE:FBpp0076745,GeneID:38677,Genbank:NP_729124.1,FLYBASE:FBgn0052406;Name=NP_729124.1;gbkey=CDS;gene=PVRAP;locus_tag=Dmel_CG32406;orig_transcript_id=gnl|FlyBase|CG32406-RA;product=PVR adaptor protein;protein_id=NP_729124.1

GO:0005172: Vascular endothelial growth factor receptor binding.

Kozak: CCAATGT ID=cds-NP_648016.1;Parent=rna-NM_139759.2;Dbxref=FLYBASE:FBpp0076752,GeneID:38687,Genbank:NP_648016.1,FLYBASE:FBgn0035669;Name=NP_648016.1;gbkey=CDS;gene=CG6592;locus_tag=Dmel_CG6592;orig_transcript_id=gnl|FlyBase|CG6592-RA;product=uncharacterized protein;protein_id=NP_648016.1

GO:0004252: Serine-type endopeptidase activity.

Kozak: CCAATGT ID=cds-NP_001287089.1;Parent=rna-NM_001300160.1;Dbxref=FLYBASE:FBpp0310636,GeneID:39884,Genbank:NP_001287089.1,FLYBASE:FBgn0003410;Name=NP_001287089.1;gbkey=CDS;gene=sina;locus_tag=Dmel_CG9949;orig_transcript_id=gnl|FlyBase|CG9949-RC;product=seven in absentia%2C isoform C;protein_id=NP_001287089.1

GO:0005737: Cytoplasm.

Kozak: TGCATGC ID=cds-NP_001262107.1;Parent=rna-NM_001275178.1;Dbxref=FLYBASE:FBpp0303190,GeneID:40249,Genbank:NP_001262107.1,FLYBASE:FBgn0036980;Name=NP_001262107.1;gbkey=CDS;gene=RhoBTB;locus_tag=Dmel_CG5701;orig_transcript_id=gnl|FlyBase|CG5701-RB;product=Rho-related BTB domain containing%2C isoform B;protein_id=NP_001262107.1;transl_except=(pos:20381696..20381698%2Caa:Other)

GO:0005938: Cell cortex.

Kozak: CCCATGT ID=cds-NP_001287133.1;Parent=rna-NM_001300204.1;Dbxref=FLYBASE:FBpp0311806,GeneID:40307,Genbank:NP_001287133.1,FLYBASE:FBgn0037030;Name=NP_001287133.1;gbkey=CDS;gene=CG3288;locus_tag=Dmel_CG3288;orig_transcript_id=gnl|FlyBase|CG3288-RB;product=uncharacterized protein%2C isoform B;protein_id=NP_001287133.1

GO:0016021: Integral component of membrane.

Kozak: CGAATGT ID=cds-NP_001097666.1;Parent=rna-NM_001104196.3;Dbxref=FLYBASE:FBpp0112349,GeneID:40495,Genbank:NP_001097666.1,FLYBASE:FBgn0052451;Name=NP_001097666.1;gbkey=CDS;gene=SPoCk;locus_tag=Dmel_CG32451;orig_transcript_id=gnl|FlyBase|CG32451-RD;product=secretory pathway calcium atpase%2C isoform D;protein_id=NP_001097666.1

GO:0012505: Endomembrane system.

Kozak: TTCATGT ID=cds-NP_001097552.1;Parent=rna-NM_001104082.1;Dbxref=FLYBASE:FBpp0111639,GeneID:5740622,Genbank:NP_001097552.1,FLYBASE:FBgn0085455;Name=NP_001097552.1;gbkey=CDS;gene=CG34426;locus_tag=Dmel_CG34426;orig_transcript_id=gnl|FlyBase|CG34426-RA;product=uncharacterized protein;protein_id=NP_001097552.1

GO:0005576: Extracellular region.

Kozak: TACATGC ID=cds-NP_001189140.1;Parent=rna-NM_001202211.1;Dbxref=FLYBASE:FBpp0292454,GeneID:10178963,Genbank:NP_001189140.1,FLYBASE:FBgn0261832;Name=NP_001189140.1;gbkey=CDS;gene=CG42764;locus_tag=Dmel_CG42764;orig_transcript_id=gnl|FlyBase|CG42764-RA;product=uncharacterized protein%2C isoform A;protein_id=NP_001189140.1

GO:0005615: Extracellular space.

Kozak: TTAATGC ID=cds-NP_728501.1;Parent=rna-NM_167813.2;Dbxref=FLYBASE:FBpp0072424,GeneID:38037,Genbank:NP_728501.1,FLYBASE:FBgn0035113;Name=NP_728501.1;gbkey=CDS;gene=pyx;locus_tag=Dmel_CG17142;orig_transcript_id=gnl|FlyBase|CG17142-RB;product=pyrexia%2C isoform B;protein_id=NP_728501.1

GO:0034703: Cation channel complex.

Kozak: TGTATGT ID=cds-NP_523928.1;Parent=rna-NM_079204.2;Dbxref=FLYBASE:FBpp0073179,GeneID:38559,Genbank:NP_523928.1,FLYBASE:FBgn0014073;Name=NP_523928.1;gbkey=CDS;gene=Tie;locus_tag=Dmel_CG7525;orig_transcript_id=gnl|FlyBase|CG7525-RA;product=Tie-like receptor tyrosine kinase;protein_id=NP_523928.1

GO:0005887: Integral component of plasma membrane.

Kozak: CCAATGT ID=cds-NP_001261756.1;Parent=rna-NM_001274827.1;Dbxref=FLYBASE:FBpp0305972,GeneID:39410,Genbank:NP_001261756.1,FLYBASE:FBgn0036279;Name=NP_001261756.1;gbkey=CDS;gene=Ncc69;locus_tag=Dmel_CG4357;orig_transcript_id=gnl|FlyBase|CG4357-RC;product=sodium chloride cotransporter 69%2C isoform C;protein_id=NP_001261756.1

GO:0016021: Integral component of membrane.

Kozak: CCAATGT ID=cds-NP_001262026.1;Parent=rna-NM_001275097.2;Dbxref=FLYBASE:FBpp0305860,GeneID:40068,Genbank:NP_001262026.1,FLYBASE:FBgn0036832;Name=NP_001262026.1;gbkey=CDS;gene=CG18223;locus_tag=Dmel_CG18223;orig_transcript_id=gnl|FlyBase|CG18223-RC;product=uncharacterized protein%2C isoform C;protein_id=NP_001262026.1

GO:0006508: Proteolysis.

Kozak: TTAATGC ID=cds-NP_001036587.1;Parent=rna-NM_001043122.2;Dbxref=FLYBASE:FBpp0110067,GeneID:4379909,Genbank:NP_001036587.1,FLYBASE:FBgn0047334;Name=NP_001036587.1;gbkey=CDS;gene=BG642312;locus_tag=Dmel_CG33943;orig_transcript_id=gnl|FlyBase|CG33943-RA;product=BG642312%2C isoform A;protein_id=NP_001036587.1

GO:0005615: Extracellular space.

Kozak: TGGATGC ID=cds-NP_001259132.1;Parent=rna-NM_001272203.1;Dbxref=FLYBASE:FBpp0304570,GeneID:31090,Genbank:NP_001259132.1,FLYBASE:FBgn0024985;Name=NP_001259132.1;gbkey=CDS;gene=Rilpl;locus_tag=Dmel_CG11448;orig_transcript_id=gnl|FlyBase|CG11448-RB;product=Rab interacting lysosomal protein like%2C isoform B;protein_id=NP_001259132.1

GO:0036064: Ciliary basal body.

Kozak: CCCATGT ID=cds-NP_727768.1;Parent=rna-NM_167398.2;Dbxref=FLYBASE:FBpp0073721,GeneID:318108,Genbank:NP_727768.1,FLYBASE:FBgn0260482;Name=NP_727768.1;gbkey=CDS;gene=CG32599;locus_tag=Dmel_CG32599;orig_transcript_id=gnl|FlyBase|CG32599-RA;product=uncharacterized protein;protein_id=NP_727768.1

GO:0016021: Integral component of membrane.

Kozak: CCAATGT ID=cds-NP_727392.1;Parent=rna-NM_167217.1;Dbxref=FLYBASE:FBpp0071355,GeneID:318158,Genbank:NP_727392.1,FLYBASE:FBgn0052693;Name=NP_727392.1;gbkey=CDS;gene=Gr9a;locus_tag=Dmel_CG32693;orig_transcript_id=gnl|FlyBase|CG32693-RA;product=gustatory receptor 9a;protein_id=NP_727392.1

GO:0030424: Axon.

Kozak: TCCATGT ID=cds-NP_001285237.1;Parent=rna-NM_001298308.1;Dbxref=FLYBASE:FBpp0308524,GeneID:32377,Genbank:NP_001285237.1,FLYBASE:FBgn0030554;Name=NP_001285237.1;gbkey=CDS;gene=CG1434;locus_tag=Dmel_CG1434;orig_transcript_id=gnl|FlyBase|CG1434-RB;product=uncharacterized protein%2C isoform B;protein_id=NP_001285237.1

GO:0005737: Cytoplasm.

Kozak: CCAATGT ID=cds-NP_726887.1;Parent=rna-NM_166990.4;Dbxref=FLYBASE:FBpp0070574,GeneID:43900,Genbank:NP_726887.1,FLYBASE:FBgn0021738;Name=NP_726887.1;gbkey=CDS;gene=Crg-1;locus_tag=Dmel_CG32788;orig_transcript_id=gnl|FlyBase|CG32788-RB;product=circadianly regulated gene;protein_id=NP_726887.1 GO;

GO:0005634: Nucleus.

Kozak: TTAATGC ID=cds-NP_001259114.1;Parent=rna-NM_001272185.2;Dbxref=FLYBASE:FBpp0303735,GeneID:31037,Genbank:NP_001259114.1,FLYBASE:FBgn0040344;Name=NP_001259114.1;gbkey=CDS;gene=Lztr1;locus_tag=Dmel_CG3711;orig_transcript_id=gnl|FlyBase|CG3711-RD;product=leucine zipper like transcription regulator 1%2C isoform D;protein_id=NP_001259114.1;transl_except=(pos:complement(924214..924216)%2Caa:Other)

GO:0005794: Golgi apparatus.

Kozak: TTCATGT ID=cds-NP_001284839.1;Parent=rna-NM_001297910.1;Dbxref=FLYBASE:FBpp0310504,GeneID:31317,Genbank:NP_001284839.1,FLYBASE:FBgn0029663;Name=NP_001284839.1;gbkey=CDS;gene=CG10804;locus_tag=Dmel_CG10804;orig_transcript_id=gnl|FlyBase|CG10804-RD;product=uncharacterized protein%2C isoform D;protein_id=NP_001284839.1

GO:0016021: Integral component of membrane.

Kozak: CGAATGT ID=cds-NP_001259240.1;Parent=rna-NM_001272311.1;Dbxref=FLYBASE:FBpp0307043,GeneID:31392,Genbank:NP_001259240.1,FLYBASE:FBgn0025387;Name=NP_001259240.1;gbkey=CDS;gene=CG12184;locus_tag=Dmel_CG12184;orig_transcript_id=gnl|FlyBase|CG12184-RB;product=uncharacterized protein%2C isoform B;protein_id=NP_001259240.1

GO:0005737: Cytoplasm.

Kozak: TGCATGC ID=cds-NP_572757.2;Parent=rna-NM_132529.4;Dbxref=FLYBASE:FBpp0073438,GeneID:32143,Genbank:NP_572757.2,FLYBASE:FBgn0030344;Name=NP_572757.2;gbkey=CDS;gene=Nrd1;locus_tag=Dmel_CG2025;orig_transcript_id=gnl|FlyBase|CG2025-RA;product=nardilysin;protein_id=NP_572757.2

GO:0101031: Chaperone complex.

Kozak: CCAATGT ID=cds-NP_001162753.1;Parent=rna-NM_001169282.1;Dbxref=FLYBASE:FBpp0291226,GeneID:32400,Genbank:NP_001162753.1,FLYBASE:FBgn0015774;Name=NP_001162753.1;gbkey=CDS;gene=NetB;locus_tag=Dmel_CG10521;orig_transcript_id=gnl|FlyBase|CG10521-RB;product=Netrin-B%2C isoform B;protein_id=NP_001162753.1

GO:0044295: Axonal growth cone.

Kozak: CCCATGT ID=cds-NP_001259572.1;Parent=rna-NM_001272643.1;Dbxref=FLYBASE:FBpp0304842,GeneID:32470,Genbank:NP_001259572.1,FLYBASE:FBgn0040285;Name=NP_001259572.1;gbkey=CDS;gene=Scamp;locus_tag=Dmel_CG9195;orig_transcript_id=gnl|FlyBase|CG9195-RC;product=scamp%2C isoform C;protein_id=NP_001259572.1

GO:0030424: Axon.

Kozak: CCCATGT ID=cds-NP_001014750.1;Parent=rna-NM_001014750.2;Dbxref=FLYBASE:FBpp0289736,GeneID:32771,Genbank:NP_001014750.1,FLYBASE:FBgn0259168;Name=NP_001014750.1;gbkey=CDS;gene=mnb;locus_tag=Dmel_CG42273;orig_transcript_id=gnl|FlyBase|CG42273-RG;product=minibrain%2C isoform G;protein_id=NP_001014750.1

GO:0005634: Nucleus.

Kozak: CCCATGT ID=cds-NP_001285444.1;Parent=rna-NM_001298515.1;Dbxref=FLYBASE:FBpp0310187,GeneID:32946,Genbank:NP_001285444.1,FLYBASE:FBgn0026430;Name=NP_001285444.1;gbkey=CDS;gene=Grip84;locus_tag=Dmel_CG3917;orig_transcript_id=gnl|FlyBase|CG3917-RF;product=Gamma-tubulin ring protein 84%2C isoform F;protein_id=NP_001285444.1

GO:0005737: Cytoplasm.

Kozak: TACATGC ID=cds-NP_523416.2;Parent=rna-NM_078692.2;Dbxref=FLYBASE:FBpp0074571,GeneID:32999,Genbank:NP_523416.2,FLYBASE:FBgn0086782;Name=NP_523416.2;gbkey=CDS;gene=amn;locus_tag=Dmel_CG11937;orig_transcript_id=gnl|FlyBase|CG11937-RA;product=amnesiac;protein_id=NP_523416.2

GO:0005615: Extracellular space.

Kozak: TCCATGT ID=cds-NP_001097034.2;Parent=rna-NM_001103564.2;Dbxref=FLYBASE:FBpp0291450,GeneID:33032,Genbank:NP_001097034.2,FLYBASE:FBgn0031104;Name=NP_001097034.2;gbkey=CDS;gene=jb;locus_tag=Dmel_CG15460;orig_transcript_id=gnl|FlyBase|CG15460-RC;product=jean-baptiste;protein_id=NP_001097034.2

GO:0035074: Pupation.

Kozak: TGCATGC ID=cds-NP_001285335.1;Parent=rna-NM_001298406.1;Dbxref=FLYBASE:FBpp0309137,GeneID:43916,Genbank:NP_001285335.1,FLYBASE:FBgn0026181;Name=NP_001285335.1;gbkey=CDS;gene=Rok;locus_tag=Dmel_CG9774;orig_transcript_id=gnl|FlyBase|CG9774-RB;product=Rho kinase%2C isoform B;protein_id=NP_001285335.1 GO; GO:0015629; C:actin cytoskeleton.

Kozak: CCAATGT ID=cds-NP_001096966.2;Parent=rna-NM_001103496.2;Dbxref=FLYBASE:FBpp0289593,GeneID:5740505,Genbank:NP_001096966.2,FLYBASE:FBgn0085440;Name=NP_001096966.2;gbkey=CDS;gene=Lgr4;locus_tag=Dmel_CG34411;orig_transcript_id=gnl|FlyBase|CG34411-RB;product=Leucine-rich repeat-containing G protein-coupled receptor 4%2C isoform B;protein_id=NP_001096966.2

GO:0016021: Integral component of membrane.

Kozak: CCAATGT ID=cds-NP_788006.1;Parent=rna-NM_175992.2;Dbxref=FLYBASE:FBpp0079336,GeneID:34208,Genbank:NP_788006.1,FLYBASE:FBgn0052986;Name=NP_788006.1;gbkey=CDS;gene=CG32986;locus_tag=Dmel_CG32986;orig_transcript_id=gnl|FlyBase|CG32986-RA;product=uncharacterized protein;protein_id=NP_788006.1

GO:0016021: Integral component of membrane.

Kozak: TACATGC ID=cds-NP_609661.3;Parent=rna-NM_135817.3;Dbxref=FLYBASE:FBpp0080085,GeneID:34769,Genbank:NP_609661.3,FLYBASE:FBgn0028942;Name=NP_609661.3;gbkey=CDS;gene=CG16852;locus_tag=Dmel_CG16852;orig_transcript_id=gnl|FlyBase|CG16852-RA;product=uncharacterized protein;protein_id=NP_609661.3

GO:0016021: Integral component of membrane.

Kozak: TTAATGC ID=cds-NP_001246034.1;Parent=rna-NM_001259105.1;Dbxref=FLYBASE:FBpp0298821,GeneID:34886,Genbank:NP_001246034.1,FLYBASE:FBgn0028879;Name=NP_001246034.1;gbkey=CDS;gene=CG15270;locus_tag=Dmel_CG15270;orig_transcript_id=gnl|FlyBase|CG15270-RC;product=uncharacterized protein%2C isoform C;protein_id=NP_001246034.1

GO:0016021: Integral component of membrane.

Kozak: TGTATGT ID=cds-NP_001260494.1;Parent=rna-NM_001273565.1;Dbxref=FLYBASE:FBpp0308002,GeneID:34962,Genbank:NP_001260494.1,FLYBASE:FBgn0028895;Name=NP_001260494.1;gbkey=CDS;gene=CG17328;locus_tag=Dmel_CG17328;orig_transcript_id=gnl|FlyBase|CG17328-RB;product=uncharacterized protein%2C isoform B;protein_id=NP_001260494.1

GO:0005634: Nucleus.

Kozak: TCCATGT ID=cds-NP_001246062.1;Parent=rna-NM_001259133.2;Dbxref=FLYBASE:FBpp0297292,GeneID:35055,Genbank:NP_001246062.1,FLYBASE:FBgn0032642;Name=NP_001246062.1;gbkey=CDS;gene=CG5110;locus_tag=Dmel_CG5110;orig_transcript_id=gnl|FlyBase|CG5110-RB;product=uncharacterized protein%2C isoform B;protein_id=NP_001246062.1

GO:0071986: Regulator complex.

Kozak: CGAATGT ID=cds-NP_001286028.1;Parent=rna-NM_001299099.1;Dbxref=FLYBASE:FBpp0309463,GeneID:35083,Genbank:NP_001286028.1,FLYBASE:FBgn0032666;Name=NP_001286028.1;gbkey=CDS;gene=CG5758;locus_tag=Dmel_CG5758;orig_transcript_id=gnl|FlyBase|CG5758-RD;product=uncharacterized protein%2C isoform D;protein_id=NP_001286028.1

GO:0031012: Extracellular matrix.

Kozak: TTAATGC ID=cds-NP_542943.2;Parent=rna-NM_080765.4;Dbxref=FLYBASE:FBpp0297339,GeneID:45683,Genbank:NP_542943.2,FLYBASE:FBgn0262872;Name=NP_542943.2;gbkey=CDS;gene=milt;locus_tag=Dmel_CG43227;orig_transcript_id=gnl|FlyBase|CG43227-RC;product=milton%2C isoform C;protein_id=NP_542943.2

GO:1904115: Axon cytoplasm.

Kozak: CCAATGT ID=cds-NP_001260335.1;Parent=rna-NM_001273406.1;Dbxref=FLYBASE:FBpp0304506,GeneID:2768916,Genbank:NP_001260335.1,FLYBASE:FBgn0053303;Name=NP_001260335.1;gbkey=CDS;gene=CG33303;locus_tag=Dmel_CG33303;orig_transcript_id=gnl|FlyBase|CG33303-RB;product=uncharacterized protein%2C isoform B;protein_id=NP_001260335.1

GO:0012505: Endomembrane system.

Kozak: TGGATGC ID=cds-NP_001014459.2;Parent=rna-NM_001014459.2;Dbxref=FLYBASE:FBpp0289895,GeneID:3346208,Genbank:NP_001014459.2,FLYBASE:FBgn0053516;Name=NP_001014459.2;gbkey=CDS;gene=dpr3;locus_tag=Dmel_CG33516;orig_transcript_id=gnl|FlyBase|CG33516-RB;product=defective proboscis extension response 3%2C isoform B;protein_id=NP_001014459.2

GO:0005887: Integral component of plasma membrane.

Kozak: CCAATGT ID=cds-NP_001260104.1;Parent=rna-NM_001273175.1;Dbxref=FLYBASE:FBpp0304813,GeneID:33802,Genbank:NP_001260104.1,FLYBASE:FBgn0031733;Name=NP_001260104.1;gbkey=CDS;gene=CG14006;locus_tag=Dmel_CG14006;orig_transcript_id=gnl|FlyBase|CG14006-RB;product=uncharacterized protein%2C isoform B;protein_id=NP_001260104.1

GO:0016021: Integral component of membrane.

Kozak: CCAATGT ID=cds-NP_001162998.1;Parent=rna-NM_001169527.2;Dbxref=FLYBASE:FBpp0290792,GeneID:35045,Genbank:NP_001162998.1,FLYBASE:FBgn0011274;Name=NP_001162998.1;gbkey=CDS;gene=Dif;locus_tag=Dmel_CG6794;orig_transcript_id=gnl|FlyBase|CG6794-RC;product=Dorsal-related immunity factor%2C isoform C;protein_id=NP_001162998.1

GO:0005737: Cytoplasm.

Kozak: TCCATGT ID=cds-NP_610052.1;Parent=rna-NM_136208.4;Dbxref=FLYBASE:FBpp0080925,GeneID:35334,Genbank:NP_610052.1,FLYBASE:FBgn0032879;Name=NP_610052.1;gbkey=CDS;gene=CarT;locus_tag=Dmel_CG9317;orig_transcript_id=gnl|FlyBase|CG9317-RA;product=carcinine transporter%2C isoform A;protein_id=NP_610052.1

GO:0043679: Axon terminus.

Kozak: CCAATGT ID=cds-NP_001188761.1;Parent=rna-NM_001201832.2;Dbxref=FLYBASE:FBpp0292912,GeneID:45248,Genbank:NP_001188761.1,FLYBASE:FBgn0028704;Name=NP_001188761.1;gbkey=CDS;gene=Nckx30C;locus_tag=Dmel_CG18660;orig_transcript_id=gnl|FlyBase|CG18660-RE;product=Nckx30C%2C isoform E;protein_id=NP_001188761.1

GO:0016021: Integral component of membrane.

Kozak: CCAATGT ID=cds-NP_001245425.1;Parent=rna-NM_001258496.3;Dbxref=FLYBASE:FBpp0297996,GeneID:43795,Genbank:NP_001245425.1,FLYBASE:FBgn0004607;Name=NP_001245425.1;gbkey=CDS;gene=zfh2;locus_tag=Dmel_CG1449;orig_transcript_id=gnl|FlyBase|CG1449-RB;product=Zn finger homeodomain 2%2C isoform B;protein_id=NP_001245425.1

GO:0005634: Nucleus.

Kozak: TGTATGC ID=cds-NP_001303591.1;Parent=rna-NM_001316662.1;Dbxref=FLYBASE:FBpp0312570,GeneID:26067070,Genbank:NP_001303591.1,FLYBASE:FBgn0267904;Name=NP_001303591.1;gbkey=CDS;gene=CG46192;locus_tag=Dmel_CG46192;orig_transcript_id=gnl|FlyBase|CG46192-RA;product=uncharacterized protein;protein_id=NP_001303591.1

GO:0035092: Sperm DNA condensation.
