# Supplementary material for: Functional analysis of the AUG initiator codon context reveals novel conserved sequences that disfavor mRNA translation in eukaryotes
Source: Nucleic Acids Res. 2023 Dec 1;52(3):1064–79. doi: 10.1093/nar/gkad1152 (PMC10853783; doi:10.1093/nar/gkad1152)
Supplement: gkad1152_supplemental_files [file gkad1152_supplemental_files.zip › Suppl. Table 8.docx]

**Supplemental Table 8**

Annotated Gene Ontology (GO) of BACS-containing genes in **Wheat.**

***n* = 1504**.

**GO annotation**

Kozak: TGTATGT ID=cds-XP_044343924.1;Parent=rna-XM_044487989.1;Dbxref=GeneID:123064522,Genbank:XP_044343924.1;Name=XP_044343924.1;gbkey=CDS;gene=LOC123064522;product=zinc finger protein GIS3-like;protein_id=XP_044343924.1

GO:0005634; Nucleus.

Kozak: TGTATGT ID=cds-XP_044344819.1;Parent=rna-XM_044488884.1;Dbxref=GeneID:123065639,Genbank:XP_044344819.1;Name=XP_044344819.1;gbkey=CDS;gene=LOC123065639;product=uncharacterized protein LOC123065639;protein_id=XP_044344819.1

GO:0003824; Catalytic activity;

Kozak: CCCATGT ID=cds-XP_044345006.1;Parent=rna-XM_044489071.1;Dbxref=GeneID:123065868,Genbank:XP_044345006.1;Name=XP_044345006.1;gbkey=CDS;gene=LOC123065868;product=uncharacterized protein At5g39865-like;protein_id=XP_044345006.1

GO:0097573; Glutathione oxidoreductase activity.

Kozak: CAGATGC ID=cds-XP_044345759.1;Parent=rna-XM_044489824.1;Dbxref=GeneID:123066819,Genbank:XP_044345759.1;Name=XP_044345759.1;gbkey=CDS;gene=LOC123066819;product=E3 ubiquitin-protein ligase RNF170-like;protein_id=XP_044345759.1

GO:0005789; Endoplasmic reticulum membrane.

Kozak: CCCATGC ID=cds-XP_044346004.1;Parent=rna-XM_044490069.1;Dbxref=GeneID:123067148,Genbank:XP_044346004.1;Name=XP_044346004.1;gbkey=CDS;gene=LOC123067148;product=

Anaphase-promoting complex subunit 10-like;protein_id=XP_044346004.1

Kozak: TACATGC ID=cds-XP_044346086.1;Parent=rna-XM_044490151.1;Dbxref=GeneID:123067287,Genbank:XP_044346086.1;Name=XP_044346086.1;gbkey=CDS;gene=LOC123067287;product=indole-2-monooxygenase-like;protein_id=XP_044346086.1

GO:0016021; Integral component of membrane.

Kozak: CAGATGC ID=cds-XP_044346110.1;Parent=rna-XM_044490175.1;Dbxref=GeneID:123067339,Genbank:XP_044346110.1;Name=XP_044346110.1;gbkey=CDS;gene=LOC123067339;product=coatomer subunit beta'-2-like;protein_id=XP_044346110.1

GO:0030126; COPI vesicle coat.

Kozak: CCCATGC ID=cds-XP_044346152.1;Parent=rna-XM_044490217.1;Dbxref=GeneID:123067413,Genbank:XP_044346152.1;Name=XP_044346152.1;gbkey=CDS;gene=LOC123067413;P

Product=probable xyloglucan endotransglucosylase/hydrolase protein 25;

Kozak: CAGATGC ID=cds-XP_044346269.1;Parent=rna-XM_044490334.1;Dbxref=GeneID:123067603,Genbank:XP_044346269.1;Name=XP_044346269.1;gbkey=CDS;gene=LOC123067603;product=cysteine-rich receptor-like protein kinase 10;protein_id=XP_044346269.1

GO:0005886; Plasma membrane.

Kozak: CCCATGT ID=cds-XP_044346537.1;Parent=rna-XM_044490602.1;Dbxref=GeneID:123068117,Genbank:XP_044346537.1;Name=XP_044346537.1;gbkey=CDS;gene=LOC123068117;product=

Dof zinc finger protein DOF5.1-like.

Kozak: CAGATGC ID=cds-XP_044346621.1;Parent=rna-XM_044490686.1;Dbxref=GeneID:123068199,Genbank:XP_044346621.1;Name=XP_044346621.1;gbkey=CDS;gene=LOC123068199;product=dephospho-CoA kinase-like;protein_id=XP_044346621.1

GO:0005524; ATP binding.

Kozak: CAGATGC ID=cds-XP_044346861.1;Parent=rna-XM_044490926.1;Dbxref=GeneID:123068351,Genbank:XP_044346861.1;Name=XP_044346861.1;gbkey=CDS;gene=LOC123068351;product=uncharacterized protein LOC123068351;protein_id=XP_044346861.1

GO:0005739; Mitochondrion.

Kozak: CCCATGC ID=cds-XP_044347060.1;Parent=rna-XM_044491125.1;Dbxref=GeneID:123068535,Genbank:XP_044347060.1;Name=XP_044347060.1;gbkey=CDS;gene=LOC123068535;product=receptor kinase-like protein Xa21;protein_id=XP_044347060.1

GO:0016021; Integral component of membrane.

Kozak: CCCATGT ID=cds-XP_044347233.1;Parent=rna-XM_044491298.1;Dbxref=GeneID:123068680,Genbank:XP_044347233.1;Name=XP_044347233.1;gbkey=CDS;gene=LOC123068680;product=KIN17-like protein;protein_id=XP_044347233.1

GO:0005634; Nucleus.

Kozak: TCCATGT ID=cds-XP_044347264.1;Parent=rna-XM_044491329.1;Dbxref=GeneID:123068708,Genbank:XP_044347264.1;Name=XP_044347264.1;gbkey=CDS;gene=LOC123068708;product=uncharacterized protein LOC123068708;protein_id=XP_044347264.1

GO:0016021; Integral component of membrane.

Kozak: CCAATGC ID=cds-XP_044347442.1;Parent=rna-XM_044491507.1;Dbxref=GeneID:123068829,Genbank:XP_044347442.1;Name=XP_044347442.1;gbkey=CDS;gene=LOC123068829;product=

Uncharacterized protein LOC123068829.

Kozak: CAGATGC ID=cds-XP_044347526.1;Parent=rna-XM_044491591.1;Dbxref=GeneID:123068900,Genbank:XP_044347526.1;Name=XP_044347526.1;gbkey=CDS;gene=LOC123068900;product=transcription factor MYBS1-like;protein_id=XP_044347526.1

GO:0003677; DNA binding.

Kozak: CCAATGC ID=cds-XP_044347763.1;Parent=rna-XM_044491828.1;Dbxref=GeneID:123069085,Genbank:XP_044347763.1;Name=XP_044347763.1;gbkey=CDS;gene=LOC123069085;product=O-fucosyltransferase 1-like isoform X1;protein_id=XP_044347763.1

GO:0005737; Cytoplasm.

Kozak: CCCATGT ID=cds-XP_044348132.1;Parent=rna-XM_044492197.1;Dbxref=GeneID:123069361,Genbank:XP_044348132.1;Name=XP_044348132.1;gbkey=CDS;gene=LOC123069361;product=Auxin-responsive protein IAA3-like;protein_id=XP_044348132.1

GO:0005634; Nucleus.

Kozak: CAGATGC ID=cds-XP_044348420.1;Parent=rna-XM_044492485.1;Dbxref=GeneID:123069600,Genbank:XP_044348420.1;Name=XP_044348420.1;gbkey=CDS;gene=LOC123069600;product=bifunctional phosphatase IMPL2%2C chloroplastic-like;protein_id=XP_044348420.1

GO:0004401; Histidinol-phosphatase activity.

Kozak: CCCATGT ID=cds-XP_044349242.1;Parent=rna-XM_044493307.1;Dbxref=GeneID:123070224,Genbank:XP_044349242.1;Name=XP_044349242.1;gbkey=CDS;gene=LOC123070224;product=uncharacterized protein LOC123070224;protein_id=XP_044349242.1

GO:0004842; Ubiquitin-protein transferase activity.

Kozak: CCAATGC ID=cds-XP_044349452.1;Parent=rna-XM_044493517.1;Dbxref=GeneID:123070357,Genbank:XP_044349452.1;Name=XP_044349452.1;gbkey=CDS;gene=LOC123070357;product=

Pentatricopeptide repeat-containing protein At4g02750-like.

Kozak: TCCATGT ID=cds-XP_044349461.1;Parent=rna-XM_044493526.1;Dbxref=GeneID:123070360,Genbank:XP_044349461.1;Name=XP_044349461.1;gbkey=CDS;gene=LOC123070360;product=ras-related protein Rab7-like;protein_id=XP_044349461.1

GO:0005774; Vacuolar membrane.

Kozak: CCCATGT ID=cds-XP_044349462.1;Parent=rna-XM_044493527.1;Dbxref=GeneID:123070361,Genbank:XP_044349462.1;Name=XP_044349462.1;gbkey=CDS;gene=LOC123070361;product=

Protein PLASTID MOVEMENT IMPAIRED 1-RELATED 1-like.

Kozak: TTAATGC ID=cds-XP_044349527.1;Parent=rna-XM_044493592.1;Dbxref=GeneID:123070388,Genbank:XP_044349527.1;Name=XP_044349527.1;gbkey=CDS;gene=LOC123070388;product=probable cytokinin riboside 5'-monophosphate phosphoribohydrolase LOGL1;protein_id=XP_044349527.1

GO:0005829; Cytosol.

Kozak: CCAATGT ID=cds-XP_044349595.1;Parent=rna-XM_044493660.1;Dbxref=GeneID:123070435,Genbank:XP_044349595.1;Name=XP_044349595.1;gbkey=CDS;gene=LOC123070435;product=uncharacterized protein LOC123070435;protein_id=XP_044349595.1

GO:0030599; Pectinesterase activity.

Kozak: CCAATGC ID=cds-XP_044349876.1;Parent=rna-XM_044493941.1;Dbxref=GeneID:123070652,Genbank:XP_044349876.1;Name=XP_044349876.1;gbkey=CDS;gene=LOC123070652;product=lysM domain receptor-like kinase 3 isoform X1;protein_id=XP_044349876.1

GO:0016021; C:integral component of membrane.

Kozak: TACATGC ID=cds-XP_044350272.1;Parent=rna-XM_044494337.1;Dbxref=GeneID:123070927,Genbank:XP_044350272.1;Name=XP_044350272.1;gbkey=CDS;gene=LOC123070927;product=copper transporter 3-like;protein_id=XP_044350272.1

GO:0016021; Integral component of membrane.

Kozak: CCAATGT ID=cds-XP_044350401.1;Parent=rna-XM_044494466.1;Dbxref=GeneID:123071010,Genbank:XP_044350401.1;Name=XP_044350401.1;gbkey=CDS;gene=LOC123071010;product=

External alternative NAD(P)H-ubiquinone oxidoreductase B3%2C mitochondrial-like. GO:0005743; Mitochondrial inner membrane.

Kozak: CCCATGT ID=cds-XP_044350495.1;Parent=rna-XM_044494560.1;Dbxref=GeneID:123071082,Genbank:XP_044350495.1;Name=XP_044350495.1;gbkey=CDS;gene=LOC123071082;product=pentatricopeptide repeat-containing protein At1g33350-like;protein_id=XP_044350495.1

GO:0003986; Acetyl-CoA hydrolase activity.

Kozak: CAGATGC ID=cds-XP_044350496.1;Parent=rna-XM_044494561.1;Dbxref=GeneID:123071083,Genbank:XP_044350496.1;Name=XP_044350496.1;gbkey=CDS;gene=LOC123071083;product=DNA-(apurinic or apyrimidinic site) endonuclease%2C chloroplastic-like isoform X1;protein_id=XP_044350496.1

GO:0005634; Nucleus.

Kozak: TACATGC ID=cds-XP_044350805.1;Parent=rna-XM_044494870.1;Dbxref=GeneID:123071327,Genbank:XP_044350805.1;Name=XP_044350805.1;gbkey=CDS;gene=LOC123071327;product=

ALP1-like.

Kozak: CCCATGC ID=cds-XP_044351076.1;Parent=rna-XM_044495141.1;Dbxref=GeneID:123071563,Genbank:XP_044351076.1;Name=XP_044351076.1;gbkey=CDS;gene=LOC123071563;product=TLC domain-containing protein 2-like;protein_id=XP_044351076.1

GO:0016021; Integral component of membrane.

Kozak: CCAATGT ID=cds-XP_044351128.1;Parent=rna-XM_044495193.1;Dbxref=GeneID:123071612,Genbank:XP_044351128.1;Name=XP_044351128.1;gbkey=CDS;gene=LOC123071612;product=splicing regulator RBM11-like;protein_id=XP_044351128.1

GO:0003723; RNA binding.

Kozak: TTCATGT ID=cds-XP_044351342.1;Parent=rna-XM_044495407.1;Dbxref=GeneID:123071828,Genbank:XP_044351342.1;Name=XP_044351342.1;Note=The sequence of the model RefSeq protein was modified relative to this genomic sequence to represent the inferred CDS: added 15 bases not found in genome assembly;exception=annotated by transcript or proteomic data;gbkey=CDS;gene=LOC123071828;inference=similar to RNA sequence%2C mRNA (same species):INSD:JV912851.1;partial=true;product=

Chromodomain-helicase-DNA-binding protein 3.

Kozak: CAGATGC ID=cds-XP_044351596.1;Parent=rna-XM_044495661.1;Dbxref=GeneID:123072105,Genbank:XP_044351596.1;Name=XP_044351596.1;gbkey=CDS;gene=LOC123072105;product=auxin response factor 4-like;protein_id=XP_044351596.1

GO:0005634; Nucleus.

Kozak: TTAATGC ID=cds-XP_044352183.1;Parent=rna-XM_044496248.1;Dbxref=GeneID:123072645,Genbank:XP_044352183.1;Name=XP_044352183.1;gbkey=CDS;gene=LOC123072645;product=long chain acyl-CoA synthetase 8-like isoform X1;protein_id=XP_044352183.1

GO:0005783; Endoplasmic reticulum.

Kozak: CCCATGC ID=cds-XP_044352195.1;Parent=rna-XM_044496260.1;Dbxref=GeneID:123072665,Genbank:XP_044352195.1;Name=XP_044352195.1;gbkey=CDS;gene=LOC123072665;product=

Translation initiation factor IF-2-like isoform X1.

Kozak: CCCATGC ID=cds-XP_044351437.1;Parent=rna-XM_044495502.1;Dbxref=GeneID:543447,Genbank:XP_044351437.1;Name=XP_044351437.1;gbkey=CDS;gene=LOC543447;product=obg-like ATPase 1;protein_id=XP_044351437.1

GO:0005737; Cytoplasm.

Kozak: TTAATGC ID=cds-XP_044344998.1;Parent=rna-XM_044489063.1;Dbxref=GeneID:778395,Genbank:XP_044344998.1;Name=XP_044344998.1;gbkey=CDS;gene=LOC778395;product=

LOB domain-containing protein 15.

Kozak: TTCATGT ID=cds-XP_044344361.1;Parent=rna-XM_044488426.1;Dbxref=GeneID:123065060,Genbank:XP_044344361.1;Name=XP_044344361.1;gbkey=CDS;gene=LOC123065060;product=aspartic proteinase 36-like isoform X2;protein_id=XP_044344361.1

GO:0004190; Aspartic-type endopeptidase activity.

Kozak: TTCATGT ID=cds-XP_044344366.1;Parent=rna-XM_044488431.1;Dbxref=GeneID:123065063,Genbank:XP_044344366.1;Name=XP_044344366.1;gbkey=CDS;gene=LOC123065063;product=aspartic proteinase 36-like isoform X2;protein_id=XP_044344366.1

GO:0004190; Aspartic-type endopeptidase activity.

Kozak: TCCATGT ID=cds-XP_044344589.1;Parent=rna-XM_044488654.1;Dbxref=GeneID:123065331,Genbank:XP_044344589.1;Name=XP_044344589.1;gbkey=CDS;gene=LOC123065331;product=uncharacterized isomerase BH0283-like;protein_id=XP_044344589.1

GO:0005737; Cytoplasm.

Kozak: TTCATGT ID=cds-XP_044344654.1;Parent=rna-XM_044488719.1;Dbxref=GeneID:123065447,Genbank:XP_044344654.1;Name=XP_044344654.1;Note=The sequence of the model RefSeq protein was modified relative to this genomic sequence to represent the inferred CDS: added 256 bases not found in genome assembly;exception=annotated by transcript or proteomic data;gbkey=CDS;gene=LOC123065447;inference=similar to RNA sequence (same species):INSD:GFFI01064411.1;partial=true;product=

WRKY transcription factor 22.

Kozak: CCAATGT ID=cds-XP_044344777.1;Parent=rna-XM_044488842.1;Dbxref=GeneID:123065592,Genbank:XP_044344777.1;Name=XP_044344777.1;gbkey=CDS;gene=LOC123065592;product=probable purine permease 4;protein_id=XP_044344777.1

GO:0016021; Integral component of membrane.

Kozak: CCCATGC ID=cds-XP_044345065.1;Parent=rna-XM_044489130.1;Dbxref=GeneID:123065951,Genbank:XP_044345065.1;Name=XP_044345065.1;gbkey=CDS;gene=LOC123065951;product=cis-prenyltransferase 4%2C chloroplastic-like;protein_id=XP_044345065.1

GO:0005783; Endoplasmic reticulum.

Kozak: CGAATGT ID=cds-XP_044345184.1;Parent=rna-XM_044489249.1;Dbxref=GeneID:123066097,Genbank:XP_044345184.1;Name=XP_044345184.1;gbkey=CDS;gene=LOC123066097;product=inactive protein kinase SELMODRAFT_444075-like;protein_id=XP_044345184.1

GO:0005886; C:plasma membrane.

Kozak: TCCATGT ID=cds-XP_044346112.1;Parent=rna-XM_044490177.1;Dbxref=GeneID:123067342,Genbank:XP_044346112.1;Name=XP_044346112.1;gbkey=CDS;gene=LOC123067342;product=uncharacterized protein LOC123067342;protein_id=XP_044346112.1

GO:0009507; Chloroplast.

Kozak: CCAATGC ID=cds-XP_044346120.1;Parent=rna-XM_044490185.1;Dbxref=GeneID:123067357,Genbank:XP_044346120.1;Name=XP_044346120.1;gbkey=CDS;gene=LOC123067357;product=

Zinc finger BED domain-containing protein RICESLEEPER 2.

Kozak: CCCATGC ID=cds-XP_044346351.1;Parent=rna-XM_044490416.1;Dbxref=GeneID:123067757,Genbank:XP_044346351.1;Name=XP_044346351.1;gbkey=CDS;gene=LOC123067757;product=uncharacterized protein LOC123067757;protein_id=XP_044346351.1

GO:0005737; Cytoplasm.

Kozak: CCCATGC ID=cds-XP_044346560.1;Parent=rna-XM_044490625.1;Dbxref=GeneID:123068149,Genbank:XP_044346560.1;Name=XP_044346560.1;gbkey=CDS;gene=LOC123068149;product=protein EXECUTER 2%2C chloroplastic-like;protein_id=XP_044346560.1

GO:0042651; Thylakoid membrane.

Kozak: CAGATGC ID=cds-XP_044346996.1;Parent=rna-XM_044491061.1;Dbxref=GeneID:123068480,Genbank:XP_044346996.1;Name=XP_044346996.1;gbkey=CDS;gene=LOC123068480;product=calcineurin B-like protein 9 isoform X2;protein_id=XP_044346996.1

GO:0005509; Calcium ion binding.

Kozak: TTAATGC ID=cds-XP_044347282.1;Parent=rna-XM_044491347.1;Dbxref=GeneID:123068722,Genbank:XP_044347282.1;Name=XP_044347282.1;gbkey=CDS;gene=LOC123068722;product=putative pentatricopeptide repeat-containing protein At2g01510;protein_id=XP_044347282.1

GO:0008270; Zinc ion binding.

Kozak: CCCATGC ID=cds-XP_044347437.1;Parent=rna-XM_044491502.1;Dbxref=GeneID:123068824,Genbank:XP_044347437.1;Name=XP_044347437.1;gbkey=CDS;gene=LOC123068824;product=probable staphylococcal-like nuclease CAN1;protein_id=XP_044347437.1

GO:0005737; Cytoplasm.

Kozak: CCCATGT ID=cds-XP_044347468.1;Parent=rna-XM_044491533.1;Dbxref=GeneID:123068849,Genbank:XP_044347468.1;Name=XP_044347468.1;gbkey=CDS;gene=LOC123068849;product=transcription initiation factor TFIID subunit 15b-like;protein_id=XP_044347468.1

GO:0046872; Metal ion binding.

Kozak: CCCATGC ID=cds-XP_044347651.1;Parent=rna-XM_044491716.1;Dbxref=GeneID:123069000,Genbank:XP_044347651.1;Name=XP_044347651.1;gbkey=CDS;gene=LOC123069000;product=uncharacterized protein LOC123069000;protein_id=XP_044347651.1

GO:0006357; Regulation of transcription by RNA polymerase II.

Kozak: CAGATGC ID=cds-XP_044347694.1;Parent=rna-XM_044491759.1;Dbxref=GeneID:123069028,Genbank:XP_044347694.1;Name=XP_044347694.1;gbkey=CDS;gene=LOC123069028;product=

Transcription factor GTE10-like isoform X1.

Kozak: CCCATGC ID=cds-XP_044347791.1;Parent=rna-XM_044491856.1;Dbxref=GeneID:123069100,Genbank:XP_044347791.1;Name=XP_044347791.1;gbkey=CDS;gene=LOC123069100;product=ABC transporter C family member 3-like;protein_id=XP_044347791.1

GO:0016021; Integral component of membrane.

Kozak: TTCATGT ID=cds-XP_044348567.1;Parent=rna-XM_044492632.1;Dbxref=GeneID:123069703,Genbank:XP_044348567.1;Name=XP_044348567.1;gbkey=CDS;gene=LOC123069703;product=exportin-7-B-like isoform X1;protein_id=XP_044348567.1

GO:0005737; Cytoplasm.

Kozak: TCCATGT ID=cds-XP_044348912.1;Parent=rna-XM_044492977.1;Dbxref=GeneID:123069977,Genbank:XP_044348912.1;Name=XP_044348912.1;gbkey=CDS;gene=LOC123069977;product=protein CONSERVED IN THE GREEN LINEAGE AND DIATOMS 27%2C chloroplastic-like;protein_id=XP_044348912.1 GO:0016021; Integral component of membrane.

Kozak: CCCATGT ID=cds-XP_044349202.1;Parent=rna-XM_044493267.1;Dbxref=GeneID:123070198,Genbank:XP_044349202.1;Name=XP_044349202.1;gbkey=CDS;gene=LOC123070198;product=

TATA-box-binding protein 1-like.

Kozak: CCAATGC ID=cds-XP_044349399.1;Parent=rna-XM_044493464.1;Dbxref=GeneID:123070321,Genbank:XP_044349399.1;Name=XP_044349399.1;gbkey=CDS;gene=LOC123070321;product=

Pentatricopeptide repeat-containing protein At5g46100-like.

Kozak: CCAATGT ID=cds-XP_044349785.1;Parent=rna-XM_044493850.1;Dbxref=GeneID:123070587,Genbank:XP_044349785.1;Name=XP_044349785.1;gbkey=CDS;gene=LOC123070587;product=auxin-responsive protein IAA5-like;protein_id=XP_044349785.1

GO:0005634; Nucleus.

Kozak: TCCATGT ID=cds-XP_044349894.1;Parent=rna-XM_044493959.1;Dbxref=GeneID:123070668,Genbank:XP_044349894.1;Name=XP_044349894.1;gbkey=CDS;gene=LOC123070668;product=serine/threonine-protein phosphatase 7 long form homolog;protein_id=XP_044349894.1

GO:0010073; Meristem maintenance.

Kozak: TTCATGT ID=cds-XP_044350454.1;Parent=rna-XM_044494519.1;Dbxref=GeneID:123071054,Genbank:XP_044350454.1;Name=XP_044350454.1;gbkey=CDS;gene=LOC123071054;product=protein translation factor SUI1 homolog;protein_id=XP_044350454.1

GO:0003723; RNA binding.

Kozak: CCCATGT ID=cds-XP_044351114.1;Parent=rna-XM_044495179.1;Dbxref=GeneID:123071597,Genbank:XP_044351114.1;Name=XP_044351114.1;gbkey=CDS;gene=LOC123071597;product=ubiquitin-like domain-containing CTD phosphatase;protein_id=XP_044351114.1

GO:0046658; Anchored component of plasma membrane.

Kozak: TTAATGC ID=cds-XP_044325898.1;Parent=rna-XM_044469963.1;Dbxref=GeneID:100037522,Genbank:XP_044325898.1;Name=XP_044325898.1;gbkey=CDS;gene=LOC100037522;product=ubiquinol oxidase 1a%2C mitochondrial;protein_id=XP_044325898.1

GO:0016021; Integral component of membrane.

Kozak: CCCATGT ID=cds-XP_044318243.1;Parent=rna-XM_044462308.1;Dbxref=GeneID:123038935,Genbank:XP_044318243.1;Name=XP_044318243.1;gbkey=CDS;gene=LOC123038935;product=ubiquitin-like;protein_id=XP_044318243.1

GO:0005737; Cytoplasm.

Kozak: CCAATGC ID=cds-XP_044318444.1;Parent=rna-XM_044462509.1;Dbxref=GeneID:123039285,Genbank:XP_044318444.1;Name=XP_044318444.1;gbkey=CDS;gene=LOC123039285;product=uncharacterized protein LOC123039285;protein_id=XP_044318444.1

GO:0030125; C:clathrin vesicle coat.

Kozak: TACATGC ID=cds-XP_044318469.1;Parent=rna-XM_044462534.1;Dbxref=GeneID:123039323,Genbank:XP_044318469.1;Name=XP_044318469.1;gbkey=CDS;gene=LOC123039323;product=probable helicase MAGATAMA 3;protein_id=XP_044318469.1

GO:0004386; Helicase activity.

Kozak: TACATGC ID=cds-XP_044318494.1;Parent=rna-XM_044462559.1;Dbxref=GeneID:123039355,Genbank:XP_044318494.1;Name=XP_044318494.1;gbkey=CDS;gene=LOC123039355;product=SKP1-like protein 1B;protein_id=XP_044318494.1

GO:0097602; Cullin family protein binding.

Kozak: CCAATGT ID=cds-XP_044319024.1;Parent=rna-XM_044463089.1;Dbxref=GeneID:123040158,Genbank:XP_044319024.1;Name=XP_044319024.1;gbkey=CDS;gene=LOC123040158;product=protein GOS9-like;protein_id=XP_044319024.1

GO:0030246; Carbohydrate binding.

Kozak: CCAATGT ID=cds-XP_044319188.1;Parent=rna-XM_044463253.1;Dbxref=GeneID:123040392,Genbank:XP_044319188.1;Name=XP_044319188.1;gbkey=CDS;gene=LOC123040392;product=

Zinc finger BED domain-containing protein RICESLEEPER 2-like.

Kozak: CCAATGC ID=cds-XP_044319278.1;Parent=rna-XM_044463343.1;Dbxref=GeneID:123040527,Genbank:XP_044319278.1;Name=XP_044319278.1;gbkey=CDS;gene=LOC123040527;product=ubiquitin-60S ribosomal protein L40-like;protein_id=XP_044319278.1

GO:0005737; Cytoplasm.

Kozak: TCCATGT ID=cds-XP_044319323.1;Parent=rna-XM_044463388.1;Dbxref=GeneID:123040589,Genbank:XP_044319323.1;Name=XP_044319323.1;gbkey=CDS;gene=LOC123040589;product=NAC domain-containing protein 92-like;protein_id=XP_044319323.1

GO:0005634; Nucleus.

Kozak: CCAATGC ID=cds-XP_044319791.1;Parent=rna-XM_044463856.1;Dbxref=GeneID:123041169,Genbank:XP_044319791.1;Name=XP_044319791.1;gbkey=CDS;gene=LOC123041169;product=cysteine-rich receptor-like protein kinase 6;protein_id=XP_044319791.1

GO:0016021; Integral component of membrane.

Kozak: CCAATGC ID=cds-XP_044319965.1;Parent=rna-XM_044464030.1;Dbxref=GeneID:123041413,Genbank:XP_044319965.1;Name=XP_044319965.1;gbkey=CDS;gene=LOC123041413;product=uncharacterized protein LOC123041413;protein_id=XP_044319965.1

GO:0016021; Integral component of membrane.

Kozak: CCAATGT ID=cds-XP_044319970.1;Parent=rna-XM_044464035.1;Dbxref=GeneID:123041418,Genbank:XP_044319970.1;Name=XP_044319970.1;gbkey=CDS;gene=LOC123041418;product=serine/threonine-protein kinase-like protein CCR4;protein_id=XP_044319970.1

GO:0016021; Integral component of membrane.

Kozak: CCAATGC ID=cds-XP_044320257.1;Parent=rna-XM_044464322.1;Dbxref=GeneID:123041757,Genbank:XP_044320257.1;Name=XP_044320257.1;gbkey=CDS;gene=LOC123041757;product=xyloglucan galactosyltransferase XLT2-like;protein_id=XP_044320257.1

GO:0005794; Golgi apparatus.

Kozak: TACATGC ID=cds-XP_044320400.1;Parent=rna-XM_044464465.1;Dbxref=GeneID:123041908,Genbank:XP_044320400.1;Name=XP_044320400.1;gbkey=CDS;gene=LOC123041908;product=probable cation transporter HKT7;protein_id=XP_044320400.1

GO:0016021; Integral component of membrane.

Kozak: CAGATGC ID=cds-XP_044320429.1;Parent=rna-XM_044464494.1;Dbxref=GeneID:123041947,Genbank:XP_044320429.1;Name=XP_044320429.1;gbkey=CDS;gene=LOC123041947;product=pathogenesis-related protein PRMS-like;protein_id=XP_044320429.1

GO:0005615; Extracellular space.

Kozak: CAGATGC ID=cds-XP_044320430.1;Parent=rna-XM_044464495.1;Dbxref=GeneID:123041948,Genbank:XP_044320430.1;Name=XP_044320430.1;gbkey=CDS;gene=LOC123041948;product=pathogenesis-related protein PRMS-like;protein_id=XP_044320430.1

GO:0005615; Extracellular space.

Kozak: CCAATGT ID=cds-XP_044320571.1;Parent=rna-XM_044464636.1;Dbxref=GeneID:123042114,Genbank:XP_044320571.1;Name=XP_044320571.1;gbkey=CDS;gene=LOC123042114;product=putative disease resistance protein RGA1;protein_id=XP_044320571.1

GO:0005634; Nucleus.

Kozak: TTCATGT ID=cds-XP_044320736.1;Parent=rna-XM_044464801.1;Dbxref=GeneID:123042329,Genbank:XP_044320736.1;Name=XP_044320736.1;gbkey=CDS;gene=LOC123042329;product=heavy metal-associated isoprenylated plant protein 2-like;protein_id=XP_044320736.1

GO:0046872; Metal ion binding.

Kozak: TGTATGT ID=cds-XP_044320947.1;Parent=rna-XM_044465012.1;Dbxref=GeneID:123042582,Genbank:XP_044320947.1;Name=XP_044320947.1;gbkey=CDS;gene=LOC123042582;product=cysteine-rich receptor-like protein kinase 10;protein_id=XP_044320947.1

GO:0016021; Integral component of membrane.

Kozak: TACATGC ID=cds-XP_044321293.1;Parent=rna-XM_044465358.1;Dbxref=GeneID:123043023,Genbank:XP_044321293.1;Name=XP_044321293.1;gbkey=CDS;gene=LOC123043023;product=cysteine-rich receptor-like protein kinase 28 isoform X1;protein_id=XP_044321293.1

GO:0005524; ATP binding.

Kozak: TCCATGT ID=cds-XP_044321802.1;Parent=rna-XM_044465867.1;Dbxref=GeneID:123043416,Genbank:XP_044321802.1;Name=XP_044321802.1;gbkey=CDS;gene=LOC123043416;product=probable LRR receptor-like serine/threonine-protein kinase At3g47570;protein_id=XP_044321802.1 GO;

GO:0016021; Integral component of membrane.

Kozak: CCCATGT ID=cds-XP_044322076.1;Parent=rna-XM_044466141.1;Dbxref=GeneID:123043628,Genbank:XP_044322076.1;Name=XP_044322076.1;gbkey=CDS;gene=LOC123043628;product=RINT1-like protein MAG2L isoform X1;protein_id=XP_044322076.1

GO:0070939; Dsl1/NZR complex.

Kozak: CCAATGC ID=cds-XP_044322927.1;Parent=rna-XM_044466992.1;Dbxref=GeneID:123044288,Genbank:XP_044322927.1;Name=XP_044322927.1;gbkey=CDS;gene=LOC123044288;product=uncharacterized hydrolase YugF-like;protein_id=XP_044322927.1

GO:0003824; Catalytic activity.

Kozak: CGAATGT ID=cds-XP_044323010.1;Parent=rna-XM_044467075.1;Dbxref=GeneID:123044350,Genbank:XP_044323010.1;Name=XP_044323010.1;gbkey=CDS;gene=LOC123044350;product=zinc finger SWIM domain-containing protein 7-like;protein_id=XP_044323010.1

GO:0097196; Shu complex.

Kozak: CCCATGC ID=cds-XP_044323599.1;Parent=rna-XM_044467664.1;Dbxref=GeneID:123044778,Genbank:XP_044323599.1;Name=XP_044323599.1;gbkey=CDS;gene=LOC123044778;product=uncharacterized protein LOC123044778 isoform X1;protein_id=XP_044323599.1

GO:0016021; Integral component of membrane.

Kozak: CCCATGC ID=cds-XP_044323702.1;Parent=rna-XM_044467767.1;Dbxref=GeneID:123044886,Genbank:XP_044323702.1;Name=XP_044323702.1;gbkey=CDS;gene=LOC123044886;product=AT-rich interactive domain-containing protein 6-like;protein_id=XP_044323702.1

GO:0005634; Nucleus.

Kozak: CCCATGC ID=cds-XP_044323820.1;Parent=rna-XM_044467885.1;Dbxref=GeneID:123044978,Genbank:XP_044323820.1;Name=XP_044323820.1;gbkey=CDS;gene=LOC123044978;product=probable LRR receptor-like serine/threonine-protein kinase At2g16250;protein_id=XP_044323820.1

GO:0016021; Integral component of membrane.

Kozak: CCCATGT ID=cds-XP_044323834.1;Parent=rna-XM_044467899.1;Dbxref=GeneID:123044991,Genbank:XP_044323834.1;Name=XP_044323834.1;gbkey=CDS;gene=LOC123044991;product=uncharacterized oxidoreductase At1g06690%2C chloroplastic-like isoform X2;protein_id=XP_044323834.1 GO; GO:0005737; cytoplasm.

Kozak: CCCATGT ID=cds-XP_044324329.1;Parent=rna-XM_044468394.1;Dbxref=GeneID:123045364,Genbank:XP_044324329.1;Name=XP_044324329.1;gbkey=CDS;gene=LOC123045364;product=uncharacterized protein At1g32220%2C chloroplastic-like;protein_id=XP_044324329.1

GO:0006952; Defense response.

Kozak: CCCATGT ID=cds-XP_044324346.1;Parent=rna-XM_044468411.1;Dbxref=GeneID:123045381,Genbank:XP_044324346.1;Name=XP_044324346.1;gbkey=CDS;gene=LOC123045381;product=cytochrome P450 77A4-like;protein_id=XP_044324346.1

GO:0016021; Integral component of membrane.

Kozak: TACATGC ID=cds-XP_044324434.1;Parent=rna-XM_044468499.1;Dbxref=GeneID:123045444,Genbank:XP_044324434.1;Name=XP_044324434.1;gbkey=CDS;gene=LOC123045444;product=RING-H2 finger protein ATL72-like;protein_id=XP_044324434.1

GO:0016021; Integral component of membrane.

Kozak: CCAATGC ID=cds-XP_044324785.1;Parent=rna-XM_044468850.1;Dbxref=GeneID:123045699,Genbank:XP_044324785.1;Name=XP_044324785.1;gbkey=CDS;gene=LOC123045699;product=protein ALTERED XYLOGLUCAN 4-like;protein_id=XP_044324785.1

GO:0005794; Golgi apparatus.

Kozak: CCCATGT ID=cds-XP_044324872.1;Parent=rna-XM_044468937.1;Dbxref=GeneID:123045764,Genbank:XP_044324872.1;Name=XP_044324872.1;gbkey=CDS;gene=LOC123045764;product=SWI/SNF complex subunit SWI3A homolog;protein_id=XP_044324872.1

GO:0043229; Intracellular organelle.

Kozak: CCCATGT ID=cds-XP_044325020.1;Parent=rna-XM_044469085.1;Dbxref=GeneID:123045863,Genbank:XP_044325020.1;Name=XP_044325020.1;gbkey=CDS;gene=LOC123045863;product=amino acid permease 3-like;protein_id=XP_044325020.1

GO:0016021; Integral component of membrane.

Kozak: CCCATGC ID=cds-XP_044325077.1;Parent=rna-XM_044469142.1;Dbxref=GeneID:123045903,Genbank:XP_044325077.1;Name=XP_044325077.1;gbkey=CDS;gene=LOC123045903;product=(+)-neomenthol dehydrogenase-like isoform X1;protein_id=XP_044325077.1

GO:0016616; Oxidoreductase activity, acting on the CH-OH group of donors, NAD or NADP as acceptor.

Kozak: CAGATGC ID=cds-XP_044325321.1;Parent=rna-XM_044469386.1;Dbxref=GeneID:123046098,Genbank:XP_044325321.1;Name=XP_044325321.1;gbkey=CDS;gene=LOC123046098;product=pentatricopeptide repeat-containing protein At3g53360%2C mitochondrial-like;protein_id=XP_044325321.1

GO:0003723; RNA binding.

Kozak: CCCATGT ID=cds-XP_044325562.1;Parent=rna-XM_044469627.1;Dbxref=GeneID:123046302,Genbank:XP_044325562.1;Name=XP_044325562.1;gbkey=CDS;gene=LOC123046302;product=uncharacterized protein LOC123046302;protein_id=XP_044325562.1

GO:0005730; Nucleolus.

Kozak: TTCATGT ID=cds-XP_044326190.1;Parent=rna-XM_044470255.1;Dbxref=GeneID:123046828,Genbank:XP_044326190.1;Name=XP_044326190.1;gbkey=CDS;gene=LOC123046828;product=receptor kinase-like protein Xa21;protein_id=XP_044326190.1 GO;

GO:0016021; Integral component of membrane.

Kozak: CCCATGT ID=cds-XP_044326621.1;Parent=rna-XM_044470686.1;Dbxref=GeneID:123047191,Genbank:XP_044326621.1;Name=XP_044326621.1;gbkey=CDS;gene=LOC123047191;product=

Phenolic glucoside malonyltransferase 2-like.

Kozak: TACATGC ID=cds-XP_044322709.1;Parent=rna-XM_044466774.1;Dbxref=GeneID:780628,Genbank:XP_044322709.1;Name=XP_044322709.1;gbkey=CDS;gene=LOC780628;product=putative MYST-like histone acetyltransferase 1;protein_id=XP_044322709.1

GO:0004402; Histone acetyltransferase activity.

Kozak: CCAATGC ID=cds-XP_044323944.1;Parent=rna-XM_044468009.1;Dbxref=GeneID:100873145,Genbank:XP_044323944.1;Name=XP_044323944.1;gbkey=CDS;gene=LOC100873145;product=transcription factor NIGTH1;protein_id=XP_044323944.1

GO:0005634; Nucleus.

Kozak: CCCATGT ID=cds-XP_044318202.1;Parent=rna-XM_044462267.1;Dbxref=GeneID:123038828,Genbank:XP_044318202.1;Name=XP_044318202.1;gbkey=CDS;gene=LOC123038828;product=probable serine/threonine-protein kinase PIX13;protein_id=XP_044318202.1

GO:0005524; F:ATP binding.

Kozak: CCAATGT ID=cds-XP_044318372.1;Parent=rna-XM_044462437.1;Dbxref=GeneID:123039165,Genbank:XP_044318372.1;Name=XP_044318372.1;gbkey=CDS;gene=LOC123039165;product=uncharacterized protein LOC123039165;protein_id=XP_044318372.1

GO:0016021; Integral component of membrane.

Kozak: TTCATGT ID=cds-XP_044318428.1;Parent=rna-XM_044462493.1;Dbxref=GeneID:123039257,Genbank:XP_044318428.1;Name=XP_044318428.1;gbkey=CDS;gene=LOC123039257;product=early nodulin-like protein 1;protein_id=XP_044318428.1

GO:0046658; Anchored component of plasma membrane.

Kozak: CCCATGC ID=cds-XP_044318488.1;Parent=rna-XM_044462553.1;Dbxref=GeneID:123039349,Genbank:XP_044318488.1;Name=XP_044318488.1;gbkey=CDS;gene=LOC123039349;product=

Proline-rich receptor-like protein kinase PERK9.

Kozak: CCCATGT ID=cds-XP_044318617.1;Parent=rna-XM_044462682.1;Dbxref=GeneID:123039570,Genbank:XP_044318617.1;Name=XP_044318617.1;gbkey=CDS;gene=LOC123039570;product=

Metalloendoproteinase 1-like.

Kozak: CCAATGT ID=cds-XP_044318774.1;Parent=rna-XM_044462839.1;Dbxref=GeneID:123039834,Genbank:XP_044318774.1;Name=XP_044318774.1;gbkey=CDS;gene=LOC123039834;product=

zinc finger BED domain-containing protein RICESLEEPER 2-like

Kozak: TACATGC ID=cds-XP_044318870.1;Parent=rna-XM_044462935.1;Dbxref=GeneID:123039960,Genbank:XP_044318870.1;Name=XP_044318870.1;gbkey=CDS;gene=LOC123039960;product=transcription factor TEOSINTE BRANCHED 1-like;protein_id=XP_044318870.1

GO:0005634; Nucleus.

Kozak: CCAATGC ID=cds-XP_044318949.1;Parent=rna-XM_044463014.1;Dbxref=GeneID:123040073,Genbank:XP_044318949.1;Name=XP_044318949.1;gbkey=CDS;gene=LOC123040073;product=uncharacterized protein LOC123040073;protein_id=XP_044318949.1

GO:0000145; Exocyst.

Kozak: CCAATGC ID=cds-XP_044319277.1;Parent=rna-XM_044463342.1;Dbxref=GeneID:123040526,Genbank:XP_044319277.1;Name=XP_044319277.1;gbkey=CDS;gene=LOC123040526;product=ubiquitin-60S ribosomal protein L40-like;protein_id=XP_044319277.1

GO:0005737; Cytoplasm.

Kozak: CCAATGT ID=cds-XP_044319329.1;Parent=rna-XM_044463394.1;Dbxref=GeneID:123040597,Genbank:XP_044319329.1;Name=XP_044319329.1;gbkey=CDS;gene=LOC123040597;product=ubiquitin-like-specific protease ESD4 isoform X2;protein_id=XP_044319329.1

GO:0005634; Nucleus.

Kozak: CCCATGC ID=cds-XP_044319383.1;Parent=rna-XM_044463448.1;Dbxref=GeneID:123040671,Genbank:XP_044319383.1;Name=XP_044319383.1;gbkey=CDS;gene=LOC123040671;product=ubiquitin-like-specific protease 1A;protein_id=XP_044319383.1

GO:0005634; Nucleus.

Kozak: CCAATGC ID=cds-XP_044319710.1;Parent=rna-XM_044463775.1;Dbxref=GeneID:123041076,Genbank:XP_044319710.1;Name=XP_044319710.1;gbkey=CDS;gene=LOC123041076;product=predicted GPI-anchored protein 58;protein_id=XP_044319710.1

GO:0110165; Cellular anatomical entity.

Kozak: CCAATGC ID=cds-XP_044319964.1;Parent=rna-XM_044464029.1;Dbxref=GeneID:123041412,Genbank:XP_044319964.1;Name=XP_044319964.1;gbkey=CDS;gene=LOC123041412;product=putative B3 domain-containing protein Os03g0621600;protein_id=XP_044319964.1

GO:0005634; C:nucleus.

Kozak: CCCATGT ID=cds-XP_044319985.1;Parent=rna-XM_044464050.1;Dbxref=GeneID:123041440,Genbank:XP_044319985.1;Name=XP_044319985.1;gbkey=CDS;gene=LOC123041440;product=

Extensin-like.

Kozak: CCCATGC ID=cds-XP_044319995.1;Parent=rna-XM_044464060.1;Dbxref=GeneID:123041456,Genbank:XP_044319995.1;Name=XP_044319995.1;gbkey=CDS;gene=LOC123041456;product=uncharacterized protein LOC123041456;protein_id=XP_044319995.1

GO:0005634; Nucleus

Kozak: CCCATGT ID=cds-XP_044320171.1;Parent=rna-XM_044464236.1;Dbxref=GeneID:123041651,Genbank:XP_044320171.1;Name=XP_044320171.1;gbkey=CDS;gene=LOC123041651;product=uncharacterized protein At4g19900-like;protein_id=XP_044320171.1

GO:0016021; Integral component of membrane

Kozak: TCCATGT ID=cds-XP_044320698.1;Parent=rna-XM_044464763.1;Dbxref=GeneID:123042283,Genbank:XP_044320698.1;Name=XP_044320698.1;gbkey=CDS;gene=LOC123042283;product=probable folate-biopterin transporter 2;protein_id=XP_044320698.1

GO:0016021; Integral component of membrane

Kozak: CCCATGT ID=cds-XP_044320918.1;Parent=rna-XM_044464983.1;Dbxref=GeneID:123042552,Genbank:XP_044320918.1;Name=XP_044320918.1;gbkey=CDS;gene=LOC123042552;product=metalloendoproteinase 1-MMP-like;protein_id=XP_044320918.1

GO:0031225; Anchored component of membrane

Kozak: CCCATGC ID=cds-XP_044321190.1;Parent=rna-XM_044465255.1;Dbxref=GeneID:123042892,Genbank:XP_044321190.1;Name=XP_044321190.1;gbkey=CDS;gene=LOC123042892;product=

nicotinate N-methyltransferase 1-like.

Kozak: TTCATGT ID=cds-XP_044321379.1;Parent=rna-XM_044465444.1;Dbxref=GeneID:123043092,Genbank:XP_044321379.1;Name=XP_044321379.1;gbkey=CDS;gene=LOC123043092;product=probable LRR receptor-like serine/threonine-protein kinase At3g47570;protein_id=XP_044321379.1

GO:0016021; Integral component of membrane

Kozak: CCAATGC ID=cds-XP_044322390.1;Parent=rna-XM_044466455.1;Dbxref=GeneID:123043868,Genbank:XP_044322390.1;Name=XP_044322390.1;gbkey=CDS;gene=LOC123043868;product=phosphatidylinositol 4-phosphate 5-kinase 6-like;protein_id=XP_044322390.1

GO:0005886; Plasma membrane

Kozak: CGAATGT ID=cds-XP_044322539.1;Parent=rna-XM_044466604.1;Dbxref=GeneID:123043991,Genbank:XP_044322539.1;Name=XP_044322539.1;gbkey=CDS;gene=LOC123043991;product=probable cytochrome P450 313a4;protein_id=XP_044322539.1

GO:0016021; Integral component of membrane

Kozak: CCAATGC ID=cds-XP_044322624.1;Parent=rna-XM_044466689.1;Dbxref=GeneID:123044064,Genbank:XP_044322624.1;Name=XP_044322624.1;gbkey=CDS;gene=LOC123044064;product=probable transcription factor At5g28040;protein_id=XP_044322624.1

GO:0005634; Nucleus

Kozak: CAGATGC ID=cds-XP_044322772.1;Parent=rna-XM_044466837.1;Dbxref=GeneID:123044179,Genbank:XP_044322772.1;Name=XP_044322772.1;gbkey=CDS;gene=LOC123044179;product=probable inactive purple acid phosphatase 16;protein_id=XP_044322772.1

GO:0016788; Hydrolase activity, acting on ester bonds

Kozak: CCCATGT ID=cds-XP_044323049.1;Parent=rna-XM_044467114.1;Dbxref=GeneID:123044386,Genbank:XP_044323049.1;Name=XP_044323049.1;gbkey=CDS;gene=LOC123044386;product=uncharacterized protein LOC123044386 isoform X1;protein_id=XP_044323049.1

GO:0005789; Endoplasmic reticulum membrane

Kozak. CCCATGC ID=cds-XP_044323515.1;Parent=rna-XM_044467580.1;Dbxref=GeneID:123044739,Genbank:XP_044323515.1;Name=XP_044323515.1;gbkey=CDS;gene=LOC123044739;product=protein trichome birefringence-like 13 isoform X1;protein_id=XP_044323515.1

GO:0005794; Golgi apparatus

Kozak: CCAATGC ID=cds-XP_044323819.1;Parent=rna-XM_044467884.1;Dbxref=GeneID:123044977,Genbank:XP_044323819.1;Name=XP_044323819.1;gbkey=CDS;gene=LOC123044977;product=leucine-rich repeat receptor-like serine/threonine-protein kinase BAM1;protein_id=XP_044323819.1

GO:0016021; Integral component of membrane

Kozak: TACATGC ID=cds-XP_044324008.1;Parent=rna-XM_044468073.1;Dbxref=GeneID:123045128,Genbank:XP_044324008.1;Name=XP_044324008.1;gbkey=CDS;gene=LOC123045128;product=dirigent protein 1-like;protein_id=XP_044324008.1

GO:0048046; Apoplast

Kozak: CGAATGT ID=cds-XP_044324194.1;Parent=rna-XM_044468259.1;Dbxref=GeneID:123045267,Genbank:XP_044324194.1;Name=XP_044324194.1;gbkey=CDS;gene=LOC123045267;product=uncharacterized protein LOC123045267;protein_id=XP_044324194.1

GO:0016021; Integral component of membrane

Kozak: TTAATGC ID=cds-XP_044324243.1;Parent=rna-XM_044468308.1;Dbxref=GeneID:123045306,Genbank:XP_044324243.1;Name=XP_044324243.1;gbkey=CDS;gene=LOC123045306;product=uncharacterized protein LOC123045306;protein_id=XP_044324243.1

GO:0016021; Integral component of membrane

Kozak: CCAATGC ID=cds-XP_044324744.1;Parent=rna-XM_044468809.1;Dbxref=GeneID:123045666,Genbank:XP_044324744.1;Name=XP_044324744.1;gbkey=CDS;gene=LOC123045666;product=

pentatricopeptide repeat-containing protein At4g26680%2C mitochondrial-like;protein

Kozak: CCAATGC ID=cds-XP_044325173.1;Parent=rna-XM_044469238.1;Dbxref=GeneID:123045973,Genbank:XP_044325173.1;Name=XP_044325173.1;gbkey=CDS;gene=LOC123045973;product=DNA mismatch repair protein MSH1%2C mitochondrial-like isoform X2;protein_id=XP_044325173.1

GO:0005739; Mitochondrion

Kozak: CCAATGC ID=cds-XP_044325426.1;Parent=rna-XM_044469491.1;Dbxref=GeneID:123046186,Genbank:XP_044325426.1;Name=XP_044325426.1;gbkey=CDS;gene=LOC123046186;product=ubiquitin carboxyl-terminal hydrolase 23-like;protein_id=XP_044325426.1

GO:0005829; Cytosol

Kozak: CAGATGC ID=cds-XP_044325614.1;Parent=rna-XM_044469679.1;Dbxref=GeneID:123046348,Genbank:XP_044325614.1;Name=XP_044325614.1;Note=The sequence of the model RefSeq protein was modified relative to this genomic sequence to represent the inferred CDS: added 250 bases not found in genome assembly;end_range=620732603,.;exception=annotated by transcript or proteomic data;gbkey=CDS;gene=LOC123046348;inference=similar to RNA sequence (same species):INSD:GFFI01048180.1;partial=true;product=polyubiquitin-like;protein_id=XP_044325614.1

GO:0005737; Cytoplasm

Kozak: CCCATGT ID=cds-XP_044325788.1;Parent=rna-XM_044469853.1;Dbxref=GeneID:123046477,Genbank:XP_044325788.1;Name=XP_044325788.1;gbkey=CDS;gene=LOC123046477;product=ent-kaur-16-ene synthase%2C chloroplastic-like isoform X1;protein_id=XP_044325788.1

GO:0000287; Magnesium ion binding

Kozak: TCCATGT ID=cds-XP_044325806.1;Parent=rna-XM_044469871.1;Dbxref=GeneID:123046487,Genbank:XP_044325806.1;Name=XP_044325806.1;gbkey=CDS;gene=LOC123046487;product=

late embryogenesis abundant protein 19-like;protein

Kozak: TACATGC ID=cds-XP_044326015.1;Parent=rna-XM_044470080.1;Dbxref=GeneID:123046674,Genbank:XP_044326015.1;Name=XP_044326015.1;gbkey=CDS;gene=LOC123046674;product=receptor-like protein kinase FERONIA;protein_id=XP_044326015.1

GO:0016021; Integral component of membrane

Kozak: TCCATGT ID=cds-XP_044326561.1;Parent=rna-XM_044470626.1;Dbxref=GeneID:123047138,Genbank:XP_044326561.1;Name=XP_044326561.1;gbkey=CDS;gene=LOC123047138;product=putative chloride channel-like protein CLC-g isoform X1;protein_id=XP_044326561.1

GO:0016021; Integral component of membrane

Kozak: TCCATGT ID=cds-XP_044340884.1;Parent=rna-XM_044484949.1;Dbxref=GeneID:101290584,Genbank:XP_044340884.1;Name=XP_044340884.1;gbkey=CDS;gene=LOC101290584;product=ras-related protein Rab7;protein_id=XP_044340884.1

GO:0005774; Vacuolar membrane

Kozak: CCCATGT ID=cds-XP_044336058.1;Parent=rna-XM_044480123.1;Dbxref=GeneID:123056888,Genbank:XP_044336058.1;Name=XP_044336058.1;gbkey=CDS;gene=LOC123056888;product=

WAS/WASL-interacting protein family member 2-like;protein

Kozak: CCAATGC ID=cds-XP_044336134.1;Parent=rna-XM_044480199.1;Dbxref=GeneID:123057020,Genbank:XP_044336134.1;Name=XP_044336134.1;gbkey=CDS;gene=LOC123057020;product=

40S ribosomal protein S26-like;protein

Kozak: CCCATGC ID=cds-XP_044336153.1;Parent=rna-XM_044480218.1;Dbxref=GeneID:123057048,Genbank:XP_044336153.1;Name=XP_044336153.1;gbkey=CDS;gene=LOC123057048;product=subtilisin-like protease SBT3;protein_id=XP_044336153.1

GO:0016020; Membrane

Kozak: CCCATGT ID=cds-XP_044336414.1;Parent=rna-XM_044480479.1;Dbxref=GeneID:123057528,Genbank:XP_044336414.1;Name=XP_044336414.1;gbkey=CDS;gene=LOC123057528;product=probable glucomannan 4-beta-mannosyltransferase 11;protein_id=XP_044336414.1

GO:0005794; Golgi apparatus

Kozak: CAGATGC ID=cds-XP_044336622.1;Parent=rna-XM_044480687.1;Dbxref=GeneID:123057798,Genbank:XP_044336622.1;Name=XP_044336622.1;gbkey=CDS;gene=LOC123057798;product=uncharacterized protein LOC123057798;protein_id=XP_044336622.1

GO:0006952; Defense response

Kozak: CCAATGT ID=cds-XP_044337120.1;Parent=rna-XM_044481185.1;Dbxref=GeneID:123058466,Genbank:XP_044337120.1;Name=XP_044337120.1;gbkey=CDS;gene=LOC123058466;product=uncharacterized protein LOC123058466;protein_id=XP_044337120.1

GO:0030599; Pectinesterase activity

Kozak: CCCATGC ID=cds-XP_044337308.1;Parent=rna-XM_044481373.1;Dbxref=GeneID:123058668,Genbank:XP_044337308.1;Name=XP_044337308.1;gbkey=CDS;gene=LOC123058668;product=salicylic acid-binding protein 2-like;protein_id=XP_044337308.1

GO:0080030; Methyl indole-3-acetate esterase activity

Kozak: CCCATGC ID=cds-XP_044337309.1;Parent=rna-XM_044481374.1;Dbxref=GeneID:123058669,Genbank:XP_044337309.1;Name=XP_044337309.1;gbkey=CDS;gene=LOC123058669;product=salicylic acid-binding protein 2-like;protein_id=XP_044337309.1

GO:0080030; Methyl indole-3-acetate esterase activity

Kozak: CCCATGC ID=cds-XP_044337419.1;Parent=rna-XM_044481484.1;Dbxref=GeneID:123058809,Genbank:XP_044337419.1;Name=XP_044337419.1;gbkey=CDS;gene=LOC123058809;product=zinc finger protein 6-like;protein_id=XP_044337419.1

GO:0005634; Nucleus

Kozak: CGAATGT ID=cds-XP_044337551.1;Parent=rna-XM_044481616.1;Dbxref=GeneID:123058948,Genbank:XP_044337551.1;Name=XP_044337551.1;gbkey=CDS;gene=LOC123058948;product=inactive protein kinase SELMODRAFT_444075-like;protein_id=XP_044337551.1

GO:0005886; Plasma membrane

Kozak: TCCATGT ID=cds-XP_044337786.1;Parent=rna-XM_044481851.1;Dbxref=GeneID:123059240,Genbank:XP_044337786.1;Name=XP_044337786.1;gbkey=CDS;gene=LOC123059240;product=

F-box protein At5g49610-like;protein

Kozak: CCCATGC ID=cds-XP_044338379.1;Parent=rna-XM_044482444.1;Dbxref=GeneID:123059905,Genbank:XP_044338379.1;Name=XP_044338379.1;gbkey=CDS;gene=LOC123059905;product=

LEAF RUST 10 DISEASE-RESISTANCE LOCUS RECEPTOR-LIKE PROTEIN KINASE-like 1.2;protein

Kozak CCAATGC ID=cds-XP_044339209.1;Parent=rna-XM_044483274.1;Dbxref=GeneID:123060517,Genbank:XP_044339209.1;Name=XP_044339209.1;gbkey=CDS;gene=LOC123060517;product=O-fucosyltransferase 1-like;protein_id=XP_044339209.1

GO:0005737; Cytoplasm

Kozak: TTCATGT ID=cds-XP_044339278.1;Parent=rna-XM_044483343.1;Dbxref=GeneID:123060575,Genbank:XP_044339278.1;Name=XP_044339278.1;gbkey=CDS;gene=LOC123060575;product=B3 domain-containing protein Os03g0620400-like;protein_id=XP_044339278.1

GO:0005634; Nucleus

Kozak: CCCATGT ID=cds-XP_044339579.1;Parent=rna-XM_044483644.1;Dbxref=GeneID:123060802,Genbank:XP_044339579.1;Name=XP_044339579.1;gbkey=CDS;gene=LOC123060802;product=auxin-responsive protein IAA3-like;protein_id=XP_044339579.1

GO:0005634; Nucleus

Kozak: CAGATGC ID=cds-XP_044339799.1;Parent=rna-XM_044483864.1;Dbxref=GeneID:123060988,Genbank:XP_044339799.1;Name=XP_044339799.1;gbkey=CDS;gene=LOC123060988;product=bifunctional phosphatase IMPL2%2C chloroplastic-like;protein_id=XP_044339799.1

GO:0004401; Histidinol-phosphatase activity

Kozak: CCCATGT ID=cds-XP_044340693.1;Parent=rna-XM_044484758.1;Dbxref=GeneID:123061593,Genbank:XP_044340693.1;Name=XP_044340693.1;gbkey=CDS;gene=LOC123061593;product=uncharacterized protein LOC123061593;protein_id=XP_044340693.1

GO:0004842; Ubiquitin-protein transferase activity

Kozak CCCATGC ID=cds-XP_044340812.1;Parent=rna-XM_044484877.1;Dbxref=GeneID:123061679,Genbank:XP_044340812.1;Name=XP_044340812.1;gbkey=CDS;gene=LOC123061679;product=

high mobility group B protein 14-like;protein

Kozak: CCAATGC ID=cds-XP_044340852.1;Parent=rna-XM_044484917.1;Dbxref=GeneID:123061707,Genbank:XP_044340852.1;Name=XP_044340852.1;gbkey=CDS;gene=LOC123061707;product=

pentatricopeptide repeat-containing protein At5g46100-like;protein

Kozak: CCAATGC ID=cds-XP_044340877.1;Parent=rna-XM_044484942.1;Dbxref=GeneID:123061724,Genbank:XP_044340877.1;Name=XP_044340877.1;gbkey=CDS;gene=LOC123061724;product=

pentatricopeptide repeat-containing protein At4g02750-like;protein

Kozak: CCCATGT ID=cds-XP_044340885.1;Parent=rna-XM_044484950.1;Dbxref=GeneID:123061727,Genbank:XP_044340885.1;Name=XP_044340885.1;gbkey=CDS;gene=LOC123061727;product=

protein PLASTID MOVEMENT IMPAIRED 1-RELATED 1-like;protein

Kozak: CCAATGC ID=cds-XP_044341184.1;Parent=rna-XM_044485249.1;Dbxref=GeneID:123061957,Genbank:XP_044341184.1;Name=XP_044341184.1;gbkey=CDS;gene=LOC123061957;product=lysM domain receptor-like kinase 3;protein_id=XP_044341184.1

GO:0016021; Integral component of membrane

Kozak: CCCATGC ID=cds-XP_044341585.1;Parent=rna-XM_044485650.1;Dbxref=GeneID:123062223,Genbank:XP_044341585.1;Name=XP_044341585.1;gbkey=CDS;gene=LOC123062223;product=small RNA degrading nuclease 5 isoform X1;protein_id=XP_044341585.1

GO:0005634; Nucleus

Kozak: CCAATGT ID=cds-XP_044341634.1;Parent=rna-XM_044485699.1;Dbxref=GeneID:123062254,Genbank:XP_044341634.1;Name=XP_044341634.1;gbkey=CDS;gene=LOC123062254;product=external alternative NAD(P)H-ubiquinone oxidoreductase B3%2C mitochondrial-like;protein_id=XP_044341634.1

GO:0005743; Mitochondrial inner membrane

Kozak CAGATGC ID=cds-XP_044341709.1;Parent=rna-XM_044485774.1;Dbxref=GeneID:123062317,Genbank:XP_044341709.1;Name=XP_044341709.1;gbkey=CDS;gene=LOC123062317;product=DNA-(apurinic or apyrimidinic site) endonuclease%2C chloroplastic-like isoform X1;protein_id=XP_044341709.1

GO:0005634; Nucleus

Kozak: CCCATGT ID=cds-XP_044341949.1;Parent=rna-XM_044486014.1;Dbxref=GeneID:123062480,Genbank:XP_044341949.1;Name=XP_044341949.1;gbkey=CDS;gene=LOC123062480;product=uncharacterized protein At5g39865-like;protein_id=XP_044341949.1

GO:0097573; Glutathione oxidoreductase activity

Kozak: TACATGC ID=cds-XP_044341999.1;Parent=rna-XM_044486064.1;Dbxref=GeneID:123062516,Genbank:XP_044341999.1;Name=XP_044341999.1;gbkey=CDS;gene=LOC123062516;product=ABC transporter G family member 6-like;protein_id=XP_044341999.1

GO:0016021; Integral component of membrane

Kozak: TTCATGT ID=cds-XP_044342773.1;Parent=rna-XM_044486838.1;Dbxref=GeneID:123063100,Genbank:XP_044342773.1;Name=XP_044342773.1;gbkey=CDS;gene=LOC123063100;product=

putative 2-hydroxyacid dehydrogenase HI_1556 isoform X1;protein

Kozak: TACATGC ID=cds-XP_044342818.1;Parent=rna-XM_044486883.1;Dbxref=GeneID:123063141,Genbank:XP_044342818.1;Name=XP_044342818.1;gbkey=CDS;gene=LOC123063141;product=G-type lectin S-receptor-like serine/threonine-protein kinase At2g19130;protein_id=XP_044342818.1

GO:0016021; Integral component of membrane

Kozak: CAGATGC ID=cds-XP_044342868.1;Parent=rna-XM_044486933.1;Dbxref=GeneID:123063198,Genbank:XP_044342868.1;Name=XP_044342868.1;gbkey=CDS;gene=LOC123063198;product=auxin response factor 4-like;protein_id=XP_044342868.1

GO:0005634; Nucleus

Kozak: TTCATGT ID=cds-XP_044341684.1;Parent=rna-XM_044485749.1;Dbxref=GeneID:100682465,Genbank:XP_044341684.1;Name=XP_044341684.1;gbkey=CDS;gene=LOC100682465;product=protein translation factor SUI1 homolog;protein_id=XP_044341684.1

GO:0003723; RNA binding

Kozak: TCCATGT ID=cds-XP_044335907.1;Parent=rna-XM_044479972.1;Dbxref=GeneID:123056650,Genbank:XP_044335907.1;Name=XP_044335907.1;gbkey=CDS;gene=LOC123056650;product=

F-box protein At5g62510-like;protein

Kozak: CCAATGT ID=cds-XP_044335930.1;Parent=rna-XM_044479995.1;Dbxref=GeneID:123056690,Genbank:XP_044335930.1;Name=XP_044335930.1;gbkey=CDS;gene=LOC123056690;product=BTB/POZ and MATH domain-containing protein 3-like;protein_id=XP_044335930.1

GO:0016567; Protein ubiquitination

Kozak: TCCATGT ID=cds-XP_044335976.1;Parent=rna-XM_044480041.1;Dbxref=GeneID:123056764,Genbank:XP_044335976.1;Name=XP_044335976.1;gbkey=CDS;gene=LOC123056764;product=uncharacterized protein LOC123056764;protein_id=XP_044335976.1

GO:0009507; Chloroplast

Kozak: CCAATGC ID=cds-XP_044336215.1;Parent=rna-XM_044480280.1;Dbxref=GeneID:123057171,Genbank:XP_044336215.1;Name=XP_044336215.1;gbkey=CDS;gene=LOC123057171;product=

zinc finger BED domain-containing protein RICESLEEPER 2-like;protein

Kozak: TACATGC ID=cds-XP_044336312.1;Parent=rna-XM_044480377.1;Dbxref=GeneID:123057358,Genbank:XP_044336312.1;Name=XP_044336312.1;gbkey=CDS;gene=LOC123057358;product=

zinc finger CCCH domain-containing protein 33-like;protein

Kozak: CAGATGC ID=cds-XP_044336525.1;Parent=rna-XM_044480590.1;Dbxref=GeneID:123057666,Genbank:XP_044336525.1;Name=XP_044336525.1;gbkey=CDS;gene=LOC123057666;product=putative receptor-like protein kinase At4g00960;protein_id=XP_044336525.1

GO:0005886; Plasma membrane

Kozak: CCCATGC ID=cds-XP_044336543.1;Parent=rna-XM_044480608.1;Dbxref=GeneID:123057690,Genbank:XP_044336543.1;Name=XP_044336543.1;gbkey=CDS;gene=LOC123057690;product=

E3 ubiquitin-protein ligase SINA-like 10 isoform X1;protein

Kozak: TACATGC ID=cds-XP_044336920.1;Parent=rna-XM_044480985.1;Dbxref=GeneID:123058170,Genbank:XP_044336920.1;Name=XP_044336920.1;gbkey=CDS;gene=LOC123058170;product=

probable nucleolar protein 5-1 isoform X3;protein

Kozak: TTCATGT ID=cds-XP_044337047.1;Parent=rna-XM_044481112.1;Dbxref=GeneID:123058378,Genbank:XP_044337047.1;Name=XP_044337047.1;gbkey=CDS;gene=LOC123058378;product=WRKY transcription factor 22-like;protein_id=XP_044337047.1

GO:0005634; Nucleus

Kozak: CCAATGC ID=cds-XP_044337121.1;Parent=rna-XM_044481186.1;Dbxref=GeneID:123058467,Genbank:XP_044337121.1;Name=XP_044337121.1;gbkey=CDS;gene=LOC123058467;product=E3 ubiquitin-protein ligase At4g11680-like;protein_id=XP_044337121.1

GO:0016021; Integral component of membrane

Kozak: CCAATGT ID=cds-XP_044337153.1;Parent=rna-XM_044481218.1;Dbxref=GeneID:123058502,Genbank:XP_044337153.1;Name=XP_044337153.1;gbkey=CDS;gene=LOC123058502;product=probable purine permease 4;protein_id=XP_044337153.1

GO:0016021; Integral component of membrane

Kozak: CCCATGC ID=cds-XP_044337413.1;Parent=rna-XM_044481478.1;Dbxref=GeneID:123058803,Genbank:XP_044337413.1;Name=XP_044337413.1;gbkey=CDS;gene=LOC123058803;product=cis-prenyltransferase 4%2C chloroplastic-like;protein_id=XP_044337413.1

GO:0005783; Endoplasmic reticulum

Kozak: CAGATGC ID=cds-XP_044337874.1;Parent=rna-XM_044481939.1;Dbxref=GeneID:123059360,Genbank:XP_044337874.1;Name=XP_044337874.1;gbkey=CDS;gene=LOC123059360;product=probable inactive purple acid phosphatase 16;protein_id=XP_044337874.1

GO:0016788; Hydrolase activity, acting on ester bonds

Kozak: CAGATGC ID=cds-XP_044337944.1;Parent=rna-XM_044482009.1;Dbxref=GeneID:123059434,Genbank:XP_044337944.1;Name=XP_044337944.1;gbkey=CDS;gene=LOC123059434;product=uncharacterized protein LOC123059434;protein_id=XP_044337944.1

GO:0016021; Integral component of membrane

Kozak: CCCATGT ID=cds-XP_044338133.1;Parent=rna-XM_044482198.1;Dbxref=GeneID:123059699,Genbank:XP_044338133.1;Name=XP_044338133.1;gbkey=CDS;gene=LOC123059699;product=uncharacterized protein LOC123059699;protein_id=XP_044338133.1

GO:0005739; Mitochondrion

Kozak: TTAATGC ID=cds-XP_044338153.1;Parent=rna-XM_044482218.1;Dbxref=GeneID:123059733,Genbank:XP_044338153.1;Name=XP_044338153.1;gbkey=CDS;gene=LOC123059733;product=uncharacterized protein LOC123059733;protein_id=XP_044338153.1

GO:0016021; Integral component of membrane

Kozak: CAGATGC ID=cds-XP_044338207.1;Parent=rna-XM_044482272.1;Dbxref=GeneID:123059786,Genbank:XP_044338207.1;Name=XP_044338207.1;gbkey=CDS;gene=LOC123059786;product=dephospho-CoA kinase-like;protein_id=XP_044338207.1

GO:0005524; ATP binding

Kozak: CCCATGT ID=cds-XP_044338937.1;Parent=rna-XM_044483002.1;Dbxref=GeneID:123060329,Genbank:XP_044338937.1;Name=XP_044338937.1;gbkey=CDS;gene=LOC123060329;product=transcription initiation factor TFIID subunit 15b;protein_id=XP_044338937.1

GO:0046872; Metal ion binding

Kozak: CCCATGC ID=cds-XP_044339121.1;Parent=rna-XM_044483186.1;Dbxref=GeneID:123060458,Genbank:XP_044339121.1;Name=XP_044339121.1;gbkey=CDS;gene=LOC123060458;product=uncharacterized protein LOC123060458 isoform X1;protein_id=XP_044339121.1

GO:0006357; Reregulation of transcription by RNA polymerase II

Kozak: TTCATGT ID=cds-XP_044339993.1;Parent=rna-XM_044484058.1;Dbxref=GeneID:123061119,Genbank:XP_044339993.1;Name=XP_044339993.1;gbkey=CDS;gene=LOC123061119;product=exportin-7-A-like isoform X1;protein_id=XP_044339993.1

GO:0005737; Cytoplasm

Kozak: TCCATGT ID=cds-XP_044340351.1;Parent=rna-XM_044484416.1;Dbxref=GeneID:123061344,Genbank:XP_044340351.1;Name=XP_044340351.1;gbkey=CDS;gene=LOC123061344;product=protein CONSERVED IN THE GREEN LINEAGE AND DIATOMS 27%2C chloroplastic-like isoform X1;protein_id=XP_044340351.1

GO:0016021; Integral component of membrane

Kozak: CCCATGC ID=cds-XP_044340692.1;Parent=rna-XM_044484757.1;Dbxref=GeneID:123061592,Genbank:XP_044340692.1;Name=XP_044340692.1;gbkey=CDS;gene=LOC123061592;product=DELLA protein GAI-like;protein_id=XP_044340692.1

GO:0005634; Nucleus

Kozak: CCCATGC ID=cds-XP_044340902.1;Parent=rna-XM_044484967.1;Dbxref=GeneID:123061732,Genbank:XP_044340902.1;Name=XP_044340902.1;gbkey=CDS;gene=LOC123061732;product=B3 domain-containing protein LFL1-like isoform X1;protein_id=XP_044340902.1

GO:0005634; Nucleus

Kozak: CCAATGT ID=cds-XP_044341115.1;Parent=rna-XM_044485180.1;Dbxref=GeneID:123061897,Genbank:XP_044341115.1;Name=XP_044341115.1;gbkey=CDS;gene=LOC123061897;product=auxin-responsive protein IAA5-like;protein_id=XP_044341115.1

GO:0005634; Nucleus

Kozak: TCCATGT ID=cds-XP_044341198.1;Parent=rna-XM_044485263.1;Dbxref=GeneID:123061971,Genbank:XP_044341198.1;Name=XP_044341198.1;gbkey=CDS;gene=LOC123061971;product=

serine/threonine-protein phosphatase 7 long form homolog;protein

Kozak: CCCATGT ID=cds-XP_044341845.1;Parent=rna-XM_044485910.1;Dbxref=GeneID:123062414,Genbank:XP_044341845.1;Name=XP_044341845.1;gbkey=CDS;gene=LOC123062414;product=calcium uniporter protein 6%2C mitochondrial-like;protein_id=XP_044341845.1

GO:0031305; Integral component of mitochondrial inner membrane

Kozak: CCAATGC ID=cds-XP_044342117.1;Parent=rna-XM_044486182.1;Dbxref=GeneID:123062603,Genbank:XP_044342117.1;Name=XP_044342117.1;gbkey=CDS;gene=LOC123062603;product=uncharacterized protein LOC123062603 isoform X1;protein_id=XP_044342117.1

GO:0005739; Mitochondrion

Kozak: CCCATGT ID=cds-XP_044342364.1;Parent=rna-XM_044486429.1;Dbxref=GeneID:123062773,Genbank:XP_044342364.1;Name=XP_044342364.1;gbkey=CDS;gene=LOC123062773;product=ubiquitin-like domain-containing CTD phosphatase;protein_id=XP_044342364.1

GO:0046658; Anchored component of plasma membrane

Kozak: TACATGC ID=cds-XP_044342901.1;Parent=rna-XM_044486966.1;Dbxref=GeneID:123063224,Genbank:XP_044342901.1;Name=XP_044342901.1;gbkey=CDS;gene=LOC123063224;product=probable LRR receptor-like serine/threonine-protein kinase At3g47570;protein_id=XP_044342901.1

GO:0016021; Integral component of membrane

Kozak: CAGATGC ID=cds-XP_044343202.1;Parent=rna-XM_044487267.1;Dbxref=GeneID:123063475,Genbank:XP_044343202.1;Name=XP_044343202.1;gbkey=CDS;gene=LOC123063475;product=protein LATERAL ROOT PRIMORDIUM 1-like;protein_id=XP_044343202.1

GO:0005634; Nucleus

Kozak: CCAATGC ID=cds-XP_044328540.1;Parent=rna-XM_044472605.1;Dbxref=GeneID:123049718,Genbank:XP_044328540.1;Name=XP_044328540.1;gbkey=CDS;gene=LOC123049718;product=xyloglucan galactosyltransferase XLT2-like;protein_id=XP_044328540.1

GO:0005794; Golgi apparatus

Kozak: TACATGC ID=cds-XP_044329505.1;Parent=rna-XM_044473570.1;Dbxref=GeneID:123050838,Genbank:XP_044329505.1;Name=XP_044329505.1;gbkey=CDS;gene=LOC123050838;product=NAC domain-containing protein 30-like;protein_id=XP_044329505.1

GO:0005634; Nucleus

Kozak: TTCATGT ID=cds-XP_044329650.1;Parent=rna-XM_044473715.1;Dbxref=GeneID:123050990,Genbank:XP_044329650.1;Name=XP_044329650.1;gbkey=CDS;gene=LOC123050990;product=receptor kinase-like protein Xa21;protein_id=XP_044329650.1

GO:0016021; Integral component of membrane

Kozak: TCCATGT ID=cds-XP_044329840.1;Parent=rna-XM_044473905.1;Dbxref=GeneID:123051119,Genbank:XP_044329840.1;Name=XP_044329840.1;gbkey=CDS;gene=LOC123051119;product=cytosolic sulfotransferase 5-like;protein_id=XP_044329840.1

GO:0005737; Cytoplasm

Kozak: CCCATGT ID=cds-XP_044329933.1;Parent=rna-XM_044473998.1;Dbxref=GeneID:123051191,Genbank:XP_044329933.1;Name=XP_044329933.1;gbkey=CDS;gene=LOC123051191;product=peptidyl-prolyl cis-trans isomerase FKBP19%2C chloroplastic-like isoform X1;protein_id=XP_044329933.1

GO:0003755; Peptidyl-prolyl cis-trans isomerase activity

Kozak: CCCATGT ID=cds-XP_044330220.1;Parent=rna-

XM_044474285.1;Dbxref=GeneID:123051418,Genbank:XP_044330220.1;Name=XP_044330220.1;gbkey=CDS;gene=LOC123051418;product=receptor-like protein kinase;protein_id=XP_044330220.1

GO:0016021; Integral component of membrane

Kozak: CCCATGT ID=cds-XP_044330330.1;Parent=rna-XM_044474395.1;Dbxref=GeneID:123051503,Genbank:XP_044330330.1;Name=XP_044330330.1;gbkey=CDS;gene=LOC123051503;product=RINT1-like protein MAG2L;protein_id=XP_044330330.1

GO:0070939; Dsl1/NZR complex

Kozak: CCAATGC ID=cds-XP_044331210.1;Parent=rna-XM_044475275.1;Dbxref=GeneID:123052168,Genbank:XP_044331210.1;Name=XP_044331210.1;gbkey=CDS;gene=LOC123052168;product=uncharacterized hydrolase YugF-like;protein_id=XP_044331210.1

GO:0003824; Catalytic activity

Kozak: CGAATGT ID=cds-XP_044331266.1;Parent=rna-XM_044475331.1;Dbxref=GeneID:123052215,Genbank:XP_044331266.1;Name=XP_044331266.1;gbkey=CDS;gene=LOC123052215;product=zinc finger SWIM domain-containing protein 7-like;protein_id=XP_044331266.1

GO:0097196; Shu complex

Kozak: CCAATGC ID=cds-XP_044331633.1;Parent=rna-XM_044475698.1;Dbxref=GeneID:123052502,Genbank:XP_044331633.1;Name=XP_044331633.1;gbkey=CDS;gene=LOC123052502;product=

cysteine-rich receptor-like protein kinase 6 isoform X1;protein

Kozak: TCCATGT ID=cds-XP_044331903.1;Parent=rna-XM_044475968.1;Dbxref=GeneID:123052664,Genbank:XP_044331903.1;Name=XP_044331903.1;gbkey=CDS;gene=LOC123052664;product=ATP-dependent DNA helicase SRS2-like protein At4g25120;protein_id=XP_044331903.1

GO:0005634; Nucleus

Kozak: CCCATGT ID=cds-XP_044332573.1;Parent=rna-XM_044476638.1;Dbxref=GeneID:123053205,Genbank:XP_044332573.1;Name=XP_044332573.1;gbkey=CDS;gene=LOC123053205;product=uncharacterized protein At1g32220%2C chloroplastic-like;protein_id=XP_044332573.1

GO:0006952; Defense response

Kozak: CCCATGT ID=cds-XP_044332599.1;Parent=rna-XM_044476664.1;Dbxref=GeneID:123053223,Genbank:XP_044332599.1;Name=XP_044332599.1;gbkey=CDS;gene=LOC123053223;product=cytochrome P450 77A4-like;protein_id=XP_044332599.1

GO:0016021; Integral component of membrane

Kozak: CCAATGC ID=cds-XP_044332742.1;Parent=rna-XM_044476807.1;Dbxref=GeneID:123053345,Genbank:XP_044332742.1;Name=XP_044332742.1;gbkey=CDS;gene=LOC123053345;product=embryonic protein DC-8-like;protein_id=XP_044332742.1

GO:0016021; Integral component of membrane

Kozak: CCAATGT ID=cds-XP_044332775.1;Parent=rna-XM_044476840.1;Dbxref=GeneID:123053375,Genbank:XP_044332775.1;Name=XP_044332775.1;gbkey=CDS;gene=LOC123053375;product=serine/threonine-protein kinase-like protein CCR4;protein_id=XP_044332775.1

GO:0016021; Integral component of membrane

Kozak: CCCATGT ID=cds-XP_044333094.1;Parent=rna-XM_044477159.1;Dbxref=GeneID:123053649,Genbank:XP_044333094.1;Name=XP_044333094.1;gbkey=CDS;gene=LOC123053649;product=SWI/SNF complex subunit SWI3A homolog;protein_id=XP_044333094.1

GO:0043229; Intracellular organelle

Kozak: CAGATGC ID=cds-XP_044333543.1;Parent=rna-XM_044477608.1;Dbxref=GeneID:123053979,Genbank:XP_044333543.1;Name=XP_044333543.1;gbkey=CDS;gene=LOC123053979;product=pentatricopeptide repeat-containing protein At3g53360%2C mitochondrial-like;protein_id=XP_044333543.1

GO:0003723; RNA binding

Kozak: CCCATGC ID=cds-XP_044333761.1;Parent=rna-XM_044477826.1;Dbxref=GeneID:123054132,Genbank:XP_044333761.1;Name=XP_044333761.1;Note=The sequence of the model RefSeq protein was modified relative to this genomic sequence to represent the inferred CDS: added 87 bases not found in genome assembly;exception=annotated by transcript or proteomic data;gbkey=CDS;gene=LOC123054132;inference=similar to RNA sequence (same species):INSD:GFFI01065727.1;partial=true;product=uncharacterized protein LOC123054132;protein_id=XP_044333761.1

GO:0016020; Membrane

Kozak: CCCATGT ID=cds-XP_044333798.1;Parent=rna-XM_044477863.1;Dbxref=GeneID:123054161,Genbank:XP_044333798.1;Name=XP_044333798.1;gbkey=CDS;gene=LOC123054161;product=uncharacterized protein LOC123054161;protein_id=XP_044333798.1

GO:0005730; Nucleolus

Kozak: CAGATGC ID=cds-XP_044333834.1;Parent=rna-XM_044477899.1;Dbxref=GeneID:123054187,Genbank:XP_044333834.1;Name=XP_044333834.1;gbkey=CDS;gene=LOC123054187;product=lysine-specific histone demethylase 1 homolog 3;protein_id=XP_044333834.1

GO:0110165; Cellular anatomical entity

Kozak: CCCATGT ID=cds-XP_044334110.1;Parent=rna-XM_044478175.1;Dbxref=GeneID:123054404,Genbank:XP_044334110.1;Name=XP_044334110.1;gbkey=CDS;gene=LOC123054404;product=maltose excess protein 1-like%2C chloroplastic;protein_id=XP_044334110.1

GO:0009941; Chloroplast envelope

Kozak: CAGATGC ID=cds-XP_044335657.1;Parent=rna-XM_044479722.1;Dbxref=GeneID:123055910,Genbank:XP_044335657.1;Name=XP_044335657.1;gbkey=CDS;gene=LOC123055910;product=pathogenesis-related protein PRMS-like;protein_id=XP_044335657.1

GO:0005615; Extracellular space

Kozak: TTAATGC ID=cds-XP_044335664.1;Parent=rna-XM_044479729.1;Dbxref=GeneID:123055920,Genbank:XP_044335664.1;Name=XP_044335664.1;gbkey=CDS;gene=LOC123055920;product=

ubiquinol oxidase 1b%2C mitochondrial-like;protein

Kozak: CCAATGT ID=cds-XP_044334994.1;Parent=rna-XM_044479059.1;Dbxref=GeneID:778420,Genbank:XP_044334994.1;Name=XP_044334994.1;gbkey=CDS;gene=LOC778420;product=probable phytol kinase%2C chloroplastic isoform X1;protein_id=XP_044334994.1

GO:0031969; Chloroplast membrane

Kozak: CCCATGT ID=cds-XP_044333990.1;Parent=rna-XM_044478055.1;Dbxref=GeneID:100873134,Genbank:XP_044333990.1;Name=XP_044333990.1;gbkey=CDS;gene=LOC100873134;product=ent-kaur-16-ene synthase%2C chloroplastic isoform X1;protein_id=XP_044333990.1

GO:0000287; Magnesium ion binding

Kozak: TCCATGT ID=cds-XP_044327391.1;Parent=rna-XM_044471456.1;Dbxref=GeneID:123048328,Genbank:XP_044327391.1;Name=XP_044327391.1;gbkey=CDS;gene=LOC123048328;product=

wall-associated receptor kinase 2-like;protein

Kozak: TGTATGT ID=cds-XP_044327540.1;Parent=rna-XM_044471605.1;Dbxref=GeneID:123048520,Genbank:XP_044327540.1;Name=XP_044327540.1;gbkey=CDS;gene=LOC123048520;product=

F-box/LRR-repeat protein At4g14103-like;protein

Kozak: TACATGC ID=cds-XP_044327612.1;Parent=rna-XM_044471677.1;Dbxref=GeneID:123048606,Genbank:XP_044327612.1;Name=XP_044327612.1;gbkey=CDS;gene=LOC123048606;product=

probable serine/threonine-protein kinase PBL4;protein

Kozak: CAGATGC ID=cds-XP_044327944.1;Parent=rna-XM_044472009.1;Dbxref=GeneID:123048999,Genbank:XP_044327944.1;Name=XP_044327944.1;gbkey=CDS;gene=LOC123048999;product=probable inactive purple acid phosphatase 16;protein_id=XP_044327944.1

GO:0016788; Hydrolase activity, acting on ester bonds

Kozak: TTAATGC ID=cds-XP_044328201.1;Parent=rna-XM_044472266.1;Dbxref=GeneID:123049334,Genbank:XP_044328201.1;Name=XP_044328201.1;gbkey=CDS;gene=LOC123049334;product=uncharacterized protein LOC123049334;protein_id=XP_044328201.1

GO:0016021; C:integral component of membrane

Kozak: CAGATGC ID=cds-XP_044328337.1;Parent=rna-XM_044472402.1;Dbxref=GeneID:123049496,Genbank:XP_044328337.1;Name=XP_044328337.1;gbkey=CDS;gene=LOC123049496;product=transcription factor MYB80-like;protein_id=XP_044328337.1

GO:0003677; DNA binding

Kozak: CCCATGC ID=cds-XP_044328530.1;Parent=rna-XM_044472595.1;Dbxref=GeneID:123049710,Genbank:XP_044328530.1;Name=XP_044328530.1;gbkey=CDS;gene=LOC123049710;product=uncharacterized protein LOC123049710;protein_id=XP_044328530.1

GO:0016021; Integral component of membrane

Kozak: TCCATGT ID=cds-XP_044328660.1;Parent=rna-XM_044472725.1;Dbxref=GeneID:123049859,Genbank:XP_044328660.1;Name=XP_044328660.1;gbkey=CDS;gene=LOC123049859;product=

late embryogenesis abundant protein At3g53040-like;protein

Kozak: CCCATGT ID=cds-XP_044328806.1;Parent=rna-XM_044472871.1;Dbxref=GeneID:123050023,Genbank:XP_044328806.1;Name=XP_044328806.1;gbkey=CDS;gene=LOC123050023;product=probable L-type lectin-domain containing receptor kinase S.5;protein_id=XP_044328806.1

GO:0016021; Integral component of membrane

Kozak: TACATGC ID=cds-XP_044329085.1;Parent=rna-XM_044473150.1;Dbxref=GeneID:123050337,Genbank:XP_044329085.1;Name=XP_044329085.1;gbkey=CDS;gene=LOC123050337;product=spermidine hydroxycinnamoyltransferase 1-like;protein_id=XP_044329085.1

GO:0016747; Acyltransferase activity

Kozak: TACATGC ID=cds-XP_044329086.1;Parent=rna-XM_044473151.1;Dbxref=GeneID:123050339,Genbank:XP_044329086.1;Name=XP_044329086.1;gbkey=CDS;gene=LOC123050339;product=

pterocarpan synthase 1-like;protein

Kozak: CCCATGT ID=cds-XP_044329202.1;Parent=rna-XM_044473267.1;Dbxref=GeneID:123050479,Genbank:XP_044329202.1;Name=XP_044329202.1;gbkey=CDS;gene=LOC123050479;product=metalloendoproteinase 1-MMP-like;protein_id=XP_044329202.1

GO:0031225; Anchored component of membrane

Kozak: CAGATGC ID=cds-XP_044329919.1;Parent=rna-XM_044473984.1;Dbxref=GeneID:123051179,Genbank:XP_044329919.1;Name=XP_044329919.1;gbkey=CDS;gene=LOC123051179;product=

putative F-box protein At1g12855;protein

Kozak: TTAATGC ID=cds-XP_044329923.1;Parent=rna-XM_044473988.1;Dbxref=GeneID:123051184,Genbank:XP_044329923.1;Name=XP_044329923.1;gbkey=CDS;gene=LOC123051184;product=DIBOA-glucoside dioxygenase BX6-like;protein_id=XP_044329923.1

GO:0051213; Dioxygenase activity

Kozak: CCCATGT ID=cds-XP_044330472.1;Parent=rna-XM_044474537.1;Dbxref=GeneID:123051603,Genbank:XP_044330472.1;Name=XP_044330472.1;gbkey=CDS;gene=LOC123051603;product=putative ubiquitin-like-specific protease 1B;protein_id=XP_044330472.1

GO:0005634; Nucleus

Kozak: CCAATGC ID=cds-XP_044330644.1;Parent=rna-XM_044474709.1;Dbxref=GeneID:123051742,Genbank:XP_044330644.1;Name=XP_044330644.1;gbkey=CDS;gene=LOC123051742;product=phosphatidylinositol 4-phosphate 5-kinase 6-like;protein_id=XP_044330644.1

GO:0005886; Plasma membrane

Kozak: CCAATGC ID=cds-XP_044330889.1;Parent=rna-XM_044474954.1;Dbxref=GeneID:123051940,Genbank:XP_044330889.1;Name=XP_044330889.1;gbkey=CDS;gene=LOC123051940;product=probable transcription factor At5g28040;protein_id=XP_044330889.1

GO:0005634; Nucleus

Kozak: CCCATGT ID=cds-XP_044331554.1;Parent=rna-XM_044475619.1;Dbxref=GeneID:123052441,Genbank:XP_044331554.1;Name=XP_044331554.1;gbkey=CDS;gene=LOC123052441;product=

probable mixed-linked glucan synthase 8 isoform X2;protein_id=XP_044331554.1

GO:0016021; Integral component of membrane

Kozak: TTCATGT ID=cds-XP_044331665.1;Parent=rna-XM_044475730.1;Dbxref=GeneID:123052520,Genbank:XP_044331665.1;Name=XP_044331665.1;gbkey=CDS;gene=LOC123052520;product=protein translation factor SUI1 homolog;protein_id=XP_044331665.1

GO:0003723; RNA binding

Kozak: CCCATGC ID=cds-XP_044331785.1;Parent=rna-XM_044475850.1;Dbxref=GeneID:123052601,Genbank:XP_044331785.1;Name=XP_044331785.1;gbkey=CDS;gene=LOC123052601;product=uncharacterized protein LOC123052601;protein_id=XP_044331785.1

GO:0005794; Golgi apparatus

Kozak: CCCATGC ID=cds-XP_044332047.1;Parent=rna-XM_044476112.1;Dbxref=GeneID:123052772,Genbank:XP_044332047.1;Name=XP_044332047.1;gbkey=CDS;gene=LOC123052772;product=AT-rich interactive domain-containing protein 6-like;protein_id=XP_044332047.1

GO:0005634; Nucleus

Kozak: CCCATGC ID=cds-XP_044332186.1;Parent=rna-XM_044476251.1;Dbxref=GeneID:123052890,Genbank:XP_044332186.1;Name=XP_044332186.1;gbkey=CDS;gene=LOC123052890;product=probable LRR receptor-like serine/threonine-protein kinase At2g16250;protein_id=XP_044332186.1

GO:0016021; Integral component of membrane

Kozak: CGAATGT ID=cds-XP_044332437.1;Parent=rna-XM_044476502.1;Dbxref=GeneID:123053103,Genbank:XP_044332437.1;Name=XP_044332437.1;gbkey=CDS;gene=LOC123053103;product=uncharacterized protein LOC123053103;protein_id=XP_044332437.1

GO:0016021; Integral component of membrane

Kozak: CCCATGT ID=cds-XP_044332702.1;Parent=rna-XM_044476767.1;Dbxref=GeneID:123053308,Genbank:XP_044332702.1;Name=XP_044332702.1;Note=The sequence of the model RefSeq protein was modified relative to this genomic sequence to represent the inferred CDS: added 16 bases not found in genome assembly;exception=annotated by transcript or proteomic data;gbkey=CDS;gene=LOC123053308;inference=similar to RNA sequence (same species):INSD:GDTJ01000728.1;partial=true;product=COP9 signalosome complex subunit 8-like;protein_id=XP_044332702.1;start_range=.,384735240

GO:0008180; Signalosome

Kozak: CCAATGC ID=cds-XP_044332938.1;Parent=rna-XM_044477003.1;Dbxref=GeneID:123053539,Genbank:XP_044332938.1;Name=XP_044332938.1;gbkey=CDS;gene=LOC123053539;product=

pentatricopeptide repeat-containing protein At4g26680%2C mitochondrial-like isoform

Kozak: CCAATGC ID=cds-XP_044333369.1;Parent=rna-XM_044477434.1;Dbxref=GeneID:123053851,Genbank:XP_044333369.1;Name=XP_044333369.1;gbkey=CDS;gene=LOC123053851;product=DNA mismatch repair protein MSH1%2C mitochondrial-like isoform X3;protein_id=XP_044333369.1

GO:0005739; Mitochondrion

Kozak: TCCATGT ID=cds-XP_044333508.1;Parent=rna-XM_044477573.1;Dbxref=GeneID:123053943,Genbank:XP_044333508.1;Name=XP_044333508.1;gbkey=CDS;gene=LOC123053943;product=cytosolic sulfotransferase 8-like;protein_id=XP_044333508.1

GO:0005737; Cytoplasm

Kozak: CAGATGC ID=cds-XP_044334353.1;Parent=rna-XM_044478418.1;Dbxref=GeneID:123054612,Genbank:XP_044334353.1;Name=XP_044334353.1;gbkey=CDS;gene=LOC123054612;product=

glycine-rich cell wall structural protein-like;protein

Kozak: TCCATGT ID=cds-XP_044334761.1;Parent=rna-XM_044478826.1;Dbxref=GeneID:123054948,Genbank:XP_044334761.1;Name=XP_044334761.1;gbkey=CDS;gene=LOC123054948;product=putative chloride channel-like protein CLC-g isoform X1;protein_id=XP_044334761.1

GO:0016021; Integral component of membrane

Kozak: CCAATGT ID=cds-XP_044334947.1;Parent=rna-XM_044479012.1;Dbxref=GeneID:123055093,Genbank:XP_044334947.1;Name=XP_044334947.1;gbkey=CDS;gene=LOC123055093;product=probable inactive purple acid phosphatase 2;protein_id=XP_044334947.1

GO:0016021; Integral component of membrane

Kozak: CAGATGC ID=cds-XP_044395123.1;Parent=rna-XM_044539188.1;Dbxref=GeneID:123119392,Genbank:XP_044395123.1;Name=XP_044395123.1;gbkey=CDS;gene=LOC123119392;product=

pentatricopeptide repeat-containing protein At4g18520%2C chloroplastic-like;protein

Kozak: CCCATGC ID=cds-XP_044395262.1;Parent=rna-XM_044539327.1;Dbxref=GeneID:123119507,Genbank:XP_044395262.1;Name=XP_044395262.1;gbkey=CDS;gene=LOC123119507;product=gamma-tubulin complex component 5-like isoform X1;protein_id=XP_044395262.1

GO:0005737; Cytoplasm

Kozak: CCAATGT ID=cds-XP_044395449.1;Parent=rna-XM_044539514.1;Dbxref=GeneID:123119650,Genbank:XP_044395449.1;Name=XP_044395449.1;gbkey=CDS;gene=LOC123119650;product=pentatricopeptide repeat-containing protein At3g29230-like;protein_id=XP_044395449.1

GO:0005737; Cytoplasm

Kozak: TGTATGT ID=cds-XP_044395640.1;Parent=rna-XM_044539705.1;Dbxref=GeneID:123119781,Genbank:XP_044395640.1;Name=XP_044395640.1;gbkey=CDS;gene=LOC123119781;product=uncharacterized protein LOC123119781;protein_id=XP_044395640.1

GO:0001709; Cell fate determination

Kozak: TCCATGT ID=cds-XP_044395892.1;Parent=rna-XM_044539957.1;Dbxref=GeneID:123119970,Genbank:XP_044395892.1;Name=XP_044395892.1;gbkey=CDS;gene=LOC123119970;product=

protein transport protein Sec24-like CEF isoform X1;protein

Kozak: CAGATGC ID=cds-XP_044396031.1;Parent=rna-XM_044540096.1;Dbxref=GeneID:123120087,Genbank:XP_044396031.1;Name=XP_044396031.1;gbkey=CDS;gene=LOC123120087;product=alpha-(1%2C4)-fucosyltransferase-like;protein_id=XP_044396031.1

GO:0032580; Golgi cisterna membrane

Kozak: TTCATGT ID=cds-XP_044396141.1;Parent=rna-XM_044540206.1;Dbxref=GeneID:123120215,Genbank:XP_044396141.1;Name=XP_044396141.1;gbkey=CDS;gene=LOC123120215;product=purple acid phosphatase 22-like;protein_id=XP_044396141.1

GO:0003993; Acid phosphatase activity

Kozak: TTCATGT ID=cds-XP_044396200.1;Parent=rna-XM_044540265.1;Dbxref=GeneID:123120296,Genbank:XP_044396200.1;Name=XP_044396200.1;gbkey=CDS;gene=LOC123120296;product=RING finger protein 10-like;protein_id=XP_044396200.1

GO:0005783; Endoplasmic reticulum

Kozak: CAGATGC ID=cds-XP_044396284.1;Parent=rna-XM_044540349.1;Dbxref=GeneID:123120378,Genbank:XP_044396284.1;Name=XP_044396284.1;gbkey=CDS;gene=LOC123120378;product=2-hydroxy-6-oxononadienedioate/2-hydroxy-6-oxononatrienedioate hydrolase 1-like;protein_id=XP_044396284.1

GO:0016021; Integral component of membrane

Kozak: CCCATGT ID=cds-XP_044396289.1;Parent=rna-XM_044540354.1;Dbxref=GeneID:123120381,Genbank:XP_044396289.1;Name=XP_044396289.1;gbkey=CDS;gene=LOC123120381;product=uncharacterized protein LOC123120381 isoform X1;protein_id=XP_044396289.1

GO:0016021; Integral component of membrane

Kozak: TTCATGT ID=cds-XP_044396405.1;Parent=rna-XM_044540470.1;Dbxref=GeneID:123120468,Genbank:XP_044396405.1;Name=XP_044396405.1;gbkey=CDS;gene=LOC123120468;product=putative pentatricopeptide repeat-containing protein At3g08820;protein_id=XP_044396405.1

GO:0008270; Zinc ion binding

Kozak: CGAATGT ID=cds-XP_044396764.1;Parent=rna-XM_044540829.1;Dbxref=GeneID:123120842,Genbank:XP_044396764.1;Name=XP_044396764.1;gbkey=CDS;gene=LOC123120842;product=

probable calcium-binding protein CML21;protein

Kozak: TACATGC ID=cds-XP_044397296.1;Parent=rna-XM_044541361.1;Dbxref=GeneID:123121401,Genbank:XP_044397296.1;Name=XP_044397296.1;gbkey=CDS;gene=LOC123121401;product=protein STRICTOSIDINE SYNTHASE-LIKE 10-like;protein_id=XP_044397296.1

GO:0005773; Vacuole

Kozak: CCCATGC ID=cds-XP_044397628.1;Parent=rna-XM_044541693.1;Dbxref=GeneID:123121679,Genbank:XP_044397628.1;Name=XP_044397628.1;gbkey=CDS;gene=LOC123121679;product=

pentatricopeptide repeat-containing protein At5g61400-like;protein

Kozak: CCCATGT ID=cds-XP_044397763.1;Parent=rna-XM_044541828.1;Dbxref=GeneID:123121782,Genbank:XP_044397763.1;Name=XP_044397763.1;gbkey=CDS;gene=LOC123121782;product=phosphoribosylaminoimidazole-succinocarboxamide synthase%2C chloroplastic-like;protein_id=XP_044397763.1

GO:0005524; ATP binding

Kozak: TTAATGC ID=cds-XP_044397779.1;Parent=rna-XM_044541844.1;Dbxref=GeneID:123121790,Genbank:XP_044397779.1;Name=XP_044397779.1;gbkey=CDS;gene=LOC123121790;product=uncharacterized protein LOC123121790;protein_id=XP_044397779.1

GO:0016021; Integral component of membrane

Kozak: TTCATGT ID=cds-XP_044397839.1;Parent=rna-XM_044541904.1;Dbxref=GeneID:123121836,Genbank:XP_044397839.1;Name=XP_044397839.1;gbkey=CDS;gene=LOC123121836;product=serine/threonine-protein kinase D6PK-like;protein_id=XP_044397839.1

GO:0005737; Cytoplasm

Kozak: CCAATGC ID=cds-XP_044397858.1;Parent=rna-XM_044541923.1;Dbxref=GeneID:123121847,Genbank:XP_044397858.1;Name=XP_044397858.1;gbkey=CDS;gene=LOC123121847;product=leucine-rich repeat receptor-like serine/threonine-protein kinase RGI4;protein_id=XP_044397858.1

GO:0016021; Integral component of membrane

Kozak: CAGATGC ID=cds-XP_044397948.1;Parent=rna-XM_044542013.1;Dbxref=GeneID:123121920,Genbank:XP_044397948.1;Name=XP_044397948.1;gbkey=CDS;gene=LOC123121920;product=

OVARIAN TUMOR DOMAIN-containing deubiquitinating enzyme 4-like;protein

Kozak: CAGATGC ID=cds-XP_044398174.1;Parent=rna-XM_044542239.1;Dbxref=GeneID:123122101,Genbank:XP_044398174.1;Name=XP_044398174.1;gbkey=CDS;gene=LOC123122101;product=probable UDP-arabinose 4-epimerase 1;protein_id=XP_044398174.1

GO:0003978; UDP-glucose 4-epimerase activity

Kozak: CCCATGC ID=cds-XP_044398190.1;Parent=rna-XM_044542255.1;Dbxref=GeneID:123122112,Genbank:XP_044398190.1;Name=XP_044398190.1;gbkey=CDS;gene=LOC123122112;product=

caffeoylshikimate esterase-like;protein

Kozak: TTCATGT ID=cds-XP_044398333.1;Parent=rna-XM_044542398.1;Dbxref=GeneID:123122245,Genbank:XP_044398333.1;Name=XP_044398333.1;gbkey=CDS;gene=LOC123122245;product=glucuronoxylan 4-O-methyltransferase 1-like;protein_id=XP_044398333.1

GO:0005794; Golgi apparatus

Kozak: CCAATGC ID=cds-XP_044398511.1;Parent=rna-XM_044542576.1;Dbxref=GeneID:123122384,Genbank:XP_044398511.1;Name=XP_044398511.1;gbkey=CDS;gene=LOC123122384;product=

protein POLLENLESS 3-LIKE 2-like isoform X1;protein

Kozak: CAGATGC ID=cds-XP_044398976.1;Parent=rna-XM_044543041.1;Dbxref=GeneID:123122716,Genbank:XP_044398976.1;Name=XP_044398976.1;gbkey=CDS;gene=LOC123122716;product=9-cis-epoxycarotenoid dioxygenase NCED3%2C chloroplastic-like;protein_id=XP_044398976.1

GO:0009570; Chloroplast stroma

Kozak: CAGATGC ID=cds-XP_044399156.1;Parent=rna-XM_044543221.1;Dbxref=GeneID:123122858,Genbank:XP_044399156.1;Name=XP_044399156.1;gbkey=CDS;gene=LOC123122858;product=protein PYRICULARIA ORYZAE RESISTANCE 21-like;protein_id=XP_044399156.1

GO:0046872; Metal ion binding

Kozak: CCAATGC ID=cds-XP_044399312.1;Parent=rna-XM_044543377.1;Dbxref=GeneID:123122986,Genbank:XP_044399312.1;Name=XP_044399312.1;gbkey=CDS;gene=LOC123122986;product=leucine-rich repeat receptor-like serine/threonine-protein kinase BAM1;protein_id=XP_044399312.1

GO:0016021; Integral component of membrane

Kozak: TCCATGT ID=cds-XP_044399477.1;Parent=rna-XM_044543542.1;Dbxref=GeneID:123123098,Genbank:XP_044399477.1;Name=XP_044399477.1;gbkey=CDS;gene=LOC123123098;product=zinc finger CCCH domain-containing protein 16-like;protein_id=XP_044399477.1

GO:0005681; Spliceosome complex

Kozak: TTCATGT ID=cds-XP_044399493.1;Parent=rna-XM_044543558.1;Dbxref=GeneID:123123113,Genbank:XP_044399493.1;Name=XP_044399493.1;gbkey=CDS;gene=LOC123123113;product=cullin-4-like;protein_id=XP_044399493.1

GO:0031461; Cullin-RING ubiquitin ligase complex

Kozak: CCCATGT ID=cds-XP_044399658.1;Parent=rna-XM_044543723.1;Dbxref=GeneID:123123246,Genbank:XP_044399658.1;Name=XP_044399658.1;gbkey=CDS;gene=LOC123123246;product=chromatin remodeling protein EBS-like isoform X2;protein_id=XP_044399658.1

GO:0003682; Chromatin binding

Kozak: CAGATGC ID=cds-XP_044399683.1;Parent=rna-XM_044543748.1;Dbxref=GeneID:123123269,Genbank:XP_044399683.1;Name=XP_044399683.1;gbkey=CDS;gene=LOC123123269;product=LEAF RUST 10 DISEASE-RESISTANCE LOCUS RECEPTOR-LIKE PROTEIN KINASE-like 1.5;protein_id=XP_044399683.1

GO:0016021; Integral component of membrane

Kozak: TCCATGT ID=cds-XP_044399999.1;Parent=rna-XM_044544064.1;Dbxref=GeneID:123123543,Genbank:XP_044399999.1;Name=XP_044399999.1;gbkey=CDS;gene=LOC123123543;product=methyltransferase N6AMT1-like isoform X1;protein_id=XP_044399999.1

GO:0035657; eRF1 methyltransferase complex

Kozak: CCAATGC ID=cds-XP_044400005.1;Parent=rna-XM_044544070.1;Dbxref=GeneID:123123546,Genbank:XP_044400005.1;Name=XP_044400005.1;gbkey=CDS;gene=LOC123123546;product=DIMBOA UDP-glucosyltransferase BX8-like;protein_id=XP_044400005.1

GO:0080043; Quercetin 3-O-glucosyltransferase activity

Kozak: CAGATGC ID=cds-XP_044400012.1;Parent=rna-XM_044544077.1;Dbxref=GeneID:123123552,Genbank:XP_044400012.1;Name=XP_044400012.1;gbkey=CDS;gene=LOC123123552;product=protein DEFECTIVE IN EXINE FORMATION 1-like;protein_id=XP_044400012.1

GO:0016021; Integral component of membrane

Kozak: TCCATGT ID=cds-XP_044400022.1;Parent=rna-XM_044544087.1;Dbxref=GeneID:123123560,Genbank:XP_044400022.1;Name=XP_044400022.1;gbkey=CDS;gene=LOC123123560;product=GDP-mannose transporter GONST1-like;protein_id=XP_044400022.1

GO:0005794; Golgi apparatus

Kozak: CCAATGC ID=cds-XP_044400038.1;Parent=rna-XM_044544103.1;Dbxref=GeneID:123123576,Genbank:XP_044400038.1;Name=XP_044400038.1;gbkey=CDS;gene=LOC123123576;product=

serine/arginine repetitive matrix protein 1-like isoform X1;protein

Kozak: CCAATGT ID=cds-XP_044400216.1;Parent=rna-XM_044544281.1;Dbxref=GeneID:123123711,Genbank:XP_044400216.1;Name=XP_044400216.1;gbkey=CDS;gene=LOC123123711;product=ATP-dependent zinc metalloprotease FTSH 5%2C mitochondrial-like;protein_id=XP_044400216.1

GO:0009534; Chloroplast thylakoid

Kozak: CCAATGC ID=cds-XP_044400286.1;Parent=rna-XM_044544351.1;Dbxref=GeneID:123123753,Genbank:XP_044400286.1;Name=XP_044400286.1;gbkey=CDS;gene=LOC123123753;product=30S ribosomal protein S6 alpha%2C chloroplastic-like;protein_id=XP_044400286.1

GO:0005737; Cytoplasm

Kozak: CCCATGC ID=cds-XP_044400438.1;Parent=rna-XM_044544503.1;Dbxref=GeneID:123123861,Genbank:XP_044400438.1;Name=XP_044400438.1;gbkey=CDS;gene=LOC123123861;product=protein FRIGIDA-like;protein_id=XP_044400438.1

GO:0030154; Cell differentiation

Kozak: CAGATGC ID=cds-XP_044400533.1;Parent=rna-XM_044544598.1;Dbxref=GeneID:123123943,Genbank:XP_044400533.1;Name=XP_044400533.1;gbkey=CDS;gene=LOC123123943;product=

RING-H2 finger protein ATL34-like;protein

Kozak: TCCATGT ID=cds-XP_044400700.1;Parent=rna-XM_044544765.1;Dbxref=GeneID:123124059,Genbank:XP_044400700.1;Name=XP_044400700.1;gbkey=CDS;gene=LOC123124059;product=obtusifoliol 14-alpha demethylase-like;protein_id=XP_044400700.1

GO:0016021; Integral component of membrane

Kozak: TGTATGT ID=cds-XP_044400800.1;Parent=rna-XM_044544865.1;Dbxref=GeneID:123124205,Genbank:XP_044400800.1;Name=XP_044400800.1;gbkey=CDS;gene=LOC123124205;product=

putative protein FAR1-RELATED SEQUENCE 10 isoform X1;protein

Kozak: CCCATGT ID=cds-XP_044401073.1;Parent=rna-XM_044545138.1;Dbxref=GeneID:123124547,Genbank:XP_044401073.1;Name=XP_044401073.1;gbkey=CDS;gene=LOC123124547;product=G-box-binding factor 1-like;protein_id=XP_044401073.1

GO:0005634; Nucleus

Kozak: TCCATGT ID=cds-XP_044401142.1;Parent=rna-XM_044545207.1;Dbxref=GeneID:123124621,Genbank:XP_044401142.1;Name=XP_044401142.1;gbkey=CDS;gene=LOC123124621;product=probable phytol kinase 2%2C chloroplastic;protein_id=XP_044401142.1

GO:0031969; Chloroplast membrane

Kozak: TACATGC ID=cds-XP_044401196.1;Parent=rna-XM_044545261.1;Dbxref=GeneID:123124686,Genbank:XP_044401196.1;Name=XP_044401196.1;gbkey=CDS;gene=LOC123124686;product=uncharacterized protein LOC123124686;protein_id=XP_044401196.1

GO:0005739; Mitochondrion

Kozak: TGTATGT ID=cds-XP_044401252.1;Parent=rna-XM_044545317.1;Dbxref=GeneID:123124757,Genbank:XP_044401252.1;Name=XP_044401252.1;gbkey=CDS;gene=LOC123124757;product=UDP-glycosyltransferase 90A1-like;protein_id=XP_044401252.1

GO:0008194; UDP-glycosyltransferase activity

Kozak: CCAATGT ID=cds-XP_044401279.1;Parent=rna-XM_044545344.1;Dbxref=GeneID:123124789,Genbank:XP_044401279.1;Name=XP_044401279.1;gbkey=CDS;gene=LOC123124789;product=transcription factor TB1-like;protein_id=XP_044401279.1

GO:0005634; Nucleus

Kozak: CCCATGC ID=cds-XP_044401292.1;Parent=rna-XM_044545357.1;Dbxref=GeneID:123124802,Genbank:XP_044401292.1;Name=XP_044401292.1;gbkey=CDS;gene=LOC123124802;product=cytoplasmic tRNA 2-thiolation protein 1;protein_id=XP_044401292.1

GO:0002144; Cytosolic tRNA wobble base thiouridylase complex

Kozak: CGAATGT ID=cds-XP_044401388.1;Parent=rna-XM_044545453.1;Dbxref=GeneID:123124903,Genbank:XP_044401388.1;Name=XP_044401388.1;gbkey=CDS;gene=LOC123124903;product=polcalcin Phl p 7-like;protein_id=XP_044401388.1

GO:0005509; Calcium ion binding

Kozak: CCCATGT ID=cds-XP_044401455.1;Parent=rna-XM_044545520.1;Dbxref=GeneID:123124975,Genbank:XP_044401455.1;Name=XP_044401455.1;gbkey=CDS;gene=LOC123124975;product=ras-related protein RABB1c-like isoform X2;protein_id=XP_044401455.1

GO:0005794; Golgi apparatus

Kozak: TCCATGT ID=cds-XP_044401688.1;Parent=rna-XM_044545753.1;Dbxref=GeneID:123125225,Genbank:XP_044401688.1;Name=XP_044401688.1;gbkey=CDS;gene=LOC123125225;product=ethylene-responsive transcription factor ERF071-like;protein_id=XP_044401688.1

GO:0005634; Nucleus

Kozak: CCAATGC ID=cds-XP_044401709.1;Parent=rna-XM_044545774.1;Dbxref=GeneID:123125252,Genbank:XP_044401709.1;Name=XP_044401709.1;gbkey=CDS;gene=LOC123125252;product=putative cyclin-F1-1;protein_id=XP_044401709.1

GO:0000307; Cyclin-dependent protein kinase holoenzyme complex

Kozak: CCAATGC ID=cds-XP_044401710.1;Parent=rna-XM_044545775.1;Dbxref=GeneID:123125253,Genbank:XP_044401710.1;Name=XP_044401710.1;gbkey=CDS;gene=LOC123125253;product=putative cyclin-F1-1;protein_id=XP_044401710.1

GO:0000307; Cyclin-dependent protein kinase holoenzyme complex

Kozak: CAGATGC ID=cds-XP_044401926.1;Parent=rna-XM_044545991.1;Dbxref=GeneID:123125514,Genbank:XP_044401926.1;Name=XP_044401926.1;gbkey=CDS;gene=LOC123125514;product=guanine nucleotide-binding protein subunit gamma 1-like;protein_id=XP_044401926.1

GO:0007186; G protein-coupled receptor signaling pathway

Kozak: TACATGC ID=cds-XP_044402231.1;Parent=rna-XM_044546296.1;Dbxref=GeneID:123125849,Genbank:XP_044402231.1;Name=XP_044402231.1;gbkey=CDS;gene=LOC123125849;product=

PLASMODESMATA CALLOSE-BINDING PROTEIN 1-like;protein

Kozak: CCCATGC ID=cds-XP_044402264.1;Parent=rna-XM_044546329.1;Dbxref=GeneID:123125885,Genbank:XP_044402264.1;Name=XP_044402264.1;gbkey=CDS;gene=LOC123125885;product=photosynthetic NDH subunit of lumenal location 3%2C chloroplastic-like;protein_id=XP_044402264.1

GO:0009507; Chloroplast

Kozak: TACATGC ID=cds-XP_044402379.1;Parent=rna-XM_044546444.1;Dbxref=GeneID:123126026,Genbank:XP_044402379.1;Name=XP_044402379.1;gbkey=CDS;gene=LOC123126026;product=cytochrome P450 709B1-like;protein_id=XP_044402379.1

GO:0016021; Integral component of membrane

Kozak: TGTATGT ID=cds-XP_044402571.1;Parent=rna-XM_044546636.1;Dbxref=GeneID:123126272,Genbank:XP_044402571.1;Name=XP_044402571.1;gbkey=CDS;gene=LOC123126272;product=beta-fructofuranosidase%2C insoluble isoenzyme 4-like isoform X1;protein_id=XP_044402571.1

GO:0016021; Integral component of membrane

Kozak: CAGATGC ID=cds-XP_044394606.1;Parent=rna-XM_044538671.1;Dbxref=GeneID:123119032,Genbank:XP_044394606.1;Name=XP_044394606.1;gbkey=CDS;gene=LOC123119032;product=HIPL1 protein-like;protein_id=XP_044394606.1

GO:0003824; Catalytic activity

Kozak: TTCATGT ID=cds-XP_044394622.1;Parent=rna-XM_044538687.1;Dbxref=GeneID:123119038,Genbank:XP_044394622.1;Name=XP_044394622.1;gbkey=CDS;gene=LOC123119038;product=

pentatricopeptide repeat-containing protein At1g18900-like;protein

Kozak: CCAATGC ID=cds-XP_044394715.1;Parent=rna-XM_044538780.1;Dbxref=GeneID:123119104,Genbank:XP_044394715.1;Name=XP_044394715.1;gbkey=CDS;gene=LOC123119104;product=putative receptor-like protein kinase At4g00960 isoform X1;protein_id=XP_044394715.1

GO:0005886; Plasma membrane

Kozak: CCAATGT ID=cds-XP_044394781.1;Parent=rna-XM_044538846.1;Dbxref=GeneID:123119145,Genbank:XP_044394781.1;Name=XP_044394781.1;gbkey=CDS;gene=LOC123119145;product=nucleolar protein dao-5-like;protein_id=XP_044394781.1

GO:0005634; Nucleus

Kozak: TCCATGT ID=cds-XP_044394871.1;Parent=rna-XM_044538936.1;Dbxref=GeneID:123119222,Genbank:XP_044394871.1;Name=XP_044394871.1;gbkey=CDS;gene=LOC123119222;product=uncharacterized protein LOC123119222;protein_id=XP_044394871.1

GO:0005737; Cytoplasm

Kozak: CCAATGC ID=cds-XP_044394908.1;Parent=rna-XM_044538973.1;Dbxref=GeneID:123119247,Genbank:XP_044394908.1;Name=XP_044394908.1;gbkey=CDS;gene=LOC123119247;product=

LRR receptor-like serine/threonine-protein kinase GSO1 isoform X1;protein

Kozak: CCAATGC ID=cds-XP_044394911.1;Parent=rna-XM_044538976.1;Dbxref=GeneID:123119249,Genbank:XP_044394911.1;Name=XP_044394911.1;gbkey=CDS;gene=LOC123119249;product=ubiquitin carboxyl-terminal hydrolase 8-like;protein_id=XP_044394911.1

GO:0004843; Thiol-dependent deubiquitinase

Kozak: CCAATGC ID=cds-XP_044395140.1;Parent=rna-XM_044539205.1;Dbxref=GeneID:123119401,Genbank:XP_044395140.1;Name=XP_044395140.1;gbkey=CDS;gene=LOC123119401;product=importin subunit beta-1-like;protein_id=XP_044395140.1

GO:0005737; Cytoplasm

Kozak: TTCATGT ID=cds-XP_044395145.1;Parent=rna-XM_044539210.1;Dbxref=GeneID:123119406,Genbank:XP_044395145.1;Name=XP_044395145.1;gbkey=CDS;gene=LOC123119406;product=uncharacterized protein LOC123119406;protein_id=XP_044395145.1

GO:0005737; Cytoplasm

Kozak: CAGATGC ID=cds-XP_044395433.1;Parent=rna-XM_044539498.1;Dbxref=GeneID:123119638,Genbank:XP_044395433.1;Name=XP_044395433.1;gbkey=CDS;gene=LOC123119638;product=polyubiquitin isoform X1;protein_id=XP_044395433.1

GO:0005737; Cytoplasm

Kozak: CAGATGC ID=cds-XP_044395583.1;Parent=rna-XM_044539648.1;Dbxref=GeneID:123119743,Genbank:XP_044395583.1;Name=XP_044395583.1;gbkey=CDS;gene=LOC123119743;product=psbP domain-containing protein 1%2C chloroplastic-like;protein_id=XP_044395583.1

GO:0009507; Chloroplast

Kozak: CCCATGC ID=cds-XP_044395631.1;Parent=rna-XM_044539696.1;Dbxref=GeneID:123119774,Genbank:XP_044395631.1;Name=XP_044395631.1;gbkey=CDS;gene=LOC123119774;product=calmodulin-binding protein 60 A-like;protein_id=XP_044395631.1

GO:0005634; Nucleus

Kozak: TCCATGT ID=cds-XP_044395738.1;Parent=rna-XM_044539803.1;Dbxref=GeneID:123119861,Genbank:XP_044395738.1;Name=XP_044395738.1;gbkey=CDS;gene=LOC123119861;product=AP-5 complex subunit mu-like;protein_id=XP_044395738.1

GO:0030119; AP-type membrane coat adaptor complex

Kozak: TACATGC ID=cds-XP_044395789.1;Parent=rna-XM_044539854.1;Dbxref=GeneID:123119892,Genbank:XP_044395789.1;Name=XP_044395789.1;gbkey=CDS;gene=LOC123119892;product=protein CLT2%2C chloroplastic-like;protein_id=XP_044395789.1

GO:0016021; Integral component of membrane

Kozak: CCAATGC ID=cds-XP_044395926.1;Parent=rna-XM_044539991.1;Dbxref=GeneID:123120003,Genbank:XP_044395926.1;Name=XP_044395926.1;gbkey=CDS;gene=LOC123120003;product=calcium-dependent protein kinase 28;protein_id=XP_044395926.1

GO:0005739; Mitochondrion

Kozak: CAGATGC ID=cds-XP_044396380.1;Parent=rna-XM_044540445.1;Dbxref=GeneID:123120449,Genbank:XP_044396380.1;Name=XP_044396380.1;gbkey=CDS;gene=LOC123120449;product=probable protein S-acyltransferase 4 isoform X2;protein_id=XP_044396380.1

GO:0005783; Endoplasmic reticulum

Kozak: CCAATGT ID=cds-XP_044396506.1;Parent=rna-XM_044540571.1;Dbxref=GeneID:123120563,Genbank:XP_044396506.1;Name=XP_044396506.1;gbkey=CDS;gene=LOC123120563;product=uncharacterized protein LOC123120563;protein_id=XP_044396506.1

GO:0030014; C:CCR4-NOT complex

Kozak: TCCATGT ID=cds-XP_044396606.1;Parent=rna-XM_044540671.1;Dbxref=GeneID:123120684,Genbank:XP_044396606.1;Name=XP_044396606.1;gbkey=CDS;gene=LOC123120684;product=receptor like protein kinase S.2-like;protein_id=XP_044396606.1

GO:0005886; Plasma membrane

Kozak: CCAATGT ID=cds-XP_044396828.1;Parent=rna-XM_044540893.1;Dbxref=GeneID:123120931,Genbank:XP_044396828.1;Name=XP_044396828.1;gbkey=CDS;gene=LOC123120931;product=ethylene-responsive transcription factor ERF025-like;protein_id=XP_044396828.1

GO:0005634; Nucleus

Kozak: TCCATGT ID=cds-XP_044396996.1;Parent=rna-XM_044541061.1;Dbxref=GeneID:123121138,Genbank:XP_044396996.1;Name=XP_044396996.1;gbkey=CDS;gene=LOC123121138;product=

receptor-like cytosolic serine/threonine-protein kinase RBK1;protein

Kozak: CCCATGT ID=cds-XP_044397101.1;Parent=rna-XM_044541166.1;Dbxref=GeneID:123121259,Genbank:XP_044397101.1;Name=XP_044397101.1;gbkey=CDS;gene=LOC123121259;product=

B3 domain-containing protein Os01g0234100-like;protein

Kozak: CCCATGT ID=cds-XP_044397110.1;Parent=rna-XM_044541175.1;Dbxref=GeneID:123121273,Genbank:XP_044397110.1;Name=XP_044397110.1;gbkey=CDS;gene=LOC123121273;product=UDP-glucose 4-epimerase 4-like;protein_id=XP_044397110.1

GO:0005829; Cytosol

Kozak: CAGATGC ID=cds-XP_044397120.1;Parent=rna-XM_044541185.1;Dbxref=GeneID:123121280,Genbank:XP_044397120.1;Name=XP_044397120.1;gbkey=CDS;gene=LOC123121280;product=pentatricopeptide repeat-containing protein At4g02750-like;protein_id=XP_044397120.1

GO:0003723; RNA binding

Kozak: CCCATGC ID=cds-XP_044397802.1;Parent=rna-XM_044541867.1;Dbxref=GeneID:123121804,Genbank:XP_044397802.1;Name=XP_044397802.1;gbkey=CDS;gene=LOC123121804;product=uncharacterized protein LOC123121804;protein_id=XP_044397802.1

GO:0005634; Nucleus

Kozak: CCCATGC ID=cds-XP_044398118.1;Parent=rna-XM_044542183.1;Dbxref=GeneID:123122051,Genbank:XP_044398118.1;Name=XP_044398118.1;gbkey=CDS;gene=LOC123122051;product=oleoyl-acyl carrier protein thioesterase 1%2C chloroplastic-like;protein_id=XP_044398118.1

GO:0009507; Chloroplast

Kozak: CAGATGC ID=cds-XP_044398458.1;Parent=rna-XM_044542523.1;Dbxref=GeneID:123122337,Genbank:XP_044398458.1;Name=XP_044398458.1;gbkey=CDS;gene=LOC123122337;product=

wall-associated receptor kinase 5-like;protein

Kozak: CCCATGC ID=cds-XP_044398634.1;Parent=rna-XM_044542699.1;Dbxref=GeneID:123122501,Genbank:XP_044398634.1;Name=XP_044398634.1;gbkey=CDS;gene=LOC123122501;product=uncharacterized protein LOC123122501;protein_id=XP_044398634.1

GO:0015031; Protein transport

Kozak: CCCATGT ID=cds-XP_044398710.1;Parent=rna-XM_044542775.1;Dbxref=GeneID:123122566,Genbank:XP_044398710.1;Name=XP_044398710.1;gbkey=CDS;gene=LOC123122566;product=F-box/kelch-repeat protein At5g42350-like;protein_id=XP_044398710.1

GO:0019005; SCF ubiquitin ligase complex

Kozak: CCCATGT ID=cds-XP_044399025.1;Parent=rna-XM_044543090.1;Dbxref=GeneID:123122752,Genbank:XP_044399025.1;Name=XP_044399025.1;gbkey=CDS;gene=LOC123122752;product=F-box/LRR-repeat protein 17-like isoform X1;protein_id=XP_044399025.1

GO:0005737; Cytoplasm

Kozak: CCAATGT ID=cds-XP_044399159.1;Parent=rna-XM_044543224.1;Dbxref=GeneID:123122863,Genbank:XP_044399159.1;Name=XP_044399159.1;gbkey=CDS;gene=LOC123122863;product=two pore potassium channel a-like;protein_id=XP_044399159.1

GO:0005887; Integral component of plasma membrane

Kozak: CCCATGC ID=cds-XP_044399182.1;Parent=rna-XM_044543247.1;Dbxref=GeneID:123122878,Genbank:XP_044399182.1;Name=XP_044399182.1;gbkey=CDS;gene=LOC123122878;product=protein LEO1 homolog isoform X1;protein_id=XP_044399182.1

GO:0016593; Cdc73/Paf1 complex

Kozak: CCCATGT ID=cds-XP_044399282.1;Parent=rna-XM_044543347.1;Dbxref=GeneID:123122962,Genbank:XP_044399282.1;Name=XP_044399282.1;gbkey=CDS;gene=LOC123122962;product=probable hexosyltransferase MUCI70;protein_id=XP_044399282.1

GO:0016021; Integral component of membrane

Kozak: CCCATGC ID=cds-XP_044399309.1;Parent=rna-XM_044543374.1;Dbxref=GeneID:123122984,Genbank:XP_044399309.1;Name=XP_044399309.1;gbkey=CDS;gene=LOC123122984;product=probable LRR receptor-like serine/threonine-protein kinase At2g16250;protein_id=XP_044399309.1

GO:0016021; Integral component of membrane

Kozak: TTAATGC ID=cds-XP_044399871.1;Parent=rna-XM_044543936.1;Dbxref=GeneID:123123434,Genbank:XP_044399871.1;Name=XP_044399871.1;gbkey=CDS;gene=LOC123123434;product=serine/threonine/tyrosine-protein kinase HT1-like;protein_id=XP_044399871.1

GO:0005737; Cytoplasm

Kozak: CAGATGC ID=cds-XP_044400271.1;Parent=rna-XM_044544336.1;Dbxref=GeneID:123123741,Genbank:XP_044400271.1;Name=XP_044400271.1;gbkey=CDS;gene=LOC123123741;product=UDP-glycosyltransferase 73E1-like;protein_id=XP_044400271.1

GO:0008194; UDP-glycosyltransferase activity

Kozak: CCCATGT ID=cds-XP_044400470.1;Parent=rna-XM_044544535.1;Dbxref=GeneID:123123894,Genbank:XP_044400470.1;Name=XP_044400470.1;gbkey=CDS;gene=LOC123123894;product=mitogen-activated protein kinase kinase 3-like;protein_id=XP_044400470.1

GO:0005524; ATP binding

Kozak: TCCATGT ID=cds-XP_044400774.1;Parent=rna-XM_044544839.1;Dbxref=GeneID:123124171,Genbank:XP_044400774.1;Name=XP_044400774.1;gbkey=CDS;gene=LOC123124171;product=

heat shock 70 kDa protein BIP1-like;protein

Kozak: TACATGC ID=cds-XP_044400952.1;Parent=rna-XM_044545017.1;Dbxref=GeneID:123124401,Genbank:XP_044400952.1;Name=XP_044400952.1;gbkey=CDS;gene=LOC123124401;product=

glutathione S-transferase T3-like;protein

Kozak: TCCATGT ID=cds-XP_044401026.1;Parent=rna-XM_044545091.1;Dbxref=GeneID:123124477,Genbank:XP_044401026.1;Name=XP_044401026.1;gbkey=CDS;gene=LOC123124477;product=NDR1/HIN1-like protein 26;protein_id=XP_044401026.1

GO:0046658; Anchored component of plasma membrane

Kozak: CCCATGT ID=cds-XP_044401356.1;Parent=rna-XM_044545421.1;Dbxref=GeneID:123124878,Genbank:XP_044401356.1;Name=XP_044401356.1;gbkey=CDS;gene=LOC123124878;product=uncharacterized protein LOC123124878;protein_id=XP_044401356.1

GO:0016021; Integral component of membrane

Kozak: TCCATGT ID=cds-XP_044401415.1;Parent=rna-XM_044545480.1;Dbxref=GeneID:123124932,Genbank:XP_044401415.1;Name=XP_044401415.1;gbkey=CDS;gene=LOC123124932;product=AT-hook motif nuclear-localized protein 17-like;protein_id=XP_044401415.1

GO:0016021; Integral component of membrane

Kozak: CCCATGT ID=cds-XP_044401438.1;Parent=rna-XM_044545503.1;Dbxref=GeneID:123124958,Genbank:XP_044401438.1;Name=XP_044401438.1;gbkey=CDS;gene=LOC123124958;product=uncharacterized protein LOC123124958;protein_id=XP_044401438.1

GO:0016021; Integral component of membrane

Kozak: CCAATGC ID=cds-XP_044401715.1;Parent=rna-XM_044545780.1;Dbxref=GeneID:123125259,Genbank:XP_044401715.1;Name=XP_044401715.1;gbkey=CDS;gene=LOC123125259;product=

Putative cyclin-F1-1;protein.

Kozak: CCAATGC ID=cds-XP_044401716.1;Parent=rna-XM_044545781.1;Dbxref=GeneID:123125260,Genbank:XP_044401716.1;Name=XP_044401716.1;gbkey=CDS;gene=LOC123125260;product=

Putative cyclin-F1-1;protein.

Kozak: CCAATGT ID=cds-XP_044400542.1;Parent=rna-XM_044544607.1;Dbxref=GeneID:778386,Genbank:XP_044400542.1;Name=XP_044400542.1;gbkey=CDS;gene=LOC778386;product=beta-fructofuranosidase%2C insoluble isoenzyme 4 isoform X2;protein_id=XP_044400542.1

GO:0016756; Glutathione gamma-glutamylcysteinyltransferase activity.

Kozak: TCCATGT ID=cds-XP_044352804.1;Parent=rna-XM_044496869.1;Dbxref=GeneID:123073858,Genbank:XP_044352804.1;Name=XP_044352804.1;gbkey=CDS;gene=LOC123073858;product=UDP-glycosyltransferase 88B1-like;protein_id=XP_044352804.1

GO:0008194; UDP-glycosyltransferase activity.

Kozak: CCAATGT ID=cds-XP_044353373.1;Parent=rna-XM_044497438.1;Dbxref=GeneID:123074653,Genbank:XP_044353373.1;Name=XP_044353373.1;gbkey=CDS;gene=LOC123074653;product=uncharacterized protein LOC123074653;protein_id=XP_044353373.1

GO:0030599; Pectinesterase activity.

Kozak: CCCATGC ID=cds-XP_044353642.1;Parent=rna-XM_044497707.1;Dbxref=GeneID:123075009,Genbank:XP_044353642.1;Name=XP_044353642.1;gbkey=CDS;gene=LOC123075009;product=zinc finger protein 6-like;protein_id=XP_044353642.1

GO:0005634; Nucleus.

Kozak: TTCATGT ID=cds-XP_044355016.1;Parent=rna-XM_044499081.1;Dbxref=GeneID:123076927,Genbank:XP_044355016.1;Name=XP_044355016.1;gbkey=CDS;gene=LOC123076927;product=vacuolar-processing enzyme beta-isozyme 1-like;protein_id=XP_044355016.1

GO:0005773; Vacuole.

Kozak: CCCATGT ID=cds-XP_044355024.1;Parent=rna-XM_044499089.1;Dbxref=GeneID:123076931,Genbank:XP_044355024.1;Name=XP_044355024.1;gbkey=CDS;gene=LOC123076931;product=uncharacterized protein LOC123076931;protein_id=XP_044355024.1

GO:0005634; Nucleus.

Kozak: TCCATGT ID=cds-XP_044355419.1;Parent=rna-XM_044499484.1;Dbxref=GeneID:123077250,Genbank:XP_044355419.1;Name=XP_044355419.1;gbkey=CDS;gene=LOC123077250;product=probable uridine nucleosidase 2;protein_id=XP_044355419.1

GO:0005829; Cytosol.

Kozak: CCAATGC ID=cds-XP_044355823.1;Parent=rna-XM_044499888.1;Dbxref=GeneID:123077598,Genbank:XP_044355823.1;Name=XP_044355823.1;gbkey=CDS;gene=LOC123077598;product=O-fucosyltransferase 1-like isoform X1;protein_id=XP_044355823.1

GO:0005737; Cytoplasm.

Kozak: CCAATGC ID=cds-XP_044355993.1;Parent=rna-XM_044500058.1;Dbxref=GeneID:123077742,Genbank:XP_044355993.1;Name=XP_044355993.1;gbkey=CDS;gene=LOC123077742;product=aarF domain-containing protein kinase 1-like isoform X1;protein_id=XP_044355993.1

GO:0032592; Integral component of mitochondrial membrane.

Kozak: CCCATGT ID=cds-XP_044356169.1;Parent=rna-XM_044500234.1;Dbxref=GeneID:123077879,Genbank:XP_044356169.1;Name=XP_044356169.1;gbkey=CDS;gene=LOC123077879;product=auxin-responsive protein IAA3-like;protein_id=XP_044356169.1

GO:0005634; Nucleus.

Kozak: CAGATGC ID=cds-XP_044356416.1;Parent=rna-XM_044500481.1;Dbxref=GeneID:123078087,Genbank:XP_044356416.1;Name=XP_044356416.1;gbkey=CDS;gene=LOC123078087;product=bifunctional phosphatase IMPL2%2C chloroplastic-like;protein_id=XP_044356416.1

GO:0004401; Histidinol-phosphatase activity.

Kozak: CCAATGC ID=cds-XP_044356819.1;Parent=rna-XM_044500884.1;Dbxref=GeneID:123078390,Genbank:XP_044356819.1;Name=XP_044356819.1;gbkey=CDS;gene=LOC123078390;product=uncharacterized protein LOC123078390;protein_id=XP_044356819.1

GO; GO:0016021; Integral component of membrane.

Kozak: TTCATGT ID=cds-XP_044356985.1;Parent=rna-XM_044501050.1;Dbxref=GeneID:123078515,Genbank:XP_044356985.1;Name=XP_044356985.1;gbkey=CDS;gene=LOC123078515;product=pentatricopeptide repeat-containing protein At4g13650-like;protein_id=XP_044356985.1

GO:0003723; RNA binding.

Kozak: CCAATGC ID=cds-XP_044357155.1;Parent=rna-XM_044501220.1;Dbxref=GeneID:123078642,Genbank:XP_044357155.1;Name=XP_044357155.1;gbkey=CDS;gene=LOC123078642;product=probable glycosyltransferase At5g03795;protein_id=XP_044357155.1

GO:0005794; Golgi apparatus.

Kozak: CCCATGT ID=cds-XP_044357187.1;Parent=rna-XM_044501252.1;Dbxref=GeneID:123078666,Genbank:XP_044357187.1;Name=XP_044357187.1;gbkey=CDS;gene=LOC123078666;product=uncharacterized protein LOC123078666;protein_id=XP_044357187.1

GO:0004842; Ubiquitin-protein transferase activity.

Kozak: CCAATGC ID=cds-XP_044357347.1;Parent=rna-XM_044501412.1;Dbxref=GeneID:123078805,Genbank:XP_044357347.1;Name=XP_044357347.1;gbkey=CDS;gene=LOC123078805;product=

Pentatricopeptide repeat-containing protein At4g02750-like;protein.

Kozak: TCCATGT ID=cds-XP_044357357.1;Parent=rna-XM_044501422.1;Dbxref=GeneID:123078809,Genbank:XP_044357357.1;Name=XP_044357357.1;gbkey=CDS;gene=LOC123078809;product=ras-related protein Rab7-like;protein_id=XP_044357357.1

GO:0005774; Vacuolar membrane.

Kozak: CCCATGT ID=cds-XP_044357358.1;Parent=rna-XM_044501423.1;Dbxref=GeneID:123078811,Genbank:XP_044357358.1;Name=XP_044357358.1;gbkey=CDS;gene=LOC123078811;product=

protein PLASTID MOVEMENT IMPAIRED 1-RELATED 1-like;protein.

Kozak: CCAATGC ID=cds-XP_044357674.1;Parent=rna-XM_044501739.1;Dbxref=GeneID:123079068,Genbank:XP_044357674.1;Name=XP_044357674.1;gbkey=CDS;gene=LOC123079068;product=lysM domain receptor-like kinase 3;protein_id=XP_044357674.1

GO:0016021; Integral component of membrane.

Kozak: CCCATGC ID=cds-XP_044358054.1;Parent=rna-XM_044502119.1;Dbxref=GeneID:123079355,Genbank:XP_044358054.1;Name=XP_044358054.1;gbkey=CDS;gene=LOC123079355;product=small RNA degrading nuclease 5-like;protein_id=XP_044358054.1

GO:0005634; Nucleus.

Kozak: CCAATGT ID=cds-XP_044358088.1;Parent=rna-XM_044502153.1;Dbxref=GeneID:123079384,Genbank:XP_044358088.1;Name=XP_044358088.1;gbkey=CDS;gene=LOC123079384;product=

External alternative NAD(P)H-ubiquinone oxidoreductase B3%2C mitochondrial-like;protein.

Kozak: CCCATGT ID=cds-XP_044358152.1;Parent=rna-XM_044502217.1;Dbxref=GeneID:123079448,Genbank:XP_044358152.1;Name=XP_044358152.1;gbkey=CDS;gene=LOC123079448;product=pentatricopeptide repeat-containing protein At1g33350-like;protein_id=XP_044358152.1

GO:0003986; Acetyl-CoA hydrolase activity.

Kozak: CAGATGC ID=cds-XP_044358155.1;Parent=rna-XM_044502220.1;Dbxref=GeneID:123079450,Genbank:XP_044358155.1;Name=XP_044358155.1;gbkey=CDS;gene=LOC123079450;product=DNA-(apurinic or apyrimidinic site) endonuclease%2C chloroplastic-like isoform X1;protein_id=XP_044358155.1

GO:0005634; Nucleus.

Kozak: CCCATGT ID=cds-XP_044358177.1;Parent=rna-XM_044502242.1;Dbxref=GeneID:123079466,Genbank:XP_044358177.1;Name=XP_044358177.1;gbkey=CDS;gene=LOC123079466;product=

Pentatricopeptide repeat-containing protein At1g55890%2C mitochondrial-like;protein.

Kozak: CCCATGT ID=cds-XP_044358357.1;Parent=rna-XM_044502422.1;Dbxref=GeneID:123079627,Genbank:XP_044358357.1;Name=XP_044358357.1;gbkey=CDS;gene=LOC123079627;product=uncharacterized protein At5g39865-like;protein_id=XP_044358357.1

GO:0097573; Glutathione oxidoreductase activity.

Kozak: CCCATGC ID=cds-XP_044358797.1;Parent=rna-XM_044502862.1;Dbxref=GeneID:123080009,Genbank:XP_044358797.1;Name=XP_044358797.1;gbkey=CDS;gene=LOC123080009;product=

Endochitinase A-like;protein.

Kozak: CCAATGC ID=cds-XP_044359225.1;Parent=rna-XM_044503290.1;Dbxref=GeneID:123080383,Genbank:XP_044359225.1;Name=XP_044359225.1;gbkey=CDS;gene=LOC123080383;product=p

Hosphatidylinositol transfer protein 3-like;protein.

Kozak: CAGATGC ID=cds-XP_044359231.1;Parent=rna-XM_044503296.1;Dbxref=GeneID:123080389,Genbank:XP_044359231.1;Name=XP_044359231.1;gbkey=CDS;gene=LOC123080389;product=auxin response factor 4-like;protein_id=XP_044359231.1

GO:0005634; Nucleus.

Kozak: CCCATGC ID=cds-XP_044359363.1;Parent=rna-XM_044503428.1;Dbxref=GeneID:123080500,Genbank:XP_044359363.1;Name=XP_044359363.1;gbkey=CDS;gene=LOC123080500;product=uncharacterized protein LOC123080500;protein_id=XP_044359363.1

GO:0016021; Integral component of membrane.

Kozak: CAGATGC ID=cds-XP_044359719.1;Parent=rna-XM_044503784.1;Dbxref=GeneID:123080834,Genbank:XP_044359719.1;Name=XP_044359719.1;gbkey=CDS;gene=LOC123080834;product=E3 ubiquitin-protein ligase RNF170-like;protein_id=XP_044359719.1

GO:0005789; Endoplasmic reticulum membrane.

Kozak: CCCATGC ID=cds-XP_044352916.1;Parent=rna-XM_044496981.1;Dbxref=GeneID:123074024,Genbank:XP_044352916.1;Name=XP_044352916.1;gbkey=CDS;gene=LOC123074024;product=uncharacterized protein LOC123074024;protein_id=XP_044352916.1

GO:0016021; Integral component of membrane.

Kozak: CCAATGT ID=cds-XP_044353400.1;Parent=rna-XM_044497465.1;Dbxref=GeneID:123074689,Genbank:XP_044353400.1;Name=XP_044353400.1;gbkey=CDS;gene=LOC123074689;product=probable purine permease 4;protein_id=XP_044353400.1

GO:0016021; Integral component of membrane.

Kozak: CCCATGT ID=cds-XP_044353403.1;Parent=rna-XM_044497468.1;Dbxref=GeneID:123074692,Genbank:XP_044353403.1;Name=XP_044353403.1;gbkey=CDS;gene=LOC123074692;product=

Cilia- and flagella-associated protein 70-like;protein.

Kozak: TCCATGT ID=cds-XP_044353531.1;Parent=rna-XM_044497596.1;Dbxref=GeneID:123074864,Genbank:XP_044353531.1;Name=XP_044353531.1;gbkey=CDS;gene=LOC123074864;product=probable DNA replication complex GINS protein PSF3 isoform X1;protein_id=XP_044353531.1

GO:0000811; GINS complex.

Kozak: CCCATGC ID=cds-XP_044353631.1;Parent=rna-XM_044497696.1;Dbxref=GeneID:123074995,Genbank:XP_044353631.1;Name=XP_044353631.1;gbkey=CDS;gene=LOC123074995;product=cis-prenyltransferase 4%2C chloroplastic-like;protein_id=XP_044353631.1

GO:0005783; Endoplasmic reticulum.

Kozak: CCAATGT ID=cds-XP_044353654.1;Parent=rna-XM_044497719.1;Dbxref=GeneID:123075027,Genbank:XP_044353654.1;Name=XP_044353654.1;gbkey=CDS;gene=LOC123075027;product=ABC transporter A family member 7-like isoform X1;protein_id=XP_044353654.1

GO:0016021; Integral component of membrane.

Kozak: TCCATGT ID=cds-XP_044354681.1;Parent=rna-XM_044498746.1;Dbxref=GeneID:123076571,Genbank:XP_044354681.1;Name=XP_044354681.1;gbkey=CDS;gene=LOC123076571;product=

B3 domain-containing protein Os03g0620400-like;protein

Kozak: CCCATGC ID=cds-XP_044354762.1;Parent=rna-XM_044498827.1;Dbxref=GeneID:123076715,Genbank:XP_044354762.1;Name=XP_044354762.1;gbkey=CDS;gene=LOC123076715;product=protein EXECUTER 2%2C chloroplastic-like;protein_id=XP_044354762.1

GO:0042651; Thylakoid membrane.

Kozak: CAGATGC ID=cds-XP_044354803.1;Parent=rna-XM_044498868.1;Dbxref=GeneID:123076754,Genbank:XP_044354803.1;Name=XP_044354803.1;gbkey=CDS;gene=LOC123076754;product=dephospho-CoA kinase;protein_id=XP_044354803.1

GO:0005524; ATP binding.

Kozak: CCCATGC ID=cds-XP_044355043.1;Parent=rna-XM_044499108.1;Dbxref=GeneID:123076942,Genbank:XP_044355043.1;Name=XP_044355043.1;gbkey=CDS;gene=LOC123076942;product=

LEAF RUST 10 DISEASE-RESISTANCE LOCUS RECEPTOR-LIKE PROTEIN KINASE-like 1.2 isoform X2;protein.

Kozak: CCCATGC ID=cds-XP_044355214.1;Parent=rna-XM_044499279.1;Dbxref=GeneID:123077076,Genbank:XP_044355214.1;Name=XP_044355214.1;gbkey=CDS;gene=LOC123077076;product=

L-type lectin-domain containing receptor kinase SIT2-like;protein.

Kozak: TTAATGC ID=cds-XP_044355393.1;Parent=rna-XM_044499458.1;Dbxref=GeneID:123077227,Genbank:XP_044355393.1;Name=XP_044355393.1;gbkey=CDS;gene=LOC123077227;product=putative pentatricopeptide repeat-containing protein At2g01510;protein_id=XP_044355393.1

GO:0008270; Zinc ion binding.

Kozak: TCCATGT ID=cds-XP_044355415.1;Parent=rna-XM_044499480.1;Dbxref=GeneID:123077246,Genbank:XP_044355415.1;Name=XP_044355415.1;gbkey=CDS;gene=LOC123077246;product=small G protein signaling modulator 1-like;protein_id=XP_044355415.1

GO:0005096; GTPase activator activity.

Kozak: CCCATGT ID=cds-XP_044355573.1;Parent=rna-XM_044499638.1;Dbxref=GeneID:123077359,Genbank:XP_044355573.1;Name=XP_044355573.1;gbkey=CDS;gene=LOC123077359;product=transcription initiation factor TFIID subunit 15b-like;protein_id=XP_044355573.1

GO:0046872; Metal ion binding.

Kozak: CCCATGC ID=cds-XP_044355744.1;Parent=rna-XM_044499809.1;Dbxref=GeneID:123077530,Genbank:XP_044355744.1;Name=XP_044355744.1;gbkey=CDS;gene=LOC123077530;product=uncharacterized protein LOC123077530;protein_id=XP_044355744.1

GO:0006357; Reregulation of transcription by RNA polymerase II.

Kozak: CAGATGC ID=cds-XP_044355773.1;Parent=rna-XM_044499838.1;Dbxref=GeneID:123077555,Genbank:XP_044355773.1;Name=XP_044355773.1;gbkey=CDS;gene=LOC123077555;product=

Transcription factor GTE9-like isoform X1;protein.

Kozak: CCCATGC ID=cds-XP_044355851.1;Parent=rna-XM_044499916.1;Dbxref=GeneID:123077617,Genbank:XP_044355851.1;Name=XP_044355851.1;gbkey=CDS;gene=LOC123077617;product=ABC transporter C family member 3-like;protein_id=XP_044355851.1

GO:0016021; integral component of membrane.

Kozak: CCCATGT ID=cds-XP_044356039.1;Parent=rna-XM_044500104.1;Dbxref=GeneID:123077779,Genbank:XP_044356039.1;Name=XP_044356039.1;gbkey=CDS;gene=LOC123077779;product=uncharacterized protein LOC123077779;protein_id=XP_044356039.1

GO:0016021; Integral component of membrane.

Kozak: TTCATGT ID=cds-XP_044356536.1;Parent=rna-XM_044500601.1;Dbxref=GeneID:123078190,Genbank:XP_044356536.1;Name=XP_044356536.1;gbkey=CDS;gene=LOC123078190;product=

Exportin-7-A-like isoform X1.

Kozak: CCCATGT ID=cds-XP_044356718.1;Parent=rna-XM_044500783.1;Dbxref=GeneID:123078315,Genbank:XP_044356718.1;Name=XP_044356718.1;gbkey=CDS;gene=LOC123078315;product=uncharacterized protein LOC123078315;protein_id=XP_044356718.1

GO:0034098; VCP-NPL4-UFD1 AAA ATPase complex.

Koxak: TGTATGT ID=cds-XP_044356767.1;Parent=rna-XM_044500832.1;Dbxref=GeneID:123078356,Genbank:XP_044356767.1;Name=XP_044356767.1;gbkey=CDS;gene=LOC123078356;product=

Protein LNK2-like isoform X1.

Kozak: TCCATGT ID=cds-XP_044356883.1;Parent=rna-XM_044500948.1;Dbxref=GeneID:123078441,Genbank:XP_044356883.1;Name=XP_044356883.1;gbkey=CDS;gene=LOC123078441;product=protein CONSERVED IN THE GREEN LINEAGE AND DIATOMS 27%2C chloroplastic-like;protein_id=XP_044356883.1

GO:0016021; Integral component of membrane.

Kozak: CCAATGC ID=cds-XP_044357322.1;Parent=rna-XM_044501387.1;Dbxref=GeneID:123078781,Genbank:XP_044357322.1;Name=XP_044357322.1;Note=The sequence of the model RefSeq protein was modified relative to this genomic sequence to represent the inferred CDS: added 215 bases not found in genome assembly;end_range=343292929,.;exception=annotated by transcript or proteomic data;gbkey=CDS;gene=LOC123078781;inference=similar to RNA sequence (same species):INSD:GFFI01009835.1;partial=true;product=

Pentatricopeptide repeat-containing protein At5g46100-like.

Kozak: CCCATGC ID=cds-XP_044357376.1;Parent=rna-XM_044501441.1;Dbxref=GeneID:123078817,Genbank:XP_044357376.1;Name=XP_044357376.1;gbkey=CDS;gene=LOC123078817;product=B3 domain-containing protein LFL1-like isoform X1;protein_id=XP_044357376.1

GO:0005634; Nucleus.

Kozak: CCAATGT ID=cds-XP_044357592.1;Parent=rna-XM_044501657.1;Dbxref=GeneID:123078993,Genbank:XP_044357592.1;Name=XP_044357592.1;gbkey=CDS;gene=LOC123078993;product=auxin-responsive protein IAA5-like;protein_id=XP_044357592.1

GO:0005634; Nucleus.

Kozak: CCCATGC ID=cds-XP_044357594.1;Parent=rna-XM_044501659.1;Dbxref=GeneID:123078995,Genbank:XP_044357594.1;Name=XP_044357594.1;gbkey=CDS;gene=LOC123078995;product=

Protein EXECUTER 2%2C chloroplastic-like.

Kozak: TCCATGT ID=cds-XP_044357690.1;Parent=rna-XM_044501755.1;Dbxref=GeneID:123079081,Genbank:XP_044357690.1;Name=XP_044357690.1;gbkey=CDS;gene=LOC123079081;product=serine/threonine-protein phosphatase 7 long form homolog isoform X1;protein_id=XP_044357690.1

GO:0010073; Meristem maintenance.

Kozak: CCCATGC ID=cds-XP_044357828.1;Parent=rna-XM_044501893.1;Dbxref=GeneID:123079188,Genbank:XP_044357828.1;Name=XP_044357828.1;gbkey=CDS;gene=LOC123079188;product=SCO-spondin-like;protein_id=XP_044357828.1 GO;

GO:0017177; Glucosidase II complex.

Kozak: CCCATGC ID=cds-XP_044357957.1;Parent=rna-XM_044502022.1;Dbxref=GeneID:123079285,Genbank:XP_044357957.1;Name=XP_044357957.1;gbkey=CDS;gene=LOC123079285;product=lysosomal Pro-X carboxypeptidase-like;protein_id=XP_044357957.1

GO:0008239; Dipeptidyl-peptidase activity.

Kozak: TTCATGT ID=cds-XP_044358130.1;Parent=rna-XM_044502195.1;Dbxref=GeneID:123079424,Genbank:XP_044358130.1;Name=XP_044358130.1;gbkey=CDS;gene=LOC123079424;product=protein translation factor SUI1 homolog;protein_id=XP_044358130.1

GO:0003723; RNA binding.

Kozak: CCCATGT ID=cds-XP_044358712.1;Parent=rna-XM_044502777.1;Dbxref=GeneID:123079943,Genbank:XP_044358712.1;Name=XP_044358712.1;gbkey=CDS;gene=LOC123079943;product=ubiquitin-like domain-containing CTD phosphatase;protein_id=XP_044358712.1

GO:0046658; Anchored component of plasma membrane.

Kozak: TTCATGT ID=cds-XP_044359127.1;Parent=rna-XM_044503192.1;Dbxref=GeneID:123080283,Genbank:XP_044359127.1;Name=XP_044359127.1;gbkey=CDS;gene=LOC123080283;product=protein CPR-5-like;protein_id=XP_044359127.1

GO:0016021; Integral component of membrane.

Kozak: CAGATGC ID=cds-XP_044359265.1;Parent=rna-XM_044503330.1;Dbxref=GeneID:123080407,Genbank:XP_044359265.1;Name=XP_044359265.1;gbkey=CDS;gene=LOC123080407;product=probable LRR receptor-like serine/threonine-protein kinase At3g47570;protein_id=XP_044359265.1

GO:0016021; Integral component of membrane.

Kozak: CAGATGC ID=cds-XP_044359507.1;Parent=rna-XM_044503572.1;Dbxref=GeneID:123080644,Genbank:XP_044359507.1;Name=XP_044359507.1;gbkey=CDS;gene=LOC123080644;product=probable inactive purple acid phosphatase 16;protein_id=XP_044359507.1

GO:0016788; Hydrolase activity, acting on ester bonds.

Kozak: TACATGC ID=cds-XP_044359906.1;Parent=rna-XM_044503971.1;Dbxref=GeneID:123081011,Genbank:XP_044359906.1;Name=XP_044359906.1;gbkey=CDS;gene=LOC123081011;product=uncharacterized protein LOC123081011;protein_id=XP_044359906.1

GO:0005634; Nucleus.

Kozak: CAGATGC ID=cds-XP_044386793.1;Parent=rna-XM_044530858.1;Dbxref=GeneID:123110364,Genbank:XP_044386793.1;Name=XP_044386793.1;gbkey=CDS;gene=LOC123110364;product=

Pentatricopeptide repeat-containing.

Kozak: TGTATGT ID=cds-XP_044386855.1;Parent=rna-XM_044530920.1;Dbxref=GeneID:123110419,Genbank:XP_044386855.1;Name=XP_044386855.1;gbkey=CDS;gene=LOC123110419;product=uncharacterized protein YnbB-like isoform X1;protein_id=XP_044386855.1

GO:0003824; Catalytic activity.

Kozak: TACATGC ID=cds-XP_044386933.1;Parent=rna-XM_044530998.1;Dbxref=GeneID:123110473,Genbank:XP_044386933.1;Name=XP_044386933.1;gbkey=CDS;gene=LOC123110473;product=

DNA topoisomerase 2-like isoform X1.

Kozak: CCCATGC ID=cds-XP_044386973.1;Parent=rna-XM_044531038.1;Dbxref=GeneID:123110502,Genbank:XP_044386973.1;Name=XP_044386973.1;gbkey=CDS;gene=LOC123110502;product=gamma-tubulin complex component 5-like isoform X1;protein_id=XP_044386973.1

GO:0005737; Cytoplasm.

Kozak: CCAATGT ID=cds-XP_044387121.1;Parent=rna-XM_044531186.1;Dbxref=GeneID:123110625,Genbank:XP_044387121.1;Name=XP_044387121.1;gbkey=CDS;gene=LOC123110625;product=pentatricopeptide repeat-containing protein At3g29230-like;protein_id=XP_044387121.1

GO:0005737; Cytoplasm.

Kozak: CCCATGT ID=cds-XP_044387285.1;Parent=rna-XM_044531350.1;Dbxref=GeneID:123110752,Genbank:XP_044387285.1;Name=XP_044387285.1;gbkey=CDS;gene=LOC123110752;product=psbP domain-containing protein 1%2C chloroplastic-like;protein_id=XP_044387285.1

GO:0009507; Chloroplast.

Kozak: CCCATGC ID=cds-XP_044387599.1;Parent=rna-XM_044531664.1;Dbxref=GeneID:123111009,Genbank:XP_044387599.1;Name=XP_044387599.1;gbkey=CDS;gene=LOC123111009;product=chaperone protein dnaJ A7A%2C chloroplastic-like;protein_id=XP_044387599.1

GO:0005737; Cytoplasm.

Kozak: TTCATGT ID=cds-XP_044387950.1;Parent=rna-XM_044532015.1;Dbxref=GeneID:123111256,Genbank:XP_044387950.1;Name=XP_044387950.1;gbkey=CDS;gene=LOC123111256;product=

Pentatricopeptide repeat-containing protein At1g52620-like.

Kozak: CCAATGC ID=cds-XP_044388107.1;Parent=rna-XM_044532172.1;Dbxref=GeneID:123111383,Genbank:XP_044388107.1;Name=XP_044388107.1;gbkey=CDS;gene=LOC123111383;product=putative pentatricopeptide repeat-containing protein At3g08820;protein_id=XP_044388107.1

GO:0008270; Zinc ion binding.

Kozak: TCCATGT ID=cds-XP_044388143.1;Parent=rna-XM_044532208.1;Dbxref=GeneID:123111414,Genbank:XP_044388143.1;Name=XP_044388143.1;gbkey=CDS;gene=LOC123111414;product=protein NRT1/ PTR FAMILY 2.7-like;protein_id=XP_044388143.1

GO:0016021; integral component of membrane.

Kozak: CAGATGC ID=cds-XP_044388165.1;Parent=rna-XM_044532230.1;Dbxref=GeneID:123111431,Genbank:XP_044388165.1;Name=XP_044388165.1;gbkey=CDS;gene=LOC123111431;product=glucan endo-1%2C3-beta-glucosidase 4-like isoform X1;protein_id=XP_044388165.1

GO:0016021; Integral component of membrane.

Kozak: CCCATGT ID=cds-XP_044389147.1;Parent=rna-XM_044533212.1;Dbxref=GeneID:123112264,Genbank:XP_044389147.1;Name=XP_044389147.1;gbkey=CDS;gene=LOC123112264;product=phosphoribosylaminoimidazole-succinocarboxamide synthase%2C chloroplastic-like;protein_id=XP_044389147.1

GO:0005524; ATP binding.

Kozak: TTCATGT ID=cds-XP_044389196.1;Parent=rna-XM_044533261.1;Dbxref=GeneID:123112310,Genbank:XP_044389196.1;Name=XP_044389196.1;gbkey=CDS;gene=LOC123112310;product=serine/threonine-protein kinase D6PK-like;protein_id=XP_044389196.1

GO:0005737; Cytoplasm.

Kozak: CCAATGC ID=cds-XP_044389216.1;Parent=rna-XM_044533281.1;Dbxref=GeneID:123112321,Genbank:XP_044389216.1;Name=XP_044389216.1;gbkey=CDS;gene=LOC123112321;product=leucine-rich repeat receptor-like serine/threonine-protein kinase RGI4;protein_id=XP_044389216.1

GO:0016021; Integral component of membrane.

Kozak: CAGATGC ID=cds-XP_044389312.1;Parent=rna-XM_044533377.1;Dbxref=GeneID:123112400,Genbank:XP_044389312.1;Name=XP_044389312.1;gbkey=CDS;gene=LOC123112400;product=

OVARIAN TUMOR DOMAIN-containing deubiquitinating enzyme 4-like.

Kozak: CAGATGC ID=cds-XP_044389582.1;Parent=rna-XM_044533647.1;Dbxref=GeneID:123112614,Genbank:XP_044389582.1;Name=XP_044389582.1;gbkey=CDS;gene=LOC123112614;product=probable UDP-arabinose 4-epimerase 1 isoform X1;protein_id=XP_044389582.1

GO:0003978; UDP-glucose 4-epimerase activity.

Kozak: CCCATGC ID=cds-XP_044389600.1;Parent=rna-XM_044533665.1;Dbxref=GeneID:123112623,Genbank:XP_044389600.1;Name=XP_044389600.1;gbkey=CDS;gene=LOC123112623;product=

Caffeoylshikimate esterase-like.

Kozak: TTCATGT ID=cds-XP_044389785.1;Parent=rna-XM_044533850.1;Dbxref=GeneID:123112765,Genbank:XP_044389785.1;Name=XP_044389785.1;gbkey=CDS;gene=LOC123112765;product=glucuronoxylan 4-O-methyltransferase 1-like;protein_id=XP_044389785.1

GO:0005794; Golgi apparatus.

Kozak: TACATGC ID=cds-XP_044389945.1;Parent=rna-XM_044534010.1;Dbxref=GeneID:123112912,Genbank:XP_044389945.1;Name=XP_044389945.1;gbkey=CDS;gene=LOC123112912;product=ABC transporter C family member 2-like isoform X1;protein_id=XP_044389945.1

GO:0016021; Integral component of membrane.

Kozak: CCCATGC ID=cds-XP_044390231.1;Parent=rna-XM_044534296.1;Dbxref=GeneID:123113145,Genbank:XP_044390231.1;Name=XP_044390231.1;gbkey=CDS;gene=LOC123113145;product=G-type lectin S-receptor-like serine/threonine-protein kinase At2g19130;protein_id=XP_044390231.1

GO:0016021; Integral component of membrane.

Kozak: CAGATGC ID=cds-XP_044390300.1;Parent=rna-XM_044534365.1;Dbxref=GeneID:123113194,Genbank:XP_044390300.1;Name=XP_044390300.1;gbkey=CDS;gene=LOC123113194;product=9-cis-epoxycarotenoid dioxygenase NCED3%2C chloroplastic-like;protein_id=XP_044390300.1

GO:0009570; Chloroplast stroma.

Kozak: CAGATGC ID=cds-XP_044390492.1;Parent=rna-XM_044534557.1;Dbxref=GeneID:123113345,Genbank:XP_044390492.1;Name=XP_044390492.1;gbkey=CDS;gene=LOC123113345;product=protein PYRICULARIA ORYZAE RESISTANCE 21-like;protein_id=XP_044390492.1

GO:0046872; Metal ion binding.

Kozak: CCAATGC ID=cds-XP_044390631.1;Parent=rna-XM_044534696.1;Dbxref=GeneID:123113464,Genbank:XP_044390631.1;Name=XP_044390631.1;gbkey=CDS;gene=LOC123113464;product=leucine-rich repeat receptor-like serine/threonine-protein kinase BAM1;protein_id=XP_044390631.1

GO:0016021; Integral component of membrane.

Kozak: TCCATGT ID=cds-XP_044390794.1;Parent=rna-XM_044534859.1;Dbxref=GeneID:123113574,Genbank:XP_044390794.1;Name=XP_044390794.1;gbkey=CDS;gene=LOC123113574;product=zinc finger CCCH domain-containing protein 16-like isoform X1;protein_id=XP_044390794.1

GO:0005681; Spliceosome complex.

Kozak: TTCATGT ID=cds-XP_044390811.1;Parent=rna-XM_044534876.1;Dbxref=GeneID:123113586,Genbank:XP_044390811.1;Name=XP_044390811.1;gbkey=CDS;gene=LOC123113586;product=cullin-4-like;protein_id=XP_044390811.1

GO:0031461; Cullin-RING ubiquitin ligase complex.

Kozak: CCCATGT ID=cds-XP_044390980.1;Parent=rna-XM_044535045.1;Dbxref=GeneID:123113738,Genbank:XP_044390980.1;Name=XP_044390980.1;gbkey=CDS;gene=LOC123113738;product=chromatin remodeling protein SHL-like isoform X1;protein_id=XP_044390980.1

GO:0005615; Extracellular space.

Kozak: CAGATGC ID=cds-XP_044391030.1;Parent=rna-XM_044535095.1;Dbxref=GeneID:123113783,Genbank:XP_044391030.1;Name=XP_044391030.1;gbkey=CDS;gene=LOC123113783;product=LEAF RUST 10 DISEASE-RESISTANCE LOCUS RECEPTOR-LIKE PROTEIN KINASE-like 1.5;protein_id=XP_044391030.1

GO:0016021; Integral component of membrane.

Kozak: TCCATGT ID=cds-XP_044391104.1;Parent=rna-XM_044535169.1;Dbxref=GeneID:123113852,Genbank:XP_044391104.1;Name=XP_044391104.1;gbkey=CDS;gene=LOC123113852;product=protein argonaute 12-like;protein_id=XP_044391104.1

GO:0005737; Cytoplasm.

Kozak: TCCATGT ID=cds-XP_044391349.1;Parent=rna-XM_044535414.1;Dbxref=GeneID:123114054,Genbank:XP_044391349.1;Name=XP_044391349.1;gbkey=CDS;gene=LOC123114054;product=methyltransferase N6AMT1-like;protein_id=XP_044391349.1

GO:0035657; eRF1 methyltransferase complex.

Kozak: CAGATGC ID=cds-XP_044391359.1;Parent=rna-XM_044535424.1;Dbxref=GeneID:123114062,Genbank:XP_044391359.1;Name=XP_044391359.1;gbkey=CDS;gene=LOC123114062;product=protein DEFECTIVE IN EXINE FORMATION 1-like;protein_id=XP_044391359.1

GO:0016021; Integral component of membrane.

Kozak: TCCATGT ID=cds-XP_044391366.1;Parent=rna-XM_044535431.1;Dbxref=GeneID:123114065,Genbank:XP_044391366.1;Name=XP_044391366.1;gbkey=CDS;gene=LOC123114065;product=GDP-mannose transporter GONST1-like;protein_id=XP_044391366.1

GO:0005794; Golgi apparatus.

Kozak: CCAATGC ID=cds-XP_044391500.1;Parent=rna-XM_044535565.1;Dbxref=GeneID:123114181,Genbank:XP_044391500.1;Name=XP_044391500.1;gbkey=CDS;gene=LOC123114181;product=IAA-amino acid hydrolase ILR1-like 4;protein_id=XP_044391500.1

GO:0016787; Hydrolase activity.

Kozak: CCAATGT ID=cds-XP_044391536.1;Parent=rna-XM_044535601.1;Dbxref=GeneID:123114217,Genbank:XP_044391536.1;Name=XP_044391536.1;gbkey=CDS;gene=LOC123114217;product=

ATP-dependent zinc metalloprotease FTSH 5%2C mitochondrial-like.

Kozak: CCAATGC ID=cds-XP_044391621.1;Parent=rna-XM_044535686.1;Dbxref=GeneID:123114269,Genbank:XP_044391621.1;Name=XP_044391621.1;gbkey=CDS;gene=LOC123114269;product=30S ribosomal protein S6 alpha%2C chloroplastic-like;protein_id=XP_044391621.1

GO:0005737; Cytoplasm.

Kozak: TGTATGT ID=cds-XP_044391822.1;Parent=rna-XM_044535887.1;Dbxref=GeneID:123114424,Genbank:XP_044391822.1;Name=XP_044391822.1;gbkey=CDS;gene=LOC123114424;product=beta-fructofuranosidase%2C insoluble isoenzyme 4-like;protein_id=XP_044391822.1

GO:0016021; Integral component of membrane.

Kozak: TCCATGT ID=cds-XP_044392209.1;Parent=rna-XM_044536274.1;Dbxref=GeneID:123114903,Genbank:XP_044392209.1;Name=XP_044392209.1;gbkey=CDS;gene=LOC123114903;product=mitochondrial import inner membrane translocase subunit TIM8-like;protein_id=XP_044392209.1

GO:0005743; Mitochondrial inner membrane.

Kozak: CCCATGT ID=cds-XP_044392233.1;Parent=rna-XM_044536298.1;Dbxref=GeneID:123114945,Genbank:XP_044392233.1;Name=XP_044392233.1;gbkey=CDS;gene=LOC123114945;product=

GDSL esterase/lipase EXL3-like.

Kozak: CCCATGT ID=cds-XP_044392895.1;Parent=rna-XM_044536960.1;Dbxref=GeneID:123115917,Genbank:XP_044392895.1;Name=XP_044392895.1;gbkey=CDS;gene=LOC123115917;product=uncharacterized protein LOC123115917;protein_id=XP_044392895.1

GO:0005634; Nucleus.

Kozak: TCCATGT ID=cds-XP_044392952.1;Parent=rna-XM_044537017.1;Dbxref=GeneID:123115995,Genbank:XP_044392952.1;Name=XP_044392952.1;gbkey=CDS;gene=LOC123115995;product=probable phytol kinase 2%2C chloroplastic isoform X1;protein_id=XP_044392952.1

GO:0031969; Chloroplast membrane.

Kozak: CCAATGT ID=cds-XP_044393092.1;Parent=rna-XM_044537157.1;Dbxref=GeneID:123116158,Genbank:XP_044393092.1;Name=XP_044393092.1;gbkey=CDS;gene=LOC123116158;product=transcription factor TB1-like;protein_id=XP_044393092.1

GO:0005634; Nucleus.

Kozak: CCCATGC ID=cds-XP_044393115.1;Parent=rna-XM_044537180.1;Dbxref=GeneID:123116180,Genbank:XP_044393115.1;Name=XP_044393115.1;gbkey=CDS;gene=LOC123116180;product=cytoplasmic tRNA 2-thiolation protein 1;protein_id=XP_044393115.1

GO:0002144; Cytosolic tRNA wobble base thiouridylase complex

Kozak: CCAATGT ID=cds-XP_044393197.1;Parent=rna-XM_044537262.1;Dbxref=GeneID:123116281,Genbank:XP_044393197.1;Name=XP_044393197.1;gbkey=CDS;gene=LOC123116281;product=polcalcin Ole e 3-like;protein_id=XP_044393197.1

GO:0005509; Calcium ion binding.

Kozak: CCCATGC ID=cds-XP_044393261.1;Parent=rna-XM_044537326.1;Dbxref=GeneID:123116362,Genbank:XP_044393261.1;Name=XP_044393261.1;gbkey=CDS;gene=LOC123116362;product=

BURP domain-containing protein 15-like.

Kozak: CCCATGC ID=cds-XP_044393466.1;Parent=rna-XM_044537531.1;Dbxref=GeneID:123116592,Genbank:XP_044393466.1;Name=XP_044393466.1;gbkey=CDS;gene=LOC123116592;product=cytochrome P450 87A3-like;protein_id=XP_044393466.1

GO:0016021; integral component of membrane.

Kozak: TCCATGT ID=cds-XP_044393501.1;Parent=rna-XM_044537566.1;Dbxref=GeneID:123116625,Genbank:XP_044393501.1;Name=XP_044393501.1;gbkey=CDS;gene=LOC123116625;product=ethylene-responsive transcription factor ERF071-like;protein_id=XP_044393501.1

GO:0005634; Nucleus

Kozak: TACATGC ID=cds-XP_044394036.1;Parent=rna-XM_044538101.1;Dbxref=GeneID:123117325,Genbank:XP_044394036.1;Name=XP_044394036.1;gbkey=CDS;gene=LOC123117325;product=

PLASMODESMATA CALLOSE-BINDING PROTEIN 3-like isoform X1.

Kozak: TCCATGT ID=cds-XP_044394268.1;Parent=rna-XM_044538333.1;Dbxref=GeneID:123117614,Genbank:XP_044394268.1;Name=XP_044394268.1;gbkey=CDS;gene=LOC123117614;product=uncharacterized protein LOC123117614;protein_id=XP_044394268.1

GO:0005829; Cytosol.

Kozak: TCCATGT ID=cds-XP_044394441.1;Parent=rna-XM_044538506.1;Dbxref=GeneID:123117833,Genbank:XP_044394441.1;Name=XP_044394441.1;gbkey=CDS;gene=LOC123117833;product=

Ethylene-response factor C3-like.

Kozak: CAGATGC ID=cds-XP_044386424.1;Parent=rna-XM_044530489.1;Dbxref=GeneID:123110069,Genbank:XP_044386424.1;Name=XP_044386424.1;gbkey=CDS;gene=LOC123110069;product=

Putative disease resistance protein At3g14460.

Kozak: CCAATGC ID=cds-XP_044386612.1;Parent=rna-XM_044530677.1;Dbxref=GeneID:123110203,Genbank:XP_044386612.1;Name=XP_044386612.1;gbkey=CDS;gene=LOC123110203;product=ubiquitin carboxyl-terminal hydrolase 8-like;protein_id=XP_044386612.1

GO:0004843; Thiol-dependent deubiquitinase.

Kozak: CCCATGC ID=cds-XP_044386707.1;Parent=rna-XM_044530772.1;Dbxref=GeneID:123110290,Genbank:XP_044386707.1;Name=XP_044386707.1;gbkey=CDS;gene=LOC123110290;product=

Lysine-rich arabinogalactan protein 19-like isoform X1.

Kozak: CCAATGC ID=cds-XP_044386808.1;Parent=rna-XM_044530873.1;Dbxref=GeneID:123110379,Genbank:XP_044386808.1;Name=XP_044386808.1;gbkey=CDS;gene=LOC123110379;product=importin subunit beta-1-like;protein_id=XP_044386808.1

GO:0005737; Cytoplasm.

Kozak: CAGATGC ID=cds-XP_044387102.1;Parent=rna-XM_044531167.1;Dbxref=GeneID:123110611,Genbank:XP_044387102.1;Name=XP_044387102.1;gbkey=CDS;gene=LOC123110611;product=polyubiquitin isoform X1;protein_id=XP_044387102.1

GO:0005737; Cytoplasm.

Kozak: CCCATGT ID=cds-XP_044387246.1;Parent=rna-XM_044531311.1;Dbxref=GeneID:123110721,Genbank:XP_044387246.1;Name=XP_044387246.1;gbkey=CDS;gene=LOC123110721;product=uncharacterized protein LOC123110721;protein_id=XP_044387246.1

GO:0000178; Exosome (RNase complex).

Kozak: TCCATGT ID=cds-XP_044387408.1;Parent=rna-XM_044531473.1;Dbxref=GeneID:123110848,Genbank:XP_044387408.1;Name=XP_044387408.1;gbkey=CDS;gene=LOC123110848;product=AP-5 complex subunit mu-like;protein_id=XP_044387408.1

GO:0030119; AP-type membrane coat adaptor complex.

Kozak: TACATGC ID=cds-XP_044387440.1;Parent=rna-XM_044531505.1;Dbxref=GeneID:123110874,Genbank:XP_044387440.1;Name=XP_044387440.1;gbkey=CDS;gene=LOC123110874;product=protein CLT2%2C chloroplastic-like;protein_id=XP_044387440.1

GO:0016021; Integral component of membrane.

Kozak: TCCATGT ID=cds-XP_044387509.1;Parent=rna-XM_044531574.1;Dbxref=GeneID:123110932,Genbank:XP_044387509.1;Name=XP_044387509.1;gbkey=CDS;gene=LOC123110932;product=protein transport protein Sec24-like CEF isoform X1;protein_id=XP_044387509.1

GO:0030127; COPII vesicle coat.

Kozak: CCAATGC ID=cds-XP_044387591.1;Parent=rna-XM_044531656.1;Dbxref=GeneID:123111001,Genbank:XP_044387591.1;Name=XP_044387591.1;gbkey=CDS;gene=LOC123111001;product=calcium-dependent protein kinase 28-like;protein_id=XP_044387591.1

GO:0005739; Mitochondrion.

Kozak: TTCATGT ID=cds-XP_044387913.1;Parent=rna-XM_044531978.1;Dbxref=GeneID:123111233,Genbank:XP_044387913.1;Name=XP_044387913.1;gbkey=CDS;gene=LOC123111233;product=

RING finger protein 10-like.

Kozak: TACATGC ID=cds-XP_044387978.1;Parent=rna-XM_044532043.1;Dbxref=GeneID:123111288,Genbank:XP_044387978.1;Name=XP_044387978.1;gbkey=CDS;gene=LOC123111288;product=cysteine protease XCP1-like;protein_id=XP_044387978.1

GO:0005615; Extracellular space.

Kozak: CCAATGT ID=cds-XP_044388174.1;Parent=rna-XM_044532239.1;Dbxref=GeneID:123111438,Genbank:XP_044388174.1;Name=XP_044388174.1;gbkey=CDS;gene=LOC123111438;product=uncharacterized protein LOC123111438 isoform X1;protein_id=XP_044388174.1

GO:0030014; CCR4-NOT complex.

Kozak: TCCATGT ID=cds-XP_044388224.1;Parent=rna-XM_044532289.1;Dbxref=GeneID:123111487,Genbank:XP_044388224.1;Name=XP_044388224.1;gbkey=CDS;gene=LOC123111487;product=receptor like protein kinase S.2-like;protein_id=XP_044388224.1

GO:0005886; Plasma membrane.

Kozak: CCCATGC ID=cds-XP_044389164.1;Parent=rna-XM_044533229.1;Dbxref=GeneID:123112280,Genbank:XP_044389164.1;Name=XP_044389164.1;gbkey=CDS;gene=LOC123112280;product=uncharacterized protein LOC123112280 isoform X1;protein_id=XP_044389164.1

GO:0005634; Nucleus.

Kozak: TCCATGT ID=cds-XP_044390091.1;Parent=rna-XM_044534156.1;Dbxref=GeneID:123113048,Genbank:XP_044390091.1;Name=XP_044390091.1;gbkey=CDS;gene=LOC123113048;product=F-box/kelch-repeat protein At5g42350-like;protein_id=XP_044390091.1

GO:0019005; SCF ubiquitin ligase complex.

Kozak: CCCATGT ID=cds-XP_044390372.1;Parent=rna-XM_044534437.1;Dbxref=GeneID:123113244,Genbank:XP_044390372.1;Name=XP_044390372.1;gbkey=CDS;gene=LOC123113244;product=F-box/LRR-repeat protein 17-like isoform X1;protein_id=XP_044390372.1

GO:0005737; Cytoplasm.

Kozak: CCAATGC ID=cds-XP_044390434.1;Parent=rna-XM_044534499.1;Dbxref=GeneID:123113304,Genbank:XP_044390434.1;Name=XP_044390434.1;gbkey=CDS;gene=LOC123113304;product=photosystem II D1 precursor processing protein PSB27-H2%2C chloroplastic-like;protein_id=XP_044390434.1

GO:0009543; Chloroplast thylakoid lumen.

Kozak: CCAATGT ID=cds-XP_044390496.1;Parent=rna-XM_044534561.1;Dbxref=GeneID:123113349,Genbank:XP_044390496.1;Name=XP_044390496.1;gbkey=CDS;gene=LOC123113349;product=two pore potassium channel a-like;protein_id=XP_044390496.1

GO:0005887; integral component of plasma membrane.

Kozak: CCCATGT ID=cds-XP_044390608.1;Parent=rna-XM_044534673.1;Dbxref=GeneID:123113445,Genbank:XP_044390608.1;Name=XP_044390608.1;gbkey=CDS;gene=LOC123113445;product=probable hexosyltransferase MUCI70;protein_id=XP_044390608.1

GO:0016021; integral component of membrane.

Kozak: CCCATGC ID=cds-XP_044390629.1;Parent=rna-XM_044534694.1;Dbxref=GeneID:123113462,Genbank:XP_044390629.1;Name=XP_044390629.1;gbkey=CDS;gene=LOC123113462;product=probable LRR receptor-like serine/threonine-protein kinase At2g16250;protein_id=XP_044390629.1

GO:0016021; integral component of membrane.

Kozak: TTAATGC ID=cds-XP_044391196.1;Parent=rna-XM_044535261.1;Dbxref=GeneID:123113930,Genbank:XP_044391196.1;Name=XP_044391196.1;gbkey=CDS;gene=LOC123113930;product=serine/threonine/tyrosine-protein kinase HT1-like;protein_id=XP_044391196.1

GO:0005737; Cytoplasm.

Kozak: TCCATGT ID=cds-XP_044391888.1;Parent=rna-XM_044535953.1;Dbxref=GeneID:123114465,Genbank:XP_044391888.1;Name=XP_044391888.1;gbkey=CDS;gene=LOC123114465;product=GDSL esterase/lipase At3g14820-like;protein_id=XP_044391888.1

GO:0016788; Hydrolase activity, acting on ester bonds.

Kozak: TTCATGT ID=cds-XP_044392376.1;Parent=rna-XM_044536441.1;Dbxref=GeneID:123115216,Genbank:XP_044392376.1;Name=XP_044392376.1;gbkey=CDS;gene=LOC123115216;product=

Protein FAR1-RELATED SEQUENCE 5-like.

Kozak: CCAATGT ID=cds-XP_044392457.1;Parent=rna-XM_044536522.1;Dbxref=GeneID:123115352,Genbank:XP_044392457.1;Name=XP_044392457.1;gbkey=CDS;gene=LOC123115352;product=

Chaperone protein ClpB1-like.

Kozak: TCCATGT ID=cds-XP_044392867.1;Parent=rna-XM_044536932.1;Dbxref=GeneID:123115856,Genbank:XP_044392867.1;Name=XP_044392867.1;gbkey=CDS;gene=LOC123115856;product=NDR1/HIN1-like protein 26;protein_id=XP_044392867.1

GO:0046658; Anchored component of plasma membrane.

Kozak: CCCATGC ID=cds-XP_044392975.1;Parent=rna-XM_044537040.1;Dbxref=GeneID:123116027,Genbank:XP_044392975.1;Name=XP_044392975.1;gbkey=CDS;gene=LOC123116027;product=

Formin-like protein 14.

Kozak: TCCATGT ID=cds-XP_044393212.1;Parent=rna-XM_044537277.1;Dbxref=GeneID:123116299,Genbank:XP_044393212.1;Name=XP_044393212.1;gbkey=CDS;gene=LOC123116299;product=serine carboxypeptidase 1-like;protein_id=XP_044393212.1

GO:0004185; Serine-type carboxypeptidase activity.

Kozak: TCCATGT ID=cds-XP_044393214.1;Parent=rna-XM_044537279.1;Dbxref=GeneID:123116301,Genbank:XP_044393214.1;Name=XP_044393214.1;gbkey=CDS;gene=LOC123116301;product=AT-hook motif nuclear-localized protein 17-like;protein_id=XP_044393214.1

GO:0016021; Integral component of membrane.

Kozak: CCCATGT ID=cds-XP_044393232.1;Parent=rna-XM_044537297.1;Dbxref=GeneID:123116330,Genbank:XP_044393232.1;Name=XP_044393232.1;gbkey=CDS;gene=LOC123116330;product=uncharacterized protein LOC123116330;protein_id=XP_044393232.1

GO:0016021; Integral component of membrane.

Kozak: CCCATGC ID=cds-XP_044393671.1;Parent=rna-XM_044537736.1;Dbxref=GeneID:123116846,Genbank:XP_044393671.1;Name=XP_044393671.1;gbkey=CDS;gene=LOC123116846;product=carboxyl-terminal-processing peptidase 2%2C chloroplastic-like;protein_id=XP_044393671.1

GO:0004175; Endopeptidase activity.

Kozak: TCCATGT ID=cds-XP_044394041.1;Parent=rna-XM_044538106.1;Dbxref=GeneID:123117336,Genbank:XP_044394041.1;Name=XP_044394041.1;gbkey=CDS;gene=LOC123117336;product=

F-box/kelch-repeat protein At4g05080-like.

Kozak: CAGATGC ID=cds-XP_044394461.1;Parent=rna-XM_044538526.1;Dbxref=GeneID:123117858,Genbank:XP_044394461.1;Name=XP_044394461.1;gbkey=CDS;gene=LOC123117858;product=E3 ubiquitin-protein ligase RNF181-like;protein_id=XP_044394461.1

GO:0044260; Cellular macromolecule metabolic process.

Kozak: CCCATGC ID=cds-XP_044389513.1;Parent=rna-XM_044533578.1;Dbxref=GeneID:543005,Genbank:XP_044389513.1;Name=XP_044389513.1;gbkey=CDS;gene=LOC543005;product=oleoyl-acyl carrier protein thioesterase 1%2C chloroplastic;protein_id=XP_044389513.1

GO:0009507; Chloroplast.

Kozak: TCCATGT ID=cds-XP_044318040.1;Parent=rna-XM_044462105.1;Dbxref=GeneID:123038215,Genbank:XP_044318040.1;Name=XP_044318040.1;gbkey=CDS;gene=LOC123038215;product=exocyst complex component EXO70A1-like;protein_id=XP_044318040.1

GO:0000145; Exocyst.

Kozak: TCCATGT ID=cds-XP_044318041.1;Parent=rna-XM_044462106.1;Dbxref=GeneID:123038216,Genbank:XP_044318041.1;Name=XP_044318041.1;gbkey=CDS;gene=LOC123038216;product=exocyst complex component EXO70A1-like;protein_id=XP_044318041.1

GO:0000145; Exocyst.

Kozak: TGTATGT ID=cds-XP_044452298.1;Parent=rna-XM_044596363.1;Dbxref=GeneID:123184205,Genbank:XP_044452298.1;Name=XP_044452298.1;gbkey=CDS;gene=LOC123184205;product=cytosolic sulfotransferase 5-like;protein_id=XP_044452298.1

GO:0005737; Cytoplasm.

Kozak: TGTATGT ID=cds-XP_044452299.1;Parent=rna-XM_044596364.1;Dbxref=GeneID:123184206,Genbank:XP_044452299.1;Name=XP_044452299.1;gbkey=CDS;gene=LOC123184206;product=cytosolic sulfotransferase 5-like;protein_id=XP_044452299.1

GO:0005737; Cytoplasm.

Kozak: TGTATGT ID=cds-XP_044452300.1;Parent=rna-XM_044596365.1;Dbxref=GeneID:123184207,Genbank:XP_044452300.1;Name=XP_044452300.1;gbkey=CDS;gene=LOC123184207;product=cytosolic sulfotransferase 5-like;protein_id=XP_044452300.1

GO:0005737; Cytoplasm

Kozak: TGTATGT ID=cds-XP_044452667.1;Parent=rna-XM_044596732.1;Dbxref=GeneID:123184644,Genbank:XP_044452667.1;Name=XP_044452667.1;gbkey=CDS;gene=LOC123184644;product=uncharacterized protein LOC123184644;protein_id=XP_044452667.1

GO:0030598; rRNA N-glycosylase activity.

Kozak: TACATGC ID=cds-XP_044452822.1;Parent=rna-XM_044596887.1;Dbxref=GeneID:123184840,Genbank:XP_044452822.1;Name=XP_044452822.1;gbkey=CDS;gene=LOC123184840;product=zinc finger A20 and AN1 domain-containing stress-associated protein 12-like;protein_id=XP_044452822.1

GO:0003677; DNA binding.

Kozak: CCCATGC ID=cds-XP_044452983.1;Parent=rna-XM_044597048.1;Dbxref=GeneID:123185027,Genbank:XP_044452983.1;Name=XP_044452983.1;gbkey=CDS;gene=LOC123185027;product=

Protein PELPK1-like.

Kozak: CCAATGC ID=cds-XP_044453310.1;Parent=rna-XM_044597375.1;Dbxref=GeneID:123185498,Genbank:XP_044453310.1;Name=XP_044453310.1;gbkey=CDS;gene=LOC123185498;product=uncharacterized protein LOC123185498;protein_id=XP_044453310.1

GO:0016021; Integral component of membrane

Kozak: CCAATGC ID=cds-XP_044453575.1;Parent=rna-XM_044597640.1;Dbxref=GeneID:123185813,Genbank:XP_044453575.1;Name=XP_044453575.1;gbkey=CDS;gene=LOC123185813;product=xyloglucan galactosyltransferase XLT2-like;protein_id=XP_044453575.1

GO:0005794; Golgi apparatus.

Kozak: CAGATGC ID=cds-XP_044454118.1;Parent=rna-XM_044598183.1;Dbxref=GeneID:123186416,Genbank:XP_044454118.1;Name=XP_044454118.1;gbkey=CDS;gene=LOC123186416;product=

Bifunctional epoxide hydrolase 2-like.

Kozak: CCCATGT ID=cds-XP_044454868.1;Parent=rna-XM_044598933.1;Dbxref=GeneID:123187161,Genbank:XP_044454868.1;Name=XP_044454868.1;gbkey=CDS;gene=LOC123187161;product=peptidyl-prolyl cis-trans isomerase FKBP19%2C chloroplastic-like;protein_id=XP_044454868.1

GO:0003755; Peptidyl-prolyl cis-trans isomerase activity.

Kozak: TTCATGT ID=cds-XP_044455011.1;Parent=rna-XM_044599076.1;Dbxref=GeneID:123187262,Genbank:XP_044455011.1;Name=XP_044455011.1;gbkey=CDS;gene=LOC123187262;product=wall-associated receptor kinase 2-like;protein_id=XP_044455011.1

GO:0016021; Integral component of membrane.

Kozak: CCCATGT ID=cds-XP_044455229.1;Parent=rna-XM_044599294.1;Dbxref=GeneID:123187425,Genbank:XP_044455229.1;Name=XP_044455229.1;gbkey=CDS;gene=LOC123187425;product=RINT1-like protein MAG2L;protein_id=XP_044455229.1

GO:0070939; Dsl1/NZR complex.

Kozak: TACATGC ID=cds-XP_044455252.1;Parent=rna-XM_044599317.1;Dbxref=GeneID:123187447,Genbank:XP_044455252.1;Name=XP_044455252.1;gbkey=CDS;gene=LOC123187447;product=NAC domain-containing protein 2-like;protein_id=XP_044455252.1

GO:0005634; Nucleus.

Kozak: CCCATGT ID=cds-XP_044455254.1;Parent=rna-XM_044599319.1;Dbxref=GeneID:123187449,Genbank:XP_044455254.1;Name=XP_044455254.1;gbkey=CDS;gene=LOC123187449;product=

Myb-related transcription factor%2C partner of profilin-like.

Kozak: CCAATGC ID=cds-XP_044455506.1;Parent=rna-XM_044599571.1;Dbxref=GeneID:123187651,Genbank:XP_044455506.1;Name=XP_044455506.1;gbkey=CDS;gene=LOC123187651;product=phosphatidylinositol 4-phosphate 5-kinase 6-like;protein_id=XP_044455506.1

GO:0005886; Plasma membrane.

Kozak: CCCATGC ID=cds-XP_044455775.1;Parent=rna-XM_044599840.1;Dbxref=GeneID:123187872,Genbank:XP_044455775.1;Name=XP_044455775.1;gbkey=CDS;gene=LOC123187872;product=15-cis-phytoene desaturase%2C chloroplastic/chromoplastic;protein_id=XP_044455775.1

GO:0110165; Cellular anatomical entity

Kozak: TACATGC ID=cds-XP_044455776.1;Parent=rna-XM_044599841.1;Dbxref=GeneID:123187873,Genbank:XP_044455776.1;Name=XP_044455776.1;gbkey=CDS;gene=LOC123187873;product=putative MYST-like histone acetyltransferase 1;protein_id=XP_044455776.1

GO:0004402; Histone acetyltransferase activity.

Kozak: CGAATGT ID=cds-XP_044456047.1;Parent=rna-XM_044600112.1;Dbxref=GeneID:123188078,Genbank:XP_044456047.1;Name=XP_044456047.1;gbkey=CDS;gene=LOC123188078;product=zinc finger SWIM domain-containing protein 7-like;protein_id=XP_044456047.1

GO:0097196; Shu complex.

Kozak: TACATGC ID=cds-XP_044456172.1;Parent=rna-XM_044600237.1;Dbxref=GeneID:123188199,Genbank:XP_044456172.1;Name=XP_044456172.1;gbkey=CDS;gene=LOC123188199;product=

Translation initiation factor IF-2-like.

Kozak: TTCATGT ID=cds-XP_044456426.1;Parent=rna-XM_044600491.1;Dbxref=GeneID:123188402,Genbank:XP_044456426.1;Name=XP_044456426.1;gbkey=CDS;gene=LOC123188402;product=protein translation factor SUI1 homolog;protein_id=XP_044456426.1

GO:0003723; RNA binding.

Kozak: CCCATGT ID=cds-XP_044456584.1;Parent=rna-XM_044600649.1;Dbxref=GeneID:123188500,Genbank:XP_044456584.1;Name=XP_044456584.1;gbkey=CDS;gene=LOC123188500;product=rRNA-processing protein UTP23 homolog;protein_id=XP_044456584.1

GO:0005730; Nucleolus

Kozak: CCCATGC ID=cds-XP_044456789.1;Parent=rna-XM_044600854.1;Dbxref=GeneID:123188644,Genbank:XP_044456789.1;Name=XP_044456789.1;gbkey=CDS;gene=LOC123188644;product=

Translation initiation factor IF-2-like.

Kozak: CGAATGT ID=cds-XP_044457261.1;Parent=rna-XM_044601326.1;Dbxref=GeneID:123189009,Genbank:XP_044457261.1;Name=XP_044457261.1;gbkey=CDS;gene=LOC123189009;product=uncharacterized protein LOC123189009;protein_id=XP_044457261.1

GO:0016021; Integral component of membrane.

Kozak: CCCATGT ID=cds-XP_044457389.1;Parent=rna-XM_044601454.1;Dbxref=GeneID:123189117,Genbank:XP_044457389.1;Name=XP_044457389.1;gbkey=CDS;gene=LOC123189117;product=uncharacterized protein At1g32220%2C chloroplastic-like;protein_id=XP_044457389.1

GO:0006952; Defense response.

Kozak: CCAATGT ID=cds-XP_044457598.1;Parent=rna-XM_044601663.1;Dbxref=GeneID:123189283,Genbank:XP_044457598.1;Name=XP_044457598.1;gbkey=CDS;gene=LOC123189283;product=serine/threonine-protein kinase-like protein CCR4;protein_id=XP_044457598.1

GO:0016021; Integral component of membrane.

Kozak: TCCATGT ID=cds-XP_044457634.1;Parent=rna-XM_044601699.1;Dbxref=GeneID:123189310,Genbank:XP_044457634.1;Name=XP_044457634.1;gbkey=CDS;gene=LOC123189310;product=uncharacterized protein LOC123189310 isoform X2;protein_id=XP_044457634.1

GO:0034991; Nuclear meiotic cohesin complex.

Kozak: CGAATGT ID=cds-XP_044458237.1;Parent=rna-XM_044602302.1;Dbxref=GeneID:123189808,Genbank:XP_044458237.1;Name=XP_044458237.1;gbkey=CDS;gene=LOC123189808;product=probable beta-D-xylosidase 6;protein_id=XP_044458237.1

GO:0046556; Alpha-L-arabinofuranosidase activity.

Kozak: CAGATGC ID=cds-XP_044458327.1;Parent=rna-XM_044602392.1;Dbxref=GeneID:123189874,Genbank:XP_044458327.1;Name=XP_044458327.1;gbkey=CDS;gene=LOC123189874;product=pentatricopeptide repeat-containing protein At3g53360%2C mitochondrial-like;protein_id=XP_044458327.1

GO:0003723; RNA binding.

Kozak: CAGATGC ID=cds-XP_044458516.1;Parent=rna-XM_044602581.1;Dbxref=GeneID:123190035,Genbank:XP_044458516.1;Name=XP_044458516.1;gbkey=CDS;gene=LOC123190035;product=homeobox-leucine zipper protein ROC4-like;protein_id=XP_044458516.1

GO:0005615; Extracellular space.

Kozak: CCCATGT ID=cds-XP_044458547.1;Parent=rna-XM_044602612.1;Dbxref=GeneID:123190051,Genbank:XP_044458547.1;Name=XP_044458547.1;gbkey=CDS;gene=LOC123190051;product=uncharacterized protein LOC123190051;protein_id=XP_044458547.1

GO:0005730; Nucleolus.

Kozak: CAGATGC ID=cds-XP_044458579.1;Parent=rna-XM_044602644.1;Dbxref=GeneID:123190077,Genbank:XP_044458579.1;Name=XP_044458579.1;gbkey=CDS;gene=LOC123190077;product=lysine-specific histone demethylase 1 homolog 3-like;protein_id=XP_044458579.1

GO:0110165; Cellular anatomical entity.

Kozak: TACATGC ID=cds-XP_044459099.1;Parent=rna-XM_044603164.1;Dbxref=GeneID:123190510,Genbank:XP_044459099.1;Name=XP_044459099.1;gbkey=CDS;gene=LOC123190510;product=

Putative disease resistance protein RGA4.

Kozak: CCCATGT ID=cds-XP_044459516.1;Parent=rna-XM_044603581.1;Dbxref=GeneID:123190849,Genbank:XP_044459516.1;Name=XP_044459516.1;gbkey=CDS;gene=LOC123190849;product=phenolic glucoside malonyltransferase 2-like;protein_id=XP_044459516.1

GO:0016747; Acyltransferase activity.

Kozak: CAGATGC ID=cds-XP_044459531.1;Parent=rna-XM_044603596.1;Dbxref=GeneID:123190862,Genbank:XP_044459531.1;Name=XP_044459531.1;gbkey=CDS;gene=LOC123190862;product=otolith matrix protein OMM-64-like;protein_id=XP_044459531.1

GO:0016021; Integral component of membrane.

Kozak: CCCATGT ID=cds-XP_044460128.1;Parent=rna-XM_044604193.1;Dbxref=GeneID:123191467,Genbank:XP_044460128.1;Name=XP_044460128.1;gbkey=CDS;gene=LOC123191467;product=

Leucine-rich repeat receptor-like protein kinase PEPR2.

Kozak: CCCATGC ID=cds-XP_044460238.1;Parent=rna-XM_044604303.1;Dbxref=GeneID:123191642,Genbank:XP_044460238.1;Name=XP_044460238.1;gbkey=CDS;gene=LOC123191642;product=

Wiskott-Aldrich syndrome protein homolog 1-like.

Kozak: CCCATGC ID=cds-XP_044460278.1;Parent=rna-XM_044604343.1;Dbxref=GeneID:123191702,Genbank:XP_044460278.1;Name=XP_044460278.1;gbkey=CDS;gene=LOC123191702;product=

Serine/arginine repetitive matrix protein 2-like.

Kozak: CAGATGC ID=cds-XP_044452945.1;Parent=rna-XM_044597010.1;Dbxref=GeneID:123184987,Genbank:XP_044452945.1;Name=XP_044452945.1;gbkey=CDS;gene=LOC123184987;product=

Probable inactive purple acid phosphatase 16.

Kozak: TCCATGT ID=cds-XP_044453037.1;Parent=rna-XM_044597102.1;Dbxref=GeneID:123185107,Genbank:XP_044453037.1;Name=XP_044453037.1;gbkey=CDS;gene=LOC123185107;product=uncharacterized protein LOC123185107;protein_id=XP_044453037.1

GO:0016021; Integral component of membrane.

Kozak: CAGATGC ID=cds-XP_044453396.1;Parent=rna-XM_044597461.1;Dbxref=GeneID:123185603,Genbank:XP_044453396.1;Name=XP_044453396.1;gbkey=CDS;gene=LOC123185603;product=transcription factor MYB80-like;protein_id=XP_044453396.1

GO:0003677; DNA binding.

Kozak: CCCATGT ID=cds-XP_044453673.1;Parent=rna-XM_044597738.1;Dbxref=GeneID:123185929,Genbank:XP_044453673.1;Name=XP_044453673.1;gbkey=CDS;gene=LOC123185929;product=

Late embryogenesis abundant protein 19-like.

Kozak: CAGATGC ID=cds-XP_044453736.1;Parent=rna-XM_044597801.1;Dbxref=GeneID:123185999,Genbank:XP_044453736.1;Name=XP_044453736.1;gbkey=CDS;gene=LOC123185999;product=pathogenesis-related protein PRMS-like;protein_id=XP_044453736.1

GO:0005615; Extracellular space.

Kozak: TCCATGT ID=cds-XP_044454215.1;Parent=rna-XM_044598280.1;Dbxref=GeneID:123186527,Genbank:XP_044454215.1;Name=XP_044454215.1;gbkey=CDS;gene=LOC123186527;product=

Loricrin-like.

Kozak: CCCATGC ID=cds-XP_044454310.1;Parent=rna-XM_044598375.1;Dbxref=GeneID:123186654,Genbank:XP_044454310.1;Name=XP_044454310.1;gbkey=CDS;gene=LOC123186654;product=desmethyl-deoxy-podophyllotoxin synthase-like;protein_id=XP_044454310.1

GO:0016021; Integral component of membrane.

Kozak: TTCATGT ID=cds-XP_044454459.1;Parent=rna-XM_044598524.1;Dbxref=GeneID:123186842,Genbank:XP_044454459.1;Name=XP_044454459.1;gbkey=CDS;gene=LOC123186842;product=

Receptor-like kinase TMK4.

Kozak: CCCATGT ID=cds-XP_044454837.1;Parent=rna-XM_044598902.1;Dbxref=GeneID:123187121,Genbank:XP_044454837.1;Name=XP_044454837.1;gbkey=CDS;gene=LOC123187121;product=sugar transporter ERD6-like 5;protein_id=XP_044454837.1

GO:0016021; Integral component of membrane.

Kozak: TACATGC ID=cds-XP_044455134.1;Parent=rna-XM_044599199.1;Dbxref=GeneID:123187339,Genbank:XP_044455134.1;Name=XP_044455134.1;gbkey=CDS;gene=LOC123187339;product=probable AMP deaminase;protein_id=XP_044455134.1

GO:0005829; Cytosol

Kozak: CCCATGC ID=cds-XP_044455360.1;Parent=rna-XM_044599425.1;Dbxref=GeneID:123187538,Genbank:XP_044455360.1;Name=XP_044455360.1;gbkey=CDS;gene=LOC123187538;product=putative ubiquitin-like-specific protease 1B;protein_id=XP_044455360.1

GO:0005634; Nucleus.

Kozak: CGAATGT ID=cds-XP_044455602.1;Parent=rna-XM_044599667.1;Dbxref=GeneID:123187743,Genbank:XP_044455602.1;Name=XP_044455602.1;gbkey=CDS;gene=LOC123187743;product=alpha-humulene 10-hydroxylase-like;protein_id=XP_044455602.1

GO:0016021; Integral component of membrane.

Kozak: CCAATGC ID=cds-XP_044455714.1;Parent=rna-XM_044599779.1;Dbxref=GeneID:123187821,Genbank:XP_044455714.1;Name=XP_044455714.1;gbkey=CDS;gene=LOC123187821;product=probable transcription factor At5g28040;protein_id=XP_044455714.1

GO:0005634; Nucleus.

Kozak: CCAATGC ID=cds-XP_044455992.1;Parent=rna-XM_044600057.1;Dbxref=GeneID:123188027,Genbank:XP_044455992.1;Name=XP_044455992.1;gbkey=CDS;gene=LOC123188027;product=uncharacterized hydrolase YugF-like;protein_id=XP_044455992.1

GO:0003824; Catalytic activity.

Kozak: CGAATGT ID=cds-XP_044456247.1;Parent=rna-XM_044600312.1;Dbxref=GeneID:123188263,Genbank:XP_044456247.1;Name=XP_044456247.1;gbkey=CDS;gene=LOC123188263;product=organic cation/carnitine transporter 2-like;protein_id=XP_044456247.1

GO:0016021; Integral component of membrane.

Kozak: TCCATGT ID=cds-XP_044456543.1;Parent=rna-XM_044600608.1;Dbxref=GeneID:123188470,Genbank:XP_044456543.1;Name=XP_044456543.1;gbkey=CDS;gene=LOC123188470;product=ATP-dependent DNA helicase SRS2-like protein At4g25120;protein_id=XP_044456543.1

GO:0005634; Nucleus.

Kozak: CCCATGC ID=cds-XP_044456591.1;Parent=rna-XM_044600656.1;Dbxref=GeneID:123188509,Genbank:XP_044456591.1;Name=XP_044456591.1;gbkey=CDS;gene=LOC123188509;product=protein trichome birefringence-like 13 isoform X1;protein_id=XP_044456591.1

GO:0005794; Golgi apparatus.

Kozak: CCCATGC ID=cds-XP_044456754.1;Parent=rna-XM_044600819.1;Dbxref=GeneID:123188629,Genbank:XP_044456754.1;Name=XP_044456754.1;gbkey=CDS;gene=LOC123188629;product=AT-rich interactive domain-containing protein 6-like;protein_id=XP_044456754.1

GO:0005634; Nucleus.

Kozak: CCCATGT ID=cds-XP_044456784.1;Parent=rna-XM_044600849.1;Dbxref=GeneID:123188643,Genbank:XP_044456784.1;Name=XP_044456784.1;gbkey=CDS;gene=LOC123188643;product=AUGMIN subunit 3-like isoform X1;protein_id=XP_044456784.1

GO:0005737; Cytoplasm.

Kozak: CCCATGC ID=cds-XP_044456958.1;Parent=rna-XM_044601023.1;Dbxref=GeneID:123188779,Genbank:XP_044456958.1;Name=XP_044456958.1;gbkey=CDS;gene=LOC123188779;product=probable LRR receptor-like serine/threonine-protein kinase At2g16250;protein_id=XP_044456958.1

GO:0016021; Integral component of membrane.

Kozak: CCAATGC ID=cds-XP_044457126.1;Parent=rna-XM_044601191.1;Dbxref=GeneID:123188912,Genbank:XP_044457126.1;Name=XP_044457126.1;gbkey=CDS;gene=LOC123188912;product=leucine-rich repeat receptor-like serine/threonine-protein kinase BAM1;protein_id=XP_044457126.1

GO:0016021; integral component of membrane.

Kozak: CCCATGT ID=cds-XP_044457410.1;Parent=rna-XM_044601475.1;Dbxref=GeneID:123189136,Genbank:XP_044457410.1;Name=XP_044457410.1;gbkey=CDS;gene=LOC123189136;product=cytochrome P450 77A4-like;protein_id=XP_044457410.1

GO:0016021; Integral component of membrane.

Kozak: CCAATGC ID=cds-XP_044457781.1;Parent=rna-XM_044601846.1;Dbxref=GeneID:123189434,Genbank:XP_044457781.1;Name=XP_044457781.1;gbkey=CDS;gene=LOC123189434;product=

Pentatricopeptide repeat-containing protein At4g26680%2C mitochondrial-like.

Kozak: CCAATGC ID=cds-XP_044458135.1;Parent=rna-XM_044602200.1;Dbxref=GeneID:123189726,Genbank:XP_044458135.1;Name=XP_044458135.1;gbkey=CDS;gene=LOC123189726;product=DNA mismatch repair protein MSH1%2C mitochondrial-like isoform X3;protein_id=XP_044458135.1

GO:0005739; Mitochondrion.

Kozak: TACATGC ID=cds-XP_044458493.1;Parent=rna-XM_044602558.1;Dbxref=GeneID:123190015,Genbank:XP_044458493.1;Name=XP_044458493.1;gbkey=CDS;gene=LOC123190015;product=

Calphotin-like.

Kozak: CAGATGC ID=cds-XP_044458603.1;Parent=rna-XM_044602668.1;Dbxref=GeneID:123190093,Genbank:XP_044458603.1;Name=XP_044458603.1;gbkey=CDS;gene=LOC123190093;product=polyubiquitin-like;protein_id=XP_044458603.1

GO:0005737; Cytoplasm.

Kozak: CAGATGC ID=cds-XP_044458607.1;Parent=rna-XM_044602672.1;Dbxref=GeneID:123190097,Genbank:XP_044458607.1;Name=XP_044458607.1;gbkey=CDS;gene=LOC123190097;product=

Polyubiquitin-like

Kozak: CCCATGT ID=cds-XP_044458743.1;Parent=rna-XM_044602808.1;Dbxref=GeneID:123190211,Genbank:XP_044458743.1;Name=XP_044458743.1;gbkey=CDS;gene=LOC123190211;product=ent-kaur-16-ene synthase%2C chloroplastic-like isoform X1;protein_id=XP_044458743.1

GO:0000287; Magnesium ion binding.

Kozak: CCCATGT ID=cds-XP_044458966.1;Parent=rna-XM_044603031.1;Dbxref=GeneID:123190407,Genbank:XP_044458966.1;Name=XP_044458966.1;gbkey=CDS;gene=LOC123190407;product=probable L-type lectin-domain containing receptor kinase S.5;protein_id=XP_044458966.1

GO:0016021; Integral component of membrane.

Kozak: CCCATGT ID=cds-XP_044460052.1;Parent=rna-XM_044604117.1;Dbxref=GeneID:123191332,Genbank:XP_044460052.1;Name=XP_044460052.1;gbkey=CDS;gene=LOC123191332;product=

Probable amidase At4g34880.

Kozak: CCAATGC ID=cds-XP_044460337.1;Parent=rna-XM_044604402.1;Dbxref=GeneID:123191810,Genbank:XP_044460337.1;Name=XP_044460337.1;gbkey=CDS;gene=LOC123191810;product=

Cyclin-D5-2-like.

Kozak: CCCATGC ID=cds-XP_044437277.1;Parent=rna-XM_044581342.1;Dbxref=GeneID:101290631,Genbank:XP_044437277.1;Name=XP_044437277.1;gbkey=CDS;gene=LOC101290631;product=superoxide dismutase [Fe] 2%2C chloroplastic;protein_id=XP_044437277.1

GO:0042644; Chloroplast nucleoid.

Kozak: CCCATGC ID=cds-XP_044437005.1;Parent=rna-XM_044581070.1;Dbxref=GeneID:123163716,Genbank:XP_044437005.1;Name=XP_044437005.1;gbkey=CDS;gene=LOC123163716;product=probable rhamnogalacturonate lyase B isoform X1;protein_id=XP_044437005.1

GO:0005576; Extracellular region.

Kozak: TCCATGT ID=cds-XP_044437093.1;Parent=rna-XM_044581158.1;Dbxref=GeneID:123163775,Genbank:XP_044437093.1;Name=XP_044437093.1;gbkey=CDS;gene=LOC123163775;product=probable transcriptional regulator SLK3;protein_id=XP_044437093.1

GO:0005634; Nucleus.

Kozak: CAGATGC ID=cds-XP_044437392.1;Parent=rna-XM_044581457.1;Dbxref=GeneID:123164034,Genbank:XP_044437392.1;Name=XP_044437392.1;gbkey=CDS;gene=LOC123164034;product=

Pentatricopeptide repeat-containing protein At1g80150%2C mitochondrial-like.

Kozak: CCCATGT ID=cds-XP_044437834.1;Parent=rna-XM_044581899.1;Dbxref=GeneID:123164430,Genbank:XP_044437834.1;Name=XP_044437834.1;gbkey=CDS;gene=LOC123164430;product=

Probable 3-beta-hydroxysteroid-Delta(8)%2CDelta(7)-isomerase isoform X1.

Kozak: TTCATGT ID=cds-XP_044438002.1;Parent=rna-XM_044582067.1;Dbxref=GeneID:123164547,Genbank:XP_044438002.1;Name=XP_044438002.1;gbkey=CDS;gene=LOC123164547;product=V-type proton ATPase subunit B 1-like;protein_id=XP_044438002.1

GO:0033180; Proton-transporting V-type ATPase, V1 domain.

Kozak: CCAATGC ID=cds-XP_044438407.1;Parent=rna-XM_044582472.1;Dbxref=GeneID:123164867,Genbank:XP_044438407.1;Name=XP_044438407.1;gbkey=CDS;gene=LOC123164867;product=probable LRR receptor-like serine/threonine-protein kinase At1g56140 isoform X1;protein_id=XP_044438407.1

GO:0016021; Integral component of membrane.

Kozak: CAGATGC ID=cds-XP_044438502.1;Parent=rna-XM_044582567.1;Dbxref=GeneID:123164948,Genbank:XP_044438502.1;Name=XP_044438502.1;gbkey=CDS;gene=LOC123164948;product=uncharacterized protein LOC123164948;protein_id=XP_044438502.1

GO:0008236; Serine-type peptidase activity.

Kozak: CCAATGC ID=cds-XP_044439316.1;Parent=rna-XM_044583381.1;Dbxref=GeneID:123165689,Genbank:XP_044439316.1;Name=XP_044439316.1;gbkey=CDS;gene=LOC123165689;product=transcription factor bHLH48-like isoform X1;protein_id=XP_044439316.1

GO:0005634; Nucleus.

Kozak: CCAATGC ID=cds-XP_044439503.1;Parent=rna-XM_044583568.1;Dbxref=GeneID:123165832,Genbank:XP_044439503.1;Name=XP_044439503.1;gbkey=CDS;gene=LOC123165832;product=RING-H2 finger protein ATL32-like;protein_id=XP_044439503.1

GO:0016021; Integral component of membrane.

Kozak: CAGATGC ID=cds-XP_044439571.1;Parent=rna-XM_044583636.1;Dbxref=GeneID:123165894,Genbank:XP_044439571.1;Name=XP_044439571.1;gbkey=CDS;gene=LOC123165894;product=

GEM-like protein 1.

Kozak: TCCATGT ID=cds-XP_044439634.1;Parent=rna-XM_044583699.1;Dbxref=GeneID:123165937,Genbank:XP_044439634.1;Name=XP_044439634.1;gbkey=CDS;gene=LOC123165937;product=

Protein NOI4-like.

Kozak: CCAATGT ID=cds-XP_044439644.1;Parent=rna-XM_044583709.1;Dbxref=GeneID:123165948,Genbank:XP_044439644.1;Name=XP_044439644.1;gbkey=CDS;gene=LOC123165948;product=

LysM domain-containing GPI-anchored protein LYP6-like.

Kozak: CCAATGC ID=cds-XP_044439729.1;Parent=rna-XM_044583794.1;Dbxref=GeneID:123166036,Genbank:XP_044439729.1;Name=XP_044439729.1;gbkey=CDS;gene=LOC123166036;product=pentatricopeptide repeat-containing protein At2g03380%2C mitochondrial-like;protein_id=XP_044439729.1

GO:0003723; :RNA binding.

Kozak: CAGATGC ID=cds-XP_044439896.1;Parent=rna-XM_044583961.1;Dbxref=GeneID:123166191,Genbank:XP_044439896.1;Name=XP_044439896.1;Note=The sequence of the model RefSeq protein was modified relative to this genomic sequence to represent the inferred CDS: added 257 bases not found in genome assembly;exception=annotated by transcript or proteomic data;gbkey=CDS;gene=LOC123166191;inference=similar to RNA sequence (same species):INSD:GIJS01155839.1;partial=true;product=polyubiquitin 11-like;protein_id=XP_044439896.1

GO:0005737; Cytoplasm.

Kozak: CAGATGC ID=cds-XP_044439897.1;Parent=rna-XM_044583962.1;Dbxref=GeneID:123166192,Genbank:XP_044439897.1;Name=XP_044439897.1;gbkey=CDS;gene=LOC123166192;product=

Polyubiquitin.

Kozak: CCCATGC ID=cds-XP_044439973.1;Parent=rna-XM_044584038.1;Dbxref=GeneID:123166263,Genbank:XP_044439973.1;Name=XP_044439973.1;gbkey=CDS;gene=LOC123166263;product=lysophospholipid acyltransferase LPEAT2-like isoform X3;protein_id=XP_044439973.1

GO:0016021; Integral component of membrane.

Kozak: CCAATGC ID=cds-XP_044440432.1;Parent=rna-XM_044584497.1;Dbxref=GeneID:123166701,Genbank:XP_044440432.1;Name=XP_044440432.1;gbkey=CDS;gene=LOC123166701;product=mannan endo-1%2C4-beta-mannosidase 6-like;protein_id=XP_044440432.1

GO:0016985; Mannan endo-1,4-beta-mannosidase activity.

Kozak: TACATGC ID=cds-XP_044440724.1;Parent=rna-XM_044584789.1;Dbxref=GeneID:123166965,Genbank:XP_044440724.1;Name=XP_044440724.1;gbkey=CDS;gene=LOC123166965;product=

GDSL esterase/lipase LTL1-like isoform X1.

Kozak: CCCATGC ID=cds-XP_044441188.1;Parent=rna-XM_044585253.1;Dbxref=GeneID:123167416,Genbank:XP_044441188.1;Name=XP_044441188.1;gbkey=CDS;gene=LOC123167416;product=

Pentatricopeptide repeat-containing protein At2g26790%2C mitochondrial-like.

Kozak: CCAATGC ID=cds-XP_044441869.1;Parent=rna-XM_044585934.1;Dbxref=GeneID:123168067,Genbank:XP_044441869.1;Name=XP_044441869.1;gbkey=CDS;gene=LOC123168067;product=

Probable cation transporter HKT9.

Kozak: CCAATGC ID=cds-XP_044441906.1;Parent=rna-XM_044585971.1;Dbxref=GeneID:123168109,Genbank:XP_044441906.1;Name=XP_044441906.1;gbkey=CDS;gene=LOC123168109;product=pentatricopeptide repeat-containing protein At4g33990-like;protein_id=XP_044441906.1

GO:0008270; Zinc ion binding.

Kozak: TTAATGC ID=cds-XP_044441927.1;Parent=rna-XM_044585992.1;Dbxref=GeneID:123168124,Genbank:XP_044441927.1;Name=XP_044441927.1;gbkey=CDS;gene=LOC123168124;product=uncharacterized protein LOC123168124;protein_id=XP_044441927.1

GO:0016021; Integral component of membrane.

Kozak: CCAATGC ID=cds-XP_044442026.1;Parent=rna-XM_044586091.1;Dbxref=GeneID:123168218,Genbank:XP_044442026.1;Name=XP_044442026.1;gbkey=CDS;gene=LOC123168218;product=BTB/POZ and MATH domain-containing protein 2-like;protein_id=XP_044442026.1

GO:0016567; Protein ubiquitination.

Kozak: CCCATGC ID=cds-XP_044442293.1;Parent=rna-XM_044586358.1;Dbxref=GeneID:123168472,Genbank:XP_044442293.1;Name=XP_044442293.1;gbkey=CDS;gene=LOC123168472;product=pentatricopeptide repeat-containing protein At1g18485-like;protein_id=XP_044442293.1

GO:0008270; Zinc ion binding.

Kozak: CCCATGC ID=cds-XP_044442349.1;Parent=rna-XM_044586414.1;Dbxref=GeneID:123168531,Genbank:XP_044442349.1;Name=XP_044442349.1;gbkey=CDS;gene=LOC123168531;product=

7-deoxyloganetin glucosyltransferase-like.

Kozak: CCCATGC ID=cds-XP_044442714.1;Parent=rna-XM_044586779.1;Dbxref=GeneID:123168915,Genbank:XP_044442714.1;Name=XP_044442714.1;gbkey=CDS;gene=LOC123168915;product=thioredoxin 1-like;protein_id=XP_044442714.1

GO:0005737; Cytoplasm.

Kozak: CCAATGT ID=cds-XP_044443329.1;Parent=rna-XM_044587394.1;Dbxref=GeneID:123169522,Genbank:XP_044443329.1;Name=XP_044443329.1;gbkey=CDS;gene=LOC123169522;product=LRR receptor-like serine/threonine-protein kinase RGI3;protein_id=XP_044443329.1

GO:0016021; Integral component of membrane.

Kozak: TCCATGT ID=cds-XP_044443374.1;Parent=rna-XM_044587439.1;Dbxref=GeneID:123169576,Genbank:XP_044443374.1;Name=XP_044443374.1;gbkey=CDS;gene=LOC123169576;product=probable mitochondrial saccharopine dehydrogenase-like oxidoreductase At5g39410;protein_id=XP_044443374.1

GO:0016021; Integral component of membrane.

Kozak: TCCATGT ID=cds-XP_044443389.1;Parent=rna-XM_044587454.1;Dbxref=GeneID:123169589,Genbank:XP_044443389.1;Name=XP_044443389.1;gbkey=CDS;gene=LOC123169589;product=basic leucine zipper 19-like;protein_id=XP_044443389.1

GO:0000981; DNA-binding transcription factor activity, RNA polymerase II-specific,

Kozak: TTAATGC ID=cds-XP_044443529.1;Parent=rna-XM_044587594.1;Dbxref=GeneID:123169725,Genbank:XP_044443529.1;Name=XP_044443529.1;gbkey=CDS;gene=LOC123169725;product=

GRF-interacting factor 1-like.

Kozak: TCCATGT ID=cds-XP_044444091.1;Parent=rna-XM_044588156.1;Dbxref=GeneID:123170309,Genbank:XP_044444091.1;Name=XP_044444091.1;gbkey=CDS;gene=LOC123170309;product=uncharacterized protein LOC123170309;protein_id=XP_044444091.1

GO:0016791; Phosphatase activity.

Kozak: TCCATGT ID=cds-XP_044444173.1;Parent=rna-XM_044588238.1;Dbxref=GeneID:123170378,Genbank:XP_044444173.1;Name=XP_044444173.1;gbkey=CDS;gene=LOC123170378;product=

Cell division cycle-associated protein 7-like.

Kozak: CCCATGT ID=cds-XP_044444374.1;Parent=rna-XM_044588439.1;Dbxref=GeneID:123170593,Genbank:XP_044444374.1;Name=XP_044444374.1;gbkey=CDS;gene=LOC123170593;product=

Protein FRA10AC1-like

Kozak: CCCATGC ID=cds-XP_044444473.1;Parent=rna-XM_044588538.1;Dbxref=GeneID:123170774,Genbank:XP_044444473.1;Name=XP_044444473.1;gbkey=CDS;gene=LOC123170774;product=

Putative E3 ubiquitin-protein ligase RING1a.

Kozak: TCCATGT ID=cds-XP_044444745.1;Parent=rna-XM_044588810.1;Dbxref=GeneID:123171201,Genbank:XP_044444745.1;Name=XP_044444745.1;gbkey=CDS;gene=LOC123171201;product=nuclear intron maturase 4%2C mitochondrial-like;protein_id=XP_044444745.1

GO:0016021; Integral component of membrane.

Kozak: CAGATGC ID=cds-XP_044444839.1;Parent=rna-XM_044588904.1;Dbxref=GeneID:123171376,Genbank:XP_044444839.1;Name=XP_044444839.1;gbkey=CDS;gene=LOC123171376;product=

Nuclear transport factor 2-like.

Kozak: CCCATGT ID=cds-XP_044442133.1;Parent=rna-XM_044586198.1;Dbxref=GeneID:100192175,Genbank:XP_044442133.1;Name=XP_044442133.1;gbkey=CDS;gene=LOC100192175;product=DNA repair protein RAD51 homolog B;protein_id=XP_044442133.1

GO:0000794; Condensed nuclear chromosome.

Kozak: CCAATGT ID=cds-XP_044440426.1;Parent=rna-XM_044584491.1;Dbxref=GeneID:100873123,Genbank:XP_044440426.1;Name=XP_044440426.1;gbkey=CDS;gene=LOC100873123;product=transcription factor KUA1 isoform X1;protein_id=XP_044440426.1

GO:0003677; DNA binding.

Kozak: TACATGC ID=cds-XP_044437130.1;Parent=rna-XM_044581195.1;Dbxref=GeneID:123163813,Genbank:XP_044437130.1;Name=XP_044437130.1;gbkey=CDS;gene=LOC123163813;product=tRNA pseudouridine synthase A-like isoform X1;protein_id=XP_044437130.1

GO:0009982; Pseudouridine synthase activity.

Kozak: TACATGC ID=cds-XP_044437400.1;Parent=rna-XM_044581465.1;Dbxref=GeneID:123164048,Genbank:XP_044437400.1;Name=XP_044437400.1;gbkey=CDS;gene=LOC123164048;product=APETALA2-like protein 4;protein_id=XP_044437400.1

GO:0005634; Nucleus.

Kozak: TACATGC ID=cds-XP_044437470.1;Parent=rna-XM_044581535.1;Dbxref=GeneID:123164115,Genbank:XP_044437470.1;Name=XP_044437470.1;gbkey=CDS;gene=LOC123164115;product=

Protein RKD4-like.

Kozak: TCCATGT ID=cds-XP_044437495.1;Parent=rna-XM_044581560.1;Dbxref=GeneID:123164136,Genbank:XP_044437495.1;Name=XP_044437495.1;gbkey=CDS;gene=LOC123164136;product=paired amphipathic helix protein Sin3-like 4;protein_id=XP_044437495.1

GO:0016021; Integral component of membrane.

Kozak: CAGATGC ID=cds-XP_044437819.1;Parent=rna-XM_044581884.1;Dbxref=GeneID:123164414,Genbank:XP_044437819.1;Name=XP_044437819.1;gbkey=CDS;gene=LOC123164414;product=

Polyubiquitin.

kozak: TACATGC ID=cds-XP_044437916.1;Parent=rna-XM_044581981.1;Dbxref=GeneID:123164470,Genbank:XP_044437916.1;Name=XP_044437916.1;gbkey=CDS;gene=LOC123164470;product=

Brassinosteroid-responsive RING protein 1-like;protein.

Kozak: CCAATGC ID=cds-XP_044438015.1;Parent=rna-XM_044582080.1;Dbxref=GeneID:123164554,Genbank:XP_044438015.1;Name=XP_044438015.1;gbkey=CDS;gene=LOC123164554;product=protein MICRORCHIDIA 2-like;protein_id=XP_044438015.1

GO:0005634; Nucleus.

Kozak: CCCATGT ID=cds-XP_044438568.1;Parent=rna-XM_044582633.1;Dbxref=GeneID:123165005,Genbank:XP_044438568.1;Name=XP_044438568.1;gbkey=CDS;gene=LOC123165005;product=afadin- and alpha-actinin-binding protein-like isoform X3;protein_id=XP_044438568.1

GO:0110165; Cellular anatomical entity.

Kozak: CCAATGC ID=cds-XP_044438659.1;Parent=rna-XM_044582724.1;Dbxref=GeneID:123165093,Genbank:XP_044438659.1;Name=XP_044438659.1;gbkey=CDS;gene=LOC123165093;product=uncharacterized protein LOC123165093 isoform X1;protein_id=XP_044438659.1

GO:0043231; Intracellular membrane-bounded organelle.

Kozak: CCCATGC ID=cds-XP_044439003.1;Parent=rna-XM_044583068.1;Dbxref=GeneID:123165432,Genbank:XP_044439003.1;Name=XP_044439003.1;gbkey=CDS;gene=LOC123165432;product=phytoene synthase 1%2C chloroplastic-like;protein_id=XP_044439003.1

GO:0010287; Plastoglobuli.

Kozak: CCAATGT ID=cds-XP_044439042.1;Parent=rna-XM_044583107.1;Dbxref=GeneID:123165459,Genbank:XP_044439042.1;Name=XP_044439042.1;gbkey=CDS;gene=LOC123165459;product=

Pentatricopeptide repeat-containing protein At5g42310%2C chloroplastic-like isoform X1.

Kozak: CCCATGC ID=cds-XP_044439322.1;Parent=rna-XM_044583387.1;Dbxref=GeneID:123165693,Genbank:XP_044439322.1;Name=XP_044439322.1;gbkey=CDS;gene=LOC123165693;product=serine/threonine-protein kinase UCN-like;protein_id=XP_044439322.1

GO:0005524; ATP binding.

Kozak: TCCATGT ID=cds-XP_044439665.1;Parent=rna-XM_044583730.1;Dbxref=GeneID:123165968,Genbank:XP_044439665.1;Name=XP_044439665.1;gbkey=CDS;gene=LOC123165968;product=kinesin-like protein KIN-14M;protein_id=XP_044439665.1

GO:0005874; Microtubule.

Kozak: CCAATGT ID=cds-XP_044439781.1;Parent=rna-XM_044583846.1;Dbxref=GeneID:123166084,Genbank:XP_044439781.1;Name=XP_044439781.1;gbkey=CDS;gene=LOC123166084;product=anthocyanidin 5%2C3-O-glucosyltransferase-like;protein_id=XP_044439781.1

GO:0005634; Nucleus.

Kozak: TCCATGT ID=cds-XP_044439969.1;Parent=rna-XM_044584034.1;Dbxref=GeneID:123166262,Genbank:XP_044439969.1;Name=XP_044439969.1;Note=The sequence of the model RefSeq protein was modified relative to this genomic sequence to represent the inferred CDS: added 32 bases not found in genome assembly;exception=annotated by transcript or proteomic data;gbkey=CDS;gene=LOC123166262;inference=similar to RNA sequence (same species):INSD:GEWU01180337.1;partial=true;product=ubiquitin carboxyl-terminal hydrolase MINDY-2-like isoform X1;protein_id=XP_044439969.1

GO:0016807; Cysteine-type carboxypeptidase activity.

Kozak: TCCATGT ID=cds-XP_044440207.1;Parent=rna-XM_044584272.1;Dbxref=GeneID:123166466,Genbank:XP_044440207.1;Name=XP_044440207.1;gbkey=CDS;gene=LOC123166466;product=probable methionine--tRNA ligase;protein_id=XP_044440207.1

GO:0005829; Cytosol.

Kozak: CCAATGT ID=cds-XP_044440227.1;Parent=rna-XM_044584292.1;Dbxref=GeneID:123166487,Genbank:XP_044440227.1;Name=XP_044440227.1;gbkey=CDS;gene=LOC123166487;product=UDP-glycosyltransferase CGT-like;protein_id=XP_044440227.1

GO:0008194; UDP-glycosyltransferase activity.

Kozak: CAGATGC ID=cds-XP_044440542.1;Parent=rna-XM_044584607.1;Dbxref=GeneID:123166807,Genbank:XP_044440542.1;Name=XP_044440542.1;gbkey=CDS;gene=LOC123166807;product=

Pentatricopeptide repeat-containing protein At3g29230-like

Kozak: CCCATGC ID=cds-XP_044440817.1;Parent=rna-XM_044584882.1;Dbxref=GeneID:123167045,Genbank:XP_044440817.1;Name=XP_044440817.1;gbkey=CDS;gene=LOC123167045;product=serine/threonine/tyrosine-protein kinase HT1-like;protein_id=XP_044440817.1

GO:0005524; ATP binding.

Kozak: CCAATGC ID=cds-XP_044440958.1;Parent=rna-XM_044585023.1;Dbxref=GeneID:123167191,Genbank:XP_044440958.1;Name=XP_044440958.1;gbkey=CDS;gene=LOC123167191;product=

Protein FAR1-RELATED SEQUENCE 6-like.

Kozak: CCAATGC ID=cds-XP_044440996.1;Parent=rna-XM_044585061.1;Dbxref=GeneID:123167228,Genbank:XP_044440996.1;Name=XP_044440996.1;gbkey=CDS;gene=LOC123167228;product=fucosyltransferase 2-like;protein_id=XP_044440996.1

GO:0032580; Golgi cisterna membrane.

Kozak: CCCATGC ID=cds-XP_044441186.1;Parent=rna-XM_044585251.1;Dbxref=GeneID:123167415,Genbank:XP_044441186.1;Name=XP_044441186.1;gbkey=CDS;gene=LOC123167415;product=putative transferase At4g12130%2C mitochondrial isoform X1;protein_id=XP_044441186.1

GO:0005759; Mitochondrial matrix.

Kozak: CCCATGT ID=cds-XP_044441204.1;Parent=rna-XM_044585269.1;Dbxref=GeneID:123167430,Genbank:XP_044441204.1;Name=XP_044441204.1;gbkey=CDS;gene=LOC123167430;product=

Formin-like protein 20.

Kozak: CCAATGC ID=cds-XP_044441584.1;Parent=rna-XM_044585649.1;Dbxref=GeneID:123167786,Genbank:XP_044441584.1;Name=XP_044441584.1;gbkey=CDS;gene=LOC123167786;product=O-methyltransferase MdmC-like;protein_id=XP_044441584.1

GO:0005829; Cytosol.

Kozak: TTCATGT ID=cds-XP_044441684.1;Parent=rna-XM_044585749.1;Dbxref=GeneID:123167885,Genbank:XP_044441684.1;Name=XP_044441684.1;gbkey=CDS;gene=LOC123167885;product=expansin-A32-like;protein_id=XP_044441684.1

GO:0005576; Extracellular region.

Kozak: TGTATGT ID=cds-XP_044441876.1;Parent=rna-XM_044585941.1;Dbxref=GeneID:123168075,Genbank:XP_044441876.1;Name=XP_044441876.1;gbkey=CDS;gene=LOC123168075;product=uncharacterized protein LOC123168075;protein_id=XP_044441876.1

GO:0016021; Integral component of membrane.

Kozak: CAGATGC ID=cds-XP_044442437.1;Parent=rna-XM_044586502.1;Dbxref=GeneID:123168618,Genbank:XP_044442437.1;Name=XP_044442437.1;gbkey=CDS;gene=LOC123168618;product=PH%2C RCC1 and FYVE domains-containing protein 1-like isoform X2;protein_id=XP_044442437.1

GO:0046872; Metal ion binding.

Kozak: CCCATGT ID=cds-XP_044442644.1;Parent=rna-XM_044586709.1;Dbxref=GeneID:123168848,Genbank:XP_044442644.1;Name=XP_044442644.1;gbkey=CDS;gene=LOC123168848;product=

Classical arabinogalactan protein 9-like.

Kozak: CAGATGC ID=cds-XP_044442791.1;Parent=rna-XM_044586856.1;Dbxref=GeneID:123168997,Genbank:XP_044442791.1;Name=XP_044442791.1;gbkey=CDS;gene=LOC123168997;product=polyubiquitin;protein_id=XP_044442791.1

GO:0005737; Cytoplasm.

Kozak: TCCATGT ID=cds-XP_044443020.1;Parent=rna-XM_044587085.1;Dbxref=GeneID:123169250,Genbank:XP_044443020.1;Name=XP_044443020.1;gbkey=CDS;gene=LOC123169250;product=NDR1/HIN1-like protein 26;protein_id=XP_044443020.1

GO:0012505; Endomembrane system.

Kozak: CCAATGC ID=cds-XP_044443295.1;Parent=rna-XM_044587360.1;Dbxref=GeneID:123169486,Genbank:XP_044443295.1;Name=XP_044443295.1;gbkey=CDS;gene=LOC123169486;product=UPF0481 protein At3g47200-like isoform X1;protein_id=XP_044443295.1

GO:0005737; Cytoplasm.

Kozak: CAGATGC ID=cds-XP_044443373.1;Parent=rna-XM_044587438.1;Dbxref=GeneID:123169575,Genbank:XP_044443373.1;Name=XP_044443373.1;gbkey=CDS;gene=LOC123169575;product=cytochrome P450 72A397-like;protein_id=XP_044443373.1

GO:0016021; Integral component of membrane.

Kozak: CAGATGC ID=cds-XP_044443570.1;Parent=rna-XM_044587635.1;Dbxref=GeneID:123169765,Genbank:XP_044443570.1;Name=XP_044443570.1;gbkey=CDS;gene=LOC123169765;product=protein trichome birefringence-like 5;protein_id=XP_044443570.1

GO:0005794; Golgi apparatus.

Kozak: CCCATGT ID=cds-XP_044444052.1;Parent=rna-XM_044588117.1;Dbxref=GeneID:123170285,Genbank:XP_044444052.1;Name=XP_044444052.1;gbkey=CDS;gene=LOC123170285;product=transcription factor TGAL3-like isoform X1;protein_id=XP_044444052.1

GO:0003700; DNA-binding transcription factor activity.

Kozak: CAGATGC ID=cds-XP_044444212.1;Parent=rna-XM_044588277.1;Dbxref=GeneID:123170412,Genbank:XP_044444212.1;Name=XP_044444212.1;gbkey=CDS;gene=LOC123170412;product=

Polyubiquitin-like.

Kozak: TCCATGT ID=cds-XP_044444291.1;Parent=rna-XM_044588356.1;Dbxref=GeneID:123170495,Genbank:XP_044444291.1;Name=XP_044444291.1;gbkey=CDS;gene=LOC123170495;product=probable transcriptional regulator SLK2 isoform X1;protein_id=XP_044444291.1

GO:0005634; Nucleus

Kozak: TCCATGT ID=cds-XP_044444382.1;Parent=rna-XM_044588447.1;Dbxref=GeneID:123170606,Genbank:XP_044444382.1;Name=XP_044444382.1;gbkey=CDS;gene=LOC123170606;product=

F-box protein At5g49610-like.

Kozak: TCCATGT ID=cds-XP_044444556.1;Parent=rna-XM_044588621.1;Dbxref=GeneID:123170903,Genbank:XP_044444556.1;Name=XP_044444556.1;gbkey=CDS;gene=LOC123170903;product=

E3 ubiquitin-protein ligase RFWD3-like.

Kozak: CAGATGC ID=cds-XP_044444704.1;Parent=rna-XM_044588769.1;Dbxref=GeneID:123171140,Genbank:XP_044444704.1;Name=XP_044444704.1;gbkey=CDS;gene=LOC123171140;product=

CASP-like protein 5B3.

Kozak: TCCATGT ID=cds-XP_044440430.1;Parent=rna-XM_044584495.1;Dbxref=GeneID:542986,Genbank:XP_044440430.1;Name=XP_044440430.1;gbkey=CDS;gene=LOC542986;product=soluble starch synthase 2-3%2C chloroplastic/amyloplastic;protein_id=XP_044440430.1

GO:0009501; Amyloplast.

Kozak: CCCATGC ID=cds-XP_044438999.1;Parent=rna-XM_044583064.1;Dbxref=GeneID:543430,Genbank:XP_044438999.1;Name=XP_044438999.1;gbkey=CDS;gene=LOC543430;product=ATP-dependent 6-phosphofructokinase 6;protein_id=XP_044438999.1

GO:0005737; Cytoplasm.

Kozak: TACATGC ID=cds-XP_044378858.1;Parent=rna-XM_044522923.1;Dbxref=GeneID:123101493,Genbank:XP_044378858.1;Name=XP_044378858.1;gbkey=CDS;gene=LOC123101493;product=

Wax ester synthase/diacylglycerol acyltransferase 7-like.

Kozak: TTCATGT ID=cds-XP_044379110.1;Parent=rna-XM_044523175.1;Dbxref=GeneID:123101931,Genbank:XP_044379110.1;Name=XP_044379110.1;gbkey=CDS;gene=LOC123101931;product=

Pentatricopeptide repeat-containing protein At1g18900-like.

Kozak: CCAATGT ID=cds-XP_044379414.1;Parent=rna-XM_044523479.1;Dbxref=GeneID:123102177,Genbank:XP_044379414.1;Name=XP_044379414.1;gbkey=CDS;gene=LOC123102177;product=

Wall-associated receptor kinase 3-like.

Kozak: CAGATGC ID=cds-XP_044379437.1;Parent=rna-XM_044523502.1;Dbxref=GeneID:123102197,Genbank:XP_044379437.1;Name=XP_044379437.1;gbkey=CDS;gene=LOC123102197;product=

Pentatricopeptide repeat-containing protein At4g18520%2C chloroplastic-like.

Kozak: CCCATGC ID=cds-XP_044379567.1;Parent=rna-XM_044523632.1;Dbxref=GeneID:123102325,Genbank:XP_044379567.1;Name=XP_044379567.1;gbkey=CDS;gene=LOC123102325;product=gamma-tubulin complex component 5-like;protein_id=XP_044379567.1

GO:0005737; Cytoplasm.

Kozak: CCAATGC ID=cds-XP_044379700.1;Parent=rna-XM_044523765.1;Dbxref=GeneID:123102417,Genbank:XP_044379700.1;Name=XP_044379700.1;gbkey=CDS;gene=LOC123102417;product=cytochrome P450 81Q32-like;protein_id=XP_044379700.1

GO:0016021; Integral component of membrane.

Kozak: CCAATGT ID=cds-XP_044379731.1;Parent=rna-XM_044523796.1;Dbxref=GeneID:123102450,Genbank:XP_044379731.1;Name=XP_044379731.1;gbkey=CDS;gene=LOC123102450;product=pentatricopeptide repeat-containing protein At3g29230-like;protein_id=XP_044379731.1

GO:0005737; Cytoplasm.

Kozak: CCCATGT ID=cds-XP_044379896.1;Parent=rna-XM_044523961.1;Dbxref=GeneID:123102561,Genbank:XP_044379896.1;Name=XP_044379896.1;gbkey=CDS;gene=LOC123102561;product=psbP domain-containing protein 1%2C chloroplastic-like;protein_id=XP_044379896.1ç

GO:0009507; Chloroplast.

Kozak: CCAATGC ID=cds-XP_044380129.1;Parent=rna-XM_044524194.1;Dbxref=GeneID:123102768,Genbank:XP_044380129.1;Name=XP_044380129.1;gbkey=CDS;gene=LOC123102768;product=

Calcium-dependent protein kinase 28-like.

Kozak: TCCATGT ID=cds-XP_044380174.1;Parent=rna-XM_044524239.1;Dbxref=GeneID:123102809,Genbank:XP_044380174.1;Name=XP_044380174.1;gbkey=CDS;gene=LOC123102809;product=quinolinate synthase%2C chloroplastic-like;protein_id=XP_044380174.1

GO:0009507; Chloroplast.

Kozak: CCCATGC ID=cds-XP_044380253.1;Parent=rna-XM_044524318.1;Dbxref=GeneID:123102859,Genbank:XP_044380253.1;Name=XP_044380253.1;gbkey=CDS;gene=LOC123102859;product=chaperone protein dnaJ A7A%2C chloroplastic-like;protein_id=XP_044380253.1

GO:0005737; Cytoplasm.

Kozak: TTCATGT ID=cds-XP_044380633.1;Parent=rna-XM_044524698.1;Dbxref=GeneID:123103193,Genbank:XP_044380633.1;Name=XP_044380633.1;gbkey=CDS;gene=LOC123103193;product=putative pentatricopeptide repeat-containing protein At3g08820;protein_id=XP_044380633.1

GO:0008270; Zinc ion binding.

Kozak: TCCATGT ID=cds-XP_044380663.1;Parent=rna-XM_044524728.1;Dbxref=GeneID:123103214,Genbank:XP_044380663.1;Name=XP_044380663.1;gbkey=CDS;gene=LOC123103214;product=protein NRT1/ PTR FAMILY 2.3-like;protein_id=XP_044380663.1

GO:0016021; Integral component of membrane.

Kozak: CAGATGC ID=cds-XP_044380702.1;Parent=rna-XM_044524767.1;Dbxref=GeneID:123103242,Genbank:XP_044380702.1;Name=XP_044380702.1;gbkey=CDS;gene=LOC123103242;product=glucan endo-1%2C3-beta-glucosidase 4-like;protein_id=XP_044380702.1

GO:0016021; Integral component of membrane.

Kozak: CAGATGC ID=cds-XP_044381001.1;Parent=rna-XM_044525066.1;Dbxref=GeneID:123103468,Genbank:XP_044381001.1;Name=XP_044381001.1;gbkey=CDS;gene=LOC123103468;product=

Protein FAR1-RELATED SEQUENCE 5-like isoform X2.

Kozak: CCCATGC ID=cds-XP_044381355.1;Parent=rna-XM_044525420.1;Dbxref=GeneID:123103746,Genbank:XP_044381355.1;Name=XP_044381355.1;gbkey=CDS;gene=LOC123103746;product=cytoplasmic tRNA 2-thiolation protein 1;protein_id=XP_044381355.1

GO:0002144; Cytosolic tRNA wobble base thiouridylase complex.

Kozak: CCAATGT ID=cds-XP_044381544.1;Parent=rna-XM_044525609.1;Dbxref=GeneID:123103910,Genbank:XP_044381544.1;Name=XP_044381544.1;gbkey=CDS;gene=LOC123103910;product=

Probable cadmium/zinc-transporting ATPase HMA1%2C chloroplastic.

Kozak: CCCATGT ID=cds-XP_044381690.1;Parent=rna-XM_044525755.1;Dbxref=GeneID:123104033,Genbank:XP_044381690.1;Name=XP_044381690.1;gbkey=CDS;gene=LOC123104033;product=phosphoribosylaminoimidazole-succinocarboxamide synthase%2C chloroplastic-like;protein_id=XP_044381690.1

GO:0005524; ATP binding.

Kozak: TTCATGT ID=cds-XP_044381741.1;Parent=rna-XM_044525806.1;Dbxref=GeneID:123104084,Genbank:XP_044381741.1;Name=XP_044381741.1;gbkey=CDS;gene=LOC123104084;product=serine/threonine-protein kinase D6PK-like;protein_id=XP_044381741.1

GO:0005737; Cytoplasm.

Kozak: CCAATGC ID=cds-XP_044381761.1;Parent=rna-XM_044525826.1;Dbxref=GeneID:123104097,Genbank:XP_044381761.1;Name=XP_044381761.1;gbkey=CDS;gene=LOC123104097;product=leucine-rich repeat receptor-like serine/threonine-protein kinase RGI4;protein_id=XP_044381761.1

GO:0016021; Integral component of membrane.

Kozak: CAGATGC ID=cds-XP_044381852.1;Parent=rna-XM_044525917.1;Dbxref=GeneID:123104161,Genbank:XP_044381852.1;Name=XP_044381852.1;gbkey=CDS;gene=LOC123104161;product=

OVARIAN TUMOR DOMAIN-containing deubiquitinating enzyme 4-like.

Kozak: CAGATGC ID=cds-XP_044382095.1;Parent=rna-XM_044526160.1;Dbxref=GeneID:123104334,Genbank:XP_044382095.1;Name=XP_044382095.1;gbkey=CDS;gene=LOC123104334;product=probable UDP-arabinose 4-epimerase 1;protein_id=XP_044382095.1

GO:0003978; UDP-glucose 4-epimerase activity.

Kozak: TACATGC ID=cds-XP_044382367.1;Parent=rna-XM_044526432.1;Dbxref=GeneID:123104558,Genbank:XP_044382367.1;Name=XP_044382367.1;gbkey=CDS;gene=LOC123104558;product=wax ester synthase/diacylglycerol acyltransferase 11-like;protein_id=XP_044382367.1

GO:0005886; Plasma membrane.

Kozak: CCCATGC ID=cds-XP_044382379.1;Parent=rna-XM_044526444.1;Dbxref=GeneID:123104570,Genbank:XP_044382379.1;Name=XP_044382379.1;gbkey=CDS;gene=LOC123104570;product=uncharacterized protein LOC123104570;protein_id=XP_044382379.1

GO:0042651; Thylakoid membrane.

Kozak: TTAATGC ID=cds-XP_044382712.1;Parent=rna-XM_044526777.1;Dbxref=GeneID:123104861,Genbank:XP_044382712.1;Name=XP_044382712.1;gbkey=CDS;gene=LOC123104861;product=protein TRIGALACTOSYLDIACYLGLYCEROL 3%2C chloroplastic-like isoform X1;protein_id=XP_044382712.1

GO:0005524 ATP binding.

Kozak: CAGATGC ID=cds-XP_044382775.1;Parent=rna-XM_044526840.1;Dbxref=GeneID:123104904,Genbank:XP_044382775.1;Name=XP_044382775.1;gbkey=CDS;gene=LOC123104904;product=9-cis-epoxycarotenoid dioxygenase NCED3%2C chloroplastic-like;protein_id=XP_044382775.1

GO:0009570; Chloroplast stroma.

Kozak: CAGATGC ID=cds-XP_044382959.1;Parent=rna-XM_044527024.1;Dbxref=GeneID:123105050,Genbank:XP_044382959.1;Name=XP_044382959.1;gbkey=CDS;gene=LOC123105050;product=protein PYRICULARIA ORYZAE RESISTANCE 21-like;protein_id=XP_044382959.1

GO:0046872; Metal ion binding.

Kozak: CCAATGC ID=cds-XP_044383121.1;Parent=rna-XM_044527186.1;Dbxref=GeneID:123105179,Genbank:XP_044383121.1;Name=XP_044383121.1;gbkey=CDS;gene=LOC123105179;product=leucine-rich repeat receptor-like serine/threonine-protein kinase BAM1;protein_id=XP_044383121.1

GO:0016021; Integral component of membrane.

Kozak: CAGATGC ID=cds-XP_044383502.1;Parent=rna-XM_044527567.1;Dbxref=GeneID:123105486,Genbank:XP_044383502.1;Name=XP_044383502.1;gbkey=CDS;gene=LOC123105486;product=LEAF RUST 10 DISEASE-RESISTANCE LOCUS RECEPTOR-LIKE PROTEIN KINASE-like 1.5;protein_id=XP_044383502.1

GO:0016021; Integral component of membrane.

Kozak: TCCATGT ID=cds-XP_044383777.1;Parent=rna-XM_044527842.1;Dbxref=GeneID:123105721,Genbank:XP_044383777.1;Name=XP_044383777.1;gbkey=CDS;gene=LOC123105721;product=methyltransferase N6AMT1-like;protein_id=XP_044383777.1

GO:0035657; eRF1 methyltransferase complex.

Kozak: CAGATGC ID=cds-XP_044383785.1;Parent=rna-XM_044527850.1;Dbxref=GeneID:123105730,Genbank:XP_044383785.1;Name=XP_044383785.1;gbkey=CDS;gene=LOC123105730;product=protein DEFECTIVE IN EXINE FORMATION 1-like;protein_id=XP_044383785.1

GO:0016021; Integral component of membrane.

Kozak: CAGATGC ID=cds-XP_044384202.1;Parent=rna-XM_044528267.1;Dbxref=GeneID:123106066,Genbank:XP_044384202.1;Name=XP_044384202.1;gbkey=CDS;gene=LOC123106066;product=

pentatricopeptide repeat-containing protein At1g03100%2C mitochondrial-like.

Kozak: CCCATGC ID=cds-XP_044384588.1;Parent=rna-XM_044528653.1;Dbxref=GeneID:123106504,Genbank:XP_044384588.1;Name=XP_044384588.1;gbkey=CDS;gene=LOC123106504;product=germin-like protein 8-4;protein_id=XP_044384588.1

GO:0048046; Apoplast.

Kozak: TTAATGC ID=cds-XP_044384767.1;Parent=rna-XM_044528832.1;Dbxref=GeneID:123106769,Genbank:XP_044384767.1;Name=XP_044384767.1;gbkey=CDS;gene=LOC123106769;product=

Basic salivary proline-rich protein 3-like.

Kozak: TTCATGT ID=cds-XP_044384795.1;Parent=rna-XM_044528860.1;Dbxref=GeneID:123106817,Genbank:XP_044384795.1;Name=XP_044384795.1;gbkey=CDS;gene=LOC123106817;product=purple acid phosphatase 22-like;protein_id=XP_044384795.1

GO:0003993; Acid phosphatase activity.

Kozak: CCAATGT ID=cds-XP_044384983.1;Parent=rna-XM_044529048.1;Dbxref=GeneID:123107038,Genbank:XP_044384983.1;Name=XP_044384983.1;gbkey=CDS;gene=LOC123107038;product=transcription factor TB1-like;protein_id=XP_044384983.1

GO:0005634; Nucleus.

Kozak: CGAATGT ID=cds-XP_044385094.1;Parent=rna-XM_044529159.1;Dbxref=GeneID:123107155,Genbank:XP_044385094.1;Name=XP_044385094.1;gbkey=CDS;gene=LOC123107155;product=polcalcin Che a 3-like;protein_id=XP_044385094.1

GO:0005509; Calcium ion binding.

Kozak: TGTATGT ID=cds-XP_044385172.1;Parent=rna-XM_044529237.1;Dbxref=GeneID:123107241,Genbank:XP_044385172.1;Name=XP_044385172.1;gbkey=CDS;gene=LOC123107241;product=

Pirin-like protein At1g50590.

Kozak: TCCATGT ID=cds-XP_044385414.1;Parent=rna-XM_044529479.1;Dbxref=GeneID:123107492,Genbank:XP_044385414.1;Name=XP_044385414.1;gbkey=CDS;gene=LOC123107492;product=ethylene-responsive transcription factor ERF071-like;protein_id=XP_044385414.1

GO:0005634; Nucleus.

Kozak: CCAATGC ID=cds-XP_044385436.1;Parent=rna-XM_044529501.1;Dbxref=GeneID:123107519,Genbank:XP_044385436.1;Name=XP_044385436.1;gbkey=CDS;gene=LOC123107519;product=putative cyclin-F1-1;protein_id=XP_044385436.1

GO:0000307; Cyclin-dependent protein kinase holoenzyme complex.

Kozak: CCAATGC ID=cds-XP_044385437.1;Parent=rna-XM_044529502.1;Dbxref=GeneID:123107521,Genbank:XP_044385437.1;Name=XP_044385437.1;gbkey=CDS;gene=LOC123107521;product=

Putative cyclin-F1-1.

Kozak: TACATGC ID=cds-XP_044385902.1;Parent=rna-XM_044529967.1;Dbxref=GeneID:123108117,Genbank:XP_044385902.1;Name=XP_044385902.1;gbkey=CDS;gene=LOC123108117;product=

PLASMODESMATA CALLOSE-BINDING PROTEIN 3-like.

Kozak: TTCATGT ID=cds-XP_044386157.1;Parent=rna-XM_044530222.1;Dbxref=GeneID:123108431,Genbank:XP_044386157.1;Name=XP_044386157.1;gbkey=CDS;gene=LOC123108431;product=

Zinc finger BED domain-containing protein RICESLEEPER 1-like.

Kozak: TCCATGT ID=cds-XP_044386179.1;Parent=rna-XM_044530244.1;Dbxref=GeneID:123108461,Genbank:XP_044386179.1;Name=XP_044386179.1;gbkey=CDS;gene=LOC123108461;product=protein TIFY 6a-like;protein_id=XP_044386179.1

GO:0005634; Nucleus.

Kozak: TCCATGT ID=cds-XP_044386249.1;Parent=rna-XM_044530314.1;Dbxref=GeneID:123108538,Genbank:XP_044386249.1;Name=XP_044386249.1;gbkey=CDS;gene=LOC123108538;product=

E3 ubiquitin-protein ligase RFWD3-like.

Kozak: CCCATGT ID=cds-XP_044378717.1;Parent=rna-XM_044522782.1;Dbxref=GeneID:123101244,Genbank:XP_044378717.1;Name=XP_044378717.1;gbkey=CDS;gene=LOC123101244;product=

Two-component response regulator ORR5-like.

Kozak: CCAATGC ID=cds-XP_044378936.1;Parent=rna-XM_044523001.1;Dbxref=GeneID:123101645,Genbank:XP_044378936.1;Name=XP_044378936.1;gbkey=CDS;gene=LOC123101645;product=

Soyasapogenol B glucuronide galactosyltransferase-like.

Kozak: CCAATGT ID=cds-XP_044378962.1;Parent=rna-XM_044523027.1;Dbxref=GeneID:123101684,Genbank:XP_044378962.1;Name=XP_044378962.1;gbkey=CDS;gene=LOC123101684;product=protein SRG1-like;protein_id=XP_044378962.1

GO:0046872; Metal ion binding.

Kozak: CAGATGC ID=cds-XP_044379108.1;Parent=rna-XM_044523173.1;Dbxref=GeneID:123101930,Genbank:XP_044379108.1;Name=XP_044379108.1;gbkey=CDS;gene=LOC123101930;product=HIPL1 protein-like;protein_id=XP_044379108.1

GO:0016021; Integral component of membrane.

Kozak: CCAATGC ID=cds-XP_044379301.1;Parent=rna-XM_044523366.1;Dbxref=GeneID:123102082,Genbank:XP_044379301.1;Name=XP_044379301.1;gbkey=CDS;gene=LOC123102082;product=ubiquitin carboxyl-terminal hydrolase 8-like;protein_id=XP_044379301.1

GO:0004843; Thiol-dependent deubiquitinase.

Kozak: CAGATGC ID=cds-XP_044379719.1;Parent=rna-XM_044523784.1;Dbxref=GeneID:123102438,Genbank:XP_044379719.1;Name=XP_044379719.1;Note=The sequence of the model RefSeq protein was modified relative to this genomic sequence to represent the inferred CDS: added 278 bases not found in genome assembly;end_range=87935115,.;exception=annotated by transcript or proteomic data;gbkey=CDS;gene=LOC123102438;inference=similar to RNA sequence%2C mRNA (same species):INSD:JV866851.1;partial=true;product=polyubiquitin 11;protein_id=XP_044379719.1

GO:0005737; Cytoplasm.

Kozak: CCCATGT ID=cds-XP_044379856.1;Parent=rna-XM_044523921.1;Dbxref=GeneID:123102533,Genbank:XP_044379856.1;Name=XP_044379856.1;gbkey=CDS;gene=LOC123102533;product=uncharacterized protein LOC123102533 isoform X1;protein_id=XP_044379856.1

GO:0000178; Exosome (RNase complex).

Kozak: CCCATGC ID=cds-XP_044379926.1;Parent=rna-XM_044523991.1;Dbxref=GeneID:123102587,Genbank:XP_044379926.1;Name=XP_044379926.1;gbkey=CDS;gene=LOC123102587;product=

Calmodulin-binding protein 60 A-like.

Kozak: CCCATGC ID=cds-XP_044379933.1;Parent=rna-XM_044523998.1;Dbxref=GeneID:123102594,Genbank:XP_044379933.1;Name=XP_044379933.1;gbkey=CDS;gene=LOC123102594;product=

Cytochrome c oxidase subunit 6b-1-like.

Kozak: TCCATGT ID=cds-XP_044380022.1;Parent=rna-XM_044524087.1;Dbxref=GeneID:123102665,Genbank:XP_044380022.1;Name=XP_044380022.1;gbkey=CDS;gene=LOC123102665;product=AP-5 complex subunit mu-like isoform X2;protein_id=XP_044380022.1

GO:0030119; AP-type membrane coat adaptor complex.

Kozak: TACATGC ID=cds-XP_044380066.1;Parent=rna-XM_044524131.1;Dbxref=GeneID:123102711,Genbank:XP_044380066.1;Name=XP_044380066.1;gbkey=CDS;gene=LOC123102711;product=protein CLT2%2C chloroplastic-like isoform X1;protein_id=XP_044380066.1

GO:0016021; Integral component of membrane.

Kozak: CCCATGT ID=cds-XP_044380088.1;Parent=rna-XM_044524153.1;Dbxref=GeneID:123102727,Genbank:XP_044380088.1;Name=XP_044380088.1;gbkey=CDS;gene=LOC123102727;product=ureide permease 1-like;protein_id=XP_044380088.1

GO:0016021; Integral component of membrane.

Kozak: CCCATGT ID=cds-XP_044380116.1;Parent=rna-XM_044524181.1;Dbxref=GeneID:123102750,Genbank:XP_044380116.1;Name=XP_044380116.1;gbkey=CDS;gene=LOC123102750;product=protein transport protein Sec24-like CEF isoform X1;protein_id=XP_044380116.1

GO:0030127; COPII vesicle coat.

Kozak: TTCATGT ID=cds-XP_044380478.1;Parent=rna-XM_044524543.1;Dbxref=GeneID:123103058,Genbank:XP_044380478.1;Name=XP_044380478.1;gbkey=CDS;gene=LOC123103058;product=RING finger protein 10-like;protein_id=XP_044380478.1

GO:0005783; Endoplasmic reticulum.

Kozak: CAGATGC ID=cds-XP_044380584.1;Parent=rna-XM_044524649.1;Dbxref=GeneID:123103153,Genbank:XP_044380584.1;Name=XP_044380584.1;gbkey=CDS;gene=LOC123103153;product=

Bifunctional epoxide hydrolase 2-like.

Kozak: CCAATGT ID=cds-XP_044380710.1;Parent=rna-XM_044524775.1;Dbxref=GeneID:123103249,Genbank:XP_044380710.1;Name=XP_044380710.1;gbkey=CDS;gene=LOC123103249;product=uncharacterized protein LOC123103249 isoform X1;protein_id=XP_044380710.1

GO:0030014; CCR4-NOT complex.

Kozak: TCCATGT ID=cds-XP_044380759.1;Parent=rna-XM_044524824.1;Dbxref=GeneID:123103295,Genbank:XP_044380759.1;Name=XP_044380759.1;gbkey=CDS;gene=LOC123103295;product=receptor like protein kinase S.2-like;protein_id=XP_044380759.1

GO:0005886; Plasma membrane.

Kozak: CCAATGC ID=cds-XP_044380978.1;Parent=rna-XM_044525043.1;Dbxref=GeneID:123103451,Genbank:XP_044380978.1;Name=XP_044380978.1;gbkey=CDS;gene=LOC123103451;product=phosphoenolpyruvate/phosphate translocator 1%2C chloroplastic-like;protein_id=XP_044380978.1

GO:0031969; Chloroplast membrane.

Kozak: CAGATGC ID=cds-XP_044381053.1;Parent=rna-XM_044525118.1;Dbxref=GeneID:123103509,Genbank:XP_044381053.1;Name=XP_044381053.1;gbkey=CDS;gene=LOC123103509;product=pentatricopeptide repeat-containing protein At4g02750-like;protein_id=XP_044381053.1

GO:0003723; RNA binding.

Kozak: CCCATGC ID=cds-XP_044381709.1;Parent=rna-XM_044525774.1;Dbxref=GeneID:123104056,Genbank:XP_044381709.1;Name=XP_044381709.1;gbkey=CDS;gene=LOC123104056;product=uncharacterized protein LOC123104056;protein_id=XP_044381709.1

GO:0005634; Nucleus.

Kozak: CCCATGC ID=cds-XP_044382053.1;Parent=rna-XM_044526118.1;Dbxref=GeneID:123104295,Genbank:XP_044382053.1;Name=XP_044382053.1;gbkey=CDS;gene=LOC123104295;product=oleoyl-acyl carrier protein thioesterase 1%2C chloroplastic-like;protein_id=XP_044382053.1

GO:0009507; Chloroplast.

Kozak: CCCATGT ID=cds-XP_044382570.1;Parent=rna-XM_044526635.1;Dbxref=GeneID:123104757,Genbank:XP_044382570.1;Name=XP_044382570.1;gbkey=CDS;gene=LOC123104757;product=F-box/kelch-repeat protein At5g42350-like;protein_id=XP_044382570.1

GO:0019005; SCF ubiquitin ligase complex.

Kozak: CCCATGT ID=cds-XP_044382828.1;Parent=rna-XM_044526893.1;Dbxref=GeneID:123104944,Genbank:XP_044382828.1;Name=XP_044382828.1;gbkey=CDS;gene=LOC123104944;product=F-box/LRR-repeat protein 17-like isoform X1;protein_id=XP_044382828.1

GO:0005737; Cytoplasm.

Kozak: CCAATGC ID=cds-XP_044382905.1;Parent=rna-XM_044526970.1;Dbxref=GeneID:123105010,Genbank:XP_044382905.1;Name=XP_044382905.1;gbkey=CDS;gene=LOC123105010;product=photosystem II D1 precursor processing protein PSB27-H2%2C chloroplastic-like isoform X2;protein_id=XP_044382905.1

GO:0009543; Chloroplast thylakoid lumen.

Kozak: CCCATGT ID=cds-XP_044383094.1;Parent=rna-XM_044527159.1;Dbxref=GeneID:123105155,Genbank:XP_044383094.1;Name=XP_044383094.1;gbkey=CDS;gene=LOC123105155;product=probable hexosyltransferase MUCI70;protein_id=XP_044383094.1

GO:0016021; Integral component of membrane.

Kozak: CCCATGC ID=cds-XP_044383119.1;Parent=rna-XM_044527184.1;Dbxref=GeneID:123105177,Genbank:XP_044383119.1;Name=XP_044383119.1;gbkey=CDS;gene=LOC123105177;product=probable LRR receptor-like serine/threonine-protein kinase At2g16250;protein_id=XP_044383119.1

GO:0016021; Integral component of membrane.

Kozak: CCCATGT ID=cds-XP_044383456.1;Parent=rna-XM_044527521.1;Dbxref=GeneID:123105449,Genbank:XP_044383456.1;Name=XP_044383456.1;gbkey=CDS;gene=LOC123105449;product=chromatin remodeling protein EBS-like isoform X1;protein_id=XP_044383456.1

GO:0003682; Chromatin binding.

Kozak: TTAATGC ID=cds-XP_044383636.1;Parent=rna-XM_044527701.1;Dbxref=GeneID:123105611,Genbank:XP_044383636.1;Name=XP_044383636.1;gbkey=CDS;gene=LOC123105611;product=serine/threonine/tyrosine-protein kinase HT1-like;protein_id=XP_044383636.1

GO:0005737; Cytoplasm.

Kozak: CCCATGT ID=cds-XP_044383931.1;Parent=rna-XM_044527996.1;Dbxref=GeneID:123105839,Genbank:XP_044383931.1;Name=XP_044383931.1;gbkey=CDS;gene=LOC123105839;product=probable serine acetyltransferase 2;protein_id=XP_044383931.1

GO:0005737; Cytoplasm.

Kozak: CCCATGT ID=cds-XP_044383940.1;Parent=rna-XM_044528005.1;Dbxref=GeneID:123105846,Genbank:XP_044383940.1;Name=XP_044383940.1;gbkey=CDS;gene=LOC123105846;product=F-box/LRR-repeat protein 10-like isoform X1;protein_id=XP_044383940.1

GO:0019005; SCF ubiquitin ligase complex.

Kozak: CCAATGC ID=cds-XP_044384038.1;Parent=rna-XM_044528103.1;Dbxref=GeneID:123105920,Genbank:XP_044384038.1;Name=XP_044384038.1;gbkey=CDS;gene=LOC123105920;product=ABC transporter C family member 13;protein_id=XP_044384038.1

GO:0000139; Golgi membrane.

Kozak: CCAATGT ID=cds-XP_044384088.1;Parent=rna-XM_044528153.1;Dbxref=GeneID:123105973,Genbank:XP_044384088.1;Name=XP_044384088.1;gbkey=CDS;gene=LOC123105973;product=

Pentatricopeptide repeat-containing protein At1g06710%2C mitochondrial-like.

Kozak: CAGATGC ID=cds-XP_044384225.1;Parent=rna-XM_044528290.1;Dbxref=GeneID:123106085,Genbank:XP_044384225.1;Name=XP_044384225.1;gbkey=CDS;gene=LOC123106085;product=flavin-containing monooxygenase FMO GS-OX-like 2 isoform X1;protein_id=XP_044384225.1

GO:0050660; Flavin adenine dinucleotide binding.

Kozak: CCCATGC ID=cds-XP_044384271.1;Parent=rna-XM_044528336.1;Dbxref=GeneID:123106123,Genbank:XP_044384271.1;Name=XP_044384271.1;gbkey=CDS;gene=LOC123106123;product=

LRR receptor-like serine/threonine-protein kinase GSO1.

Kozak: CCAATGC ID=cds-XP_044384292.1;Parent=rna-XM_044528357.1;Dbxref=GeneID:123106140,Genbank:XP_044384292.1;Name=XP_044384292.1;gbkey=CDS;gene=LOC123106140;product=uncharacterized protein LOC123106140;protein_id=XP_044384292.1

GO:0003755; Peptidyl-prolyl cis-trans isomerase activity.

Kozak: TCCATGT ID=cds-XP_044384747.1;Parent=rna-XM_044528812.1;Dbxref=GeneID:123106723,Genbank:XP_044384747.1;Name=XP_044384747.1;gbkey=CDS;gene=LOC123106723;product=NDR1/HIN1-like protein 26;protein_id=XP_044384747.1

GO:0046658; Anchored component of plasma membrane.

Kozak. TTCATGT ID=cds-XP_044385101.1;Parent=rna-XM_044529166.1;Dbxref=GeneID:123107164,Genbank:XP_044385101.1;Name=XP_044385101.1;gbkey=CDS;gene=LOC123107164;product=uncharacterized protein LOC123107164 isoform X1;protein_id=XP_044385101.1

GO:0012505; Endomembrane system.

Kozak: TCCATGT ID=cds-XP_044385115.1;Parent=rna-XM_044529180.1;Dbxref=GeneID:123107175,Genbank:XP_044385115.1;Name=XP_044385115.1;gbkey=CDS;gene=LOC123107175;product=AT-hook motif nuclear-localized protein 17-like;protein_id=XP_044385115.1

GO:0016021; Integral component of membrane.

Kozak: CCCATGT ID=cds-XP_044385129.1;Parent=rna-XM_044529194.1;Dbxref=GeneID:123107192,Genbank:XP_044385129.1;Name=XP_044385129.1;gbkey=CDS;gene=LOC123107192;product=uncharacterized protein LOC123107192;protein_id=XP_044385129.1

GO:0016021; Integral component of membrane.

Kozak: CCAATGC ID=cds-XP_044385441.1;Parent=rna-XM_044529506.1;Dbxref=GeneID:123107525,Genbank:XP_044385441.1;Name=XP_044385441.1;gbkey=CDS;gene=LOC123107525;product=putative cyclin-F1-1;protein_id=XP_044385441.1

GO:0000307; Cyclin-dependent protein kinase holoenzyme complex.

Kozak: CCAATGC ID=cds-XP_044385442.1;Parent=rna-XM_044529507.1;Dbxref=GeneID:123107526,Genbank:XP_044385442.1;Name=XP_044385442.1;gbkey=CDS;gene=LOC123107526;product=

Putative cyclin-F1-1.

Kozak: CCCATGT ID=cds-XP_044385541.1;Parent=rna-XM_044529606.1;Dbxref=GeneID:123107645,Genbank:XP_044385541.1;Name=XP_044385541.1;gbkey=CDS;gene=LOC123107645;product=uncharacterized protein LOC123107645;protein_id=XP_044385541.1

GO:0005737; Cytoplasm.

Kozak: TACATGC ID=cds-XP_044385553.1;Parent=rna-XM_044529618.1;Dbxref=GeneID:123107670,Genbank:XP_044385553.1;Name=XP_044385553.1;gbkey=CDS;gene=LOC123107670;product=

11S globulin seed storage protein 2-like.

Kozak: CAGATGC ID=cds-XP_044386269.1;Parent=rna-XM_044530334.1;Dbxref=GeneID:123108566,Genbank:XP_044386269.1;Name=XP_044386269.1;gbkey=CDS;gene=LOC123108566;product=polyubiquitin-like;protein_id=XP_044386269.1 NA

543432:cds-XP_044382585.1,cds-XP_044382586.1 CCCATGC ID=cds-XP_044382585.1;Parent=rna-XM_044526650.1;Dbxref=GeneID:543432,Genbank:XP_044382585.1;Name=XP_044382585.1;gbkey=CDS;gene=LOC543432;product=carboxyl-terminal-processing peptidase 2%2C chloroplastic isoform X1;protein_id=XP_044382585.1

GO:0004175; Endopeptidase activity.

Kozak: CCAATGC ID=cds-XP_044422046.1;Parent=rna-XM_044566111.1;Dbxref=GeneID:123146835,Genbank:XP_044422046.1;Name=XP_044422046.1;gbkey=CDS;gene=LOC123146835;product=BTB/POZ and MATH domain-containing protein 2-like;protein_id=XP_044422046.1

GO:0016567; Protein ubiquitination.

Kozak: CCAATGC ID=cds-XP_044422257.1;Parent=rna-XM_044566322.1;Dbxref=GeneID:123147081,Genbank:XP_044422257.1;Name=XP_044422257.1;gbkey=CDS;gene=LOC123147081;product=cysteine-rich receptor-like protein kinase 25;protein_id=XP_044422257.1

GO:0005886; Plasma membrane.

Kozak: TACATGC ID=cds-XP_044422280.1;Parent=rna-XM_044566345.1;Dbxref=GeneID:123147108,Genbank:XP_044422280.1;Name=XP_044422280.1;gbkey=CDS;gene=LOC123147108;product=

Dirigent protein 20-like.

Kozak: CAGATGC ID=cds-XP_044422430.1;Parent=rna-XM_044566495.1;Dbxref=GeneID:123147248,Genbank:XP_044422430.1;Name=XP_044422430.1;gbkey=CDS;gene=LOC123147248;product=

Polyubiquitin-like.

Kozak: TACATGC ID=cds-XP_044422440.1;Parent=rna-XM_044566505.1;Dbxref=GeneID:123147264,Genbank:XP_044422440.1;Name=XP_044422440.1;gbkey=CDS;gene=LOC123147264;product=lichenase-2-like;protein_id=XP_044422440.1

GO:0046658; Anchored component of plasma membrane.

Kozak: CCCATGC ID=cds-XP_044422475.1;Parent=rna-XM_044566540.1;Dbxref=GeneID:123147313,Genbank:XP_044422475.1;Name=XP_044422475.1;gbkey=CDS;gene=LOC123147313;product=thioredoxin 1-like;protein_id=XP_044422475.1

GO:0005737; Cytoplasm.

Kozak: CGAATGT ID=cds-XP_044422862.1;Parent=rna-XM_044566927.1;Dbxref=GeneID:123147662,Genbank:XP_044422862.1;Name=XP_044422862.1;gbkey=CDS;gene=LOC123147662;product=putative pentatricopeptide repeat-containing protein At5g52630 isoform X1;protein_id=XP_044422862.1

GO:0003723; RNA binding.

Kozak: TCCATGT ID=cds-XP_044423247.1;Parent=rna-XM_044567312.1;Dbxref=GeneID:123147977,Genbank:XP_044423247.1;Name=XP_044423247.1;gbkey=CDS;gene=LOC123147977;product=kinesin-like protein KIN-14M isoform X1;protein_id=XP_044423247.1

GO:0005874; Microtubule.

Kozak: CCAATGT ID=cds-XP_044423317.1;Parent=rna-XM_044567382.1;Dbxref=GeneID:123148028,Genbank:XP_044423317.1;Name=XP_044423317.1;gbkey=CDS;gene=LOC123148028;product=uncharacterized protein LOC123148028 isoform X1;protein_id=XP_044423317.1

GO:0005524; ATP binding.

Kozak: CCCATGC ID=cds-XP_044423534.1;Parent=rna-XM_044567599.1;Dbxref=GeneID:123148220,Genbank:XP_044423534.1;Name=XP_044423534.1;gbkey=CDS;gene=LOC123148220;product=pentatricopeptide repeat-containing protein At1g18485-like;protein_id=XP_044423534.1

GO:0008270; Zinc ion binding.

Kozak: TCCATGT ID=cds-XP_044423648.1;Parent=rna-XM_044567713.1;Dbxref=GeneID:123148327,Genbank:XP_044423648.1;Name=XP_044423648.1;gbkey=CDS;gene=LOC123148327;product=nuclear intron maturase 4%2C mitochondrial-like isoform X1;protein_id=XP_044423648.1

GO:0005739; Mitochondrion.

Kozak: CCAATGC ID=cds-XP_044424040.1;Parent=rna-XM_044568105.1;Dbxref=GeneID:123148632,Genbank:XP_044424040.1;Name=XP_044424040.1;gbkey=CDS;gene=LOC123148632;product=probable cation transporter HKT9;protein_id=XP_044424040.1

GO:0016021; Integral component of membrane

Kozak: TACATGC ID=cds-XP_044425250.1;Parent=rna-XM_044569315.1;Dbxref=GeneID:123149624,Genbank:XP_044425250.1;Name=XP_044425250.1;gbkey=CDS;gene=LOC123149624;product=

Putative receptor-like protein kinase At4g00960 isoform X1.

Kozak: CCAATGT ID=cds-XP_044425445.1;Parent=rna-XM_044569510.1;Dbxref=GeneID:123149774,Genbank:XP_044425445.1;Name=XP_044425445.1;gbkey=CDS;gene=LOC123149774;product=probable LRR receptor-like serine/threonine-protein kinase At3g47570;protein_id=XP_044425445.1

GO:0005524; ATP binding.

Kozak: CCAATGT ID=cds-XP_044425577.1;Parent=rna-XM_044569642.1;Dbxref=GeneID:123149871,Genbank:XP_044425577.1;Name=XP_044425577.1;gbkey=CDS;gene=LOC123149871;product=

LysM domain-containing GPI-anchored protein LYP6-like.

Kozak: CAGATGC ID=cds-XP_044425701.1;Parent=rna-XM_044569766.1;Dbxref=GeneID:123149975,Genbank:XP_044425701.1;Name=XP_044425701.1;gbkey=CDS;gene=LOC123149975;product=

GEM-like protein 1.

Kozak: CCCATGC ID=cds-XP_044425902.1;Parent=rna-XM_044569967.1;Dbxref=GeneID:123150155,Genbank:XP_044425902.1;Name=XP_044425902.1;gbkey=CDS;gene=LOC123150155;product=nudix hydrolase 21%2C chloroplastic-like;protein_id=XP_044425902.1

GO:0005737; Cytoplasm.

Kozak: TCCATGT ID=cds-XP_044425927.1;Parent=rna-XM_044569992.1;Dbxref=GeneID:123150165,Genbank:XP_044425927.1;Name=XP_044425927.1;gbkey=CDS;gene=LOC123150165;product=protochlorophyllide-dependent translocon component 52%2C chloroplastic-like;protein_id=XP_044425927.1

GO:0009507; Chloroplast.

Kozak: TCCATGT ID=cds-XP_044426000.1;Parent=rna-XM_044570065.1;Dbxref=GeneID:123150230,Genbank:XP_044426000.1;Name=XP_044426000.1;gbkey=CDS;gene=LOC123150230;product=

Protein NOI4.

Kozak: CCAATGC ID=cds-XP_044426191.1;Parent=rna-XM_044570256.1;Dbxref=GeneID:123150402,Genbank:XP_044426191.1;Name=XP_044426191.1;gbkey=CDS;gene=LOC123150402;product=pentatricopeptide repeat-containing protein At2g03380%2C mitochondrial-like;protein_id=XP_044426191.1

GO:0003723; RNA binding.

Kozak: CCAATGC ID=cds-XP_044426213.1;Parent=rna-XM_044570278.1;Dbxref=GeneID:123150425,Genbank:XP_044426213.1;Name=XP_044426213.1;gbkey=CDS;gene=LOC123150425;product=triphosphate tunnel metalloenzyme 3-like;protein_id=XP_044426213.1

GO:0016462; Pyrophosphatase activity.

Kozak: CAGATGC ID=cds-XP_044426256.1;Parent=rna-XM_044570321.1;Dbxref=GeneID:123150475,Genbank:XP_044426256.1;Name=XP_044426256.1;gbkey=CDS;gene=LOC123150475;product=ATP-dependent Clp protease proteolytic subunit-related protein 2%2C chloroplastic-like;protein_id=XP_044426256.1

GO:0009368; Endopeptidase Clp complex

Kozak: CGAATGT ID=cds-XP_044426525.1;Parent=rna-XM_044570590.1;Dbxref=GeneID:123150761,Genbank:XP_044426525.1;Name=XP_044426525.1;gbkey=CDS;gene=LOC123150761;product=UDP-glycosyltransferase CGT-like;protein_id=XP_044426525.1

GO:0008194; UDP-glycosyltransferase activity.

Kozak: CCCATGC ID=cds-XP_044426618.1;Parent=rna-XM_044570683.1;Dbxref=GeneID:123150866,Genbank:XP_044426618.1;Name=XP_044426618.1;gbkey=CDS;gene=LOC123150866;product=

Pentatricopeptide repeat-containing protein At2g26790%2C mitochondrial-like.

Kozak: CAGATGC ID=cds-XP_044426717.1;Parent=rna-XM_044570782.1;Dbxref=GeneID:123150985,Genbank:XP_044426717.1;Name=XP_044426717.1;gbkey=CDS;gene=LOC123150985;product=

Pentatricopeptide repeat-containing protein At1g80150%2C mitochondrial-like.

Kozak: CCAATGC ID=cds-XP_044426817.1;Parent=rna-XM_044570882.1;Dbxref=GeneID:123151108,Genbank:XP_044426817.1;Name=XP_044426817.1;gbkey=CDS;gene=LOC123151108;product=

Mannan endo-1%2C4-beta-mannosidase 6-like isoform X2.

Kozak: TACATGC ID=cds-XP_044427338.1;Parent=rna-XM_044571403.1;Dbxref=GeneID:123151743,Genbank:XP_044427338.1;Name=XP_044427338.1;gbkey=CDS;gene=LOC123151743;product=proline-rich receptor-like protein kinase PERK12;protein_id=XP_044427338.1

GO:0016021; Integral component of membrane.

Kozak: CCCATGC ID=cds-XP_044428226.1;Parent=rna-XM_044572291.1;Dbxref=GeneID:123152917,Genbank:XP_044428226.1;Name=XP_044428226.1;gbkey=CDS;gene=LOC123152917;product=

Elongator complex protein 4-like.

Kozak: TTAATGC ID=cds-XP_044428378.1;Parent=rna-XM_044572443.1;Dbxref=GeneID:123153225,Genbank:XP_044428378.1;Name=XP_044428378.1;gbkey=CDS;gene=LOC123153225;product=autophagy-related protein 2-like;protein_id=XP_044428378.1

GO:0005789; Endoplasmic reticulum membrane.

Kozak: CCAATGT ID=cds-XP_044428403.1;Parent=rna-XM_044572468.1;Dbxref=GeneID:123153268,Genbank:XP_044428403.1;Name=XP_044428403.1;gbkey=CDS;gene=LOC123153268;product=LRR receptor-like serine/threonine-protein kinase RGI3;protein_id=XP_044428403.1

GO:0016021; Integral component of membrane.

Kozak: TCCATGT ID=cds-XP_044428806.1;Parent=rna-XM_044572871.1;Dbxref=GeneID:123154069,Genbank:XP_044428806.1;Name=XP_044428806.1;gbkey=CDS;gene=LOC123154069;product=

F-box protein At5g07610-like.

Kozak: TCCATGT ID=cds-XP_044428970.1;Parent=rna-XM_044573035.1;Dbxref=GeneID:123154264,Genbank:XP_044428970.1;Name=XP_044428970.1;gbkey=CDS;gene=LOC123154264;product=probable transcriptional regulator SLK3 isoform X1;protein_id=XP_044428970.1

GO:0005634; Nucleus.

Kozak: TCCATGT ID=cds-XP_044429031.1;Parent=rna-XM_044573096.1;Dbxref=GeneID:123154341,Genbank:XP_044429031.1;Name=XP_044429031.1;gbkey=CDS;gene=LOC123154341;product=

F-box protein At5g07610-like.

Kozak: TCCATGT ID=cds-XP_044429293.1;Parent=rna-XM_044573358.1;Dbxref=GeneID:123154703,Genbank:XP_044429293.1;Name=XP_044429293.1;gbkey=CDS;gene=LOC123154703;product=

Cell division cycle-associated protein 7-like.

Kozak: CCCATGT ID=cds-XP_044423587.1;Parent=rna-XM_044567652.1;Dbxref=GeneID:100240700,Genbank:XP_044423587.1;Name=XP_044423587.1;gbkey=CDS;gene=LOC100240700;product=DNA repair protein RAD51 homolog B;protein_id=XP_044423587.1

GO:0000794; Condensed nuclear chromosome.

Kozak: CCAATGT ID=cds-XP_044422573.1;Parent=rna-XM_044566638.1;Dbxref=GeneID:123147401,Genbank:XP_044422573.1;Name=XP_044422573.1;gbkey=CDS;gene=LOC123147401;product=probable transcription factor At5g61620 isoform X1;protein_id=XP_044422573.1

GO:0003677; DNA binding.

Kozak: CCAATGC ID=cds-XP_044422812.1;Parent=rna-XM_044566877.1;Dbxref=GeneID:123147613,Genbank:XP_044422812.1;Name=XP_044422812.1;gbkey=CDS;gene=LOC123147613;product=serine/threonine-protein kinase-like protein CCR4;protein_id=XP_044422812.1

GO:0016021; Integral component of membrane.

Kozak: CCAATGT ID=cds-XP_044422989.1;Parent=rna-XM_044567054.1;Dbxref=GeneID:123147748,Genbank:XP_044422989.1;Name=XP_044422989.1;gbkey=CDS;gene=LOC123147748;product=anthocyanidin 5%2C3-O-glucosyltransferase-like;protein_id=XP_044422989.1

GO:0005634; Nucleus.

Kozak: CCAATGC ID=cds-XP_044423058.1;Parent=rna-XM_044567123.1;Dbxref=GeneID:123147812,Genbank:XP_044423058.1;Name=XP_044423058.1;gbkey=CDS;gene=LOC123147812;product=protein MICRORCHIDIA 2-like;protein_id=XP_044423058.1

GO:0005634; Nucleus.

Kozak: CAGATGC ID=cds-XP_044423388.1;Parent=rna-XM_044567453.1;Dbxref=GeneID:123148098,Genbank:XP_044423388.1;Name=XP_044423388.1;gbkey=CDS;gene=LOC123148098;product=

Diphosphomevalonate decarboxylase MVD1%2C peroxisomal-like.

Kozak: TTAATGC ID=cds-XP_044423571.1;Parent=rna-XM_044567636.1;Dbxref=GeneID:123148248,Genbank:XP_044423571.1;Name=XP_044423571.1;gbkey=CDS;gene=LOC123148248;product=MLO-like protein 13 isoform X2;protein_id=XP_044423571.1

GO:0016021; Integral component of membrane.

Kozak: TCCATGT ID=cds-XP_044423782.1;Parent=rna-XM_044567847.1;Dbxref=GeneID:123148417,Genbank:XP_044423782.1;Name=XP_044423782.1;gbkey=CDS;gene=LOC123148417;product=probable transcriptional regulator SLK3;protein_id=XP_044423782.1

GO:0005634; Nucleus.

Kozak: TCCATGT ID=cds-XP_044423857.1;Parent=rna-XM_044567922.1;Dbxref=GeneID:123148497,Genbank:XP_044423857.1;Name=XP_044423857.1;Note=The sequence of the model RefSeq protein was modified relative to this genomic sequence to represent the inferred CDS: added 41 bases not found in genome assembly;exception=annotated by transcript or proteomic data;gbkey=CDS;gene=LOC123148497;inference=similar to RNA sequence (same species):INSD:GFFI01006612.1;partial=true;product=ubiquitin carboxyl-terminal hydrolase MINDY-2-like;protein_id=XP_044423857.1

GO:0016807; Cysteine-type carboxypeptidase activity.

Kozak: CCCATGC ID=cds-XP_044423878.1;Parent=rna-XM_044567943.1;Dbxref=GeneID:123148514,Genbank:XP_044423878.1;Name=XP_044423878.1;gbkey=CDS;gene=LOC123148514;product=superoxide dismutase [Fe] 2%2C chloroplastic-like isoform X1;protein_id=XP_044423878.1

GO:0042644; Chloroplast nucleoid.

Kozak: CAGATGC ID=cds-XP_044424296.1;Parent=rna-XM_044568361.1;Dbxref=GeneID:123148846,Genbank:XP_044424296.1;Name=XP_044424296.1;gbkey=CDS;gene=LOC123148846;product=uncharacterized protein LOC123148846;protein_id=XP_044424296.1

GO:0005681; Spliceosomal complex.

Kozak: CAGATGC ID=cds-XP_044424464.1;Parent=rna-XM_044568529.1;Dbxref=GeneID:123148995,Genbank:XP_044424464.1;Name=XP_044424464.1;Note=The sequence of the model RefSeq protein was modified relative to this genomic sequence to represent the inferred CDS: added 68 bases not found in genome assembly;end_range=652068484,.;exception=annotated by transcript or proteomic data;gbkey=CDS;gene=LOC123148995;inference=similar to RNA sequence%2C mRNA (same species):INSD:AK446922.1;partial=true;product=polyubiquitin 11;protein_id=XP_044424464.1

GO:0005737; Cytoplasm.

Kozak: CAGATGC ID=cds-XP_044424466.1;Parent=rna-XM_044568531.1;Dbxref=GeneID:123149000,Genbank:XP_044424466.1;Name=XP_044424466.1;gbkey=CDS;gene=LOC123149000;product=polyubiquitin;protein_id=XP_044424466.1

GO:0005737; Cytoplasm.

Kozak: CAGATGC ID=cds-XP_044424683.1;Parent=rna-XM_044568748.1;Dbxref=GeneID:123149191,Genbank:XP_044424683.1;Name=XP_044424683.1;gbkey=CDS;gene=LOC123149191;product=

Protein FAR1-RELATED SEQUENCE 7-like isoform X1.

Kozak: CCAATGC ID=cds-XP_044424776.1;Parent=rna-XM_044568841.1;Dbxref=GeneID:123149232,Genbank:XP_044424776.1;Name=XP_044424776.1;gbkey=CDS;gene=LOC123149232;product=probable galactinol--sucrose galactosyltransferase 6;protein_id=XP_044424776.1

GO:0003824; Catalytic activity.

Kozak: TACATGC ID=cds-XP_044424860.1;Parent=rna-XM_044568925.1;Dbxref=GeneID:123149289,Genbank:XP_044424860.1;Name=XP_044424860.1;gbkey=CDS;gene=LOC123149289;product=tRNA pseudouridine synthase A-like isoform X1;protein_id=XP_044424860.1

GO:0009982; Pseudouridine synthase activity.

Kozak: TCCATGT ID=cds-XP_044425188.1;Parent=rna-XM_044569253.1;Dbxref=GeneID:123149570,Genbank:XP_044425188.1;Name=XP_044425188.1;gbkey=CDS;gene=LOC123149570;product=probable transcriptional regulator SLK2;protein_id=XP_044425188.1

GO:0005634; Nucleus.

Kozak: CCCATGT ID=cds-XP_044425219.1;Parent=rna-XM_044569284.1;Dbxref=GeneID:123149595,Genbank:XP_044425219.1;Name=XP_044425219.1;gbkey=CDS;gene=LOC123149595;product=

Formin-like protein 20 isoform X1.

Kozak: CCAATGC ID=cds-XP_044426004.1;Parent=rna-XM_044570069.1;Dbxref=GeneID:123150236,Genbank:XP_044426004.1;Name=XP_044426004.1;gbkey=CDS;gene=LOC123150236;product=

Fibronectin-binding protein A-like isoform X1.

Kozak: CCCATGC ID=cds-XP_044426062.1;Parent=rna-XM_044570127.1;Dbxref=GeneID:123150290,Genbank:XP_044426062.1;Name=XP_044426062.1;gbkey=CDS;gene=LOC123150290;product=T-complex protein 1 subunit alpha-like;protein_id=XP_044426062.1

GO:0005832; Chaperonin-containing T-complex.

Kozak: CAGATGC ID=cds-XP_044426253.1;Parent=rna-XM_044570318.1;Dbxref=GeneID:123150470,Genbank:XP_044426253.1;Name=XP_044426253.1;gbkey=CDS;gene=LOC123150470;product=

Pentatricopeptide repeat-containing protein At3g29230-like.

Kozak: CCCATGC ID=cds-XP_044426296.1;Parent=rna-XM_044570361.1;Dbxref=GeneID:123150521,Genbank:XP_044426296.1;Name=XP_044426296.1;gbkey=CDS;gene=LOC123150521;product=

Mucin-2-like.

Kozak: CCAATGC ID=cds-XP_044427478.1;Parent=rna-XM_044571543.1;Dbxref=GeneID:123151910,Genbank:XP_044427478.1;Name=XP_044427478.1;gbkey=CDS;gene=LOC123151910;product=fucosyltransferase 2-like;protein_id=XP_044427478.1

GO:0032580; Golgi cisterna membrane.

Kozak: CCCATGC ID=cds-XP_044427720.1;Parent=rna-XM_044571785.1;Dbxref=GeneID:123152163,Genbank:XP_044427720.1;Name=XP_044427720.1;gbkey=CDS;gene=LOC123152163;product=serine/threonine/tyrosine-protein kinase HT1-like;protein_id=XP_044427720.1

GO:0005524; ATP binding.

Kozak: CCAATGT ID=cds-XP_044427796.1;Parent=rna-XM_044571861.1;Dbxref=GeneID:123152272,Genbank:XP_044427796.1;Name=XP_044427796.1;gbkey=CDS;gene=LOC123152272;product=

Protein ALP1-like.

Kozak: CCAATGC ID=cds-XP_044428498.1;Parent=rna-XM_044572563.1;Dbxref=GeneID:123153440,Genbank:XP_044428498.1;Name=XP_044428498.1;gbkey=CDS;gene=LOC123153440;product=RING-H2 finger protein ATL39-like;protein_id=XP_044428498.1

GO:0016021; Integral component of membrane.

Kozak: CAGATGC ID=cds-XP_044428720.1;Parent=rna-XM_044572785.1;Dbxref=GeneID:123153885,Genbank:XP_044428720.1;Name=XP_044428720.1;gbkey=CDS;gene=LOC123153885;product=uncharacterized protein LOC123153885;protein_id=XP_044428720.1

GO:0016021; Integral component of membrane.

Kozak: CCAATGC ID=cds-XP_044428863.1;Parent=rna-XM_044572928.1;Dbxref=GeneID:123154131,Genbank:XP_044428863.1;Name=XP_044428863.1;gbkey=CDS;gene=LOC123154131;product=

Basic leucine zipper 19-like.

Kozak: CAGATGC ID=cds-XP_044428924.1;Parent=rna-XM_044572989.1;Dbxref=GeneID:123154211,Genbank:XP_044428924.1;Name=XP_044428924.1;gbkey=CDS;gene=LOC123154211;product=cytochrome P450 72A397-like;protein_id=XP_044428924.1

GO:0016021; Integral component of membrane.

Kozak: TCCATGT ID=cds-XP_044428995.1;Parent=rna-XM_044573060.1;Dbxref=GeneID:123154303,Genbank:XP_044428995.1;Name=XP_044428995.1;gbkey=CDS;gene=LOC123154303;product=NDR1/HIN1-like protein 26;protein_id=XP_044428995.1

GO:0046658; Anchored component of plasma membrane.

Kozak: CCAATGT ID=cds-XP_044422879.1;Parent=rna-XM_044566944.1;Dbxref=GeneID:543490,Genbank:XP_044422879.1;Name=XP_044422879.1;gbkey=CDS;gene=LOC543490;product=glycine-rich RNA-binding protein RZ1A;protein_id=XP_044422879.1

GO:0003723; RNA binding.

Kozak: CAGATGC ID=cds-XP_044425026.1;Parent=rna-XM_044569091.1;Dbxref=GeneID:780603,Genbank:XP_044425026.1;Name=XP_044425026.1;gbkey=CDS;gene=LOC780603;product=60S ribosomal protein L2%2C mitochondrial isoform X2;protein_id=XP_044425026.1

GO:0005762; Mitochondrial large ribosomal subunit.

Kozak: CAGATGC ID=cds-XP_044430391.1;Parent=rna-XM_044574456.1;Dbxref=GeneID:123156317,Genbank:XP_044430391.1;Name=XP_044430391.1;gbkey=CDS;gene=LOC123156317;product=

Pentatricopeptide repeat-containing protein At1g80150%2C mitochondrial-like.

Kozak: TCCATGT ID=cds-XP_044430401.1;Parent=rna-XM_044574466.1;Dbxref=GeneID:123156332,Genbank:XP_044430401.1;Name=XP_044430401.1;gbkey=CDS;gene=LOC123156332;product=

Cell division cycle-associated protein 7-like.

Kozak: CCCATGC ID=cds-XP_044430430.1;Parent=rna-XM_044574495.1;Dbxref=GeneID:123156357,Genbank:XP_044430430.1;Name=XP_044430430.1;gbkey=CDS;gene=LOC123156357;product=receptor-like serine/threonine-protein kinase SD1-6;protein_id=XP_044430430.1

GO:0005524; ATP binding.

Kozak: CAGATGC ID=cds-XP_044430922.1;Parent=rna-XM_044574987.1;Dbxref=GeneID:123156801,Genbank:XP_044430922.1;Name=XP_044430922.1;gbkey=CDS;gene=LOC123156801;product=ubiquitin;protein_id=XP_044430922.1

GO:0005737; Cytoplasm.

Kozak: CCCATGT ID=cds-XP_044431317.1;Parent=rna-XM_044575382.1;Dbxref=GeneID:123157137,Genbank:XP_044431317.1;Name=XP_044431317.1;gbkey=CDS;gene=LOC123157137;product=

Eukaryotic translation initiation factor 5B-like.

Kozak: CCCATGC ID=cds-XP_044431372.1;Parent=rna-XM_044575437.1;Dbxref=GeneID:123157183,Genbank:XP_044431372.1;Name=XP_044431372.1;gbkey=CDS;gene=LOC123157183;product=21 kDa protein-like;protein_id=XP_044431372.1

GO:0004857; Enzyme inhibitor activity.

Kozak: CCAATGT ID=cds-XP_044431548.1;Parent=rna-XM_044575613.1;Dbxref=GeneID:123157352,Genbank:XP_044431548.1;Name=XP_044431548.1;gbkey=CDS;gene=LOC123157352;product=

LysM domain-containing GPI-anchored protein LYP6-like.

Kozak: CCCATGC ID=cds-XP_044431574.1;Parent=rna-XM_044575639.1;Dbxref=GeneID:123157374,Genbank:XP_044431574.1;Name=XP_044431574.1;gbkey=CDS;gene=LOC123157374;product=

Pentatricopeptide repeat-containing protein At2g26790%2C mitochondrial-like.

Kozak: CCCATGC ID=cds-XP_044431828.1;Parent=rna-XM_044575893.1;Dbxref=GeneID:123157634,Genbank:XP_044431828.1;Name=XP_044431828.1;gbkey=CDS;gene=LOC123157634;product=probable histidine kinase 2;protein_id=XP_044431828.1

GO:0016021; Integral component of membrane.

Kozak: CCCATGC ID=cds-XP_044431896.1;Parent=rna-XM_044575961.1;Dbxref=GeneID:123157733,Genbank:XP_044431896.1;Name=XP_044431896.1;gbkey=CDS;gene=LOC123157733;product=galacturonosyltransferase 8-like;protein_id=XP_044431896.1

GO:0000139; Golgi membrane.

Kozak: CCCATGT ID=cds-XP_044432153.1;Parent=rna-XM_044576218.1;Dbxref=GeneID:123158091,Genbank:XP_044432153.1;Name=XP_044432153.1;gbkey=CDS;gene=LOC123158091;product=

Extensin-like.

Kozak: CCCATGC ID=cds-XP_044432162.1;Parent=rna-XM_044576227.1;Dbxref=GeneID:123158104,Genbank:XP_044432162.1;Name=XP_044432162.1;gbkey=CDS;gene=LOC123158104;product=U5 small nuclear ribonucleoprotein 40 kDa protein-like;protein_id=XP_044432162.1

GO:0071013; Catalytic step 2 spliceosome.

Kozak: CCAATGC ID=cds-XP_044432281.1;Parent=rna-XM_044576346.1;Dbxref=GeneID:123158300,Genbank:XP_044432281.1;Name=XP_044432281.1;gbkey=CDS;gene=LOC123158300;product=probable cation transporter HKT9;protein_id=XP_044432281.1

GO:0016021; Integral component of membrane.

Kozak: TTCATGT ID=cds-XP_044432400.1;Parent=rna-XM_044576465.1;Dbxref=GeneID:123158508,Genbank:XP_044432400.1;Name=XP_044432400.1;gbkey=CDS;gene=LOC123158508;product=

Translation initiation factor IF-2-like.

Kozak: CCAATGC ID=cds-XP_044432616.1;Parent=rna-XM_044576681.1;Dbxref=GeneID:123158861,Genbank:XP_044432616.1;Name=XP_044432616.1;gbkey=CDS;gene=LOC123158861;product=phosphoenolpyruvate phosphatase-like;protein_id=XP_044432616.1

GO:0003993; Acid phosphatase activity.

Kozak: CAGATGC ID=cds-XP_044432969.1;Parent=rna-XM_044577034.1;Dbxref=GeneID:123159204,Genbank:XP_044432969.1;Name=XP_044432969.1;gbkey=CDS;gene=LOC123159204;product=polyubiquitin;protein_id=XP_044432969.1

GO:0005737; Cytoplasm.

Kozak: TTAATGC ID=cds-XP_044433201.1;Parent=rna-XM_044577266.1;Dbxref=GeneID:123159431,Genbank:XP_044433201.1;Name=XP_044433201.1;gbkey=CDS;gene=LOC123159431;product=pentatricopeptide repeat-containing protein At2g37320-like isoform X1;protein_id=XP_044433201.1

GO:0003723; RNA binding.

Kozak: CAGATGC ID=cds-XP_044433264.1;Parent=rna-XM_044577329.1;Dbxref=GeneID:123159493,Genbank:XP_044433264.1;Name=XP_044433264.1;gbkey=CDS;gene=LOC123159493;product=

GEM-like protein 1.

Kozak: CCAATGT ID=cds-XP_044433523.1;Parent=rna-XM_044577588.1;Dbxref=GeneID:123159778,Genbank:XP_044433523.1;Name=XP_044433523.1;gbkey=CDS;gene=LOC123159778;product=UDP-glycosyltransferase CGT-like;protein_id=XP_044433523.1

GO:0008194; UDP-glycosyltransferase activity.

Kozak: CCAATGC ID=cds-XP_044433845.1;Parent=rna-XM_044577910.1;Dbxref=GeneID:123160090,Genbank:XP_044433845.1;Name=XP_044433845.1;gbkey=CDS;gene=LOC123160090;product=mannan endo-1%2C4-beta-mannosidase 6-like;protein_id=XP_044433845.1

GO:0016985; Mannan endo-1,4-beta-mannosidase activity.

Kozak: TACATGC ID=cds-XP_044434391.1;Parent=rna-XM_044578456.1;Dbxref=GeneID:123160632,Genbank:XP_044434391.1;Name=XP_044434391.1;gbkey=CDS;gene=LOC123160632;product=60S ribosomal protein L36-2-like;protein_id=XP_044434391.1

GO:0022625; Cytosolic large ribosomal subunit.

Kozak: CCCATGC ID=cds-XP_044434674.1;Parent=rna-XM_044578739.1;Dbxref=GeneID:123160892,Genbank:XP_044434674.1;Name=XP_044434674.1;gbkey=CDS;gene=LOC123160892;product=pentatricopeptide repeat-containing protein At1g18485-like;protein_id=XP_044434674.1

GO:0008270; Zinc ion binding.

Kozak: CAGATGC ID=cds-XP_044434996.1;Parent=rna-XM_044579061.1;Dbxref=GeneID:123161213,Genbank:XP_044434996.1;Name=XP_044434996.1;gbkey=CDS;gene=LOC123161213;product=

Putative F-box/LRR-repeat protein 23.

Kozak: CAGATGC ID=cds-XP_044435047.1;Parent=rna-XM_044579112.1;Dbxref=GeneID:123161257,Genbank:XP_044435047.1;Name=XP_044435047.1;gbkey=CDS;gene=LOC123161257;product=

Zinc finger BED domain-containing protein RICESLEEPER 2-like.

Kozak: CCAATGC ID=cds-XP_044435441.1;Parent=rna-XM_044579506.1;Dbxref=GeneID:123161695,Genbank:XP_044435441.1;Name=XP_044435441.1;gbkey=CDS;gene=LOC123161695;product=uncharacterized protein LOC123161695 isoform X1;protein_id=XP_044435441.1

GO:0016021; Integral component of membrane.

Kozak: CAGATGC ID=cds-XP_044435635.1;Parent=rna-XM_044579700.1;Dbxref=GeneID:123161887,Genbank:XP_044435635.1;Name=XP_044435635.1;gbkey=CDS;gene=LOC123161887;product=uncharacterized protein LOC123161887 isoform X1;protein_id=XP_044435635.1

GO:0016021; Integral component of membrane.

Kozak: CAGATGC ID=cds-XP_044435895.1;Parent=rna-XM_044579960.1;Dbxref=GeneID:123162169,Genbank:XP_044435895.1;Name=XP_044435895.1;gbkey=CDS;gene=LOC123162169;product=

Polyubiquitin-like.

Kozak: CCAATGC ID=cds-XP_044435925.1;Parent=rna-XM_044579990.1;Dbxref=GeneID:123162200,Genbank:XP_044435925.1;Name=XP_044435925.1;gbkey=CDS;gene=LOC123162200;product=LRR receptor-like serine/threonine-protein kinase RGI3;protein_id=XP_044435925.1

GO:0016021; Integral component of membrane.

Kozak: TACATGC ID=cds-XP_044436236.1;Parent=rna-XM_044580301.1;Dbxref=GeneID:123162524,Genbank:XP_044436236.1;Name=XP_044436236.1;Note=The sequence of the model RefSeq protein was modified relative to this genomic sequence to represent the inferred CDS: added 1048 bases not found in genome assembly;end_range=627103219,.;exception=annotated by transcript or proteomic data;gbkey=CDS;gene=LOC123162524;inference=similar to RNA sequence (same species):INSD:GIJS01025835.1;partial=true;product=

Protein root UVB sensitive 5-like.

Kozak: TCCATGT ID=cds-XP_044436383.1;Parent=rna-XM_044580448.1;Dbxref=GeneID:123162671,Genbank:XP_044436383.1;Name=XP_044436383.1;gbkey=CDS;gene=LOC123162671;product=

Probable transcriptional regulator SLK3 isoform X1.

Kozak: CCAATGT ID=cds-XP_044436557.1;Parent=rna-XM_044580622.1;Dbxref=GeneID:123162866,Genbank:XP_044436557.1;Name=XP_044436557.1;gbkey=CDS;gene=LOC123162866;product=anthocyanidin 5%2C3-O-glucosyltransferase-like;protein_id=XP_044436557.1

GO:0005634; Nucleus.

Kozak: TCCATGT ID=cds-XP_044429576.1;Parent=rna-XM_044573641.1;Dbxref=GeneID:123155464,Genbank:XP_044429576.1;Name=XP_044429576.1;gbkey=CDS;gene=LOC123155464;product=paired amphipathic helix protein Sin3-like 3;protein_id=XP_044429576.1

GO:0016021; Integral component of membrane.

Kozak: TACATGC ID=cds-XP_044429645.1;Parent=rna-XM_044573710.1;Dbxref=GeneID:123155561,Genbank:XP_044429645.1;Name=XP_044429645.1;gbkey=CDS;gene=LOC123155561;product=tRNA pseudouridine synthase A-like isoform X1;protein_id=XP_044429645.1

GO:0009982; Pseudouridine synthase activity.

Kozak: TCCATGT ID=cds-XP_044430204.1;Parent=rna-XM_044574269.1;Dbxref=GeneID:123156101,Genbank:XP_044430204.1;Name=XP_044430204.1;gbkey=CDS;gene=LOC123156101;product=probable transcriptional regulator SLK2;protein_id=XP_044430204.1

GO:0005634; Nucleus.

Kozak: CAGATGC ID=cds-XP_044430210.1;Parent=rna-XM_044574275.1;Dbxref=GeneID:123156106,Genbank:XP_044430210.1;Name=XP_044430210.1;Note=The sequence of the model RefSeq protein was modified relative to this genomic sequence to represent the inferred CDS: added 218 bases not found in genome assembly;end_range=617339427,.;exception=annotated by transcript or proteomic data;gbkey=CDS;gene=LOC123156106;inference=similar to RNA sequence%2C mRNA (same species):INSD:JP824260.1;partial=true;product=polyubiquitin;protein_id=XP_044430210.1GO:0005737; Cytoplasm.

Kozak: CCAATGC ID=cds-XP_044430219.1;Parent=rna-XM_044574284.1;Dbxref=GeneID:123156117,Genbank:XP_044430219.1;Name=XP_044430219.1;gbkey=CDS;gene=LOC123156117;product=

Splicing factor 3B subunit 2-like isoform X1.

Kozak: TTCATGT ID=cds-XP_044430380.1;Parent=rna-XM_044574445.1;Dbxref=GeneID:123156309,Genbank:XP_044430380.1;Name=XP_044430380.1;gbkey=CDS;gene=LOC123156309;product=protein S-acyltransferase 24-like;protein_id=XP_044430380.1

GO:0000139; Golgi membrane.

Kozak: CCAATGC ID=cds-XP_044430497.1;Parent=rna-XM_044574562.1;Dbxref=GeneID:123156432,Genbank:XP_044430497.1;Name=XP_044430497.1;gbkey=CDS;gene=LOC123156432;product=factor of DNA methylation 1-like isoform X1;protein_id=XP_044430497.1

GO:0080188; Gene silencing by RNA-directed DNA methylation.

Kozak: CCCATGT ID=cds-XP_044430780.1;Parent=rna-XM_044574845.1;Dbxref=GeneID:123156684,Genbank:XP_044430780.1;Name=XP_044430780.1;gbkey=CDS;gene=LOC123156684;product=

Formin-like protein 20.

Kozak: CCCATGT ID=cds-XP_044430781.1;Parent=rna-XM_044574846.1;Dbxref=GeneID:123156685,Genbank:XP_044430781.1;Name=XP_044430781.1;gbkey=CDS;gene=LOC123156685;product=

Formin-like protein 20.

Kozak: CCCATGT ID=cds-XP_044430782.1;Parent=rna-XM_044574847.1;Dbxref=GeneID:123156686,Genbank:XP_044430782.1;Name=XP_044430782.1;gbkey=CDS;gene=LOC123156686;product=

Formin-like protein 20.

Kozak: TCCATGT ID=cds-XP_044430956.1;Parent=rna-XM_044575021.1;Dbxref=GeneID:123156835,Genbank:XP_044430956.1;Name=XP_044430956.1;gbkey=CDS;gene=LOC123156835;product=uncharacterized protein LOC123156835 isoform X1;protein_id=XP_044430956.1

GO:0005634; Nucleus.

Kozak: CCCATGT ID=cds-XP_044430972.1;Parent=rna-XM_044575037.1;Dbxref=GeneID:123156851,Genbank:XP_044430972.1;Name=XP_044430972.1;gbkey=CDS;gene=LOC123156851;product=afadin- and alpha-actinin-binding protein-like isoform X1;protein_id=XP_044430972.1

GO:0110165; Cellular anatomical entity.

Kozak: CCAATGC ID=cds-XP_044430974.1;Parent=rna-XM_044575039.1;Dbxref=GeneID:123156852,Genbank:XP_044430974.1;Name=XP_044430974.1;gbkey=CDS;gene=LOC123156852;product=protein MICRORCHIDIA 1-like;protein_id=XP_044430974.1

GO:0005634; Nucleus.

Kozak: TACATGC ID=cds-XP_044431842.1;Parent=rna-XM_044575907.1;Dbxref=GeneID:123157654,Genbank:XP_044431842.1;Name=XP_044431842.1;gbkey=CDS;gene=LOC123157654;product=

Aspartic proteinase nepenthesin-1-like.

Kozak: TTAATGC ID=cds-XP_044432157.1;Parent=rna-XM_044576222.1;Dbxref=GeneID:123158095,Genbank:XP_044432157.1;Name=XP_044432157.1;gbkey=CDS;gene=LOC123158095;product=

Vesicle-associated protein 2-2-like.

Kozak: CCCATGT ID=cds-XP_044432374.1;Parent=rna-XM_044576439.1;Dbxref=GeneID:123158453,Genbank:XP_044432374.1;Name=XP_044432374.1;gbkey=CDS;gene=LOC123158453;product=GDSL esterase/lipase At1g28600-like;protein_id=XP_044432374.1

GO:0016788; Hydrolase activity, acting on ester bonds.

Kozak: CCCATGC ID=cds-XP_044432379.1;Parent=rna-XM_044576444.1;Dbxref=GeneID:123158460,Genbank:XP_044432379.1;Name=XP_044432379.1;gbkey=CDS;gene=LOC123158460;product=

Probable histone H2AXb.

Kozak: CAGATGC ID=cds-XP_044432380.1;Parent=rna-XM_044576445.1;Dbxref=GeneID:123158461,Genbank:XP_044432380.1;Name=XP_044432380.1;gbkey=CDS;gene=LOC123158461;product=

Probable histone H2AXb.

Kozak: TACATGC ID=cds-XP_044432475.1;Parent=rna-XM_044576540.1;Dbxref=GeneID:123158624,Genbank:XP_044432475.1;Name=XP_044432475.1;gbkey=CDS;gene=LOC123158624;product=

Glutathione S-transferase 1-like.

Kozak: CAGATGC ID=cds-XP_044432626.1;Parent=rna-XM_044576691.1;Dbxref=GeneID:123158869,Genbank:XP_044432626.1;Name=XP_044432626.1;gbkey=CDS;gene=LOC123158869;product=cytochrome P450 72A397-like;protein_id=XP_044432626.1

GO:0016021; Integral component of membrane.

Kozak: TCCATGT ID=cds-XP_044432785.1;Parent=rna-XM_044576850.1;Dbxref=GeneID:123159049,Genbank:XP_044432785.1;Name=XP_044432785.1;gbkey=CDS;gene=LOC123159049;product=ubiquitin carboxyl-terminal hydrolase MINDY-2-like isoform X1;protein_id=XP_044432785.1

GO:0016807; Cysteine-type carboxypeptidase activity.

Kozak: CCCATGC ID=cds-XP_044432950.1;Parent=rna-XM_044577015.1;Dbxref=GeneID:123159186,Genbank:XP_044432950.1;Name=XP_044432950.1;gbkey=CDS;gene=LOC123159186;product=uncharacterized protein LOC123159186;protein_id=XP_044432950.1

GO:0016021; Integral component of membrane.

Kozak: CAGATGC ID=cds-XP_044433073.1;Parent=rna-XM_044577138.1;Dbxref=GeneID:123159311,Genbank:XP_044433073.1;Name=XP_044433073.1;gbkey=CDS;gene=LOC123159311;product=uncharacterized protein LOC123159311;protein_id=XP_044433073.1

GO:0008168; Methyltransferase activity.

Kozak: TTCATGT ID=cds-XP_044433156.1;Parent=rna-XM_044577221.1;Dbxref=GeneID:123159396,Genbank:XP_044433156.1;Name=XP_044433156.1;gbkey=CDS;gene=LOC123159396;product=

Sentrin-specific protease-like.

Kozak: CCCATGC ID=cds-XP_044433181.1;Parent=rna-XM_044577246.1;Dbxref=GeneID:123159417,Genbank:XP_044433181.1;Name=XP_044433181.1;gbkey=CDS;gene=LOC123159417;product=mitogen-activated protein kinase kinase kinase 1;protein_id=XP_044433181.1

GO:0061630; Ubiquitin protein ligase activity.

Kozak: CCCATGC ID=cds-XP_044433255.1;Parent=rna-XM_044577320.1;Dbxref=GeneID:123159481,Genbank:XP_044433255.1;Name=XP_044433255.1;gbkey=CDS;gene=LOC123159481;product=serine/threonine/tyrosine-protein kinase HT1-like;protein_id=XP_044433255.1

GO:0005524; ATP binding.

Kozak: CCAATGC ID=cds-XP_044433899.1;Parent=rna-XM_044577964.1;Dbxref=GeneID:123160148,Genbank:XP_044433899.1;Name=XP_044433899.1;gbkey=CDS;gene=LOC123160148;product=transcription factor bHLH48-like;protein_id=XP_044433899.1

GO:0005634; Nucleus.

Kozak: CCAATGC ID=cds-XP_044433949.1;Parent=rna-XM_044578014.1;Dbxref=GeneID:123160203,Genbank:XP_044433949.1;Name=XP_044433949.1;gbkey=CDS;gene=LOC123160203;product=fucosyltransferase 2-like;protein_id=XP_044433949.1

GO:0032580; Golgi cisterna membrane.

Kozak: TACATGC ID=cds-XP_044434229.1;Parent=rna-XM_044578294.1;Dbxref=GeneID:123160482,Genbank:XP_044434229.1;Name=XP_044434229.1;gbkey=CDS;gene=LOC123160482;product=carbonic anhydrase%2C chloroplastic-like;protein_id=XP_044434229.1

GO:0004089; Carbonate dehydratase activity.

Kozak: TTAATGC ID=cds-XP_044434372.1;Parent=rna-XM_044578437.1;Dbxref=GeneID:123160622,Genbank:XP_044434372.1;Name=XP_044434372.1;gbkey=CDS;gene=LOC123160622;product=

Putative protein FAR1-RELATED SEQUENCE 10

Kozak: TTCATGT ID=cds-XP_044434702.1;Parent=rna-XM_044578767.1;Dbxref=GeneID:123160922,Genbank:XP_044434702.1;Name=XP_044434702.1;gbkey=CDS;gene=LOC123160922;product=expansin-A32-like;protein_id=XP_044434702.1

GO:0005576; Extracellular region.

Kozak: CCCATGT ID=cds-XP_044434722.1;Parent=rna-XM_044578787.1;Dbxref=GeneID:123160934,Genbank:XP_044434722.1;Name=XP_044434722.1;gbkey=CDS;gene=LOC123160934;product=

Protein ALP1-like

Kozak: CAGATGC ID=cds-XP_044434754.1;Parent=rna-XM_044578819.1;Dbxref=GeneID:123160968,Genbank:XP_044434754.1;Name=XP_044434754.1;gbkey=CDS;gene=LOC123160968;product=PH%2C RCC1 and FYVE domains-containing protein 1-like isoform X3;protein_id=XP_044434754.1

GO:0046872; Metal ion binding.

Kozak: CCAATGC ID=cds-XP_044434921.1;Parent=rna-XM_044578986.1;Dbxref=GeneID:123161132,Genbank:XP_044434921.1;Name=XP_044434921.1;gbkey=CDS;gene=LOC123161132;product=

Putative pentatricopeptide repeat-containing protein At5g37570.

Kozak: CCAATGC ID=cds-XP_044434945.1;Parent=rna-XM_044579010.1;Dbxref=GeneID:123161167,Genbank:XP_044434945.1;Name=XP_044434945.1;gbkey=CDS;gene=LOC123161167;product=serine/threonine-protein kinase-like protein CCR4;protein_id=XP_044434945.1

GO:0016021; Integral component of membrane.

Kozak: CCCATGT ID=cds-XP_044435016.1;Parent=rna-XM_044579081.1;Dbxref=GeneID:123161234,Genbank:XP_044435016.1;Name=XP_044435016.1;gbkey=CDS;gene=LOC123161234;product=transcription factor TGAL3-like isoform X1;protein_id=XP_044435016.1

GO:0003700; DNA-binding transcription factor activity.

Kozak: CCCATGT ID=cds-XP_044435204.1;Parent=rna-XM_044579269.1;Dbxref=GeneID:123161438,Genbank:XP_044435204.1;Name=XP_044435204.1;gbkey=CDS;gene=LOC123161438;product=DNA repair protein RAD51 homolog B-like;protein_id=XP_044435204.1

GO:0000794; Condensed nuclear chromosome.

Kozak: CAGATGC ID=cds-XP_044435300.1;Parent=rna-XM_044579365.1;Dbxref=GeneID:123161559,Genbank:XP_044435300.1;Name=XP_044435300.1;gbkey=CDS;gene=LOC123161559;product=polyubiquitin;protein_id=XP_044435300.1

GO:0005737; Cytoplasm.

Kozak: CAGATGC ID=cds-XP_044435903.1;Parent=rna-XM_044579968.1;Dbxref=GeneID:123162179,Genbank:XP_044435903.1;Name=XP_044435903.1;gbkey=CDS;gene=LOC123162179;product=polyubiquitin;protein_id=XP_044435903.1

GO:0005737; Cytoplasm.

Kozak: CAGATGC ID=cds-XP_044435904.1;Parent=rna-XM_044579969.1;Dbxref=GeneID:123162180,Genbank:XP_044435904.1;Name=XP_044435904.1;gbkey=CDS;gene=LOC123162180;product=polyubiquitin;protein_id=XP_044435904.1

GO:0005737; Cytoplasm.

Kozak: CAGATGC ID=cds-XP_044436396.1;Parent=rna-XM_044580461.1;Dbxref=GeneID:123162684,Genbank:XP_044436396.1;Name=XP_044436396.1;gbkey=CDS;gene=LOC123162684;product=polyubiquitin;protein_id=XP_044436396.1

GO:0005737; Cytoplasm.

Kozak: CAGATGC ID=cds-XP_044436397.1;Parent=rna-XM_044580462.1;Dbxref=GeneID:123162685,Genbank:XP_044436397.1;Name=XP_044436397.1;gbkey=CDS;gene=LOC123162685;product=polyubiquitin;protein_id=XP_044436397.1

GO:0005737; Cytoplasm.

Kozak: TCCATGT ID=cds-XP_044436402.1;Parent=rna-XM_044580467.1;Dbxref=GeneID:123162690,Genbank:XP_044436402.1;Name=XP_044436402.1;gbkey=CDS;gene=LOC123162690;product=kinesin-like protein KIN-14M isoform X1;protein_id=XP_044436402.1

GO:0005874; Microtubule.

Kozak: CCAATGC ID=cds-XP_044436431.1;Parent=rna-XM_044580496.1;Dbxref=GeneID:123162728,Genbank:XP_044436431.1;Name=XP_044436431.1;gbkey=CDS;gene=LOC123162728;product=cleavage stimulating factor 64-like;protein_id=XP_044436431.1

GO:0005847; mRNA cleavage and polyadenylation specificity factor complex.

Kozak: CCAATGC ID=cds-XP_044436641.1;Parent=rna-XM_044580706.1;Dbxref=GeneID:123162935,Genbank:XP_044436641.1;Name=XP_044436641.1;gbkey=CDS;gene=LOC123162935;product=uncharacterized protein LOC123162935;protein_id=XP_044436641.1

GO:0043231; Intracellular membrane-bounded organelle.

Kozak: CAGATGC ID=cds-XP_044436758.1;Parent=rna-XM_044580823.1;Dbxref=GeneID:123163063,Genbank:XP_044436758.1;Name=XP_044436758.1;gbkey=CDS;gene=LOC123163063;product=starch synthase 1%2C chloroplastic/amyloplastic-like;protein_id=XP_044436758.1

GO:0009501; Amyloplast.

Kozak: TCCATGT ID=cds-XP_044408701.1;Parent=rna-XM_044552766.1;Dbxref=GeneID:123133252,Genbank:XP_044408701.1;Name=XP_044408701.1;gbkey=CDS;gene=LOC123133252;product=GDSL esterase/lipase At5g45910-like;protein_id=XP_044408701.1

GO:0016788; Hydrolase activity.

Kozak: TTCATGT ID=cds-XP_044408971.1;Parent=rna-XM_044553036.1;Dbxref=GeneID:123133573,Genbank:XP_044408971.1;Name=XP_044408971.1;gbkey=CDS;gene=LOC123133573;product=

Protein H2A.6-like.

Kozak: CCCATGT ID=cds-XP_044409067.1;Parent=rna-XM_044553132.1;Dbxref=GeneID:123133709,Genbank:XP_044409067.1;Name=XP_044409067.1;gbkey=CDS;gene=LOC123133709;product=pentatricopeptide repeat-containing protein At4g01990%2C mitochondrial-like;protein_id=XP_044409067.1

GO:0005739; Mitochondrion.

Kozak: CCAATGT ID=cds-XP_044409160.1;Parent=rna-XM_044553225.1;Dbxref=GeneID:123133821,Genbank:XP_044409160.1;Name=XP_044409160.1;gbkey=CDS;gene=LOC123133821;product=serrate RNA effector molecule-like;protein_id=XP_044409160.1

GO:0016604; Nuclear body.

Kozak: TACATGC ID=cds-XP_044409544.1;Parent=rna-XM_044553609.1;Dbxref=GeneID:123134340,Genbank:XP_044409544.1;Name=XP_044409544.1;gbkey=CDS;gene=LOC123134340;product=probable WRKY transcription factor 12;protein_id=XP_044409544.1

GO:0005634; Nucleus.

Kozak: CCCATGT ID=cds-XP_044410080.1;Parent=rna-XM_044554145.1;Dbxref=GeneID:123135011,Genbank:XP_044410080.1;Name=XP_044410080.1;gbkey=CDS;gene=LOC123135011;product=uncharacterized protein LOC123135011;protein_id=XP_044410080.1

GO:0006952; Defense response.

Kozak: CCCATGT ID=cds-XP_044410238.1;Parent=rna-XM_044554303.1;Dbxref=GeneID:123135240,Genbank:XP_044410238.1;Name=XP_044410238.1;gbkey=CDS;gene=LOC123135240;product=putative ripening-related protein 1;protein_id=XP_044410238.1

GO:0005576; Extracellular region.

Kozak: CCCATGC ID=cds-XP_044410792.1;Parent=rna-XM_044554857.1;Dbxref=GeneID:123135679,Genbank:XP_044410792.1;Name=XP_044410792.1;gbkey=CDS;gene=LOC123135679;product=peptidyl-prolyl cis-trans isomerase FKBP18%2C chloroplastic-like;protein_id=XP_044410792.1

GO:0003755; Peptidylprolyl cis-trans isomerase activity.

Kozak: CCCATGC ID=cds-XP_044410800.1;Parent=rna-XM_044554865.1;Dbxref=GeneID:123135689,Genbank:XP_044410800.1;Name=XP_044410800.1;gbkey=CDS;gene=LOC123135689;product=RING-H2 finger protein ATL52-like;protein_id=XP_044410800.1

GO:0016021; integral component of membrane.

Kozak: CCCATGC ID=cds-XP_044410981.1;Parent=rna-XM_044555046.1;Dbxref=GeneID:123135822,Genbank:XP_044410981.1;Name=XP_044410981.1;gbkey=CDS;gene=LOC123135822;product=transcription termination factor MTERF6%2C chloroplastic/mitochondrial-like;protein_id=XP_044410981.1

GO:0009507; Chloroplast.

Kozak: CCCATGC ID=cds-XP_044411211.1;Parent=rna-XM_044555276.1;Dbxref=GeneID:123136001,Genbank:XP_044411211.1;Name=XP_044411211.1;gbkey=CDS;gene=LOC123136001;product=ribose-phosphate pyrophosphokinase 1%2C chloroplastic-like;protein_id=XP_044411211.1

GO:0005737; Cytoplasm.

Kozak: CCCATGC ID=cds-XP_044411284.1;Parent=rna-XM_044555349.1;Dbxref=GeneID:123136063,Genbank:XP_044411284.1;Name=XP_044411284.1;gbkey=CDS;gene=LOC123136063;product=pentatricopeptide repeat-containing protein At4g01990%2C mitochondrial-like;protein_id=XP_044411284.1

GO:0005739; Mitochondrion.

Kozak: CCCATGT ID=cds-XP_044411329.1;Parent=rna-XM_044555394.1;Dbxref=GeneID:123136106,Genbank:XP_044411329.1;Name=XP_044411329.1;gbkey=CDS;gene=LOC123136106;product=

Phosphatidylinositol/phosphatidylcholine transfer protein SFH6-like isoform X1.

Kozak: CAGATGC ID=cds-XP_044411590.1;Parent=rna-XM_044555655.1;Dbxref=GeneID:123136303,Genbank:XP_044411590.1;Name=XP_044411590.1;gbkey=CDS;gene=LOC123136303;product=polyubiquitin 11;protein_id=XP_044411590.1

GO:0005737; Cytoplasm.

Kozak: CCCATGC ID=cds-XP_044411916.1;Parent=rna-XM_044555981.1;Dbxref=GeneID:123136573,Genbank:XP_044411916.1;Name=XP_044411916.1;gbkey=CDS;gene=LOC123136573;product=DNA-directed RNA polymerase III subunit 2-like;protein_id=XP_044411916.1

GO:0005666; RNA polymerase III complex.

Kozak: CAGATGC ID=cds-XP_044412125.1;Parent=rna-XM_044556190.1;Dbxref=GeneID:123136717,Genbank:XP_044412125.1;Name=XP_044412125.1;gbkey=CDS;gene=LOC123136717;product=

Ultraviolet-B receptor UVR8-like.

Kozak: TCCATGT ID=cds-XP_044412945.1;Parent=rna-XM_044557010.1;Dbxref=GeneID:123137306,Genbank:XP_044412945.1;Name=XP_044412945.1;gbkey=CDS;gene=LOC123137306;product=inorganic phosphate transporter 2-1%2C chloroplastic-like;protein_id=XP_044412945.1

GO:0005887; integral component of plasma membrane.

Kozak: TCCATGT ID=cds-XP_044413121.1;Parent=rna-XM_044557186.1;Dbxref=GeneID:123137433,Genbank:XP_044413121.1;Name=XP_044413121.1;gbkey=CDS;gene=LOC123137433;product=acetolactate synthase small subunit 2%2C chloroplastic-like isoform X1;protein_id=XP_044413121.1

GO:0005737; Cytoplasm.

Kozak: CCAATGC ID=cds-XP_044413391.1;Parent=rna-XM_044557456.1;Dbxref=GeneID:123137635,Genbank:XP_044413391.1;Name=XP_044413391.1;gbkey=CDS;gene=LOC123137635;product=U-box domain-containing protein 26-like;protein_id=XP_044413391.1

GO:0061630; Ubiquitin protein ligase activity.

Kozak: TCCATGT ID=cds-XP_044413525.1;Parent=rna-XM_044557590.1;Dbxref=GeneID:123137741,Genbank:XP_044413525.1;Name=XP_044413525.1;gbkey=CDS;gene=LOC123137741;product=transcription initiation factor TFIID subunit 4b-like;protein_id=XP_044413525.1

GO:0005669; Transcription factor TFIID complex

Kozak: CCCATGC ID=cds-XP_044413824.1;Parent=rna-XM_044557889.1;Dbxref=GeneID:123137999,Genbank:XP_044413824.1;Name=XP_044413824.1;gbkey=CDS;gene=LOC123137999;product=ribose-phosphate pyrophosphokinase 4-like;protein_id=XP_044413824.1

GO:0005737; Cytoplasm.

Kozak: CAGATGC ID=cds-XP_044414074.1;Parent=rna-XM_044558139.1;Dbxref=GeneID:123138207,Genbank:XP_044414074.1;Name=XP_044414074.1;gbkey=CDS;gene=LOC123138207;product=pentatricopeptide repeat-containing protein PPR5 homolog%2C chloroplastic-like;protein_id=XP_044414074.1

GO:0003729; mRNA binding.

Kozak: TTCATGT ID=cds-XP_044414155.1;Parent=rna-XM_044558220.1;Dbxref=GeneID:123138278,Genbank:XP_044414155.1;Name=XP_044414155.1;gbkey=CDS;gene=LOC123138278;product=cation/H(+) antiporter 15-like;protein_id=XP_044414155.1

GO:0009941; Chloroplast envelope.

Kozak: CCCATGT ID=cds-XP_044414175.1;Parent=rna-XM_044558240.1;Dbxref=GeneID:123138291,Genbank:XP_044414175.1;Name=XP_044414175.1;gbkey=CDS;gene=LOC123138291;product=dynamin-2B-like;protein_id=XP_044414175.1

GO:0005874; Microtubule.

Kozak: CCCATGC ID=cds-XP_044414280.1;Parent=rna-XM_044558345.1;Dbxref=GeneID:123138379,Genbank:XP_044414280.1;Name=XP_044414280.1;gbkey=CDS;gene=LOC123138379;product=putative D-cysteine desulfhydrase 1%2C mitochondrial;protein_id=XP_044414280.1

GO:0019148; D-cysteine desulfhydrase activity.

Kozak: CCAATGC ID=cds-XP_044414394.1;Parent=rna-XM_044558459.1;Dbxref=GeneID:123138480,Genbank:XP_044414394.1;Name=XP_044414394.1;gbkey=CDS;gene=LOC123138480;product=LRR receptor-like serine/threonine-protein kinase ER2 isoform X1;protein_id=XP_044414394.1

GO:0016021; integral component of membrane.

Kozak: TACATGC ID=cds-XP_044414631.1;Parent=rna-XM_044558696.1;Dbxref=GeneID:123138835,Genbank:XP_044414631.1;Name=XP_044414631.1;gbkey=CDS;gene=LOC123138835;product=

60S ribosomal protein L36-2-like.

Kozak: CCCATGC ID=cds-XP_044414855.1;Parent=rna-XM_044558920.1;Dbxref=GeneID:123139121,Genbank:XP_044414855.1;Name=XP_044414855.1;gbkey=CDS;gene=LOC123139121;product=transcription factor LRL3-like;protein_id=XP_044414855.1

GO:0005634; Nucleus

Kozak: CAGATGC ID=cds-XP_044414923.1;Parent=rna-XM_044558988.1;Dbxref=GeneID:123139176,Genbank:XP_044414923.1;Name=XP_044414923.1;gbkey=CDS;gene=LOC123139176;product=elongation factor 1-gamma 2-like;protein_id=XP_044414923.1

GO:0004364; Glutathione transferase activity.

Kozak: CCCATGC ID=cds-XP_044415500.1;Parent=rna-XM_044559565.1;Dbxref=GeneID:123139872,Genbank:XP_044415500.1;Name=XP_044415500.1;gbkey=CDS;gene=LOC123139872;product=protein LUTEIN DEFICIENT 5%2C chloroplastic-like;protein_id=XP_044415500.1

GO:0016021; Integral component of membrane.

Kozak: CCCATGC ID=cds-XP_044413933.1;Parent=rna-XM_044557998.1;Dbxref=GeneID:100037553,Genbank:XP_044413933.1;Name=XP_044413933.1;gbkey=CDS;gene=LOC100037553;product=dof zinc finger protein 4;protein_id=XP_044413933.1

GO:0005634; Nucleus.

Kozak: TTCATGT ID=cds-XP_044409085.1;Parent=rna-XM_044553150.1;Dbxref=GeneID:123133734,Genbank:XP_044409085.1;Name=XP_044409085.1;gbkey=CDS;gene=LOC123133734;product=ethylene-responsive transcription factor 1-like;protein_id=XP_044409085.1

GO:0005634; Nucleus.

Kozak: CCAATGC ID=cds-XP_044409192.1;Parent=rna-XM_044553257.1;Dbxref=GeneID:123133864,Genbank:XP_044409192.1;Name=XP_044409192.1;gbkey=CDS;gene=LOC123133864;product=tyrosine-sulfated glycopeptide receptor 1-like;protein_id=XP_044409192.1

GO:0016021; Integral component of membrane.

Kozak: TACATGC ID=cds-XP_044409252.1;Parent=rna-XM_044553317.1;Dbxref=GeneID:123133939,Genbank:XP_044409252.1;Name=XP_044409252.1;gbkey=CDS;gene=LOC123133939;product=B-box zinc finger protein 22-like;protein_id=XP_044409252.1

GO:0005634; Nucleus.

Kozak: CCAATGC ID=cds-XP_044409762.1;Parent=rna-XM_044553827.1;Dbxref=GeneID:123134607,Genbank:XP_044409762.1;Name=XP_044409762.1;gbkey=CDS;gene=LOC123134607;product=thioredoxin-like 3-1%2C chloroplastic;protein_id=XP_044409762.1

GO:0009570; Chloroplast stroma.

Kozak: TCCATGT ID=cds-XP_044409823.1;Parent=rna-XM_044553888.1;Dbxref=GeneID:123134688,Genbank:XP_044409823.1;Name=XP_044409823.1;gbkey=CDS;gene=LOC123134688;product=

Probable CCR4-associated factor 1 homolog 11.

Kozak: CCCATGC ID=cds-XP_044409920.1;Parent=rna-XM_044553985.1;Dbxref=GeneID:123134815,Genbank:XP_044409920.1;Name=XP_044409920.1;gbkey=CDS;gene=LOC123134815;product=ethylene-responsive transcription factor ERF003-like;protein_id=XP_044409920.1

GO:0005634; Nucleus.

Kozak: CCCATGC ID=cds-XP_044410394.1;Parent=rna-XM_044554459.1;Dbxref=GeneID:123135395,Genbank:XP_044410394.1;Name=XP_044410394.1;gbkey=CDS;gene=LOC123135395;product=WAT1-related protein At3g30340-like;protein_id=XP_044410394.1

GO:0016021; integral component of membrane.

Kozak: TCCATGT ID=cds-XP_044410470.1;Parent=rna-XM_044554535.1;Dbxref=GeneID:123135452,Genbank:XP_044410470.1;Name=XP_044410470.1;gbkey=CDS;gene=LOC123135452;product=sucrose:sucrose 1-fructosyltransferase-like;protein_id=XP_044410470.1

GO:0016021; integral component of membrane.

Kozak: CCCATGC ID=cds-XP_044410482.1;Parent=rna-XM_044554547.1;Dbxref=GeneID:123135465,Genbank:XP_044410482.1;Name=XP_044410482.1;gbkey=CDS;gene=LOC123135465;product=salt tolerance receptor-like cytoplasmic kinase 1;protein_id=XP_044410482.1

GO:0005886; Plasma membrane.

Kozak: TACATGC ID=cds-XP_044411187.1;Parent=rna-XM_044555252.1;Dbxref=GeneID:123135979,Genbank:XP_044411187.1;Name=XP_044411187.1;gbkey=CDS;gene=LOC123135979;product=

Putative pentatricopeptide repeat-containing protein At1g19290.

Kozak: CCCATGT ID=cds-XP_044411335.1;Parent=rna-XM_044555400.1;Dbxref=GeneID:123136107,Genbank:XP_044411335.1;Name=XP_044411335.1;gbkey=CDS;gene=LOC123136107;product=

phosphatidylinositol/phosphatidylcholine transfer protein SFH12-like isoform X1.

Kozak: CAGATGC ID=cds-XP_044411502.1;Parent=rna-XM_044555567.1;Dbxref=GeneID:123136238,Genbank:XP_044411502.1;Name=XP_044411502.1;gbkey=CDS;gene=LOC123136238;product=polyubiquitin;protein_id=XP_044411502.1

GO:0005737; Cytoplasm.

Kozak: CCAATGC ID=cds-XP_044411677.1;Parent=rna-XM_044555742.1;Dbxref=GeneID:123136380,Genbank:XP_044411677.1;Name=XP_044411677.1;gbkey=CDS;gene=LOC123136380;product=

Receptor-like protein 2 isoform X1.

Kozak: CAGATGC ID=cds-XP_044411732.1;Parent=rna-XM_044555797.1;Dbxref=GeneID:123136436,Genbank:XP_044411732.1;Name=XP_044411732.1;gbkey=CDS;gene=LOC123136436;product=polyubiquitin-like;protein_id=XP_044411732.1

GO:0005737; Cytoplasm.

Kozak: CAGATGC ID=cds-XP_044411733.1;Parent=rna-XM_044555798.1;Dbxref=GeneID:123136437,Genbank:XP_044411733.1;Name=XP_044411733.1;gbkey=CDS;gene=LOC123136437;product=polyubiquitin 11;protein_id=XP_044411733.1

GO:0005737; Cytoplasm.

Kozak: CAGATGC ID=cds-XP_044411734.1;Parent=rna-XM_044555799.1;Dbxref=GeneID:123136438,Genbank:XP_044411734.1;Name=XP_044411734.1;gbkey=CDS;gene=LOC123136438;product=polyubiquitin;protein_id=XP_044411734.1

GO:0005737; Cytoplasm

Kozak: CAGATGC ID=cds-XP_044411818.1;Parent=rna-XM_044555883.1;Dbxref=GeneID:123136499,Genbank:XP_044411818.1;Name=XP_044411818.1;gbkey=CDS;gene=LOC123136499;product=

B3 domain-containing protein LOC_Os12g40090-like isoform X1

Kozak: TTCATGT ID=cds-XP_044412263.1;Parent=rna-XM_044556328.1;Dbxref=GeneID:123136824,Genbank:XP_044412263.1;Name=XP_044412263.1;gbkey=CDS;gene=LOC123136824;product=

Putative F-box protein At2g02030 isoform X1.

Kozak: TTCATGT ID=cds-XP_044412266.1;Parent=rna-XM_044556331.1;Dbxref=GeneID:123136826,Genbank:XP_044412266.1;Name=XP_044412266.1;gbkey=CDS;gene=LOC123136826;product=

putative F-box protein At2g02030 isoform X1.

Kozak: TTCATGT ID=cds-XP_044412270.1;Parent=rna-XM_044556335.1;Dbxref=GeneID:123136827,Genbank:XP_044412270.1;Name=XP_044412270.1;gbkey=CDS;gene=LOC123136827;product=

Putative F-box protein At2g02030 isoform X1.

Kozak: CCCATGT ID=cds-XP_044412467.1;Parent=rna-XM_044556532.1;Dbxref=GeneID:123137001,Genbank:XP_044412467.1;Name=XP_044412467.1;gbkey=CDS;gene=LOC123137001;product=probable serine/threonine-protein kinase SIS8;protein_id=XP_044412467.1

GO:0005737; Cytoplasm.

Kozak: TCCATGT ID=cds-XP_044412474.1;Parent=rna-XM_044556539.1;Dbxref=GeneID:123137007,Genbank:XP_044412474.1;Name=XP_044412474.1;gbkey=CDS;gene=LOC123137007;product=kinesin-like protein KIN-14D isoform X1;protein_id=XP_044412474.1

GO:0005874; Microtubule

Kozak: CCCATGC ID=cds-XP_044412685.1;Parent=rna-XM_044556750.1;Dbxref=GeneID:123137141,Genbank:XP_044412685.1;Name=XP_044412685.1;gbkey=CDS;gene=LOC123137141;product=

Extensin-like

Kozak. CCAATGC ID=cds-XP_044413361.1;Parent=rna-XM_044557426.1;Dbxref=GeneID:123137609,Genbank:XP_044413361.1;Name=XP_044413361.1;gbkey=CDS;gene=LOC123137609;product=protein SHORTAGE IN CHIASMATA 1 homolog;protein_id=XP_044413361.1

GO:0000712; Resolution of meiotic recombination intermediates.

Kozak: CCCATGC ID=cds-XP_044413568.1;Parent=rna-XM_044557633.1;Dbxref=GeneID:123137787,Genbank:XP_044413568.1;Name=XP_044413568.1;gbkey=CDS;gene=LOC123137787;product=plasmodesmata-located protein 8-like;protein_id=XP_044413568.1

GO:0016021; Integral component of membrane.

Kozak: CAGATGC ID=cds-XP_044413642.1;Parent=rna-XM_044557707.1;Dbxref=GeneID:123137854,Genbank:XP_044413642.1;Name=XP_044413642.1;gbkey=CDS;gene=LOC123137854;product=polyubiquitin-like;protein_id=XP_044413642.1

GO:0005737; Cytoplasm.

Kozak: CCCATGT ID=cds-XP_044413671.1;Parent=rna-XM_044557736.1;Dbxref=GeneID:123137882,Genbank:XP_044413671.1;Name=XP_044413671.1;gbkey=CDS;gene=LOC123137882;product=zinc finger CCCH domain-containing protein 15-like;protein_id=XP_044413671.1

GO:0003677; DNA binding

Kozak: CCCATGC ID=cds-XP_044413741.1;Parent=rna-XM_044557806.1;Dbxref=GeneID:123137916,Genbank:XP_044413741.1;Name=XP_044413741.1;gbkey=CDS;gene=LOC123137916;product=uncharacterized protein LOC123137916;protein_id=XP_044413741.1

GO:0016021; Integral component of membrane

Kozak CCCATGT ID=cds-XP_044413750.1;Parent=rna-XM_044557815.1;Dbxref=GeneID:123137923,Genbank:XP_044413750.1;Name=XP_044413750.1;gbkey=CDS;gene=LOC123137923;product=

Pheophytinase%2C chloroplastic-like.

Kozak: TGTATGT ID=cds-XP_044413894.1;Parent=rna-XM_044557959.1;Dbxref=GeneID:123138064,Genbank:XP_044413894.1;Name=XP_044413894.1;gbkey=CDS;gene=LOC123138064;product=

Tyrosine--tRNA ligase 1%2C cytoplasmic-like.

Kozak: CCCATGC ID=cds-XP_044414240.1;Parent=rna-XM_044558305.1;Dbxref=GeneID:123138337,Genbank:XP_044414240.1;Name=XP_044414240.1;gbkey=CDS;gene=LOC123138337;product=

Protein MOR1-like.

Kozak: TCCATGT ID=cds-XP_044414756.1;Parent=rna-XM_044558821.1;Dbxref=GeneID:123138987,Genbank:XP_044414756.1;Name=XP_044414756.1;gbkey=CDS;gene=LOC123138987;product=eukaryotic translation initiation factor 5-like;protein_id=XP_044414756.1

GO:0003743; Translation initiation factor activity.

Kozak: CCAATGC ID=cds-XP_044414815.1;Parent=rna-XM_044558880.1;Dbxref=GeneID:123139065,Genbank:XP_044414815.1;Name=XP_044414815.1;gbkey=CDS;gene=LOC123139065;product=uncharacterized protein LOC123139065;protein_id=XP_044414815.1

GO:0044260; cellular macromolecule metabolic process

Kozak: CCAATGT ID=cds-XP_044414829.1;Parent=rna-XM_044558894.1;Dbxref=GeneID:123139085,Genbank:XP_044414829.1;Name=XP_044414829.1;gbkey=CDS;gene=LOC123139085;product=

Protein TRACHEARY ELEMENT DIFFERENTIATION-RELATED 7A-like.

Kozak: CCCATGC ID=cds-XP_044415076.1;Parent=rna-XM_044559141.1;Dbxref=GeneID:123139339,Genbank:XP_044415076.1;Name=XP_044415076.1;gbkey=CDS;gene=LOC123139339;product=GATA transcription factor 5-like;protein_id=XP_044415076.1

GO:0043565; Sequence-specific DNA binding.

Kozak: CCCATGC ID=cds-XP_044415111.1;Parent=rna-XM_044559176.1;Dbxref=GeneID:123139366,Genbank:XP_044415111.1;Name=XP_044415111.1;gbkey=CDS;gene=LOC123139366;product=

Poly [ADP-ribose] polymerase 2-like

Kozak: CCAATGC ID=cds-XP_044415351.1;Parent=rna-XM_044559416.1;Dbxref=GeneID:123139682,Genbank:XP_044415351.1;Name=XP_044415351.1;gbkey=CDS;gene=LOC123139682;product=protein DMP3-like;protein_id=XP_044415351.1

GO:0016021; Integral component of membrane.

Kozak: TCCATGT ID=cds-XP_044360060.1;Parent=rna-XM_044504125.1;Dbxref=GeneID:123081565,Genbank:XP_044360060.1;Name=XP_044360060.1;gbkey=CDS;gene=LOC123081565;product=

Putative MO25-like protein At5g47540 isoform X1.

Kozak: CCCATGC ID=cds-XP_044360232.1;Parent=rna-XM_044504297.1;Dbxref=GeneID:123081763,Genbank:XP_044360232.1;Name=XP_044360232.1;gbkey=CDS;gene=LOC123081763;product=

Proline-rich protein 4-like.

Kozak: TACATGC ID=cds-XP_044360319.1;Parent=rna-XM_044504384.1;Dbxref=GeneID:123081869,Genbank:XP_044360319.1;Name=XP_044360319.1;gbkey=CDS;gene=LOC123081869;product=sex determination protein tasselseed-2-like;protein_id=XP_044360319.1

GO:0005938; Cell cortex.

Kozak: CGAATGT ID=cds-XP_044360360.1;Parent=rna-XM_044504425.1;Dbxref=GeneID:123081923,Genbank:XP_044360360.1;Name=XP_044360360.1;gbkey=CDS;gene=LOC123081923;product=

Macro domain-containing protein VPA0103-like isoform X1.

Kozak: CCCATGC ID=cds-XP_044360457.1;Parent=rna-XM_044504522.1;Dbxref=GeneID:123082107,Genbank:XP_044360457.1;Name=XP_044360457.1;gbkey=CDS;gene=LOC123082107;product=translation initiation factor IF-2-like;protein_id=XP_044360457.1

GO:0005634; Nucleus.

Kozak: CCCATGT ID=cds-XP_044360593.1;Parent=rna-XM_044504658.1;Dbxref=GeneID:123082296,Genbank:XP_044360593.1;Name=XP_044360593.1;gbkey=CDS;gene=LOC123082296;product=

Extensin-like.

Kozak: TCCATGT ID=cds-XP_044360697.1;Parent=rna-XM_044504762.1;Dbxref=GeneID:123082434,Genbank:XP_044360697.1;Name=XP_044360697.1;gbkey=CDS;gene=LOC123082434;product=protein argonaute 12-like;protein_id=XP_044360697.1

GO:0005737; Cytoplasm.

Kozak: CCCATGC ID=cds-XP_044360888.1;Parent=rna-XM_044504953.1;Dbxref=GeneID:123082665,Genbank:XP_044360888.1;Name=XP_044360888.1;gbkey=CDS;gene=LOC123082665;product=

Probable pathogenesis-related protein ARB_02861.

Kozak: CCAATGT ID=cds-XP_044361791.1;Parent=rna-XM_044505856.1;Dbxref=GeneID:123084055,Genbank:XP_044361791.1;Name=XP_044361791.1;gbkey=CDS;gene=LOC123084055;product=

Chaperone protein ClpB1-like.

Kozak: CAGATGC ID=cds-XP_044362239.1;Parent=rna-XM_044506304.1;Dbxref=GeneID:123084792,Genbank:XP_044362239.1;Name=XP_044362239.1;gbkey=CDS;gene=LOC123084792;product=pentatricopeptide repeat-containing protein At5g15300-like;protein_id=XP_044362239.1

GO:0005886; Plasma membrane.

Kozak: TCCATGT ID=cds-XP_044362338.1;Parent=rna-XM_044506403.1;Dbxref=GeneID:123084873,Genbank:XP_044362338.1;Name=XP_044362338.1;gbkey=CDS;gene=LOC123084873;product=

Late embryogenesis abundant protein D-34-like.

Kozak: TTCATGT ID=cds-XP_044362413.1;Parent=rna-XM_044506478.1;Dbxref=GeneID:123084936,Genbank:XP_044362413.1;Name=XP_044362413.1;gbkey=CDS;gene=LOC123084936;product=deSI-like protein At4g17486 isoform X1;protein_id=XP_044362413.1

GO:0101005; Deubiquitinase activity.

Kozak: TCCATGT ID=cds-XP_044363287.1;Parent=rna-XM_044507352.1;Dbxref=GeneID:123085675,Genbank:XP_044363287.1;Name=XP_044363287.1;gbkey=CDS;gene=LOC123085675;product=DEAD-box ATP-dependent RNA helicase 24;protein_id=XP_044363287.1

GO:0005634; Nucleus.

Kozak: CCAATGT ID=cds-XP_044363358.1;Parent=rna-XM_044507423.1;Dbxref=GeneID:123085727,Genbank:XP_044363358.1;Name=XP_044363358.1;gbkey=CDS;gene=LOC123085727;product=tRNA-specific adenosine deaminase TAD2-like isoform X1;protein_id=XP_044363358.1

GO:0052717; tRNA-specific adenosine-34 deaminase activity.

Kozak: CCAATGC ID=cds-XP_044363444.1;Parent=rna-XM_044507509.1;Dbxref=GeneID:123085799,Genbank:XP_044363444.1;Name=XP_044363444.1;gbkey=CDS;gene=LOC123085799;product=transcription factor RF2b-like;protein_id=XP_044363444.1

GO:0005634; Nucleus.

Kozak: CAGATGC ID=cds-XP_044363586.1;Parent=rna-XM_044507651.1;Dbxref=GeneID:123085947,Genbank:XP_044363586.1;Name=XP_044363586.1;gbkey=CDS;gene=LOC123085947;product=armadillo repeat-containing protein 8-like;protein_id=XP_044363586.1

GO:0005737; Cytoplasm.

Kozak: CCAATGC ID=cds-XP_044363705.1;Parent=rna-XM_044507770.1;Dbxref=GeneID:123086070,Genbank:XP_044363705.1;Name=XP_044363705.1;gbkey=CDS;gene=LOC123086070;product=O-fucosyltransferase 1-like isoform X1;protein_id=XP_044363705.1

GO:0005737; Cytoplasm.

Kozak: TGTATGT ID=cds-XP_044363952.1;Parent=rna-XM_044508017.1;Dbxref=GeneID:123086285,Genbank:XP_044363952.1;Name=XP_044363952.1;gbkey=CDS;gene=LOC123086285;product=alanine--tRNA ligase-like;protein_id=XP_044363952.1

GO:0009507; Chloroplast.

Kozak: CCCATGT ID=cds-XP_044364239.1;Parent=rna-XM_044508304.1;Dbxref=GeneID:123086545,Genbank:XP_044364239.1;Name=XP_044364239.1;gbkey=CDS;gene=LOC123086545;product=protein transport protein Sec24-like CEF;protein_id=XP_044364239.1

GO:0030127; COPII vesicle coat.

Kozak: CCCATGT ID=cds-XP_044364258.1;Parent=rna-XM_044508323.1;Dbxref=GeneID:123086565,Genbank:XP_044364258.1;Name=XP_044364258.1;gbkey=CDS;gene=LOC123086565;product=probable LRR receptor-like serine/threonine-protein kinase RKF3;protein_id=XP_044364258.1

GO:0016021; Integral component of membrane.

Kozak: CCAATGC ID=cds-XP_044364259.1;Parent=rna-XM_044508324.1;Dbxref=GeneID:123086566,Genbank:XP_044364259.1;Name=XP_044364259.1;gbkey=CDS;gene=LOC123086566;product=protein SHORTAGE IN CHIASMATA 1 homolog;protein_id=XP_044364259.1

GO:0061630; Ubiquitin protein ligase activity.

Kozak: CCAATGC ID=cds-XP_044364265.1;Parent=rna-XM_044508330.1;Dbxref=GeneID:123086570,Genbank:XP_044364265.1;Name=XP_044364265.1;gbkey=CDS;gene=LOC123086570;product=calcium-dependent protein kinase 24-like;protein_id=XP_044364265.1

GO:0005737; Cytoplasm.

Kozak: CAGATGC ID=cds-XP_044364549.1;Parent=rna-XM_044508614.1;Dbxref=GeneID:123086807,Genbank:XP_044364549.1;Name=XP_044364549.1;gbkey=CDS;gene=LOC123086807;product=

Tetrapyrrole-binding protein%2C chloroplastic-like.

Kozak: CCCATGC ID=cds-XP_044364556.1;Parent=rna-XM_044508621.1;Dbxref=GeneID:123086811,Genbank:XP_044364556.1;Name=XP_044364556.1;gbkey=CDS;gene=LOC123086811;product=endonuclease III homolog 1%2C chloroplastic-like isoform X1;protein_id=XP_044364556.1

GO:0042644; Chloroplast nucleoid.

Kozak: CAGATGC ID=cds-XP_044364653.1;Parent=rna-XM_044508718.1;Dbxref=GeneID:123086893,Genbank:XP_044364653.1;Name=XP_044364653.1;gbkey=CDS;gene=LOC123086893;product=B3 domain-containing protein Os03g0622100-like;protein_id=XP_044364653.1

GO:0005634; Nucleus.

Kozak: CCCATGC ID=cds-XP_044365422.1;Parent=rna-XM_044509487.1;Dbxref=GeneID:123087469,Genbank:XP_044365422.1;Name=XP_044365422.1;gbkey=CDS;gene=LOC123087469;product=uncharacterized protein LOC123087469;protein_id=XP_044365422.1

GO:0045927; Positive regulation of growth.

Kozak: CCAATGC ID=cds-XP_044365946.1;Parent=rna-XM_044510011.1;Dbxref=GeneID:123087890,Genbank:XP_044365946.1;Name=XP_044365946.1;gbkey=CDS;gene=LOC123087890;product=protein DJ-1 homolog C-like;protein_id=XP_044365946.1

GO:0005829; Cytosol.

Kozak: CCCATGC ID=cds-XP_044366230.1;Parent=rna-XM_044510295.1;Dbxref=GeneID:123088130,Genbank:XP_044366230.1;Name=XP_044366230.1;gbkey=CDS;gene=LOC123088130;product=beta-glucuronosyltransferase GlcAT14A-like;protein_id=XP_044366230.1

GO:0016020; Membrane.123088504:cds-XP_044366643.1

Kozak: TACATGC ID=cds-XP_044366643.1;Parent=rna-XM_044510708.1;Dbxref=GeneID:123088504,Genbank:XP_044366643.1;Name=XP_044366643.1;gbkey=CDS;gene=LOC123088504;product=protein CWC15 homolog;protein_id=XP_044366643.1

GO:0071013; Catalytic step 2 spliceosome.

Kozak: CCCATGT ID=cds-XP_044362178.1;Parent=rna-XM_044506243.1;Dbxref=GeneID:543243,Genbank:XP_044362178.1;Name=XP_044362178.1;gbkey=CDS;gene=LOC543243;product=adenylosuccinate synthetase%2C chloroplastic;protein_id=XP_044362178.1

GO:0009507; Chloroplast.

Kozak: TCCATGT ID=cds-XP_044360831.1;Parent=rna-XM_044504896.1;Dbxref=GeneID:100192132,Genbank:XP_044360831.1;Name=XP_044360831.1;gbkey=CDS;gene=LOC100192132;product=ethylene-response factor C3;protein_id=XP_044360831.1

GO:0005634; Nucleus.

Kozak: CCAATGT ID=cds-XP_044360293.1;Parent=rna-XM_044504358.1;Dbxref=GeneID:123081844,Genbank:XP_044360293.1;Name=XP_044360293.1;gbkey=CDS;gene=LOC123081844;product=probable membrane-associated kinase regulator 4;protein_id=XP_044360293.1

GO:0005886; Plasma membrane.

Kozak: CCAATGT ID=cds-XP_044360420.1;Parent=rna-XM_044504485.1;Dbxref=GeneID:123082060,Genbank:XP_044360420.1;Name=XP_044360420.1;gbkey=CDS;gene=LOC123082060;product=protein DMP6-like;protein_id=XP_044360420.1

GO:0016021; Integral component of membrane.

Kozak: CCAATGC ID=cds-XP_044360701.1;Parent=rna-XM_044504766.1;Dbxref=GeneID:123082440,Genbank:XP_044360701.1;Name=XP_044360701.1;gbkey=CDS;gene=LOC123082440;product=probable galacturonosyltransferase-like 1;protein_id=XP_044360701.1

GO:0005794; Golgi apparatus.

Kozak: TCCATGT ID=cds-XP_044360807.1;Parent=rna-XM_044504872.1;Dbxref=GeneID:123082563,Genbank:XP_044360807.1;Name=XP_044360807.1;gbkey=CDS;gene=LOC123082563;product=

Receptor-like protein EIX1.

Kozak: TGTATGT ID=cds-XP_044361297.1;Parent=rna-XM_044505362.1;Dbxref=GeneID:123083285,Genbank:XP_044361297.1;Name=XP_044361297.1;gbkey=CDS;gene=LOC123083285;product=

Wiskott-Aldrich syndrome protein family member 2-like.

Kozak: CAGATGC ID=cds-XP_044361385.1;Parent=rna-XM_044505450.1;Dbxref=GeneID:123083399,Genbank:XP_044361385.1;Name=XP_044361385.1;gbkey=CDS;gene=LOC123083399;product=uncharacterized protein LOC123083399;protein_id=XP_044361385.1

GO:0016021; Integral component of membrane.

Kozak: CCAATGC ID=cds-XP_044361627.1;Parent=rna-XM_044505692.1;Dbxref=GeneID:123083749,Genbank:XP_044361627.1;Name=XP_044361627.1;gbkey=CDS;gene=LOC123083749;product=

Zinc finger BED domain-containing protein RICESLEEPER 2-like.

Kozak: CCCATGT ID=cds-XP_044361797.1;Parent=rna-XM_044505862.1;Dbxref=GeneID:123084069,Genbank:XP_044361797.1;Name=XP_044361797.1;gbkey=CDS;gene=LOC123084069;partial=true;product=

Protein FAR1-RELATED SEQUENCE 5-like.

Kozak: CAGATGC ID=cds-XP_044361875.1;Parent=rna-XM_044505940.1;Dbxref=GeneID:123084220,Genbank:XP_044361875.1;Name=XP_044361875.1;gbkey=CDS;gene=LOC123084220;product=

Uncharacterized methyltransferase At2g41040%2C chloroplastic-like.

Kozak: CCCATGC ID=cds-XP_044362176.1;Parent=rna-XM_044506241.1;Dbxref=GeneID:123084748,Genbank:XP_044362176.1;Name=XP_044362176.1;gbkey=CDS;gene=LOC123084748;product=tRNA-specific 2-thiouridylase MnmA-like;protein_id=XP_044362176.1

GO:0005524; ATP binding.

Kozak: TTCATGT ID=cds-XP_044362341.1;Parent=rna-XM_044506406.1;Dbxref=GeneID:123084877,Genbank:XP_044362341.1;Name=XP_044362341.1;gbkey=CDS;gene=LOC123084877;product=probable serine/threonine-protein kinase PBL16;protein_id=XP_044362341.1

GO:0005524; ATP binding.

Kozak: TCCATGT ID=cds-XP_044362470.1;Parent=rna-XM_044506535.1;Dbxref=GeneID:123084985,Genbank:XP_044362470.1;Name=XP_044362470.1;gbkey=CDS;gene=LOC123084985;product=yrdC domain-containing protein%2C mitochondrial-like;protein_id=XP_044362470.1

GO:0005737; Cytoplasm.

Kozak: CCAATGT ID=cds-XP_044362475.1;Parent=rna-XM_044506540.1;Dbxref=GeneID:123084988,Genbank:XP_044362475.1;Name=XP_044362475.1;gbkey=CDS;gene=LOC123084988;product=dual-specificity RNA methyltransferase RlmN-like;protein_id=XP_044362475.1

GO:0005737; Cytoplasm.

Kozak: CCCATGT ID=cds-XP_044362675.1;Parent=rna-XM_044506740.1;Dbxref=GeneID:123085142,Genbank:XP_044362675.1;Name=XP_044362675.1;gbkey=CDS;gene=LOC123085142;product=

Peroxygenase-like.

Kozak: CGAATGT ID=cds-XP_044362786.1;Parent=rna-XM_044506851.1;Dbxref=GeneID:123085227,Genbank:XP_044362786.1;Name=XP_044362786.1;gbkey=CDS;gene=LOC123085227;product=anaphase-promoting complex subunit 6-like isoform X1;protein_id=XP_044362786.1

GO:0005680; Anaphase-promoting complex.

Kozak: CCCATGT ID=cds-XP_044362863.1;Parent=rna-XM_044506928.1;Dbxref=GeneID:123085307,Genbank:XP_044362863.1;Name=XP_044362863.1;gbkey=CDS;gene=LOC123085307;product=zinc finger CCCH domain-containing protein 15-like;protein_id=XP_044362863.1

GO:0003677; DNA binding.

Kozak: CCAATGC ID=cds-XP_044362886.1;Parent=rna-XM_044506951.1;Dbxref=GeneID:123085324,Genbank:XP_044362886.1;Name=XP_044362886.1;gbkey=CDS;gene=LOC123085324;product=imidazole glycerol phosphate synthase hisHF%2C chloroplastic-like;protein_id=XP_044362886.1

GO:0009507; Chloroplast.

Kozak: CCCATGT ID=cds-XP_044363001.1;Parent=rna-XM_044507066.1;Dbxref=GeneID:123085432,Genbank:XP_044363001.1;Name=XP_044363001.1;gbkey=CDS;gene=LOC123085432;product=

31 kDa ribonucleoprotein%2C chloroplastic-like isoform X2.

Kozak: CCCATGC ID=cds-XP_044363100.1;Parent=rna-XM_044507165.1;Dbxref=GeneID:123085521,Genbank:XP_044363100.1;Name=XP_044363100.1;gbkey=CDS;gene=LOC123085521;product=cardiolipin synthase (CMP-forming)%2C mitochondrial-like;protein_id=XP_044363100.1

GO:0016021; Integral component of membrane.

Kozak: CCCATGT ID=cds-XP_044363290.1;Parent=rna-XM_044507355.1;Dbxref=GeneID:123085678,Genbank:XP_044363290.1;Name=XP_044363290.1;gbkey=CDS;gene=LOC123085678;product=FIP1[III]-like protein isoform X1;protein_id=XP_044363290.1

GO:0005634; Nucleus.

Kozak: CCAATGC ID=cds-XP_044363546.1;Parent=rna-XM_044507611.1;Dbxref=GeneID:123085907,Genbank:XP_044363546.1;Name=XP_044363546.1;gbkey=CDS;gene=LOC123085907;product=phosphatidylinositol 4-phosphate 5-kinase 6-like isoform X1;protein_id=XP_044363546.1

GO:0005886; Plasma membrane.

Kozak: TCCATGT ID=cds-XP_044364029.1;Parent=rna-XM_044508094.1;Dbxref=GeneID:123086339,Genbank:XP_044364029.1;Name=XP_044364029.1;gbkey=CDS;gene=LOC123086339;product=

Proline-%2C glutamic acid- and leucine-rich protein 1-like isoform X2.

Kozak: CCAATGC ID=cds-XP_044364049.1;Parent=rna-XM_044508114.1;Dbxref=GeneID:123086357,Genbank:XP_044364049.1;Name=XP_044364049.1;gbkey=CDS;gene=LOC123086357;product=2-carboxy-D-arabinitol-1-phosphatase-like isoform X1;protein_id=XP_044364049.1

GO:0005737; Cytoplasm.

Kozak: CAGATGC ID=cds-XP_044364515.1;Parent=rna-XM_044508580.1;Dbxref=GeneID:123086782,Genbank:XP_044364515.1;Name=XP_044364515.1;gbkey=CDS;gene=LOC123086782;product=pentatricopeptide repeat-containing protein At3g12770-like;protein_id=XP_044364515.1

GO:0000145; Exocyst.

Kozak: TTCATGT ID=cds-XP_044364784.1;Parent=rna-XM_044508849.1;Dbxref=GeneID:123086981,Genbank:XP_044364784.1;Name=XP_044364784.1;gbkey=CDS;gene=LOC123086981;product=uncharacterized protein LOC123086981 isoform X1;protein_id=XP_044364784.1

GO:0016021; Integral component of membrane.

Kozak: CCCATGT ID=cds-XP_044364928.1;Parent=rna-XM_044508993.1;Dbxref=GeneID:123087091,Genbank:XP_044364928.1;Name=XP_044364928.1;gbkey=CDS;gene=LOC123087091;product=protein POLLEN DEFECTIVE IN GUIDANCE 1-like;protein_id=XP_044364928.1

GO:0030176; Integral component of endoplasmic reticulum membrane.

Kozak. TTCATGT ID=cds-XP_044365129.1;Parent=rna-XM_044509194.1;Dbxref=GeneID:123087238,Genbank:XP_044365129.1;Name=XP_044365129.1;gbkey=CDS;gene=LOC123087238;product=plasma membrane ATPase 1-like isoform X1;protein_id=XP_044365129.1

GO:0016021; Integral component of membrane.

Kozak: CCCATGC ID=cds-XP_044365540.1;Parent=rna-XM_044509605.1;Dbxref=GeneID:123087564,Genbank:XP_044365540.1;Name=XP_044365540.1;gbkey=CDS;gene=LOC123087564;product=translation initiation factor IF-2-like;protein_id=XP_044365540.1

GO:0030154; Cell differentiation.

Kozak: CCAATGC ID=cds-XP_044365657.1;Parent=rna-XM_044509722.1;Dbxref=GeneID:123087671,Genbank:XP_044365657.1;Name=XP_044365657.1;gbkey=CDS;gene=LOC123087671;product=30S ribosomal protein S6 alpha%2C chloroplastic-like;protein_id=XP_044365657.1

GO:0005737; Cytoplasm.

Kozak: CCAATGC ID=cds-XP_044365744.1;Parent=rna-XM_044509809.1;Dbxref=GeneID:123087713,Genbank:XP_044365744.1;Name=XP_044365744.1;gbkey=CDS;gene=LOC123087713;product=pentatricopeptide repeat-containing protein At3g13880-like;protein_id=XP_044365744.1

GO:0003723; RNA binding.

Kozak: CCAATGT ID=cds-XP_044365753.1;Parent=rna-XM_044509818.1;Dbxref=GeneID:123087720,Genbank:XP_044365753.1;Name=XP_044365753.1;gbkey=CDS;gene=LOC123087720;product=ATP-dependent zinc metalloprotease FTSH 5%2C mitochondrial-like;protein_id=XP_044365753.1

GO:0009534; Chloroplast thylakoid.

Kozak: CCCATGT ID=cds-XP_044365755.1;Parent=rna-XM_044509820.1;Dbxref=GeneID:123087721,Genbank:XP_044365755.1;Name=XP_044365755.1;gbkey=CDS;gene=LOC123087721;product=

ATP-dependent zinc metalloprotease FTSH 4%2C mitochondrial-like.

Kozak: CCAATGC ID=cds-XP_044365889.1;Parent=rna-XM_044509954.1;Dbxref=GeneID:123087837,Genbank:XP_044365889.1;Name=XP_044365889.1;gbkey=CDS;gene=LOC123087837;product=uncharacterized protein LOC123087837 isoform X1;protein_id=XP_044365889.1

GO:0046872; Metal ion binding.

Kozak: CCCATGC ID=cds-XP_044365908.1;Parent=rna-XM_044509973.1;Dbxref=GeneID:123087855,Genbank:XP_044365908.1;Name=XP_044365908.1;gbkey=CDS;gene=LOC123087855;product=putative transferase At4g12130%2C mitochondrial;protein_id=XP_044365908.1

GO:0005759; Mitochondrial matrix.

Kozak: TCCATGT ID=cds-XP_044366060.1;Parent=rna-XM_044510125.1;Dbxref=GeneID:123087990,Genbank:XP_044366060.1;Name=XP_044366060.1;gbkey=CDS;gene=LOC123087990;product=probable leucine-rich repeat receptor-like protein kinase At1g35710;protein_id=XP_044366060.1

GO:0016021; Integral component of membrane.

Kozak: CCCATGC ID=cds-XP_044366067.1;Parent=rna-XM_044510132.1;Dbxref=GeneID:123088002,Genbank:XP_044366067.1;Name=XP_044366067.1;gbkey=CDS;gene=LOC123088002;product=superoxide dismutase [Fe] 2%2C chloroplastic-like;protein_id=XP_044366067.1

GO:0042644; Chloroplast nucleoid.

Kozak: CCCATGT ID=cds-XP_044366238.1;Parent=rna-XM_044510303.1;Dbxref=GeneID:123088138,Genbank:XP_044366238.1;Name=XP_044366238.1;gbkey=CDS;gene=LOC123088138;product=pentatricopeptide repeat-containing protein At1g76280-like;protein_id=XP_044366238.1

GO:0008663; 2',3'-cyclic-nucleotide 2'-phosphodiesterase activity.

Kozak: TCCATGT ID=cds-XP_044366432.1;Parent=rna-XM_044510497.1;Dbxref=GeneID:123088304,Genbank:XP_044366432.1;Name=XP_044366432.1;gbkey=CDS;gene=LOC123088304;product=probable transcriptional regulator SLK3;protein_id=XP_044366432.1

GO:0005634; Nucleus.

Kozak: TCCATGT ID=cds-XP_044366579.1;Parent=rna-XM_044510644.1;Dbxref=GeneID:123088439,Genbank:XP_044366579.1;Name=XP_044366579.1;gbkey=CDS;gene=LOC123088439;product=

Disease resistance protein RGA5-like.

Kozak: CCCATGC ID=cds-XP_044354373.1;Parent=rna-XM_044498438.1;Dbxref=GeneID:123075942,Genbank:XP_044354373.1;Name=XP_044354373.1;gbkey=CDS;gene=LOC123075942;product=

Wall-associated receptor kinase 1-like.

Kozak: TGTATGT ID=cds-XP_044355404.1;Parent=rna-XM_044499469.1;Dbxref=GeneID:123077238,Genbank:XP_044355404.1;Name=XP_044355404.1;gbkey=CDS;gene=LOC123077238;product=

Protein FAR1-RELATED SEQUENCE 7-like.

Kozak: CCAATGC ID=cds-XP_044356534.1;Parent=rna-XM_044500599.1;Dbxref=GeneID:123078184,Genbank:XP_044356534.1;Name=XP_044356534.1;gbkey=CDS;gene=LOC123078184;product=

Zinc finger MYM-type protein 1-like.

Kozak: CCAATGT ID=cds-XP_044358093.1;Parent=rna-XM_044502158.1;Dbxref=GeneID:123079386,Genbank:XP_044358093.1;Name=XP_044358093.1;gbkey=CDS;gene=LOC123079386;product=uncharacterized protein LOC123079386;protein_id=XP_044358093.1

GO:0016021; Integral component of membrane.

Kozak: TCCATGT ID=cds-XP_044358950.1;Parent=rna-XM_044503015.1;Dbxref=GeneID:123080125,Genbank:XP_044358950.1;Name=XP_044358950.1;gbkey=CDS;gene=LOC123080125;product=

Proline-rich receptor-like protein kinase PERK2.

Kozak: TCCATGT ID=cds-XP_044363357.1;Parent=rna-XM_044507422.1;Dbxref=GeneID:123085725,Genbank:XP_044363357.1;Name=XP_044363357.1;gbkey=CDS;gene=LOC123085725;product=

Keratin%2C type II cytoskeletal 1-like.

Kozak: TTCATGT ID=cds-XP_044369427.1;Parent=rna-XM_044513492.1;Dbxref=GeneID:123091879,Genbank:XP_044369427.1;Name=XP_044369427.1;gbkey=CDS;gene=LOC123091879;product=

Cortical cell-delineating protein-like.

Kozak: CCCATGT ID=cds-XP_044370581.1;Parent=rna-XM_044514646.1;Dbxref=GeneID:123092811,Genbank:XP_044370581.1;Name=XP_044370581.1;gbkey=CDS;gene=LOC123092811;product=methyl-CpG-binding domain-containing protein 4-like;protein_id=XP_044370581.1

GO:0005634; Nucleus.

Kozak: TACATGC ID=cds-XP_044376298.1;Parent=rna-XM_044520363.1;Dbxref=GeneID:123098389,Genbank:XP_044376298.1;Name=XP_044376298.1;gbkey=CDS;gene=LOC123098389;product=probable purine permease 4;protein_id=XP_044376298.1

GO:0016021; Integral component of membrane

Kozak: TCCATGT ID=cds-XP_044377253.1;Parent=rna-XM_044521318.1;Dbxref=GeneID:123099153,Genbank:XP_044377253.1;Name=XP_044377253.1;gbkey=CDS;gene=LOC123099153;product=

WRKY transcription factor 28-like.

Kozak: CAGATGC ID=cds-XP_044378322.1;Parent=rna-XM_044522387.1;Dbxref=GeneID:123100447,Genbank:XP_044378322.1;Name=XP_044378322.1;gbkey=CDS;gene=LOC123100447;product=E3 ubiquitin-protein ligase WAV3-like;protein_id=XP_044378322.1

GO:0044260; Cellular macromolecule metabolic process.

Kozak: CCCATGC ID=cds-XP_044378702.1;Parent=rna-XM_044522767.1;Dbxref=GeneID:123101214,Genbank:XP_044378702.1;Name=XP_044378702.1;gbkey=CDS;gene=LOC123101214;product=

Putative disease resistance protein RGA3.

Kozak: TTCATGT ID=cds-XP_044378733.1;Parent=rna-XM_044522798.1;Dbxref=GeneID:123101272,Genbank:XP_044378733.1;Name=XP_044378733.1;gbkey=CDS;gene=LOC123101272;product=calmodulin-like protein 3;protein_id=XP_044378733.1

GO:0016021; Integral component of membrane.

Kozak: TTCATGT ID=cds-XP_044378739.1;Parent=rna-XM_044522804.1;Dbxref=GeneID:123101282,Genbank:XP_044378739.1;Name=XP_044378739.1;gbkey=CDS;gene=LOC123101282;product=

Calmodulin-like protein 3.

Kozak: TTCATGT ID=cds-XP_044378761.1;Parent=rna-XM_044522826.1;Dbxref=GeneID:123101320,Genbank:XP_044378761.1;Name=XP_044378761.1;gbkey=CDS;gene=LOC123101320;product=

Calmodulin-like protein 3.

Kozak: TTCATGT ID=cds-XP_044378774.1;Parent=rna-XM_044522839.1;Dbxref=GeneID:123101343,Genbank:XP_044378774.1;Name=XP_044378774.1;gbkey=CDS;gene=LOC123101343;product=calmodulin-like protein 3;protein_id=XP_044378774.1

GO:0016021; Integral component of membrane.

Kozak: TTCATGT ID=cds-XP_044378783.1;Parent=rna-XM_044522848.1;Dbxref=GeneID:123101354,Genbank:XP_044378783.1;Name=XP_044378783.1;gbkey=CDS;gene=LOC123101354;product=

Calmodulin-like protein 3.

Kozak: CAGATGC ID=cds-XP_044380047.1;Parent=rna-XM_044524112.1;Dbxref=GeneID:123102689,Genbank:XP_044380047.1;Name=XP_044380047.1;gbkey=CDS;gene=LOC123102689;product=

Probable leucine-rich repeat receptor-like protein kinase At1g35710.

KOZAK: CCAATGC ID=cds-XP_044384075.1;Parent=rna-XM_044528140.1;Dbxref=GeneID:123105956,Genbank:XP_044384075.1;Name=XP_044384075.1;gbkey=CDS;gene=LOC123105956;product=formin-like protein 14;protein_id=XP_044384075.1

GO:0051015; Actin filament binding.

Kozak: CCAATGT ID=cds-XP_044386030.1;Parent=rna-XM_044530095.1;Dbxref=GeneID:123108271,Genbank:XP_044386030.1;Name=XP_044386030.1;gbkey=CDS;gene=LOC123108271;product=protein Rf1%2C mitochondrial-like;protein_id=XP_044386030.1

GO:0010256; Endomembrane system organization.

Kozak:CCAATGT ID=cds-XP_044386303.1;Parent=rna-XM_044530368.1;Dbxref=GeneID:123108734,Genbank:XP_044386303.1;Name=XP_044386303.1;Note=The sequence of the model RefSeq protein was modified relative to this genomic sequence to represent the inferred CDS: added 1979 bases not found in genome assembly;exception=annotated by transcript or proteomic data;gbkey=CDS;gene=LOC123108734;inference=similar to RNA sequence (same species):INSD:GIJS01025333.1;partial=true;product=

Microtubule-associated protein futsch-like.

Kozak: TCCATGT ID=cds-XP_044388716.1;Parent=rna-XM_044532781.1;Dbxref=GeneID:123111898,Genbank:XP_044388716.1;Name=XP_044388716.1;gbkey=CDS;gene=LOC123111898;product=

Pentatricopeptide repeat-containing protein At3g05340-like.

Kozak: CCCATGC ID=cds-XP_044388846.1;Parent=rna-XM_044532911.1;Dbxref=GeneID:123112011,Genbank:XP_044388846.1;Name=XP_044388846.1;gbkey=CDS;gene=LOC123112011;product=

Pentatricopeptide repeat-containing protein At1g11900-like.

Kozak: CCAATGC ID=cds-XP_044396685.1;Parent=rna-XM_044540750.1;Dbxref=GeneID:123120750,Genbank:XP_044396685.1;Name=XP_044396685.1;gbkey=CDS;gene=LOC123120750;product=probable leucine-rich repeat receptor-like protein kinase At1g35710;protein_id=XP_044396685.1

GO:0016021; Integral component of membrane.

Kozak: TTCATGT ID=cds-XP_044398845.1;Parent=rna-XM_044542910.1;Dbxref=GeneID:123122620,Genbank:XP_044398845.1;Name=XP_044398845.1;gbkey=CDS;gene=LOC123122620;product=DNA ligase 1-like;protein_id=XP_044398845.1

GO:0005737; Cytoplasm.

Kozak: CCAATGC ID=cds-XP_044401077.1;Parent=rna-XM_044545142.1;Dbxref=GeneID:123124552,Genbank:XP_044401077.1;Name=XP_044401077.1;gbkey=CDS;gene=LOC123124552;product=uncharacterized protein LOC123124552;protein_id=XP_044401077.1

GO:0008375; Acetylglucosaminyltransferase activity.

Kozak: CCCATGT ID=cds-XP_044403814.1;Parent=rna-XM_044547879.1;Dbxref=GeneID:123127990,Genbank:XP_044403814.1;Name=XP_044403814.1;gbkey=CDS;gene=LOC123127990;product=

Haloacid dehalogenase-like hydrolase domain-containing protein Sgpp.

Kozak: CCAATGC ID=cds-XP_044408472.1;Parent=rna-XM_044552537.1;Dbxref=GeneID:123132798,Genbank:XP_044408472.1;Name=XP_044408472.1;gbkey=CDS;gene=LOC123132798;product=uncharacterized protein LOC123132798;protein_id=XP_044408472.1

GO:0009507; Chloroplast.

Kozak: TTCATGT ID=cds-XP_044414264.1;Parent=rna-XM_044558329.1;Dbxref=GeneID:123138361,Genbank:XP_044414264.1;Name=XP_044414264.1;gbkey=CDS;gene=LOC123138361;product=protein translation factor SUI1 homolog;protein_id=XP_044414264.1

GO:0003723; RNA binding.

Kozak: TTCATGT ID=cds-XP_044414436.1;Parent=rna-XM_044558501.1;Dbxref=GeneID:123138521,Genbank:XP_044414436.1;Name=XP_044414436.1;gbkey=CDS;gene=LOC123138521;product=peroxidase 1-like;protein_id=XP_044414436.1

GO:0005576; Eextracellular region.

Kozak: CAGATGC ID=cds-XP_044415365.1;Parent=rna-XM_044559430.1;Dbxref=GeneID:123139696,Genbank:XP_044415365.1;Name=XP_044415365.1;gbkey=CDS;gene=LOC123139696;product=regulator of MON1-CCZ1 complex-like;protein_id=XP_044415365.1

GO:0035658; Mon1-Ccz1 complex.

Kozak: CAGATGC ID=cds-XP_044420334.1;Parent=rna-XM_044564399.1;Dbxref=GeneID:123145079,Genbank:XP_044420334.1;Name=XP_044420334.1;gbkey=CDS;gene=LOC123145079;product=60S ribosomal protein L2%2C mitochondrial-like;protein_id=XP_044420334.1

GO:0015934; Large ribosomal subunit

Kozak: TACATGC ID=cds-XP_044420798.1;Parent=rna-XM_044564863.1;Dbxref=GeneID:123145446,Genbank:XP_044420798.1;Name=XP_044420798.1;gbkey=CDS;gene=LOC123145446;product=probable beta-1%2C4-xylosyltransferase GT43E;protein_id=XP_044420798.1

GO:0000139; Golgi membrane.

Kozak: TCCATGT ID=cds-XP_044425700.1;Parent=rna-XM_044569765.1;Dbxref=GeneID:123149969,Genbank:XP_044425700.1;Name=XP_044425700.1;gbkey=CDS;gene=LOC123149969;product=DEAD-box ATP-dependent RNA helicase 15-like;protein_id=XP_044425700.1

GO:0005524; ATP binding.

Kozak: TCCATGT ID=cds-XP_044423802.1;Parent=rna-XM_044567867.1;Dbxref=GeneID:100192150,Genbank:XP_044423802.1;Name=XP_044423802.1;gbkey=CDS;gene=LOC100192150;product=WRKY transcription factor 71 isoform X1;protein_id=XP_044423802.1

GO:0005634; Nucleus.

Kozak: CCCATGT ID=cds-XP_044359433.1;Parent=rna-XM_044503498.1;Dbxref=GeneID:123080566,Genbank:XP_044359433.1;Name=XP_044359433.1;gbkey=CDS;gene=LOC123080566;product=

BTB/POZ and MATH domain-containing protein 2-like.

Kozak: TCCATGT ID=cds-XP_044359896.1;Parent=rna-XM_044503961.1;Dbxref=GeneID:123081000,Genbank:XP_044359896.1;Name=XP_044359896.1;gbkey=CDS;gene=LOC123081000;product=fatty acyl-CoA reductase 2%2C chloroplastic-like;protein_id=XP_044359896.1

GO:0043231; Intracellular membrane-bounded organelle.

Kozak: CCCATGC ID=cds-XP_044361883.1;Parent=rna-XM_044505948.1;Dbxref=GeneID:123084236,Genbank:XP_044361883.1;Name=XP_044361883.1;gbkey=CDS;gene=LOC123084236;product=protein trichome birefringence-like 8;protein_id=XP_044361883.1

GO:0005794; Golgi apparatus.

Kozak: TTCATGT ID=cds-XP_044366868.1;Parent=rna-XM_044510933.1;Dbxref=GeneID:123088993,Genbank:XP_044366868.1;Name=XP_044366868.1;gbkey=CDS;gene=LOC123088993;product=

LOB domain-containing protein 15-like.

Kozak: CCCATGC ID=cds-XP_044369222.1;Parent=rna-XM_044513287.1;Dbxref=GeneID:123091688,Genbank:XP_044369222.1;Name=XP_044369222.1;gbkey=CDS;gene=LOC123091688;product=uncharacterized protein LOC123091688;protein_id=XP_044369222.1

GO:0005768; Endosome.

Kozak: CGAATGT ID=cds-XP_044370845.1;Parent=rna-XM_044514910.1;Dbxref=GeneID:123093033,Genbank:XP_044370845.1;Name=XP_044370845.1;gbkey=CDS;gene=LOC123093033;product=protein AUXIN-REGULATED GENE INVOLVED IN ORGAN SIZE-like;protein_id=XP_044370845.1

GO:0005737; Cytoplasm.

Kozak: TACATGC ID=cds-XP_044376278.1;Parent=rna-XM_044520343.1;Dbxref=GeneID:123098369,Genbank:XP_044376278.1;Name=XP_044376278.1;gbkey=CDS;gene=LOC123098369;product=probable purine permease 4;protein_id=XP_044376278.1

GO:0016021; Integral component of membrane.

Kozak: CCAATGT ID=cds-XP_044381658.1;Parent=rna-XM_044525723.1;Dbxref=GeneID:123104009,Genbank:XP_044381658.1;Name=XP_044381658.1;gbkey=CDS;gene=LOC123104009;product=subtilisin-chymotrypsin inhibitor-2B-like;protein_id=XP_044381658.1

GO:0004867; Serine-type endopeptidase inhibitor activity.

Kozak: CAGATGC ID=cds-XP_044381760.1;Parent=rna-XM_044525825.1;Dbxref=GeneID:123104094,Genbank:XP_044381760.1;Name=XP_044381760.1;gbkey=CDS;gene=LOC123104094;product=

Pumilio homolog 2-like.

Kozak: CAGATGC ID=cds-XP_044382043.1;Parent=rna-XM_044526108.1;Dbxref=GeneID:123104286,Genbank:XP_044382043.1;Name=XP_044382043.1;gbkey=CDS;gene=LOC123104286;product=60S ribosomal protein L2%2C mitochondrial-like;protein_id=XP_044382043.1

GO:0005737; Cytoplasm.

Kozak: CCCATGC ID=cds-XP_044382090.1;Parent=rna-XM_044526155.1;Dbxref=GeneID:123104326,Genbank:XP_044382090.1;Name=XP_044382090.1;gbkey=CDS;gene=LOC123104326;product=

Protein Rf1%2C mitochondrial-like.

Kozak: CCCATGC ID=cds-XP_044382385.1;Parent=rna-XM_044526450.1;Dbxref=GeneID:123104573,Genbank:XP_044382385.1;Name=XP_044382385.1;gbkey=CDS;gene=LOC123104573;product=pectin acetylesterase 5-like;protein_id=XP_044382385.1

GO:0005576; Extracellular region.

Kozak: TACATGC ID=cds-XP_044384130.1;Parent=rna-XM_044528195.1;Dbxref=GeneID:123105996,Genbank:XP_044384130.1;Name=XP_044384130.1;gbkey=CDS;gene=LOC123105996;product=protein trichome birefringence-like 10;protein_id=XP_044384130.1

GO:0005794; Golgi apparatus.

Kozak: CCAATGC ID=cds-XP_044386714.1;Parent=rna-XM_044530779.1;Dbxref=GeneID:123110296,Genbank:XP_044386714.1;Name=XP_044386714.1;gbkey=CDS;gene=LOC123110296;product=

Protein AGENET DOMAIN (AGD)-CONTAINING P1-like.

Kozak: CCAATGT ID=cds-XP_044391917.1;Parent=rna-XM_044535982.1;Dbxref=GeneID:123114486,Genbank:XP_044391917.1;Name=XP_044391917.1;gbkey=CDS;gene=LOC123114486;product=uncharacterized protein At2g34160-like;protein_id=XP_044391917.1

GO:0005634; Nucleus.

Kozak: CCAATGT ID=cds-XP_044392676.1;Parent=rna-XM_044536741.1;Dbxref=GeneID:123115623,Genbank:XP_044392676.1;Name=XP_044392676.1;gbkey=CDS;gene=LOC123115623;product=probable serine/threonine-protein kinase PBL7 isoform X1;protein_id=XP_044392676.1

GO:0016021; Integral component of membrane.

Kozak: TACATGC ID=cds-XP_044395081.1;Parent=rna-XM_044539146.1;Dbxref=GeneID:123119349,Genbank:XP_044395081.1;Name=XP_044395081.1;gbkey=CDS;gene=LOC123119349;product=protein fluG-like isoform X2;protein_id=XP_044395081.1

GO:0004356; Glutamate-ammonia ligase activity.

Kozak: CAGATGC ID=cds-XP_044396783.1;Parent=rna-XM_044540848.1;Dbxref=GeneID:123120865,Genbank:XP_044396783.1;Name=XP_044396783.1;gbkey=CDS;gene=LOC123120865;product=DExH-box ATP-dependent RNA helicase DExH1-like isoform X1;protein_id=XP_044396783.1

GO:0005634; Nucleus.

Kozak: CCCATGT ID=cds-XP_044397553.1;Parent=rna-XM_044541618.1;Dbxref=GeneID:123121597,Genbank:XP_044397553.1;Name=XP_044397553.1;gbkey=CDS;gene=LOC123121597;product=uncharacterized protein LOC123121597 isoform X1;protein_id=XP_044397553.1

GO:0016021; Integral component of membrane.

Kozak: CCAATGC ID=cds-XP_044401523.1;Parent=rna-XM_044545588.1;Dbxref=GeneID:123125052,Genbank:XP_044401523.1;Name=XP_044401523.1;gbkey=CDS;gene=LOC123125052;product=electron transfer flavoprotein-ubiquinone oxidoreductase%2C mitochondrial-like isoform X1;protein_id=XP_044401523.1

GO:0031305; Integral component of mitochondrial inner membrane.

Kozak: CCCATGT ID=cds-XP_044402679.1;Parent=rna-XM_044546744.1;Dbxref=GeneID:123126944,Genbank:XP_044402679.1;Name=XP_044402679.1;gbkey=CDS;gene=LOC123126944;product=

SUMO-conjugating enzyme SCE1-like.

Kozak: CCCATGT ID=cds-XP_044403232.1;Parent=rna-XM_044547297.1;Dbxref=GeneID:123127559,Genbank:XP_044403232.1;Name=XP_044403232.1;gbkey=CDS;gene=LOC123127559;product=tetratricopeptide repeat protein SKI3-like;protein_id=XP_044403232.1

GO:0055087; Ski complex.

Kozak. CCCATGC ID=cds-XP_044407117.1;Parent=rna-XM_044551182.1;Dbxref=GeneID:123131536,Genbank:XP_044407117.1;Name=XP_044407117.1;gbkey=CDS;gene=LOC123131536;product=argininosuccinate synthase%2C chloroplastic-like;protein_id=XP_044407117.1

GO:0005737; Cytoplasm.

Kozak: CAGATGC ID=cds-XP_044407468.1;Parent=rna-XM_044551533.1;Dbxref=GeneID:123131839,Genbank:XP_044407468.1;Name=XP_044407468.1;gbkey=CDS;gene=LOC123131839;product=probable E3 ubiquitin-protein ligase LUL4;protein_id=XP_044407468.1

GO:0061630; Ubiquitin protein ligase activity.

Kozak: TACATGC ID=cds-XP_044407633.1;Parent=rna-XM_044551698.1;Dbxref=GeneID:123131965,Genbank:XP_044407633.1;Name=XP_044407633.1;gbkey=CDS;gene=LOC123131965;product=

Vegetative cell wall protein gp1-like.

Kozak: CCCATGC ID=cds-XP_044409623.1;Parent=rna-XM_044553688.1;Dbxref=GeneID:123134434,Genbank:XP_044409623.1;Name=XP_044409623.1;gbkey=CDS;gene=LOC123134434;product=DAR GTPase 3%2C chloroplastic-like;protein_id=XP_044409623.1

GO:0005739; Mitochondrion.

Kozak: CCCATGC ID=cds-XP_044411205.1;Parent=rna-XM_044555270.1;Dbxref=GeneID:123135998,Genbank:XP_044411205.1;Name=XP_044411205.1;gbkey=CDS;gene=LOC123135998;product=

Coatomer subunit epsilon-2-like isoform X1.

Kozak: CAGATGC ID=cds-XP_044415234.1;Parent=rna-XM_044559299.1;Dbxref=GeneID:123139517,Genbank:XP_044415234.1;Name=XP_044415234.1;gbkey=CDS;gene=LOC123139517;product=

Protein FAR1-RELATED SEQUENCE 5-like.

Kozak: TACATGC ID=cds-XP_044417310.1;Parent=rna-XM_044561375.1;Dbxref=GeneID:123142465,Genbank:XP_044417310.1;Name=XP_044417310.1;gbkey=CDS;gene=LOC123142465;product=probable serine/threonine-protein kinase PBL7 isoform X2;protein_id=XP_044417310.1

GO:0005524; ATP binding.

Kozak: CCCATGC ID=cds-XP_044417661.1;Parent=rna-XM_044561726.1;Dbxref=GeneID:123142984,Genbank:XP_044417661.1;Name=XP_044417661.1;gbkey=CDS;gene=LOC123142984;product=uncharacterized protein LOC123142984;protein_id=XP_044417661.1

GO:0016021; Integral component of membrane.

Kozak: CCAATGC ID=cds-XP_044420321.1;Parent=rna-XM_044564386.1;Dbxref=GeneID:123145071,Genbank:XP_044420321.1;Name=XP_044420321.1;gbkey=CDS;gene=LOC123145071;product=

Formin-like protein 14.

Kozak: CCAATGC ID=cds-XP_044420702.1;Parent=rna-XM_044564767.1;Dbxref=GeneID:123145366,Genbank:XP_044420702.1;Name=XP_044420702.1;gbkey=CDS;gene=LOC123145366;product=auxin-responsive protein IAA19-like;protein_id=XP_044420702.1

GO:0005634; Nucleus.

Kozak: CGAATGT ID=cds-XP_044423545.1;Parent=rna-XM_044567610.1;Dbxref=GeneID:123148228,Genbank:XP_044423545.1;Name=XP_044423545.1;gbkey=CDS;gene=LOC123148228;product=glucose-1-phosphate adenylyltransferase large subunit%2C chloroplastic/amyloplastic-like;protein_id=XP_044423545.1

GO:0009501; Amyloplast.

Kozak: CCAATGC ID=cds-XP_044432368.1;Parent=rna-XM_044576433.1;Dbxref=GeneID:123158442,Genbank:XP_044432368.1;Name=XP_044432368.1;gbkey=CDS;gene=LOC123158442;product=

Probable fibrosin-1.

Kozak: CCCATGC ID=cds-XP_044433707.1;Parent=rna-XM_044577772.1;Dbxref=GeneID:123159959,Genbank:XP_044433707.1;Name=XP_044433707.1;gbkey=CDS;gene=LOC123159959;product=40S ribosomal protein S15-like;protein_id=XP_044433707.1

GO:0022627; Cytosolic small ribosomal subunit.

Kozak: TCCATGT ID=cds-XP_044434463.1;Parent=rna-XM_044578528.1;Dbxref=GeneID:123160678,Genbank:XP_044434463.1;Name=XP_044434463.1;gbkey=CDS;gene=LOC123160678;product=

CDP-diacylglycerol--serine O-phosphatidyltransferase 2-like.

Kozak: TCCATGT ID=cds-XP_044442017.1;Parent=rna-XM_044586082.1;Dbxref=GeneID:123168206,Genbank:XP_044442017.1;Name=XP_044442017.1;gbkey=CDS;gene=LOC123168206;product=protein DETOXIFICATION 49-like;protein_id=XP_044442017.1

GO:0016021; Integral component of membrane.

Kozak: CAGATGC ID=cds-XP_044442446.1;Parent=rna-XM_044586511.1;Dbxref=GeneID:123168627,Genbank:XP_044442446.1;Name=XP_044442446.1;gbkey=CDS;gene=LOC123168627;product=ubiquitin-NEDD8-like protein RUB2;protein_id=XP_044442446.1

GO:0005737; Cytoplasm.

Kozak: CAGATGC ID=cds-XP_044444988.1;Parent=rna-XM_044589053.1;Dbxref=GeneID:123171728,Genbank:XP_044444988.1;Name=XP_044444988.1;gbkey=CDS;gene=LOC123171728;product=blue copper protein 1a-like;protein_id=XP_044444988.1

GO:0046658; Anchored component of plasma membrane.

Kozak: CCCATGC ID=cds-XP_044446029.1;Parent=rna-XM_044590094.1;Dbxref=GeneID:123174826,Genbank:XP_044446029.1;Name=XP_044446029.1;gbkey=CDS;gene=LOC123174826;product=

Pentatricopeptide repeat-containing protein At1g07740%2C mitochondrial-like.

Kozak: TCCATGT ID=cds-XP_044446212.1;Parent=rna-XM_044590277.1;Dbxref=GeneID:123175679,Genbank:XP_044446212.1;Name=XP_044446212.1;gbkey=CDS;gene=LOC123175679;product=BTB/POZ and MATH domain-containing protein 2-like;protein_id=XP_044446212.1

GO:0016567; Protein ubiquitination.

Kozak: TACATGC ID=cds-XP_044446408.1;Parent=rna-XM_044590473.1;Dbxref=GeneID:123176080,Genbank:XP_044446408.1;Name=XP_044446408.1;gbkey=CDS;gene=LOC123176080;product=probable purine permease 4;protein_id=XP_044446408.1

GO:0016021; Integral component of membrane.

Kozak: TCCATGT ID=cds-XP_044446782.1;Parent=rna-XM_044590847.1;Dbxref=GeneID:123176785,Genbank:XP_044446782.1;Name=XP_044446782.1;gbkey=CDS;gene=LOC123176785;product=

WRKY transcription factor 28-like.

Kozak: CAGATGC ID=cds-XP_044447322.1;Parent=rna-XM_044591387.1;Dbxref=GeneID:123178059,Genbank:XP_044447322.1;Name=XP_044447322.1;gbkey=CDS;gene=LOC123178059;product=

E3 ubiquitin-protein ligase WAV3-like.

Kozak: TTCATGT ID=cds-XP_044447443.1;Parent=rna-XM_044591508.1;Dbxref=GeneID:123178855,Genbank:XP_044447443.1;Name=XP_044447443.1;gbkey=CDS;gene=LOC123178855;product=

Calmodulin-like protein 3.

Kozak: CCCATGC ID=cds-XP_044447665.1;Parent=rna-XM_044591730.1;Dbxref=GeneID:123179837,Genbank:XP_044447665.1;Name=XP_044447665.1;Note=The sequence of the model RefSeq protein was modified relative to this genomic sequence to represent the inferred CDS: added 105 bases not found in genome assembly;end_range=7331937,.;exception=annotated by transcript or proteomic data;gbkey=CDS;gene=LOC123179837;inference=similar to RNA sequence%2C mRNA (same species):INSD:GAEF01105328.1;partial=true;product=G-type lectin S-receptor-like serine/threonine-protein kinase At2g19130;protein_id=XP_044447665.1

GO:0016021; Integral component of membrane.

Kozak: CAGATGC ID=cds-XP_044448049.1;Parent=rna-XM_044592114.1;Dbxref=GeneID:123180145,Genbank:XP_044448049.1;Name=XP_044448049.1;gbkey=CDS;gene=LOC123180145;product=ribonuclease II%2C chloroplastic/mitochondrial-like;protein_id=XP_044448049.1

GO:0005739; Mitochondrion.

Kozak: CCAATGC ID=cds-XP_044448142.1;Parent=rna-XM_044592207.1;Dbxref=GeneID:123180224,Genbank:XP_044448142.1;Name=XP_044448142.1;gbkey=CDS;gene=LOC123180224;product=L-type lectin-domain containing receptor kinase VIII.2-like;protein_id=XP_044448142.1

GO:0016021; Integral component of membrane.

Kozak: TCCATGT ID=cds-XP_044448435.1;Parent=rna-XM_044592500.1;Dbxref=GeneID:123180451,Genbank:XP_044448435.1;Name=XP_044448435.1;gbkey=CDS;gene=LOC123180451;product=

Pentatricopeptide repeat-containing protein At3g05340-like.

Kozak: CCCATGC ID=cds-XP_044448441.1;Parent=rna-XM_044592506.1;Dbxref=GeneID:123180456,Genbank:XP_044448441.1;Name=XP_044448441.1;gbkey=CDS;gene=LOC123180456;product=

Pentatricopeptide repeat-containing protein At1g11900-like.

Kozak: CCAATGC ID=cds-XP_044449135.1;Parent=rna-XM_044593200.1;Dbxref=GeneID:123180995,Genbank:XP_044449135.1;Name=XP_044449135.1;gbkey=CDS;gene=LOC123180995;product=probable leucine-rich repeat receptor-like protein kinase At1g35710;protein_id=XP_044449135.1

GO:0016021; Integral component of membrane.

Kozak: TCCATGT ID=cds-XP_044449226.1;Parent=rna-XM_044593291.1;Dbxref=GeneID:123181074,Genbank:XP_044449226.1;Name=XP_044449226.1;gbkey=CDS;gene=LOC123181074;product=F-box/kelch-repeat protein At5g42350-like;protein_id=XP_044449226.1

GO:0019005; SCF ubiquitin ligase complex.

Kozak: CCCATGT ID=cds-XP_044449321.1;Parent=rna-XM_044593386.1;Dbxref=GeneID:123181151,Genbank:XP_044449321.1;Name=XP_044449321.1;gbkey=CDS;gene=LOC123181151;product=

Ubiquitin C-terminal hydrolase 12-like.

Kozak: CCAATGC ID=cds-XP_044449353.1;Parent=rna-XM_044593418.1;Dbxref=GeneID:123181172,Genbank:XP_044449353.1;Name=XP_044449353.1;gbkey=CDS;gene=LOC123181172;product=electron transfer flavoprotein-ubiquinone oxidoreductase%2C mitochondrial isoform X1;protein_id=XP_044449353.1

GO:0031305; Integral component of mitochondrial inner membrane.

Kozak: TCCATGT ID=cds-XP_044449686.1;Parent=rna-XM_044593751.1;Dbxref=GeneID:123181485,Genbank:XP_044449686.1;Name=XP_044449686.1;gbkey=CDS;gene=LOC123181485;product=phenylalanine--tRNA ligase beta subunit%2C cytoplasmic-like isoform X1;protein_id=XP_044449686.1

GO:0009328; Phenylalanine-tRNA ligase complex.

Kozak: CCCATGT ID=cds-XP_044449972.1;Parent=rna-XM_044594037.1;Dbxref=GeneID:123181713,Genbank:XP_044449972.1;Name=XP_044449972.1;gbkey=CDS;gene=LOC123181713;product=probable inactive receptor-like protein kinase At3g56050;protein_id=XP_044449972.1

GO:0016021; Integral component of membrane.

Kozak: CCAATGC ID=cds-XP_044450386.1;Parent=rna-XM_044594451.1;Dbxref=GeneID:123182018,Genbank:XP_044450386.1;Name=XP_044450386.1;gbkey=CDS;gene=LOC123182018;product=transcription termination factor MTERF4%2C chloroplastic-like isoform X1;protein_id=XP_044450386.1

GO:0009507; Chloroplast.

Kozak: TTCATGT ID=cds-XP_044451010.1;Parent=rna-XM_044595075.1;Dbxref=GeneID:123182496,Genbank:XP_044451010.1;Name=XP_044451010.1;gbkey=CDS;gene=LOC123182496;product=protein translation factor SUI1 homolog;protein_id=XP_044451010.1

GO:0003723; RNA binding.

Kozak: CAGATGC ID=cds-XP_044451162.1;Parent=rna-XM_044595227.1;Dbxref=GeneID:123182597,Genbank:XP_044451162.1;Name=XP_044451162.1;gbkey=CDS;gene=LOC123182597;product=regulator of MON1-CCZ1 complex-like;protein_id=XP_044451162.1

GO:0035658; Mon1-Ccz1 complex.

Kozak: TCCATGT ID=cds-XP_044451220.1;Parent=rna-XM_044595285.1;Dbxref=GeneID:123182642,Genbank:XP_044451220.1;Name=XP_044451220.1;gbkey=CDS;gene=LOC123182642;product=proline-rich receptor-like protein kinase PERK2;protein_id=XP_044451220.1

GO:0005524; ATP binding.

Kozak: CCAATGT ID=cds-XP_044451297.1;Parent=rna-XM_044595362.1;Dbxref=GeneID:123182704,Genbank:XP_044451297.1;Name=XP_044451297.1;gbkey=CDS;gene=LOC123182704;product=anthocyanidin 3-O-glucosyltransferase 2-like;protein_id=XP_044451297.1

GO:0008194; UDP-glycosyltransferase activity.

Kozak: CCCATGT ID=cds-XP_044451474.1;Parent=rna-XM_044595539.1;Dbxref=GeneID:123182865,Genbank:XP_044451474.1;Name=XP_044451474.1;gbkey=CDS;gene=LOC123182865;product=

Transcription factor GTE1-like.

Kozak: CCCATGT ID=cds-XP_044451726.1;Parent=rna-XM_044595791.1;Dbxref=GeneID:123183056,Genbank:XP_044451726.1;Name=XP_044451726.1;gbkey=CDS;gene=LOC123183056;product=cell number regulator 2-like;protein_id=XP_044451726.1

GO:0016021; Integral component of membrane.

Kozak: TACATGC ID=cds-XP_044451728.1;Parent=rna-XM_044595793.1;Dbxref=GeneID:123183058,Genbank:XP_044451728.1;Name=XP_044451728.1;gbkey=CDS;gene=LOC123183058;product=probable beta-1%2C4-xylosyltransferase GT43E;protein_id=XP_044451728.1

GO:0000139; Golgi membrane.

Kozak: TCCATGT ID=cds-XP_044452170.1;Parent=rna-XM_044596235.1;Dbxref=GeneID:123183423,Genbank:XP_044452170.1;Name=XP_044452170.1;gbkey=CDS;gene=LOC123183423;product=DEAD-box ATP-dependent RNA helicase 15-like;protein_id=XP_044452170.1

GO:0005524; ATP binding.

Kozak: CAGATGC ID=cds-XP_044447640.1;Parent=rna-XM_044591705.1;Dbxref=GeneID:780602,Genbank:XP_044447640.1;Name=XP_044447640.1;gbkey=CDS;gene=LOC780602;product=60S ribosomal protein L2%2C mitochondrial;protein_id=XP_044447640.1

GO:0005762; Mitochondrial large ribosomal subunit.

Kozak: TTCATGT ID=cds-XP_044432913.1;Parent=rna-XM_044576978.1;Dbxref=GeneID:123159153,Genbank:XP_044432913.1;Name=XP_044432913.1;gbkey=CDS;gene=LOC123159153;product=protein synthesis inhibitor II-like;protein_id=XP_044432913.1

GO:0030598; rRNA N-glycosylase activity.

Kozak: CAGATGC ID=cds-XP_044433805.1;Parent=rna-XM_044577870.1;Dbxref=GeneID:123160043,Genbank:XP_044433805.1;Name=XP_044433805.1;gbkey=CDS;gene=LOC123160043;product=beta-arabinofuranosyltransferase RAY1-like;protein_id=XP_044433805.1

GO:0016757; Gycosyltransferase activity.

Kozak: TACATGC ID=cds-XP_044434715.1;Parent=rna-XM_044578780.1;Dbxref=GeneID:123160926,Genbank:XP_044434715.1;Name=XP_044434715.1;gbkey=CDS;gene=LOC123160926;product=

dnaJ homolog subfamily B member 4-like.

Kozak: CCCATGC ID=cds-XP_044436908.1;Parent=rna-XM_044580973.1;Dbxref=GeneID:123163371,Genbank:XP_044436908.1;Name=XP_044436908.1;gbkey=CDS;gene=LOC123163371;product=

F-box protein At1g30790-like.

Kozak: CCCATGC ID=cds-XP_044439833.1;Parent=rna-XM_044583898.1;Dbxref=GeneID:123166129,Genbank:XP_044439833.1;Name=XP_044439833.1;gbkey=CDS;gene=LOC123166129;product=

4-hydroxyphenylpyruvate dioxygenase-like.

Kozak: CCAATGT ID=cds-XP_044440265.1;Parent=rna-XM_044584330.1;Dbxref=GeneID:123166519,Genbank:XP_044440265.1;Name=XP_044440265.1;gbkey=CDS;gene=LOC123166519;product=uncharacterized protein At2g34160-like;protein_id=XP_044440265.1

GO:0005634; Nucleus.

Kozak: CCCATGC ID=cds-XP_044441953.1;Parent=rna-XM_044586018.1;Dbxref=GeneID:123168142,Genbank:XP_044441953.1;Name=XP_044441953.1;gbkey=CDS;gene=LOC123168142;product=uncharacterized protein LOC123168142;protein_id=XP_044441953.1

GO:0016021; Integral component of membrane.

Kozak: CCCATGC ID=cds-XP_044442379.1;Parent=rna-XM_044586444.1;Dbxref=GeneID:123168554,Genbank:XP_044442379.1;Name=XP_044442379.1;gbkey=CDS;gene=LOC123168554;product=uncharacterized protein LOC123168554;protein_id=XP_044442379.1

GO:0005768; Endosome.

Kozak: CCCATGT ID=cds-XP_044443617.1;Parent=rna-XM_044587682.1;Dbxref=GeneID:123169806,Genbank:XP_044443617.1;Name=XP_044443617.1;gbkey=CDS;gene=LOC123169806;product=methyl-CpG-binding domain-containing protein 4-like;protein_id=XP_044443617.1

GO:0005634; Nucleus.

Kozak: CGAATGT ID=cds-XP_044443750.1;Parent=rna-XM_044587815.1;Dbxref=GeneID:123169960,Genbank:XP_044443750.1;Name=XP_044443750.1;gbkey=CDS;gene=LOC123169960;product=protein AUXIN-REGULATED GENE INVOLVED IN ORGAN SIZE-like;protein_id=XP_044443750.1

GO:0005737; Cytoplasm.

Kozak: TACATGC ID=cds-XP_044445985.1;Parent=rna-XM_044590050.1;Dbxref=GeneID:123174475,Genbank:XP_044445985.1;Name=XP_044445985.1;gbkey=CDS;gene=LOC123174475;product=

Mannose/glucose-specific lectin-like.

Kozak TACATGC ID=cds-XP_044446391.1;Parent=rna-XM_044590456.1;Dbxref=GeneID:123176058,Genbank:XP_044446391.1;Name=XP_044446391.1;gbkey=CDS;gene=LOC123176058;product=probable purine permease 4;protein_id=XP_044446391.1

GO:0016021; Integral component of membrane.

Kozak: CCCATGT ID=cds-XP_044447267.1;Parent=rna-XM_044591332.1;Dbxref=GeneID:123177860,Genbank:XP_044447267.1;Name=XP_044447267.1;gbkey=CDS;gene=LOC123177860;product=WRKY transcription factor 71-like;protein_id=XP_044447267.1

GO:0005634; Nucleus.

Kozak: TCCATGT ID=cds-XP_044447499.1;Parent=rna-XM_044591564.1;Dbxref=GeneID:123179475,Genbank:XP_044447499.1;Name=XP_044447499.1;gbkey=CDS;gene=LOC123179475;product=uncharacterized protein LOC123179475;protein_id=XP_044447499.1

GO:0016021; Integral component of membrane.

Kozak: TACATGC ID=cds-XP_044447596.1;Parent=rna-XM_044591661.1;Dbxref=GeneID:123179783,Genbank:XP_044447596.1;Name=XP_044447596.1;gbkey=CDS;gene=LOC123179783;product=

MDIS1-interacting receptor like kinase 2-like.

Kozak: CCAATGC ID=cds-XP_044447639.1;Parent=rna-XM_044591704.1;Dbxref=GeneID:123179813,Genbank:XP_044447639.1;Name=XP_044447639.1;gbkey=CDS;gene=LOC123179813;product=formin-like protein 14;protein_id=XP_044447639.1

GO:0051015; Aactin filament binding.

Kozak: CCCATGC ID=cds-XP_044447657.1;Parent=rna-XM_044591722.1;Dbxref=GeneID:123179830,Genbank:XP_044447657.1;Name=XP_044447657.1;gbkey=CDS;gene=LOC123179830;product=G-type lectin S-receptor-like serine/threonine-protein kinase At2g19130;protein_id=XP_044447657.1

GO:0016021; Integral component of membrane.

Kozak: CCCATGC ID=cds-XP_044447698.1;Parent=rna-XM_044591763.1;Dbxref=GeneID:123179866,Genbank:XP_044447698.1;Name=XP_044447698.1;gbkey=CDS;gene=LOC123179866;product=protein trichome birefringence-like 8;protein_id=XP_044447698.1

GO:0005794; Golgi apparatus.

Kozak: CCAATGT ID=cds-XP_044447758.1;Parent=rna-XM_044591823.1;Dbxref=GeneID:123179913,Genbank:XP_044447758.1;Name=XP_044447758.1;gbkey=CDS;gene=LOC123179913;product=subtilisin-chymotrypsin inhibitor-2B-like;protein_id=XP_044447758.1

GO:0004867; Serine-type endopeptidase inhibitor activity.

Kozak: CCCATGC ID=cds-XP_044447854.1;Parent=rna-XM_044591919.1;Dbxref=GeneID:123179979,Genbank:XP_044447854.1;Name=XP_044447854.1;gbkey=CDS;gene=LOC123179979;product=pectin acetylesterase 5-like;protein_id=XP_044447854.1

GO:0005576; Extracellular region.

Kozak: CCAATGT ID=cds-XP_044448030.1;Parent=rna-XM_044592095.1;Dbxref=GeneID:123180132,Genbank:XP_044448030.1;Name=XP_044448030.1;gbkey=CDS;gene=LOC123180132;product=GDSL esterase/lipase LIP-4-like isoform X1;protein_id=XP_044448030.1

GO:0016788; Hydrolase activity.

Kozak: CAGATGC ID=cds-XP_044448096.1;Parent=rna-XM_044592161.1;Dbxref=GeneID:123180190,Genbank:XP_044448096.1;Name=XP_044448096.1;gbkey=CDS;gene=LOC123180190;product=uncharacterized protein LOC123180190 isoform X3;protein_id=XP_044448096.1

GO:0032196; Transposition.

Kozak: CCAATGC ID=cds-XP_044448268.1;Parent=rna-XM_044592333.1;Dbxref=GeneID:123180315,Genbank:XP_044448268.1;Name=XP_044448268.1;gbkey=CDS;gene=LOC123180315;product=

Protein AGENET DOMAIN (AGD)-CONTAINING P1-like isoform X1.

Kozak: TTCATGT ID=cds-XP_044448652.1;Parent=rna-XM_044592717.1;Dbxref=GeneID:123180633,Genbank:XP_044448652.1;Name=XP_044448652.1;gbkey=CDS;gene=LOC123180633;product=

LOB domain-containing protein 15-like.

Kozak: CCAATGT ID=cds-XP_044448861.1;Parent=rna-XM_044592926.1;Dbxref=GeneID:123180790,Genbank:XP_044448861.1;Name=XP_044448861.1;gbkey=CDS;gene=LOC123180790;product=probable serine/threonine-protein kinase PBL7 isoform X1;protein_id=XP_044448861.1

GO:0016021; Integral component of membrane.

Kozak: CCCATGT ID=cds-XP_044449255.1;Parent=rna-XM_044593320.1;Dbxref=GeneID:123181096,Genbank:XP_044449255.1;Name=XP_044449255.1;gbkey=CDS;gene=LOC123181096;product=uncharacterized protein LOC123181096 isoform X1;protein_id=XP_044449255.1

GO:0016021; Integral component of membrane.

Kozak: TTCATGT ID=cds-XP_044449461.1;Parent=rna-XM_044593526.1;Dbxref=GeneID:123181261,Genbank:XP_044449461.1;Name=XP_044449461.1;gbkey=CDS;gene=LOC123181261;product=DNA ligase 1-like;protein_id=XP_044449461.1

GO:0005737; Cytoplasm.

Kozak: CCAATGC ID=cds-XP_044449514.1;Parent=rna-XM_044593579.1;Dbxref=GeneID:123181318,Genbank:XP_044449514.1;Name=XP_044449514.1;gbkey=CDS;gene=LOC123181318;product=uncharacterized protein LOC123181318;protein_id=XP_044449514.1

GO:0008375; Acetylglucosaminyltransferase activity.

Kozak: CCCATGT ID=cds-XP_044449742.1;Parent=rna-XM_044593807.1;Dbxref=GeneID:123181527,Genbank:XP_044449742.1;Name=XP_044449742.1;gbkey=CDS;gene=LOC123181527;product=

SUMO-conjugating enzyme SCE1-like.

Kozak: CCCATGC ID=cds-XP_044449973.1;Parent=rna-XM_044594038.1;Dbxref=GeneID:123181714,Genbank:XP_044449973.1;Name=XP_044449973.1;gbkey=CDS;gene=LOC123181714;product=

N-myc 2 proto-oncogene protein-like.

Kozak: CCAATGT ID=cds-XP_044450107.1;Parent=rna-XM_044594172.1;Dbxref=GeneID:123181802,Genbank:XP_044450107.1;Name=XP_044450107.1;gbkey=CDS;gene=LOC123181802;product=pentatricopeptide repeat-containing protein At1g20230-like;protein_id=XP_044450107.1

GO:0008270; Zinc ion binding.

Kozak: CCCATGC ID=cds-XP_044450244.1;Parent=rna-XM_044594309.1;Dbxref=GeneID:123181902,Genbank:XP_044450244.1;Name=XP_044450244.1;gbkey=CDS;gene=LOC123181902;product=argininosuccinate synthase%2C chloroplastic-like;protein_id=XP_044450244.1

GO:0005737; Cytoplasm.

Kozak: CAGATGC ID=cds-XP_044450268.1;Parent=rna-XM_044594333.1;Dbxref=GeneID:123181925,Genbank:XP_044450268.1;Name=XP_044450268.1;gbkey=CDS;gene=LOC123181925;product=probable E3 ubiquitin-protein ligase LUL4;protein_id=XP_044450268.1

GO:0061630; Ubiquitin protein ligase activity.

Kozak: CCCATGC ID=cds-XP_044450521.1;Parent=rna-XM_044594586.1;Dbxref=GeneID:123182121,Genbank:XP_044450521.1;Name=XP_044450521.1;gbkey=CDS;gene=LOC123182121;product=DAR GTPase 3%2C chloroplastic-like;protein_id=XP_044450521.1

GO:0005739; Mitochondrion.

Kozak: CAGATGC ID=cds-XP_044450534.1;Parent=rna-XM_044594599.1;Dbxref=GeneID:123182131,Genbank:XP_044450534.1;Name=XP_044450534.1;gbkey=CDS;gene=LOC123182131;product=embryogenesis-associated protein EMB8-like isoform X1;protein_id=XP_044450534.1

GO:0016021; Integral component of membrane.

Kozak: CCCATGC ID=cds-XP_044450714.1;Parent=rna-XM_044594779.1;Dbxref=GeneID:123182268,Genbank:XP_044450714.1;Name=XP_044450714.1;gbkey=CDS;gene=LOC123182268;product=probable monofunctional riboflavin biosynthesis protein RIBA 3%2C chloroplastic isoform X2;protein_id=XP_044450714.1

GO:0009507; Chloroplast.

Kozak: CAGATGC ID=cds-XP_044451145.1;Parent=rna-XM_044595210.1;Dbxref=GeneID:123182579,Genbank:XP_044451145.1;Name=XP_044451145.1;gbkey=CDS;gene=LOC123182579;product=

Protein FAR1-RELATED SEQUENCE 5-like.

Kozak: CCCATGC ID=cds-XP_044451447.1;Parent=rna-XM_044595512.1;Dbxref=GeneID:123182840,Genbank:XP_044451447.1;Name=XP_044451447.1;gbkey=CDS;gene=LOC123182840;product=uncharacterized protein LOC123182840;protein_id=XP_044451447.1

GO:0005634; Nucleus.

Kozak: CCAATGC ID=cds-XP_044451721.1;Parent=rna-XM_044595786.1;Dbxref=GeneID:123183053,Genbank:XP_044451721.1;Name=XP_044451721.1;gbkey=CDS;gene=LOC123183053;product=auxin-responsive protein IAA19-like;protein_id=XP_044451721.1

GO:0005634; Nucleus.

Kozak: CGAATGT ID=cds-XP_044451957.1;Parent=rna-XM_044596022.1;Dbxref=GeneID:123183253,Genbank:XP_044451957.1;Name=XP_044451957.1;gbkey=CDS;gene=LOC123183253;product=glucose-1-phosphate adenylyltransferase large subunit%2C chloroplastic/amyloplastic;protein_id=XP_044451957.1

GO:0009501; Amyloplast.

Kozak: TCCATGT ID=cds-XP_044451976.1;Parent=rna-XM_044596041.1;Dbxref=GeneID:123183268,Genbank:XP_044451976.1;Name=XP_044451976.1;gbkey=CDS;gene=LOC123183268;product=WRKY transcription factor 28-like;protein_id=XP_044451976.1

GO:0005634; Nucleus.

Kozak: CCAATGT ID=cds-XP_044320170.1;Parent=rna-XM_044464235.1;Dbxref=GeneID:123041647,Genbank:XP_044320170.1;Name=XP_044320170.1;gbkey=CDS;gene=LOC123041647;product=probable serine/threonine-protein kinase PBL7 isoform X1;protein_id=XP_044320170.1

GO:0016021; Integral component of membrane.

Kozak: CCAATGC ID=cds-XP_044323952.1;Parent=rna-XM_044468017.1;Dbxref=GeneID:123045082,Genbank:XP_044323952.1;Name=XP_044323952.1;gbkey=CDS;gene=LOC123045082;product=probable leucine-rich repeat receptor-like protein kinase At1g35710;protein_id=XP_044323952.1

GO:0016021; Integral component of membrane.

Kozak: CCAATGC ID=cds-XP_044327755.1;Parent=rna-XM_044471820.1;Dbxref=GeneID:123048783,Genbank:XP_044327755.1;Name=XP_044327755.1;gbkey=CDS;gene=LOC123048783;product=uncharacterized protein LOC123048783;protein_id=XP_044327755.1

GO:0008375; Acetylglucosaminyltransferase activity.

Kozak: CCCATGT ID=cds-XP_044330905.1;Parent=rna-XM_044474970.1;Dbxref=GeneID:123051953,Genbank:XP_044330905.1;Name=XP_044330905.1;gbkey=CDS;gene=LOC123051953;product=

Haloacid dehalogenase-like hydrolase domain-containing protein Sgpp.

Kozak: TCCATGT ID=cds-XP_044334039.1;Parent=rna-XM_044478104.1;Dbxref=GeneID:123054345,Genbank:XP_044334039.1;Name=XP_044334039.1;gbkey=CDS;gene=LOC123054345;product=probable transmembrane GTPase FZO-like%2C chloroplastic;protein_id=XP_044334039.1

GO:0003824; Catalytic activity.

Kozak: CCAATGC ID=cds-XP_044335506.1;Parent=rna-XM_044479571.1;Dbxref=GeneID:123055646,Genbank:XP_044335506.1;Name=XP_044335506.1;gbkey=CDS;gene=LOC123055646;product=uncharacterized protein LOC123055646;protein_id=XP_044335506.1

GO:0009507; Chloroplast.

Kozak: TCCATGT ID=cds-XP_044337486.1;Parent=rna-XM_044481551.1;Dbxref=GeneID:123058866,Genbank:XP_044337486.1;Name=XP_044337486.1;gbkey=CDS;gene=LOC123058866;product=proteasome subunit beta type-5-B-like;protein_id=XP_044337486.1

GO:0005634; Nucleus.

Kozak: TTCATGT ID=cds-XP_044340135.1;Parent=rna-XM_044484200.1;Dbxref=GeneID:123061213,Genbank:XP_044340135.1;Name=XP_044340135.1;gbkey=CDS;gene=LOC123061213;product=protein translation factor SUI1 homolog;protein_id=XP_044340135.1

GO:0003723; RNA binding.

Kozak: TTCATGT ID=cds-XP_044340425.1;Parent=rna-XM_044484490.1;Dbxref=GeneID:123061400,Genbank:XP_044340425.1;Name=XP_044340425.1;gbkey=CDS;gene=LOC123061400;product=peroxidase 1-like;protein_id=XP_044340425.1

GO:0005576; Extracellular region.

Kozak: CAGATGC ID=cds-XP_044341895.1;Parent=rna-XM_044485960.1;Dbxref=GeneID:123062436,Genbank:XP_044341895.1;Name=XP_044341895.1;gbkey=CDS;gene=LOC123062436;product=regulator of MON1-CCZ1 complex-like;protein_id=XP_044341895.1

GO:0035658; Mon1-Ccz1 complex.

Kozak: TTCATGT ID=cds-XP_044342083.1;Parent=rna-XM_044486148.1;Dbxref=GeneID:123062565,Genbank:XP_044342083.1;Name=XP_044342083.1;gbkey=CDS;gene=LOC123062565;product=uncharacterized protein LOC123062565 isoform X1;protein_id=XP_044342083.1

GO:0016021; Integral component of membrane.

Kozak: TCCATGT ID=cds-XP_044342936.1;Parent=rna-XM_044487001.1;Dbxref=GeneID:123063258,Genbank:XP_044342936.1;Name=XP_044342936.1;gbkey=CDS;gene=LOC123063258;product=

Proline-rich receptor-like protein kinase PERK2.

Kozak: CCAATGC ID=cds-XP_044346181.1;Parent=rna-XM_044490246.1;Dbxref=GeneID:123067458,Genbank:XP_044346181.1;Name=XP_044346181.1;gbkey=CDS;gene=LOC123067458;product=uncharacterized protein LOC123067458;protein_id=XP_044346181.1

GO:0016021; Integral component of membrane.

Kozak: TACATGC ID=cds-XP_044346409.1;Parent=rna-XM_044490474.1;Dbxref=GeneID:123067864,Genbank:XP_044346409.1;Name=XP_044346409.1;gbkey=CDS;gene=LOC123067864;product=probable beta-1%2C4-xylosyltransferase GT43E;protein_id=XP_044346409.1

GO:0000139; Golgi membrane.

Kozak: TCCATGT ID=cds-XP_044350467.1;Parent=rna-XM_044494532.1;Dbxref=GeneID:123071061,Genbank:XP_044350467.1;Name=XP_044350467.1;gbkey=CDS;gene=LOC123071061;product=DEAD-box ATP-dependent RNA helicase 56-like;protein_id=XP_044350467.1

GO:0005524; ATP binding.

Kozak: CCAATGC ID=cds-XP_044410007.1;Parent=rna-XM_044554072.1;Dbxref=GeneID:123134891,Genbank:XP_044410007.1;Name=XP_044410007.1;gbkey=CDS;gene=LOC123134891;product=beta-fructofuranosidase%2C insoluble isoenzyme 3-like;protein_id=XP_044410007.1

GO:0004553; Hydrolase activity, hydrolyzing O-glycosyl compounds.

Kozak: TACATGC ID=cds-XP_044449206.1;Parent=rna-XM_044593271.1;Dbxref=GeneID:123181050,Genbank:XP_044449206.1;Name=XP_044449206.1;gbkey=CDS;gene=LOC123181050;product=probable purine permease 4;protein_id=XP_044449206.1

GO:0016021; Integral component of membrane.

Kozak: TCCATGT ID=cds-XP_044449884.1;Parent=rna-XM_044593949.1;Dbxref=GeneID:123181632,Genbank:XP_044449884.1;Name=XP_044449884.1;gbkey=CDS;gene=LOC123181632;product=WRKY transcription factor 28-like;protein_id=XP_044449884.1

GO:0005634; Nucleus.

Kozak: CAGATGC ID=cds-XP_044450882.1;Parent=rna-XM_044594947.1;Dbxref=GeneID:123182399,Genbank:XP_044450882.1;Name=XP_044450882.1;gbkey=CDS;gene=LOC123182399;product=

Leucine-rich repeat extensin-like protein 5.

Kozak: TTCATGT ID=cds-XP_044451739.1;Parent=rna-XM_044595804.1;Dbxref=GeneID:123183066,Genbank:XP_044451739.1;Name=XP_044451739.1;gbkey=CDS;gene=LOC123183066;product=probable calcium-binding protein CML27;protein_id=XP_044451739.1

GO:0005509; Calcium ion binding.

Kozak: TTCATGT ID=cds-XP_044451749.1;Parent=rna-XM_044595814.1;Dbxref=GeneID:123183077,Genbank:XP_044451749.1;Name=XP_044451749.1;gbkey=CDS;gene=LOC123183077;product=calmodulin-like protein 3;protein_id=XP_044451749.1

GO:0016021; Integral component of membrane.

Kozak: TTCATGT ID=cds-XP_044451894.1;Parent=rna-XM_044595959.1;Dbxref=GeneID:123183190,Genbank:XP_044451894.1;Name=XP_044451894.1;gbkey=CDS;gene=LOC123183190;product=uncharacterized protein LOC123183190 isoform X3;protein_id=XP_044451894.1

GO:0016021; Integral component of membrane.

Kozak: CCCATGC ID=cds-XP_044453242.1;Parent=rna-XM_044597307.1;Dbxref=GeneID:123185427,Genbank:XP_044453242.1;Name=XP_044453242.1;gbkey=CDS;gene=LOC123185427;product=G-type lectin S-receptor-like serine/threonine-protein kinase At2g19130;protein_id=XP_044453242.1

GO:0016021; Integral component of membrane.

Kozak: CCAATGT ID=cds-XP_044454592.1;Parent=rna-XM_044598657.1;Dbxref=GeneID:123186923,Genbank:XP_044454592.1;Name=XP_044454592.1;gbkey=CDS;gene=LOC123186923;product=

Protein Rf1%2C mitochondrial-like.

Kozak. CCCATGC ID=cds-XP_044454778.1;Parent=rna-XM_044598843.1;Dbxref=GeneID:123187075,Genbank:XP_044454778.1;Name=XP_044454778.1;gbkey=CDS;gene=LOC123187075;product=pectin acetylesterase 5-like;protein_id=XP_044454778.1

GO:0005576; Extracellular region.

Kozak: CCCATGC ID=cds-XP_044456911.1;Parent=rna-XM_044600976.1;Dbxref=GeneID:123188715,Genbank:XP_044456911.1;Name=XP_044456911.1;Note=The sequence of the model RefSeq protein was modified relative to this genomic sequence to represent the inferred CDS: added 238 bases not found in genome assembly;exception=annotated by transcript or proteomic data;gbkey=CDS;gene=LOC123188715;inference=similar to RNA sequence (same species):INSD:GEWU01032847.1;partial=true;product=

Nascent polypeptide-associated complex subunit alpha%2C muscle-specific form-like.

Kozak: CCCATGT ID=cds-XP_044457665.1;Parent=rna-XM_044601730.1;Dbxref=GeneID:123189331,Genbank:XP_044457665.1;Name=XP_044457665.1;gbkey=CDS;gene=LOC123189331;product=

Protein Rf1%2C mitochondrial-like.

Kozak: CCAATGT ID=cds-XP_044457813.1;Parent=rna-XM_044601878.1;Dbxref=GeneID:123189429,Genbank:XP_044457813.1;Name=XP_044457813.1;gbkey=CDS;gene=LOC123189429;product=uncharacterized protein LOC123189429;protein_id=XP_044457813.1

GO:0016021; Integral component of membrane.

Kozak: TTCATGT ID=cds-XP_044459110.1;Parent=rna-XM_044603175.1;Dbxref=GeneID:123190518,Genbank:XP_044459110.1;Name=XP_044459110.1;gbkey=CDS;gene=LOC123190518;product=uncharacterized protein LOC123190518;protein_id=XP_044459110.1

GO:0016021; Integral component of membrane.

Kozak: CGAATGT ID=cds-XP_044348521.1;Parent=rna-XM_044492586.1;Dbxref=GeneID:100037637,Genbank:XP_044348521.1;Name=XP_044348521.1;gbkey=CDS;gene=LOC100037637;product=glucose-1-phosphate adenylyltransferase large subunit%2C chloroplastic/amyloplastic-like;protein_id=XP_044348521.1

GO:0009501; Amyloplast.

Kozak: CCCATGC ID=cds-XP_044318068.1;Parent=rna-XM_044462133.1;Dbxref=GeneID:123038257,Genbank:XP_044318068.1;Name=XP_044318068.1;gbkey=CDS;gene=LOC123038257;product=

Pentatricopeptide repeat-containing protein At1g11900-like.

Kozak: TCCATGT ID=cds-XP_044318101.1;Parent=rna-XM_044462166.1;Dbxref=GeneID:123038311,Genbank:XP_044318101.1;Name=XP_044318101.1;gbkey=CDS;gene=LOC123038311;product=

Pentatricopeptide repeat-containing protein At3g05340-like.

Kozak: TACATGC ID=cds-XP_044320016.1;Parent=rna-XM_044464081.1;Dbxref=GeneID:123041477,Genbank:XP_044320016.1;Name=XP_044320016.1;gbkey=CDS;gene=LOC123041477;product=

Putative protein TPRXL.

Kozak: CCCATGT ID=cds-XP_044325358.1;Parent=rna-XM_044469423.1;Dbxref=GeneID:123046133,Genbank:XP_044325358.1;Name=XP_044325358.1;gbkey=CDS;gene=LOC123046133;product=uncharacterized protein LOC123046133 isoform X1;protein_id=XP_044325358.1

GO:0016021; Integral component of membrane.

Kozak: TTCATGT ID=cds-XP_044327321.1;Parent=rna-XM_044471386.1;Dbxref=GeneID:123048238,Genbank:XP_044327321.1;Name=XP_044327321.1;gbkey=CDS;gene=LOC123048238;product=

SET and MYND domain-containing protein 4-like isoform X2.

Kozak: CCAATGC ID=cds-XP_044327452.1;Parent=rna-XM_044471517.1;Dbxref=GeneID:123048404,Genbank:XP_044327452.1;Name=XP_044327452.1;gbkey=CDS;gene=LOC123048404;product=electron transfer flavoprotein-ubiquinone oxidoreductase%2C mitochondrial-like isoform X2;protein_id=XP_044327452.1

GO:0031305; Integral component of mitochondrial inner membrane.

Kozak: CCCATGT ID=cds-XP_044329725.1;Parent=rna-XM_044473790.1;Dbxref=GeneID:123051040,Genbank:XP_044329725.1;Name=XP_044329725.1;gbkey=CDS;gene=LOC123051040;product=

SUMO-conjugating enzyme SCE1-like.

Kozak: CAGATGC ID=cds-XP_044331839.1;Parent=rna-XM_044475904.1;Dbxref=GeneID:123052626,Genbank:XP_044331839.1;Name=XP_044331839.1;gbkey=CDS;gene=LOC123052626;product=

Cytosolic endo-beta-N-acetylglucosaminidase 1-like.

Kozak: CCAATGT ID=cds-XP_044333361.1;Parent=rna-XM_044477426.1;Dbxref=GeneID:123053846,Genbank:XP_044333361.1;Name=XP_044333361.1;gbkey=CDS;gene=LOC123053846;product=pentatricopeptide repeat-containing protein At1g20230-like;protein_id=XP_044333361.1

GO:0008270; Zinc ion binding.

Kozak: TTCATGT ID=cds-XP_044333986.1;Parent=rna-XM_044478051.1;Dbxref=GeneID:123054313,Genbank:XP_044333986.1;Name=XP_044333986.1;gbkey=CDS;gene=LOC123054313;product=

Protein FAR1-RELATED SEQUENCE 5-like.

Kozak: CCCATGC ID=cds-XP_044334378.1;Parent=rna-XM_044478443.1;Dbxref=GeneID:123054635,Genbank:XP_044334378.1;Name=XP_044334378.1;gbkey=CDS;gene=LOC123054635;product=argininosuccinate synthase%2C chloroplastic-like;protein_id=XP_044334378.1

GO:0005737; Cytoplasm.

Kozak: CCCATGC ID=cds-XP_044336158.1;Parent=rna-XM_044480223.1;Dbxref=GeneID:123057053,Genbank:XP_044336158.1;Name=XP_044336158.1;gbkey=CDS;gene=LOC123057053;product=DAR GTPase 3%2C chloroplastic-like;protein_id=XP_044336158.1

GO:0005739; Mitochondrion.

Kozak: CAGATGC ID=cds-XP_044342448.1;Parent=rna-XM_044486513.1;Dbxref=GeneID:123062836,Genbank:XP_044342448.1;Name=XP_044342448.1;gbkey=CDS;gene=LOC123062836;product=beta-arabinofuranosyltransferase RAY1-like;protein_id=XP_044342448.1

GO:0016757; Glycosyltransferase activity.

Kozak: CCCATGC ID=cds-XP_044344655.1;Parent=rna-XM_044488720.1;Dbxref=GeneID:123065444,Genbank:XP_044344655.1;Name=XP_044344655.1;gbkey=CDS;gene=LOC123065444;product=uncharacterized protein LOC123065444;protein_id=XP_044344655.1

GO:0016021; Integral component of membrane.

Kozak: CCCATGC ID=cds-XP_044345643.1;Parent=rna-XM_044489708.1;Dbxref=GeneID:123066634,Genbank:XP_044345643.1;Name=XP_044345643.1;gbkey=CDS;gene=LOC123066634;product=G-type lectin S-receptor-like serine/threonine-protein kinase At2g19130;protein_id=XP_044345643.1

GO:0016021; Integral component of membrane.

Kozak: CCAATGC ID=cds-XP_044346360.1;Parent=rna-XM_044490425.1;Dbxref=GeneID:123067774,Genbank:XP_044346360.1;Name=XP_044346360.1;gbkey=CDS;gene=LOC123067774;product=auxin-responsive protein IAA19-like;protein_id=XP_044346360.1

GO:0005634; Nucleus.

Kozak: CCCATGC ID=cds-XP_044346912.1;Parent=rna-XM_044490977.1;Dbxref=GeneID:123068366,Genbank:XP_044346912.1;Name=XP_044346912.1;gbkey=CDS;gene=LOC123068366;product=protein trichome birefringence-like 8;protein_id=XP_044346912.1

GO:0005794; Golgi apparatus.

Kozak: TCCATGT ID=cds-XP_044348620.1;Parent=rna-XM_044492685.1;Dbxref=GeneID:123069739,Genbank:XP_044348620.1;Name=XP_044348620.1;gbkey=CDS;gene=LOC123069739;product=

WRKY transcription factor 71-like.

Kozak: CCAATGT ID=cds-XP_044351869.1;Parent=rna-XM_044495934.1;Dbxref=GeneID:123072338,Genbank:XP_044351869.1;Name=XP_044351869.1;gbkey=CDS;gene=LOC123072338;product=uncharacterized protein LOC123072338;protein_id=XP_044351869.1

GO:0004867; Serine-type endopeptidase inhibitor activity.

Kozak: CCAATGT ID=cds-XP_044352037.1;Parent=rna-XM_044496102.1;Dbxref=GeneID:123072504,Genbank:XP_044352037.1;Name=XP_044352037.1;gbkey=CDS;gene=LOC123072504;product=uncharacterized protein LOC123072504;protein_id=XP_044352037.1

GO:0004867; Serine-type endopeptidase inhibitor activity.

Kozak: CCAATGT ID=cds-XP_044352122.1;Parent=rna-XM_044496187.1;Dbxref=GeneID:123072579,Genbank:XP_044352122.1;Name=XP_044352122.1;gbkey=CDS;gene=LOC123072579;product=uncharacterized protein LOC123072579;protein_id=XP_044352122.1

GO:0004867; Serine-type endopeptidase inhibitor activity.

Kozak: TTCATGT ID=cds-XP_044383505.1;Parent=rna-XM_044527570.1;Dbxref=GeneID:123105466,Genbank:XP_044383505.1;Name=XP_044383505.1;gbkey=CDS;gene=LOC123105466;product=

LOB domain-containing protein 15-like.

Kozak: TCCATGT ID=cds-XP_044402643.1;Parent=rna-XM_044546708.1;Dbxref=GeneID:123126376,Genbank:XP_044402643.1;Name=XP_044402643.1;gbkey=CDS;gene=LOC123126376;product=protein DETOXIFICATION 49-like;protein_id=XP_044402643.1

GO:0016021; Integral component of membrane.

Kozak: CGAATGT ID=cds-XP_044422044.1;Parent=rna-XM_044566109.1;Dbxref=GeneID:123146743,Genbank:XP_044422044.1;Name=XP_044422044.1;gbkey=CDS;gene=LOC123146743;product=

Protein AUXIN-REGULATED GENE INVOLVED IN ORGAN SIZE-like.

Kozak: CAGATGC ID=cds-XP_044447248.1;Parent=rna-XM_044591313.1;Dbxref=GeneID:123177745,Genbank:XP_044447248.1;Name=XP_044447248.1;gbkey=CDS;gene=LOC123177745;product=uncharacterized protein LOC123177745;protein_id=XP_044447248.1

GO:0005681; Spliceosomal complex.

Kozak: TACATGC ID=cds-XP_044449189.1;Parent=rna-XM_044593254.1;Dbxref=GeneID:123181034,Genbank:XP_044449189.1;Name=XP_044449189.1;gbkey=CDS;gene=LOC123181034;product=probable purine permease 4;protein_id=XP_044449189.1

GO:0016021; Integral component of membrane.

Kozak: TCCATGT ID=cds-XP_044451070.1;Parent=rna-XM_044595135.1;Dbxref=GeneID:123182528,Genbank:XP_044451070.1;Name=XP_044451070.1;gbkey=CDS;gene=LOC123182528;product=WRKY transcription factor 23-like;protein_id=XP_044451070.1

GO:0005634; Nucleus.

Kozak: CCAATGC ID=cds-XP_044458395.1;Parent=rna-XM_044602460.1;Dbxref=GeneID:123189934,Genbank:XP_044458395.1;Name=XP_044458395.1;gbkey=CDS;gene=LOC123189934;product=

Protein AGENET DOMAIN (AGD)-CONTAINING P1-like.

Kozak: TCCATGT ID=cds-XP_044367162.1;Parent=rna-XM_044511227.1;Dbxref=GeneID:123089589,Genbank:XP_044367162.1;Name=XP_044367162.1;gbkey=CDS;gene=LOC123089589;product=

Acetylserotonin O-methyltransferase 3-like.

Kozak: TCCATGT ID=cds-XP_044367202.1;Parent=rna-XM_044511267.1;Dbxref=GeneID:123089636,Genbank:XP_044367202.1;Name=XP_044367202.1;gbkey=CDS;gene=LOC123089636;product=protein TIFY 6a-like;protein_id=XP_044367202.1

GO:0005634; Nucleus.

Kozak: CAGATGC ID=cds-XP_044367228.1;Parent=rna-XM_044511293.1;Dbxref=GeneID:123089676,Genbank:XP_044367228.1;Name=XP_044367228.1;gbkey=CDS;gene=LOC123089676;product=flavin-containing monooxygenase FMO GS-OX-like 3;protein_id=XP_044367228.1

GO:0050660; Flavin adenine dinucleotide binding.

Kozak: CCAATGT ID=cds-XP_044367261.1;Parent=rna-XM_044511326.1;Dbxref=GeneID:123089735,Genbank:XP_044367261.1;Name=XP_044367261.1;gbkey=CDS;gene=LOC123089735;product=uncharacterized protein LOC123089735;protein_id=XP_044367261.1

GO:0005634; Nucleus.

Kozak: CAGATGC ID=cds-XP_044367462.1;Parent=rna-XM_044511527.1;Dbxref=GeneID:123090038,Genbank:XP_044367462.1;Name=XP_044367462.1;gbkey=CDS;gene=LOC123090038;product=

MADS-box transcription factor 58-like.

Kozak: TTCATGT ID=cds-XP_044367519.1;Parent=rna-XM_044511584.1;Dbxref=GeneID:123090159,Genbank:XP_044367519.1;Name=XP_044367519.1;gbkey=CDS;gene=LOC123090159;product=serine/arginine-rich splicing factor SR45a-like;protein_id=XP_044367519.1

GO:0003723; RNA binding.

Kozak: CCCATGC ID=cds-XP_044367594.1;Parent=rna-XM_044511659.1;Dbxref=GeneID:123090282,Genbank:XP_044367594.1;Name=XP_044367594.1;gbkey=CDS;gene=LOC123090282;product=molybdate transporter 1-like;protein_id=XP_044367594.1

GO:0016021; Integral component of membrane.

Kozak: CCAATGT ID=cds-XP_044367653.1;Parent=rna-XM_044511718.1;Dbxref=GeneID:123090398,Genbank:XP_044367653.1;Name=XP_044367653.1;gbkey=CDS;gene=LOC123090398;product=E3 ubiquitin-protein ligase RFWD3-like;protein_id=XP_044367653.1

GO:0005634; Nucleus.

Kozak: CCCATGT ID=cds-XP_044368162.1;Parent=rna-XM_044512227.1;Dbxref=GeneID:123090873,Genbank:XP_044368162.1;Name=XP_044368162.1;gbkey=CDS;gene=LOC123090873;product=protein POLLEN DEFECTIVE IN GUIDANCE 1-like;protein_id=XP_044368162.1

GO:0030176; Integral component of endoplasmic reticulum membrane.

Kozak: CAGATGC ID=cds-XP_044368561.1;Parent=rna-XM_044512626.1;Dbxref=GeneID:123091194,Genbank:XP_044368561.1;Name=XP_044368561.1;gbkey=CDS;gene=LOC123091194;product=pentatricopeptide repeat-containing protein At3g12770-like;protein_id=XP_044368561.1

GO:0003723; RNA binding.

Kozak: CCCATGT ID=cds-XP_044368667.1;Parent=rna-XM_044512732.1;Dbxref=GeneID:123091272,Genbank:XP_044368667.1;Name=XP_044368667.1;gbkey=CDS;gene=LOC123091272;product=

Putative B3 domain-containing protein Os04g0346900.

Kozak: CCCATGT ID=cds-XP_044368813.1;Parent=rna-XM_044512878.1;Dbxref=GeneID:123091382,Genbank:XP_044368813.1;Name=XP_044368813.1;gbkey=CDS;gene=LOC123091382;product=protein transport protein Sec24-like CEF;protein_id=XP_044368813.1

GO:0030127; COPII vesicle coat.

Kozak: CCCATGT ID=cds-XP_044368848.1;Parent=rna-XM_044512913.1;Dbxref=GeneID:123091409,Genbank:XP_044368848.1;Name=XP_044368848.1;gbkey=CDS;gene=LOC123091409;product=probable LRR receptor-like serine/threonine-protein kinase RKF3;protein_id=XP_044368848.1

GO:0016021; Integral component of membrane.

Kozak: CCAATGC ID=cds-XP_044368849.1;Parent=rna-XM_044512914.1;Dbxref=GeneID:123091413,Genbank:XP_044368849.1;Name=XP_044368849.1;gbkey=CDS;gene=LOC123091413;product=protein SHORTAGE IN CHIASMATA 1 homolog;protein_id=XP_044368849.1

GO:0000712; Resolution of meiotic recombination intermediates.

Kozak: CCAATGC ID=cds-XP_044368855.1;Parent=rna-XM_044512920.1;Dbxref=GeneID:123091417,Genbank:XP_044368855.1;Name=XP_044368855.1;gbkey=CDS;gene=LOC123091417;product=calcium-dependent protein kinase 24-like;protein_id=XP_044368855.1

GO:0005737; Cytoplasm.

Kozak: CAGATGC ID=cds-XP_044369355.1;Parent=rna-XM_044513420.1;Dbxref=GeneID:123091812,Genbank:XP_044369355.1;Name=XP_044369355.1;gbkey=CDS;gene=LOC123091812;product=armadillo repeat-containing protein 8-like isoform X1;protein_id=XP_044369355.1

GO:0005737; Cytoplasm.

Kozak: CCAATGC ID=cds-XP_044369624.1;Parent=rna-XM_044513689.1;Dbxref=GeneID:123092036,Genbank:XP_044369624.1;Name=XP_044369624.1;gbkey=CDS;gene=LOC123092036;product=phosphatidylinositol 4-phosphate 5-kinase 6-like isoform X1;protein_id=XP_044369624.1

GO:0005886; Plasma membrane.

Kozak: CCAATGC ID=cds-XP_044369704.1;Parent=rna-XM_044513769.1;Dbxref=GeneID:123092087,Genbank:XP_044369704.1;Name=XP_044369704.1;gbkey=CDS;gene=LOC123092087;product=O-fucosyltransferase 1-like;protein_id=XP_044369704.1

GO:0005737; Cytoplasm.

Kozak: CGAATGT ID=cds-XP_044369840.1;Parent=rna-XM_044513905.1;Dbxref=GeneID:123092203,Genbank:XP_044369840.1;Name=XP_044369840.1;gbkey=CDS;gene=LOC123092203;product=

Macro domain-containing protein VPA0103-like.

Kozak: TCCATGT ID=cds-XP_044370015.1;Parent=rna-XM_044514080.1;Dbxref=GeneID:123092332,Genbank:XP_044370015.1;Name=XP_044370015.1;gbkey=CDS;gene=LOC123092332;product=DEAD-box ATP-dependent RNA helicase 24-like;protein_id=XP_044370015.1

GO:0005634; Nucleus.

Kozak: CGAATGT ID=cds-XP_044370140.1;Parent=rna-XM_044514205.1;Dbxref=GeneID:123092428,Genbank:XP_044370140.1;Name=XP_044370140.1;gbkey=CDS;gene=LOC123092428;product=E3 ubiquitin-protein ligase BIG BROTHER-like;protein_id=XP_044370140.1

GO:0031624; Ubiquitin conjugating enzyme binding.

Kozak: CCAATGC ID=cds-XP_044370474.1;Parent=rna-XM_044514539.1;Dbxref=GeneID:123092717,Genbank:XP_044370474.1;Name=XP_044370474.1;gbkey=CDS;gene=LOC123092717;product=imidazole glycerol phosphate synthase hisHF%2C chloroplastic-like;protein_id=XP_044370474.1

GO:0009507; Chloroplast.

Kozak: CAGATGC ID=cds-XP_044370700.1;Parent=rna-XM_044514765.1;Dbxref=GeneID:123092910,Genbank:XP_044370700.1;Name=XP_044370700.1;Note=The sequence of the model RefSeq protein was modified relative to this genomic sequence to represent the inferred CDS: added 54 bases not found in genome assembly;exception=annotated by transcript or proteomic data;gbkey=CDS;gene=LOC123092910;inference=similar to RNA sequence (same species):INSD:GIJS01123763.1;partial=true;product=

Pentatricopeptide repeat-containing protein At5g47360-like.

Kozak: CCCATGC ID=cds-XP_044371204.1;Parent=rna-XM_044515269.1;Dbxref=GeneID:123093333,Genbank:XP_044371204.1;Name=XP_044371204.1;gbkey=CDS;gene=LOC123093333;product=tRNA-specific 2-thiouridylase MnmA-like;protein_id=XP_044371204.1

GO:0005524; ATP binding.

Kozak: CCCATGC ID=cds-XP_044371393.1;Parent=rna-XM_044515458.1;Dbxref=GeneID:123093481,Genbank:XP_044371393.1;Name=XP_044371393.1;gbkey=CDS;gene=LOC123093481;product=beta-glucuronosyltransferase GlcAT14A-like;protein_id=XP_044371393.1

GO:0016020; Membrane.

Kozak: CCCATGC ID=cds-XP_044371922.1;Parent=rna-XM_044515987.1;Dbxref=GeneID:123093925,Genbank:XP_044371922.1;Name=XP_044371922.1;gbkey=CDS;gene=LOC123093925;product=palmitoyl-protein thioesterase 1-like isoform X1;protein_id=XP_044371922.1

GO:0043231; Intracellular membrane-bounded organelle.

Kozak: CCAATGT ID=cds-XP_044372582.1;Parent=rna-XM_044516647.1;Dbxref=GeneID:123094710,Genbank:XP_044372582.1;Name=XP_044372582.1;gbkey=CDS;gene=LOC123094710;product=protein DMP6-like;protein_id=XP_044372582.1

GO:0016021; Integral component of membrane.

Kozak: TACATGC ID=cds-XP_044372658.1;Parent=rna-XM_044516723.1;Dbxref=GeneID:123094839,Genbank:XP_044372658.1;Name=XP_044372658.1;gbkey=CDS;gene=LOC123094839;product=60S ribosomal protein L36-2-like;protein_id=XP_044372658.1

GO:0022625; Cytosolic large ribosomal subunit.

Kozak: CCAATGT ID=cds-XP_044372736.1;Parent=rna-XM_044516801.1;Dbxref=GeneID:123094943,Genbank:XP_044372736.1;Name=XP_044372736.1;gbkey=CDS;gene=LOC123094943;product=probable membrane-associated kinase regulator 4;protein_id=XP_044372736.1

GO:0005886; Plasma membrane.

Kozak: CCCATGT ID=cds-XP_044372878.1;Parent=rna-XM_044516943.1;Dbxref=GeneID:123095124,Genbank:XP_044372878.1;Name=XP_044372878.1;gbkey=CDS;gene=LOC123095124;product=F-box protein SNE-like;protein_id=XP_044372878.1

GO:0019005;SCF ubiquitin ligase complex.

Kozak: TCCATGT ID=cds-XP_044366896.1;Parent=rna-XM_044510961.1;Dbxref=GeneID:123089237,Genbank:XP_044366896.1;Name=XP_044366896.1;gbkey=CDS;gene=LOC123089237;product=

Putative MO25-like protein At5g47540 isoform X1.

Kozak: CCCATGC ID=cds-XP_044367001.1;Parent=rna-XM_044511066.1;Dbxref=GeneID:123089368,Genbank:XP_044367001.1;Name=XP_044367001.1;gbkey=CDS;gene=LOC123089368;product=WAT1-related protein At1g43650-like;protein_id=XP_044367001.1

GO:0016021; Integral component of membrane.

Kozak: CCCATGC ID=cds-XP_044367268.1;Parent=rna-XM_044511333.1;Dbxref=GeneID:123089744,Genbank:XP_044367268.1;Name=XP_044367268.1;gbkey=CDS;gene=LOC123089744;product=

Nucleolin-like.

Kozak: TCCATGT ID=cds-XP_044367382.1;Parent=rna-XM_044511447.1;Dbxref=GeneID:123089896,Genbank:XP_044367382.1;Name=XP_044367382.1;gbkey=CDS;gene=LOC123089896;product=protein argonaute 12-like;protein_id=XP_044367382.1

GO:0005737; Cytoplasm.

Kozak: CCAATGC ID=cds-XP_044367666.1;Parent=rna-XM_044511731.1;Dbxref=GeneID:123090414,Genbank:XP_044367666.1;Name=XP_044367666.1;gbkey=CDS;gene=LOC123090414;product=

Kinase-interacting family protein-like.

Kozak: TACATGC ID=cds-XP_044367981.1;Parent=rna-XM_044512046.1;Dbxref=GeneID:123090713,Genbank:XP_044367981.1;Name=XP_044367981.1;gbkey=CDS;gene=LOC123090713;product=

Protein FLOURY ENDOSPERM 6%2C chloroplastic-like.

Kozak: CCCATGT ID=cds-XP_044367987.1;Parent=rna-XM_044512052.1;Dbxref=GeneID:123090717,Genbank:XP_044367987.1;Name=XP_044367987.1;gbkey=CDS;gene=LOC123090717;product=B3 domain-containing protein Os12g0591400-like;protein_id=XP_044367987.1

GO:0005634; Nucleus.

Kozak: TTAATGC ID=cds-XP_044368003.1;Parent=rna-XM_044512068.1;Dbxref=GeneID:123090734,Genbank:XP_044368003.1;Name=XP_044368003.1;gbkey=CDS;gene=LOC123090734;product=protein SOSEKI 3-like isoform X1;protein_id=XP_044368003.1

GO:0031234; Extrinsic component of cytoplasmic side of plasma membrane.

Kozak: CCCATGC ID=cds-XP_044368344.1;Parent=rna-XM_044512409.1;Dbxref=GeneID:123091021,Genbank:XP_044368344.1;Name=XP_044368344.1;gbkey=CDS;gene=LOC123091021;product=

Atherin-like.

Kozak. CCCATGC ID=cds-XP_044368383.1;Parent=rna-XM_044512448.1;Dbxref=GeneID:123091054,Genbank:XP_044368383.1;Name=XP_044368383.1;gbkey=CDS;gene=LOC123091054;product=calmodulin calcium-dependent NAD kinase-like;protein_id=XP_044368383.1

GO:0005524; ATP binding.

Kozak: CAGATGC ID=cds-XP_044368413.1;Parent=rna-XM_044512478.1;Dbxref=GeneID:123091080,Genbank:XP_044368413.1;Name=XP_044368413.1;gbkey=CDS;gene=LOC123091080;product=B3 domain-containing protein Os03g0622200-like;protein_id=XP_044368413.1

GO:0005634; Nucleus.

Kozak: TGTATGT ID=cds-XP_044369306.1;Parent=rna-XM_044513371.1;Dbxref=GeneID:123091754,Genbank:XP_044369306.1;Name=XP_044369306.1;gbkey=CDS;gene=LOC123091754;product=alanine--tRNA ligase-like;protein_id=XP_044369306.1

GO:0009507; Chloroplast.

Kozak: CCAATGC ID=cds-XP_044369842.1;Parent=rna-XM_044513907.1;Dbxref=GeneID:123092205,Genbank:XP_044369842.1;Name=XP_044369842.1;gbkey=CDS;gene=LOC123092205;product=transcription factor RF2b-like;protein_id=XP_044369842.1

GO:0005634; Nucleus.

Kozak: CCAATGT ID=cds-XP_044369929.1;Parent=rna-XM_044513994.1;Dbxref=GeneID:123092275,Genbank:XP_044369929.1;Name=XP_044369929.1;gbkey=CDS;gene=LOC123092275;product=tRNA-specific adenosine deaminase TAD2-like isoform X1;protein_id=XP_044369929.1

GO:0052717; tRNA-specific adenosine-34 deaminase activity.

Kozak: CCCATGT ID=cds-XP_044370019.1;Parent=rna-XM_044514084.1;Dbxref=GeneID:123092336,Genbank:XP_044370019.1;Name=XP_044370019.1;gbkey=CDS;gene=LOC123092336;product=FIP1[III]-like protein isoform X1;protein_id=XP_044370019.1

GO:0005634; Nucleus.

Kozak. TCCATGT ID=cds-XP_044370184.1;Parent=rna-XM_044514249.1;Dbxref=GeneID:123092467,Genbank:XP_044370184.1;Name=XP_044370184.1;gbkey=CDS;gene=LOC123092467;product=rhodanese-like domain-containing protein 10;protein_id=XP_044370184.1

GO:0016021; Integral component of membrane.

Kozak: CGAATGT ID=cds-XP_044370626.1;Parent=rna-XM_044514691.1;Dbxref=GeneID:123092842,Genbank:XP_044370626.1;Name=XP_044370626.1;gbkey=CDS;gene=LOC123092842;product=anaphase-promoting complex subunit 6-like;protein_id=XP_044370626.1

GO:0005680; Anaphase-promoting complex.

Kozak: CCCATGT ID=cds-XP_044370722.1;Parent=rna-XM_044514787.1;Dbxref=GeneID:123092931,Genbank:XP_044370722.1;Name=XP_044370722.1;gbkey=CDS;gene=LOC123092931;product=

Peroxygenase-like.

Kozak: CCAATGT ID=cds-XP_044370824.1;Parent=rna-XM_044514889.1;Dbxref=GeneID:123093013,Genbank:XP_044370824.1;Name=XP_044370824.1;gbkey=CDS;gene=LOC123093013;product=glucan endo-1%2C3-beta-glucosidase 1-like;protein_id=XP_044370824.1

GO:0046658; Anchored component of plasma membrane.

Kozak: CCCATGT ID=cds-XP_044371202.1;Parent=rna-XM_044515267.1;Dbxref=GeneID:123093331,Genbank:XP_044371202.1;Name=XP_044371202.1;gbkey=CDS;gene=LOC123093331;product=adenylosuccinate synthetase%2C chloroplastic-like;protein_id=XP_044371202.1

GO:0009507; Chloroplast.

Kozak: CCCATGT ID=cds-XP_044371410.1;Parent=rna-XM_044515475.1;Dbxref=GeneID:123093496,Genbank:XP_044371410.1;Name=XP_044371410.1;gbkey=CDS;gene=LOC123093496;product=pentatricopeptide repeat-containing protein At1g76280-like;protein_id=XP_044371410.1

GO:0008663; 2',3'-cyclic-nucleotide 2'-phosphodiesterase activity.

Kozak: CCCATGC ID=cds-XP_044371447.1;Parent=rna-XM_044515512.1;Dbxref=GeneID:123093529,Genbank:XP_044371447.1;Name=XP_044371447.1;gbkey=CDS;gene=LOC123093529;product=

Protein GDAP2 homolog.

Kozak: TCCATGT ID=cds-XP_044371579.1;Parent=rna-XM_044515644.1;Dbxref=GeneID:123093638,Genbank:XP_044371579.1;Name=XP_044371579.1;gbkey=CDS;gene=LOC123093638;product=probable serine acetyltransferase 2;protein_id=XP_044371579.1

GO:0005737; Cytoplasm.

Kozak: CCAATGC ID=cds-XP_044371728.1;Parent=rna-XM_044515793.1;Dbxref=GeneID:123093748,Genbank:XP_044371728.1;Name=XP_044371728.1;gbkey=CDS;gene=LOC123093748;product=ABC transporter C family member 13-like;protein_id=XP_044371728.1

GO:0000139; Golgi membrane.

Kozak: CCCATGT ID=cds-XP_044371789.1;Parent=rna-XM_044515854.1;Dbxref=GeneID:123093800,Genbank:XP_044371789.1;Name=XP_044371789.1;gbkey=CDS;gene=LOC123093800;product=

Pentatricopeptide repeat-containing protein At4g20090-like.

Kozak: CAGATGC ID=cds-XP_044371885.1;Parent=rna-XM_044515950.1;Dbxref=GeneID:123093886,Genbank:XP_044371885.1;Name=XP_044371885.1;gbkey=CDS;gene=LOC123093886;product=

Pentatricopeptide repeat-containing protein At1g03100%2C mitochondrial-like.

Kozak: CCAATGC ID=cds-XP_044371979.1;Parent=rna-XM_044516044.1;Dbxref=GeneID:123093981,Genbank:XP_044371979.1;Name=XP_044371979.1;gbkey=CDS;gene=LOC123093981;product=uncharacterized protein LOC123093981;protein_id=XP_044371979.1

GO:0003755; Peptidyl-prolyl cis-trans isomerase activity.

Kozak: CCCATGC ID=cds-XP_044372053.1;Parent=rna-XM_044516118.1;Dbxref=GeneID:123094040,Genbank:XP_044372053.1;Name=XP_044372053.1;gbkey=CDS;gene=LOC123094040;product=folylpolyglutamate synthase-like isoform X1;protein_id=XP_044372053.1

GO:0005737; Cytoplasm

Kozak: TCCATGT ID=cds-XP_044372061.1;Parent=rna-XM_044516126.1;Dbxref=GeneID:123094046,Genbank:XP_044372061.1;Name=XP_044372061.1;gbkey=CDS;gene=LOC123094046;product=

CCR4-NOT transcription complex subunit 9-like isoform X1.

Kozak: TGTATGT ID=cds-XP_044372097.1;Parent=rna-XM_044516162.1;Dbxref=GeneID:123094083,Genbank:XP_044372097.1;Name=XP_044372097.1;gbkey=CDS;gene=LOC123094083;product=alanine--tRNA ligase-like;protein_id=XP_044372097.1

GO:0009507; Chloroplast.

Kozak: CAGATGC ID=cds-XP_044372536.1;Parent=rna-XM_044516601.1;Dbxref=GeneID:123094639,Genbank:XP_044372536.1;Name=XP_044372536.1;gbkey=CDS;gene=LOC123094639;product=epsin-1-like;protein_id=XP_044372536.1

GO:0005634; Nucleus.

Kozak: CCAATGT ID=cds-XP_044372621.1;Parent=rna-XM_044516686.1;Dbxref=GeneID:123094774,Genbank:XP_044372621.1;Name=XP_044372621.1;gbkey=CDS;gene=LOC123094774;product=

Pre-mRNA-splicing factor CWC22-like.

Kozak: TACATGC ID=cds-XP_044372707.1;Parent=rna-XM_044516772.1;Dbxref=GeneID:123094902,Genbank:XP_044372707.1;Name=XP_044372707.1;gbkey=CDS;gene=LOC123094902;product=sex determination protein tasselseed-2-like;protein_id=XP_044372707.1

GO:0005938; Cell cortex.

Kozak: CCCATGT ID=cds-XP_044372718.1;Parent=rna-XM_044516783.1;Dbxref=GeneID:123094913,Genbank:XP_044372718.1;Name=XP_044372718.1;gbkey=CDS;gene=LOC123094913;product=uncharacterized protein LOC123094913;protein_id=XP_044372718.1

GO:0005634; Nucleus

Kozak: TCCATGT ID=cds-XP_044372941.1;Parent=rna-XM_044517006.1;Dbxref=GeneID:123095197,Genbank:XP_044372941.1;Name=XP_044372941.1;gbkey=CDS;gene=LOC123095197;product=

Late embryogenesis abundant protein D-34-like.

Kozak: TTCATGT ID=cds-XP_044420013.1;Parent=rna-XM_044564078.1;Dbxref=GeneID:100146082,Genbank:XP_044420013.1;Name=XP_044420013.1;gbkey=CDS;gene=LOC100146082;product=putative receptor-like protein kinase At3g47110 isoform X1;protein_id=XP_044420013.1

GO:0016021; Integral component of membrane.

Kozak: CCCATGC ID=cds-XP_044416096.1;Parent=rna-XM_044560161.1;Dbxref=GeneID:123140898,Genbank:XP_044416096.1;Name=XP_044416096.1;gbkey=CDS;gene=LOC123140898;product=E3 ubiquitin-protein ligase Os04g0590900-like;protein_id=XP_044416096.1

GO:0016021; Integral component of membrane.

Kozak: CCCATGT ID=cds-XP_044416177.1;Parent=rna-XM_044560242.1;Dbxref=GeneID:123140988,Genbank:XP_044416177.1;Name=XP_044416177.1;gbkey=CDS;gene=LOC123140988;product=beta-fructofuranosidase%2C insoluble isoenzyme 4-like;protein_id=XP_044416177.1

GO:0004553; Hydrolase activity, hydrolyzing O-glycosyl compounds.

Kozak: CCCATGC ID=cds-XP_044416257.1;Parent=rna-XM_044560322.1;Dbxref=GeneID:123141090,Genbank:XP_044416257.1;Name=XP_044416257.1;gbkey=CDS;gene=LOC123141090;product=

Pentatricopeptide repeat-containing protein At1g60770-like.

Kozak: CCCATGC ID=cds-XP_044416260.1;Parent=rna-XM_044560325.1;Dbxref=GeneID:123141094,Genbank:XP_044416260.1;Name=XP_044416260.1;gbkey=CDS;gene=LOC123141094;product=pentatricopeptide repeat-containing protein At1g60770-like;protein_id=XP_044416260.1

GO:0005739; Mitochondrion.

Kozak: TACATGC ID=cds-XP_044416425.1;Parent=rna-XM_044560490.1;Dbxref=GeneID:123141313,Genbank:XP_044416425.1;Name=XP_044416425.1;gbkey=CDS;gene=LOC123141313;product=

Protease Do-like 7.

Kozak: CCAATGT ID=cds-XP_044416657.1;Parent=rna-XM_044560722.1;Dbxref=GeneID:123141629,Genbank:XP_044416657.1;Name=XP_044416657.1;gbkey=CDS;gene=LOC123141629;product=receptor-like protein 52;protein_id=XP_044416657.1

GO:0016021; Integral component of membrane.

Kozak: TACATGC ID=cds-XP_044416770.1;Parent=rna-XM_044560835.1;Dbxref=GeneID:123141759,Genbank:XP_044416770.1;Name=XP_044416770.1;gbkey=CDS;gene=LOC123141759;product=protein TIFY 6b-like;protein_id=XP_044416770.1

GO:0005634; Nucleus.

Kozak: CCCATGT ID=cds-XP_044416999.1;Parent=rna-XM_044561064.1;Dbxref=GeneID:123142030,Genbank:XP_044416999.1;Name=XP_044416999.1;gbkey=CDS;gene=LOC123142030;product=

F-box/kelch-repeat protein At1g57790-like.

Kozak: CCCATGT ID=cds-XP_044417333.1;Parent=rna-XM_044561398.1;Dbxref=GeneID:123142510,Genbank:XP_044417333.1;Name=XP_044417333.1;gbkey=CDS;gene=LOC123142510;product=

Protein indeterminate-domain 14-like.

Kozak: CCCATGT ID=cds-XP_044417419.1;Parent=rna-XM_044561484.1;Dbxref=GeneID:123142684,Genbank:XP_044417419.1;Name=XP_044417419.1;gbkey=CDS;gene=LOC123142684;product=

3-ketoacyl-CoA synthase 5-like.

Kozak: CCCATGC ID=cds-XP_044417588.1;Parent=rna-XM_044561653.1;Dbxref=GeneID:123142936,Genbank:XP_044417588.1;Name=XP_044417588.1;gbkey=CDS;gene=LOC123142936;product=WAT1-related protein At3g30340-like isoform X2;protein_id=XP_044417588.1

GO:0016021; Integral component of membrane.

Kozak: CCCATGC ID=cds-XP_044418039.1;Parent=rna-XM_044562104.1;Dbxref=GeneID:123143249,Genbank:XP_044418039.1;Name=XP_044418039.1;gbkey=CDS;gene=LOC123143249;product=peptidyl-prolyl cis-trans isomerase FKBP18%2C chloroplastic-like;protein_id=XP_044418039.1

GO:0003755; Peptidyl-prolyl cis-trans isomerase activity.

Kozak: CCCATGC ID=cds-XP_044418296.1;Parent=rna-XM_044562361.1;Dbxref=GeneID:123143431,Genbank:XP_044418296.1;Name=XP_044418296.1;gbkey=CDS;gene=LOC123143431;product=

3-ketoacyl-CoA synthase 20-like.

Kozak: CCCATGC ID=cds-XP_044418430.1;Parent=rna-XM_044562495.1;Dbxref=GeneID:123143548,Genbank:XP_044418430.1;Name=XP_044418430.1;gbkey=CDS;gene=LOC123143548;product=ribose-phosphate pyrophosphokinase 1%2C chloroplastic-like;protein_id=XP_044418430.1

GO:0005737; C:ytoplasm.

Kozak: CCCATGT ID=cds-XP_044418451.1;Parent=rna-XM_044562516.1;Dbxref=GeneID:123143572,Genbank:XP_044418451.1;Name=XP_044418451.1;gbkey=CDS;gene=LOC123143572;product=uncharacterized protein LOC123143572;protein_id=XP_044418451.1

GO:0005739; Mitochondrion.

Kozak: CCCATGT ID=cds-XP_044418484.1;Parent=rna-XM_044562549.1;Dbxref=GeneID:123143614,Genbank:XP_044418484.1;Name=XP_044418484.1;gbkey=CDS;gene=LOC123143614;product=

Phosphatidylinositol/phosphatidylcholine transfer protein SFH6-like isoform X1.

Kozak: CCAATGT ID=cds-XP_044418734.1;Parent=rna-XM_044562799.1;Dbxref=GeneID:123143804,Genbank:XP_044418734.1;Name=XP_044418734.1;gbkey=CDS;gene=LOC123143804;product=serrate RNA effector molecule-like isoform X1;protein_id=XP_044418734.1

GO:0016604; Nuclear body.

Kozak: CAGATGC ID=cds-XP_044418742.1;Parent=rna-XM_044562807.1;Dbxref=GeneID:123143811,Genbank:XP_044418742.1;Name=XP_044418742.1;gbkey=CDS;gene=LOC123143811;product=polyubiquitin-like;protein_id=XP_044418742.1

GO:0005737; Cytoplasm.

Kozak: CCCATGC ID=cds-XP_044419032.1;Parent=rna-XM_044563097.1;Dbxref=GeneID:123144083,Genbank:XP_044419032.1;Name=XP_044419032.1;gbkey=CDS;gene=LOC123144083;product=DNA-directed RNA polymerase III subunit 2-like;protein_id=XP_044419032.1

GO:0005666; RNA polymerase III complex.

Kozak: CCCATGC ID=cds-XP_044419128.1;Parent=rna-XM_044563193.1;Dbxref=GeneID:123144150,Genbank:XP_044419128.1;Name=XP_044419128.1;gbkey=CDS;gene=LOC123144150;product=

COBRA-like protein 7.

Kozak: CAGATGC ID=cds-XP_044419184.1;Parent=rna-XM_044563249.1;Dbxref=GeneID:123144197,Genbank:XP_044419184.1;Name=XP_044419184.1;gbkey=CDS;gene=LOC123144197;product=

Serine/threonine-protein kinase Nek8-like.

Kozak: TCCATGT ID=cds-XP_044419538.1;Parent=rna-XM_044563603.1;Dbxref=GeneID:123144453,Genbank:XP_044419538.1;Name=XP_044419538.1;gbkey=CDS;gene=LOC123144453;product=kinesin-like protein KIN-14D isoform X1;protein_id=XP_044419538.1

GO:0005874; Microtubule.

Kozak: CAGATGC ID=cds-XP_044419587.1;Parent=rna-XM_044563652.1;Dbxref=GeneID:123144489,Genbank:XP_044419587.1;Name=XP_044419587.1;gbkey=CDS;gene=LOC123144489;product=protein CbxX%2C chromosomal-like isoform X1;protein_id=XP_044419587.1

GO:0005524; ATP binding.

Kozak: TCCATGT ID=cds-XP_044419723.1;Parent=rna-XM_044563788.1;Dbxref=GeneID:123144583,Genbank:XP_044419723.1;Name=XP_044419723.1;gbkey=CDS;gene=LOC123144583;product=prohibitin-3%2C mitochondrial-like;protein_id=XP_044419723.1

GO:0005743; Mitochondrial inner membrane.

Kozak: TCCATGT ID=cds-XP_044419951.1;Parent=rna-XM_044564016.1;Dbxref=GeneID:123144779,Genbank:XP_044419951.1;Name=XP_044419951.1;gbkey=CDS;gene=LOC123144779;product=acetolactate synthase small subunit 2%2C chloroplastic-like;protein_id=XP_044419951.1

GO:0005737; Cytoplasm.

Kozak: CCCATGC ID=cds-XP_044420160.1;Parent=rna-XM_044564225.1;Dbxref=GeneID:123144951,Genbank:XP_044420160.1;Name=XP_044420160.1;gbkey=CDS;gene=LOC123144951;product=plasmodesmata-located protein 8-like;protein_id=XP_044420160.1

GO:0016021; Integral component of membrane.

Kozak: CCCATGC ID=cds-XP_044420589.1;Parent=rna-XM_044564654.1;Dbxref=GeneID:123145281,Genbank:XP_044420589.1;Name=XP_044420589.1;gbkey=CDS;gene=LOC123145281;product=ribose-phosphate pyrophosphokinase 4;protein_id=XP_044420589.1

GO:0005737; Cytoplasm.

Kozak: CCCATGT ID=cds-XP_044420911.1;Parent=rna-XM_044564976.1;Dbxref=GeneID:123145545,Genbank:XP_044420911.1;Name=XP_044420911.1;gbkey=CDS;gene=LOC123145545;product=dynamin-2B-like;protein_id=XP_044420911.1

GO:0005874; Microtubule.

Kozak: CCCATGC ID=cds-XP_044421034.1;Parent=rna-XM_044565099.1;Dbxref=GeneID:123145635,Genbank:XP_044421034.1;Name=XP_044421034.1;gbkey=CDS;gene=LOC123145635;product=putative D-cysteine desulfhydrase 1%2C mitochondrial;protein_id=XP_044421034.1

GO:0019148; D-cysteine desulfhydrase activity.

Kozak: CCAATGC ID=cds-XP_044421137.1;Parent=rna-XM_044565202.1;Dbxref=GeneID:123145730,Genbank:XP_044421137.1;Name=XP_044421137.1;gbkey=CDS;gene=LOC123145730;product=LRR receptor-like serine/threonine-protein kinase ER2;protein_id=XP_044421137.1

GO:0016021; Integral component of membrane.

Kozak: CCCATGC ID=cds-XP_044421356.1;Parent=rna-XM_044565421.1;Dbxref=GeneID:123145907,Genbank:XP_044421356.1;Name=XP_044421356.1;gbkey=CDS;gene=LOC123145907;product=transcription factor LRL3-like;protein_id=XP_044421356.1

GO:0005634; Nucleus.

Kozak: CCCATGC ID=cds-XP_044421697.1;Parent=rna-XM_044565762.1;Dbxref=GeneID:123146173,Genbank:XP_044421697.1;Name=XP_044421697.1;gbkey=CDS;gene=LOC123146173;product=protein LUTEIN DEFICIENT 5%2C chloroplastic-like;protein_id=XP_044421697.1

GO:0016021; Integral component of membrane.

Kozak: TCCATGT ID=cds-XP_044419825.1;Parent=rna-XM_044563890.1;Dbxref=GeneID:543106,Genbank:XP_044419825.1;Name=XP_044419825.1;gbkey=CDS;gene=LOC543106;product=inorganic phosphate transporter 2-1%2C chloroplastic;protein_id=XP_044419825.1

GO:0005887; Integral component of plasma membrane.

Kozak: TACATGC ID=cds-XP_044416432.1;Parent=rna-XM_044560497.1;Dbxref=GeneID:123141320,Genbank:XP_044416432.1;Name=XP_044416432.1;gbkey=CDS;gene=LOC123141320;product=B-box zinc finger protein 22-like;protein_id=XP_044416432.1

GO:0005634; Nucleus.

Kozak: TTCATGT ID=cds-XP_044416472.1;Parent=rna-XM_044560537.1;Dbxref=GeneID:123141368,Genbank:XP_044416472.1;Name=XP_044416472.1;gbkey=CDS;gene=LOC123141368;product=zinc finger CCCH domain-containing protein 14-like;protein_id=XP_044416472.1

GO:0003677; DNA binding.

Kozak: CCAATGC ID=cds-XP_044417049.1;Parent=rna-XM_044561114.1;Dbxref=GeneID:123142096,Genbank:XP_044417049.1;Name=XP_044417049.1;gbkey=CDS;gene=LOC123142096;product=two-component response regulator ORR29-like;protein_id=XP_044417049.1

GO:0000407; Phagophore assembly site.

Kozak: CCAATGC ID=cds-XP_044417457.1;Parent=rna-XM_044561522.1;Dbxref=GeneID:123142756,Genbank:XP_044417457.1;Name=XP_044417457.1;gbkey=CDS;gene=LOC123142756;product=protein DMP3-like;protein_id=XP_044417457.1

GO:0016021; Integral component of membrane.

Kozak: TCCATGT ID=cds-XP_044417752.1;Parent=rna-XM_044561817.1;Dbxref=GeneID:123143047,Genbank:XP_044417752.1;Name=XP_044417752.1;gbkey=CDS;gene=LOC123143047;product=sucrose:sucrose 1-fructosyltransferase-like;protein_id=XP_044417752.1

GO:0016021; Integral component of membrane.

Kozak: CCCATGC ID=cds-XP_044418042.1;Parent=rna-XM_044562107.1;Dbxref=GeneID:123143253,Genbank:XP_044418042.1;Name=XP_044418042.1;gbkey=CDS;gene=LOC123143253;product=protein trichome birefringence-like 12;protein_id=XP_044418042.1

GO:0005794; Golgi apparatus.

Kozak: TACATGC ID=cds-XP_044418336.1;Parent=rna-XM_044562401.1;Dbxref=GeneID:123143471,Genbank:XP_044418336.1;Name=XP_044418336.1;gbkey=CDS;gene=LOC123143471;product=

Putative pentatricopeptide repeat-containing protein At1g19290 isoform X1.

Kozak: CAGATGC ID=cds-XP_044418422.1;Parent=rna-XM_044562487.1;Dbxref=GeneID:123143542,Genbank:XP_044418422.1;Name=XP_044418422.1;gbkey=CDS;gene=LOC123143542;product=polyubiquitin 11;protein_id=XP_044418422.1

GO:0005737; Cytoplasm.

Kozak: CAGATGC ID=cds-XP_044418426.1;Parent=rna-XM_044562491.1;Dbxref=GeneID:123143545,Genbank:XP_044418426.1;Name=XP_044418426.1;Note=The sequence of the model RefSeq protein was modified relative to this genomic sequence to represent the inferred CDS: added 195 bases not found in genome assembly;end_range=62479877,.;exception=annotated by transcript or proteomic data;gbkey=CDS;gene=LOC123143545;inference=similar to RNA sequence%2C mRNA (same species):INSD:JP824514.1;partial=true;product=polyubiquitin-like;protein_id=XP_044418426.1

GO:0005737; Cytoplasm.

Kozak: CCCATGT ID=cds-XP_044418486.1;Parent=rna-XM_044562551.1;Dbxref=GeneID:123143615,Genbank:XP_044418486.1;Name=XP_044418486.1;gbkey=CDS;gene=LOC123143615;product=

Phosphatidylinositol/phosphatidylcholine transfer protein SFH12-like isoform X1.

Kozak: CCAATGC ID=cds-XP_044418821.1;Parent=rna-XM_044562886.1;Dbxref=GeneID:123143890,Genbank:XP_044418821.1;Name=XP_044418821.1;gbkey=CDS;gene=LOC123143890;product=tyrosine-sulfated glycopeptide receptor 1-like;protein_id=XP_044418821.1

GO:0016021; Integral component of membrane.

Kozak: CCAATGC ID=cds-XP_044418822.1;Parent=rna-XM_044562887.1;Dbxref=GeneID:123143891,Genbank:XP_044418822.1;Name=XP_044418822.1;gbkey=CDS;gene=LOC123143891;product=tyrosine-sulfated glycopeptide receptor 1-like;protein_id=XP_044418822.1

GO:0016021; Integral component of membrane.

Kozak: CAGATGC ID=cds-XP_044418869.1;Parent=rna-XM_044562934.1;Dbxref=GeneID:123143944,Genbank:XP_044418869.1;Name=XP_044418869.1;gbkey=CDS;gene=LOC123143944;product=polyubiquitin isoform X1;protein_id=XP_044418869.1

GO:0005737; Cytoplasm.

Kozak: CAGATGC ID=cds-XP_044418872.1;Parent=rna-XM_044562937.1;Dbxref=GeneID:123143946,Genbank:XP_044418872.1;Name=XP_044418872.1;gbkey=CDS;gene=LOC123143946;product=polyubiquitin 11;protein_id=XP_044418872.1

GO:0005737; Cytoplasm.

Kozak: CAGATGC ID=cds-XP_044418873.1;Parent=rna-XM_044562938.1;Dbxref=GeneID:123143947,Genbank:XP_044418873.1;Name=XP_044418873.1;gbkey=CDS;gene=LOC123143947;product=polyubiquitin isoform X1;protein_id=XP_044418873.1

GO:0005737; Cytoplasm.

Kozak: CCCATGT ID=cds-XP_044419456.1;Parent=rna-XM_044563521.1;Dbxref=GeneID:123144397,Genbank:XP_044419456.1;Name=XP_044419456.1;gbkey=CDS;gene=LOC123144397;product=probable serine/threonine-protein kinase SIS8;protein_id=XP_044419456.1

GO:0005737; Cytoplasm.

Kozak: CAGATGC ID=cds-XP_044419677.1;Parent=rna-XM_044563742.1;Dbxref=GeneID:123144539,Genbank:XP_044419677.1;Name=XP_044419677.1;gbkey=CDS;gene=LOC123144539;product=putative elongation factor TypA-like SVR3%2C chloroplastic isoform X1;protein_id=XP_044419677.1

GO:0043231; Intracellular membrane-bounded organelle.

Kozak: CCAATGC ID=cds-XP_044420149.1;Parent=rna-XM_044564214.1;Dbxref=GeneID:123144940,Genbank:XP_044420149.1;Name=XP_044420149.1;gbkey=CDS;gene=LOC123144940;product=protein SHORTAGE IN CHIASMATA 1 homolog;protein_id=XP_044420149.1

GO:0000712; Resolution of meiotic recombination intermediates.

Kozak: TACATGC ID=cds-XP_044420183.1;Parent=rna-XM_044564248.1;Dbxref=GeneID:123144972,Genbank:XP_044420183.1;Name=XP_044420183.1;gbkey=CDS;gene=LOC123144972;product=probable WRKY transcription factor 12;protein_id=XP_044420183.1

GO:0005634; Nucleus.

Kozak: TCCATGT ID=cds-XP_044420193.1;Parent=rna-XM_044564258.1;Dbxref=GeneID:123144983,Genbank:XP_044420193.1;Name=XP_044420193.1;gbkey=CDS;gene=LOC123144983;product=

Transcription initiation factor TFIID subunit 4b-like.

Kozak: CAGATGC ID=cds-XP_044420316.1;Parent=rna-XM_044564381.1;Dbxref=GeneID:123145070,Genbank:XP_044420316.1;Name=XP_044420316.1;gbkey=CDS;gene=LOC123145070;product=protein argonaute 1A-like isoform X3;protein_id=XP_044420316.1

GO:0005737; Cytoplasm.

Kozak: CCAATGC ID=cds-XP_044420338.1;Parent=rna-XM_044564403.1;Dbxref=GeneID:123145088,Genbank:XP_044420338.1;Name=XP_044420338.1;gbkey=CDS;gene=LOC123145088;product=U-box domain-containing protein 26-like;protein_id=XP_044420338.1

GO:0061630; Ubiquitin protein ligase activity.

Kozak: CCAATGC ID=cds-XP_044420348.1;Parent=rna-XM_044564413.1;Dbxref=GeneID:123145097,Genbank:XP_044420348.1;Name=XP_044420348.1;gbkey=CDS;gene=LOC123145097;product=

Pentatricopeptide repeat-containing protein At1g74900%2C mitochondrial-like.

Kozak: CCCATGC ID=cds-XP_044420512.1;Parent=rna-XM_044564577.1;Dbxref=GeneID:123145212,Genbank:XP_044420512.1;Name=XP_044420512.1;gbkey=CDS;gene=LOC123145212;product=uncharacterized protein LOC123145212;protein_id=XP_044420512.1

GO:0016021; Integral component of membrane.

Kozak: CCCATGC ID=cds-XP_044420689.1;Parent=rna-XM_044564754.1;Dbxref=GeneID:123145354,Genbank:XP_044420689.1;Name=XP_044420689.1;gbkey=CDS;gene=LOC123145354;product=dof zinc finger protein 4-like;protein_id=XP_044420689.1

GO:0005634; Nucleus.

Kozak: CAGATGC ID=cds-XP_044420826.1;Parent=rna-XM_044564891.1;Dbxref=GeneID:123145463,Genbank:XP_044420826.1;Name=XP_044420826.1;gbkey=CDS;gene=LOC123145463;product=pentatricopeptide repeat-containing protein PPR5 homolog%2C chloroplastic-like;protein_id=XP_044420826.1

GO:0003729; mRNA binding.

Kozak: CCAATGC ID=cds-XP_044420914.1;Parent=rna-XM_044564979.1;Dbxref=GeneID:123145548,Genbank:XP_044420914.1;Name=XP_044420914.1;gbkey=CDS;gene=LOC123145548;product=factor of DNA methylation 1-like;protein_id=XP_044420914.1

GO:0080188; Gene silencing by RNA-directed DNA methylation.

Kozak: CAGATGC ID=cds-XP_044420925.1;Parent=rna-XM_044564990.1;Dbxref=GeneID:123145558,Genbank:XP_044420925.1;Name=XP_044420925.1;gbkey=CDS;gene=LOC123145558;product=

MDIS1-interacting receptor like kinase 2-like.

Kozak: CCAATGC ID=cds-XP_044421027.1;Parent=rna-XM_044565092.1;Dbxref=GeneID:123145629,Genbank:XP_044421027.1;Name=XP_044421027.1;gbkey=CDS;gene=LOC123145629;product=thioredoxin-like 3-1%2C chloroplastic;protein_id=XP_044421027.1

GO:0009570; Chloroplast stroma.

Kozak: CCAATGC ID=cds-XP_044421331.1;Parent=rna-XM_044565396.1;Dbxref=GeneID:123145884,Genbank:XP_044421331.1;Name=XP_044421331.1;gbkey=CDS;gene=LOC123145884;product=uncharacterized protein LOC123145884;protein_id=XP_044421331.1

GO:0044260; Cellular macromolecule metabolic process.

Kozak: CGAATGT ID=cds-XP_044421619.1;Parent=rna-XM_044565684.1;Dbxref=GeneID:123146099,Genbank:XP_044421619.1;Name=XP_044421619.1;gbkey=CDS;gene=LOC123146099;product=

Putative F-box protein PP2-B12.

Kozak: TCCATGT ID=cds-XP_044373173.1;Parent=rna-XM_044517238.1;Dbxref=GeneID:123095690,Genbank:XP_044373173.1;Name=XP_044373173.1;gbkey=CDS;gene=LOC123095690;product=

Probable beta-D-xylosidase 7.

Kozak: CCCATGC ID=cds-XP_044373189.1;Parent=rna-XM_044517254.1;Dbxref=GeneID:123095704,Genbank:XP_044373189.1;Name=XP_044373189.1;gbkey=CDS;gene=LOC123095704;product=

Protein saal1-like.

Kozak: CCCATGT ID=cds-XP_044373403.1;Parent=rna-XM_044517468.1;Dbxref=GeneID:123095898,Genbank:XP_044373403.1;Name=XP_044373403.1;gbkey=CDS;gene=LOC123095898;product=protein POLLEN DEFECTIVE IN GUIDANCE 1-like;protein_id=XP_044373403.1

GO:0030176; Integral component of endoplasmic reticulum membrane.

Kozak: CAGATGC ID=cds-XP_044373766.1;Parent=rna-XM_044517831.1;Dbxref=GeneID:123096184,Genbank:XP_044373766.1;Name=XP_044373766.1;gbkey=CDS;gene=LOC123096184;product=pentatricopeptide repeat-containing protein At3g12770-like;protein_id=XP_044373766.1

GO:0000145; Exocyst.

Kozak: CCCATGT ID=cds-XP_044373996.1;Parent=rna-XM_044518061.1;Dbxref=GeneID:123096354,Genbank:XP_044373996.1;Name=XP_044373996.1;gbkey=CDS;gene=LOC123096354;product=protein transport protein Sec24-like CEF;protein_id=XP_044373996.1

GO:0030127; COPII vesicle.

Kozak: CCCATGT ID=cds-XP_044374037.1;Parent=rna-XM_044518102.1;Dbxref=GeneID:123096376,Genbank:XP_044374037.1;Name=XP_044374037.1;gbkey=CDS;gene=LOC123096376;product=probable LRR receptor-like serine/threonine-protein kinase RKF3;protein_id=XP_044374037.1

GO:0016021; Integral component of membrane.

Kozak: CCAATGC ID=cds-XP_044374038.1;Parent=rna-XM_044518103.1;Dbxref=GeneID:123096377,Genbank:XP_044374038.1;Name=XP_044374038.1;gbkey=CDS;gene=LOC123096377;product=protein SHORTAGE IN CHIASMATA 1 homolog isoform X1;protein_id=XP_044374038.1

GO:0061630; Ubiquitin protein ligase activity.

Kozak: CCCATGT ID=cds-XP_044374503.1;Parent=rna-XM_044518568.1;Dbxref=GeneID:123096789,Genbank:XP_044374503.1;Name=XP_044374503.1;gbkey=CDS;gene=LOC123096789;product=NADH-ubiquinone oxidoreductase chain 4-like;protein_id=XP_044374503.1

GO:0016021; Integral component of membrane.

Kozak: CCCATGC ID=cds-XP_044374691.1;Parent=rna-XM_044518756.1;Dbxref=GeneID:123096970,Genbank:XP_044374691.1;Name=XP_044374691.1;gbkey=CDS;gene=LOC123096970;product=O-fucosyltransferase 9-like;protein_id=XP_044374691.1

GO:0016740; Transferase activity.

Kozak: CAGATGC ID=cds-XP_044374883.1;Parent=rna-XM_044518948.1;Dbxref=GeneID:123097249,Genbank:XP_044374883.1;Name=XP_044374883.1;gbkey=CDS;gene=LOC123097249;product=armadillo repeat-containing protein 8-like isoform X1;protein_id=XP_044374883.1

GO:0005737; Cytoplasm.

Kozak: CCAATGC ID=cds-XP_044375036.1;Parent=rna-XM_044519101.1;Dbxref=GeneID:123097390,Genbank:XP_044375036.1;Name=XP_044375036.1;gbkey=CDS;gene=LOC123097390;product=phosphatidylinositol 4-phosphate 5-kinase 6-like;protein_id=XP_044375036.1

GO:0005886; Plasma membrane.

Kozak: CCCATGT ID=cds-XP_044375406.1;Parent=rna-XM_044519471.1;Dbxref=GeneID:123097664,Genbank:XP_044375406.1;Name=XP_044375406.1;gbkey=CDS;gene=LOC123097664;product=FIP1[III]-like protein isoform X1;protein_id=XP_044375406.1

GO:0005634; Nucleus.

Kozak: CCCATGC ID=cds-XP_044375602.1;Parent=rna-XM_044519667.1;Dbxref=GeneID:123097830,Genbank:XP_044375602.1;Name=XP_044375602.1;gbkey=CDS;gene=LOC123097830;product=cardiolipin synthase (CMP-forming)%2C mitochondrial-like;protein_id=XP_044375602.1

GO:0016021; Integral component of membrane.

Kozak: CCAATGC ID=cds-XP_044375819.1;Parent=rna-XM_044519884.1;Dbxref=GeneID:123098015,Genbank:XP_044375819.1;Name=XP_044375819.1;gbkey=CDS;gene=LOC123098015;product=imidazole glycerol phosphate synthase hisHF%2C chloroplastic-like;protein_id=XP_044375819.1

GO:0009507; Chloroplast.

Kozak: CCCATGC ID=cds-XP_044375850.1;Parent=rna-XM_044519915.1;Dbxref=GeneID:123098042,Genbank:XP_044375850.1;Name=XP_044375850.1;gbkey=CDS;gene=LOC123098042;product=

Mucin-7-like.

Kozak: CAGATGC ID=cds-XP_044376036.1;Parent=rna-XM_044520101.1;Dbxref=GeneID:123098188,Genbank:XP_044376036.1;Name=XP_044376036.1;gbkey=CDS;gene=LOC123098188;product=

Pentatricopeptide repeat-containing protein At5g47360-like.

Kozak: CCCATGC ID=cds-XP_044376584.1;Parent=rna-XM_044520649.1;Dbxref=GeneID:123098615,Genbank:XP_044376584.1;Name=XP_044376584.1;gbkey=CDS;gene=LOC123098615;product=tRNA-specific 2-thiouridylase MnmA-like;protein_id=XP_044376584.1

GO:0005524; ATP binding.

Kozak: TACATGC ID=cds-XP_044376600.1;Parent=rna-XM_044520665.1;Dbxref=GeneID:123098631,Genbank:XP_044376600.1;Name=XP_044376600.1;gbkey=CDS;gene=LOC123098631;product=

GDSL esterase/lipase LTL1-like isoform X1.

Kozak: TACATGC ID=cds-XP_044376763.1;Parent=rna-XM_044520828.1;Dbxref=GeneID:123098757,Genbank:XP_044376763.1;Name=XP_044376763.1;gbkey=CDS;gene=LOC123098757;product=

GDSL esterase/lipase LTL1-like.

Kozak: CCCATGC ID=cds-XP_044376775.1;Parent=rna-XM_044520840.1;Dbxref=GeneID:123098761,Genbank:XP_044376775.1;Name=XP_044376775.1;gbkey=CDS;gene=LOC123098761;product=beta-glucuronosyltransferase GlcAT14A-like;protein_id=XP_044376775.1

GO:0016020; Membrane.

Kozak: CGAATGT ID=cds-XP_044376909.1;Parent=rna-XM_044520974.1;Dbxref=GeneID:123098900,Genbank:XP_044376909.1;Name=XP_044376909.1;gbkey=CDS;gene=LOC123098900;product=ATP phosphoribosyltransferase%2C chloroplastic-like;protein_id=XP_044376909.1

GO:0005737; Cytoplasm.

Kozak: CCCATGC ID=cds-XP_044377001.1;Parent=rna-XM_044521066.1;Dbxref=GeneID:123098968,Genbank:XP_044377001.1;Name=XP_044377001.1;gbkey=CDS;gene=LOC123098968;product=uncharacterized protein LOC123098968;protein_id=XP_044377001.1

GO:0016021; Integral component of membrane.

Kozak: CCCATGC ID=cds-XP_044377155.1;Parent=rna-XM_044521220.1;Dbxref=GeneID:123099083,Genbank:XP_044377155.1;Name=XP_044377155.1;gbkey=CDS;gene=LOC123099083;product=BTB/POZ and MATH domain-containing protein 2-like;protein_id=XP_044377155.1

GO:0005634; Nucleus.

Kozak: CCCATGC ID=cds-XP_044377295.1;Parent=rna-XM_044521360.1;Dbxref=GeneID:123099194,Genbank:XP_044377295.1;Name=XP_044377295.1;gbkey=CDS;gene=LOC123099194;product=

Nucleolin-like.

Kozak: CAGATGC ID=cds-XP_044377863.1;Parent=rna-XM_044521928.1;Dbxref=GeneID:123099896,Genbank:XP_044377863.1;Name=XP_044377863.1;gbkey=CDS;gene=LOC123099896;product=

Proline-rich receptor-like protein kinase PERK10.

Kozak: CCAATGT ID=cds-XP_044377952.1;Parent=rna-XM_044522017.1;Dbxref=GeneID:123100015,Genbank:XP_044377952.1;Name=XP_044377952.1;gbkey=CDS;gene=LOC123100015;product=probable membrane-associated kinase regulator 4;protein_id=XP_044377952.1

GO:0005886; Plasma membrane.

Kozak: CCAATGC ID=cds-XP_044374053.1;Parent=rna-XM_044518118.1;Dbxref=GeneID:780573,Genbank:XP_044374053.1;Name=XP_044374053.1;gbkey=CDS;gene=LOC780573;product=calcium-dependent protein kinase 24;protein_id=XP_044374053.1

GO:0005737; Cytoplasm.

Kozak: CCCATGT ID=cds-XP_044373243.1;Parent=rna-XM_044517308.1;Dbxref=GeneID:123095745,Genbank:XP_044373243.1;Name=XP_044373243.1;gbkey=CDS;gene=LOC123095745;product=beta-glucuronosyltransferase GlcAT14B-like;protein_id=XP_044373243.1

GO:0016020; Membrane.

Kozak: CCCATGC ID=cds-XP_044373730.1;Parent=rna-XM_044517795.1;Dbxref=GeneID:123096165,Genbank:XP_044373730.1;Name=XP_044373730.1;gbkey=CDS;gene=LOC123096165;product=endonuclease III homolog 1%2C chloroplastic-like isoform X2;protein_id=XP_044373730.1

GO:0042644; Chloroplast nucleoid.

Kozak: TGTATGT ID=cds-XP_044374436.1;Parent=rna-XM_044518501.1;Dbxref=GeneID:123096740,Genbank:XP_044374436.1;Name=XP_044374436.1;gbkey=CDS;gene=LOC123096740;product=alanine--tRNA ligase-like;protein_id=XP_044374436.1

GO:0009507; Chloroplast.

Kozak: CCCATGC ID=cds-XP_044374451.1;Parent=rna-XM_044518516.1;Dbxref=GeneID:123096749,Genbank:XP_044374451.1;Name=XP_044374451.1;gbkey=CDS;gene=LOC123096749;product=scarecrow-like protein 34;protein_id=XP_044374451.1

GO:0005634; Nucleus.

Kozak: CCAATGC ID=cds-XP_044374859.1;Parent=rna-XM_044518924.1;Dbxref=GeneID:123097204,Genbank:XP_044374859.1;Name=XP_044374859.1;gbkey=CDS;gene=LOC123097204;product=O-fucosyltransferase 1-like;protein_id=XP_044374859.1

GO:0005737; Cytoplasm.

Kozak: CCAATGC ID=cds-XP_044375244.1;Parent=rna-XM_044519309.1;Dbxref=GeneID:123097541,Genbank:XP_044375244.1;Name=XP_044375244.1;gbkey=CDS;gene=LOC123097541;product=transcription factor RF2b-like;protein_id=XP_044375244.1

GO:0005634; Nucleus.

Kozak: TCCATGT ID=cds-XP_044375413.1;Parent=rna-XM_044519478.1;Dbxref=GeneID:123097670,Genbank:XP_044375413.1;Name=XP_044375413.1;gbkey=CDS;gene=LOC123097670;product=DEAD-box ATP-dependent RNA helicase 24-like;protein_id=XP_044375413.1

GO:0005634; Nucleus.

Kozak: TACATGC ID=cds-XP_044375482.1;Parent=rna-XM_044519547.1;Dbxref=GeneID:123097728,Genbank:XP_044375482.1;Name=XP_044375482.1;gbkey=CDS;gene=LOC123097728;product=sex determination protein tasselseed-2-like;protein_id=XP_044375482.1

GO:0005938; Cell cortex.

Kozak: CAGATGC ID=cds-XP_044375713.1;Parent=rna-XM_044519778.1;Dbxref=GeneID:123097931,Genbank:XP_044375713.1;Name=XP_044375713.1;gbkey=CDS;gene=LOC123097931;product=uncharacterized protein LOC123097931;protein_id=XP_044375713.1

GO:0016021; Integral component of membrane.

Kozak: CGAATGT ID=cds-XP_044375956.1;Parent=rna-XM_044520021.1;Dbxref=GeneID:123098128,Genbank:XP_044375956.1;Name=XP_044375956.1;gbkey=CDS;gene=LOC123098128;product=anaphase-promoting complex subunit 6-like;protein_id=XP_044375956.1

GO:0005680; Anaphase-promoting complex.

Kozak: CCAATGT ID=cds-XP_044376187.1;Parent=rna-XM_044520252.1;Dbxref=GeneID:123098292,Genbank:XP_044376187.1;Name=XP_044376187.1;gbkey=CDS;gene=LOC123098292;product=glucan endo-1%2C3-beta-glucosidase 1-like;protein_id=XP_044376187.1

GO:0046658; Anchored component of plasma membrane.

Kozak: TTCATGT ID=cds-XP_044376272.1;Parent=rna-XM_044520337.1;Dbxref=GeneID:123098366,Genbank:XP_044376272.1;Name=XP_044376272.1;gbkey=CDS;gene=LOC123098366;product=

Pentatricopeptide repeat-containing protein At2g16880-like.

Kozak: TACATGC ID=cds-XP_044376292.1;Parent=rna-XM_044520357.1;Dbxref=GeneID:123098385,Genbank:XP_044376292.1;Name=XP_044376292.1;gbkey=CDS;gene=LOC123098385;product=probable GMP synthase [glutamine-hydrolyzing];protein_id=XP_044376292.1

GO:0008725; DNA-3-methyladenine glycosylase activity.

Kozak: TGTATGT ID=cds-XP_044376385.1;Parent=rna-XM_044520450.1;Dbxref=GeneID:123098463,Genbank:XP_044376385.1;Name=XP_044376385.1;gbkey=CDS;gene=LOC123098463;product=ABC transporter G family member 22-like;protein_id=XP_044376385.1

GO:0046658; Anchored component of plasma membrane.

Kozak: CAGATGC ID=cds-XP_044376532.1;Parent=rna-XM_044520597.1;Dbxref=GeneID:123098564,Genbank:XP_044376532.1;Name=XP_044376532.1;gbkey=CDS;gene=LOC123098564;product=

Pentatricopeptide repeat-containing protein At5g15300-like isoform X1.

Kozak: CCCATGT ID=cds-XP_044376582.1;Parent=rna-XM_044520647.1;Dbxref=GeneID:123098613,Genbank:XP_044376582.1;Name=XP_044376582.1;gbkey=CDS;gene=LOC123098613;product=adenylosuccinate synthetase%2C chloroplastic-like;protein_id=XP_044376582.1

GO:0009507; Chloroplast.

Koak: CCCATGT ID=cds-XP_044376786.1;Parent=rna-XM_044520851.1;Dbxref=GeneID:123098773,Genbank:XP_044376786.1;Name=XP_044376786.1;gbkey=CDS;gene=LOC123098773;product=pentatricopeptide repeat-containing protein At1g76280-like;protein_id=XP_044376786.1

GO:0008663; 2',3'-cyclic-nucleotide 2'-phosphodiesterase activity.

Kozak: CCCATGT ID=cds-XP_044376837.1;Parent=rna-XM_044520902.1;Dbxref=GeneID:123098826,Genbank:XP_044376837.1;Name=XP_044376837.1;gbkey=CDS;gene=LOC123098826;product=uncharacterized protein LOC123098826;protein_id=XP_044376837.1

GO:0016021; Integral component of membrane.

Kozak: TCCATGT ID=cds-XP_044376908.1;Parent=rna-XM_044520973.1;Dbxref=GeneID:123098899,Genbank:XP_044376908.1;Name=XP_044376908.1;gbkey=CDS;gene=LOC123098899;product=probable serine acetyltransferase 2;protein_id=XP_044376908.1

GO:0005737; Cytoplasm.

Kozak: CCAATGT ID=cds-XP_044377079.1;Parent=rna-XM_044521144.1;Dbxref=GeneID:123099034,Genbank:XP_044377079.1;Name=XP_044377079.1;gbkey=CDS;gene=LOC123099034;product=

Pentatricopeptide repeat-containing protein At3g04760%2C chloroplastic-like isoform X1.

Kozak: CCCATGT ID=cds-XP_044377218.1;Parent=rna-XM_044521283.1;Dbxref=GeneID:123099138,Genbank:XP_044377218.1;Name=XP_044377218.1;gbkey=CDS;gene=LOC123099138;product=metalloendoproteinase 4-MMP-like;protein_id=XP_044377218.1

GO:0031225; Anchored component of membrane.

Kozak: CCAATGC ID=cds-XP_044377274.1;Parent=rna-XM_044521339.1;Dbxref=GeneID:123099176,Genbank:XP_044377274.1;Name=XP_044377274.1;gbkey=CDS;gene=LOC123099176;product=uncharacterized protein LOC123099176;protein_id=XP_044377274.1

GO:0003755; Peptidyl-prolyl cis-trans isomerase activity.

Kozak: TGTATGT ID=cds-XP_044377383.1;Parent=rna-XM_044521448.1;Dbxref=GeneID:123099269,Genbank:XP_044377383.1;Name=XP_044377383.1;gbkey=CDS;gene=LOC123099269;product=alanine--tRNA ligase-like;protein_id=XP_044377383.1

GO:0009507; Chloroplast.

Kozak: TCCATGT ID=cds-XP_044377492.1;Parent=rna-XM_044521557.1;Dbxref=GeneID:123099386,Genbank:XP_044377492.1;Name=XP_044377492.1;gbkey=CDS;gene=LOC123099386;product=protein argonaute 12-like;protein_id=XP_044377492.1

GO:0005737; Cytoplasm.

Kozak: TTAATGC ID=cds-XP_044377857.1;Parent=rna-XM_044521922.1;Dbxref=GeneID:123099879,Genbank:XP_044377857.1;Name=XP_044377857.1;gbkey=CDS;gene=LOC123099879;product=uncharacterized protein LOC123099879;protein_id=XP_044377857.1

GO:0098791; Golgi apparatus subcompartment.

Kozak: TCCATGT ID=cds-XP_044378141.1;Parent=rna-XM_044522206.1;Dbxref=GeneID:123100252,Genbank:XP_044378141.1;Name=XP_044378141.1;gbkey=CDS;gene=LOC123100252;product=

Late embryogenesis abundant protein D-34-like.

Kozak: TCCATGT ID=cds-XP_044378183.1;Parent=rna-XM_044522248.1;Dbxref=GeneID:123100306,Genbank:XP_044378183.1;Name=XP_044378183.1;gbkey=CDS;gene=LOC123100306;product=

Putative MO25-like protein At5g47540 isoform X1

Kozak: TCCATGT ID=cds-XP_044402950.1;Parent=rna-XM_044547015.1;Dbxref=GeneID:123127366,Genbank:XP_044402950.1;Name=XP_044402950.1;gbkey=CDS;gene=LOC123127366;product=kinesin-like protein KIN-14D;protein_id=XP_044402950.1

GO:0005874; Microtubule.

Kozak: TACATGC ID=cds-XP_044403242.1;Parent=rna-XM_044547307.1;Dbxref=GeneID:123127563,Genbank:XP_044403242.1;Name=XP_044403242.1;gbkey=CDS;gene=LOC123127563;product=

Protein FAR1-RELATED SEQUENCE 5-like.

Kozak: TCCATGT ID=cds-XP_044403258.1;Parent=rna-XM_044547323.1;Dbxref=GeneID:123127575,Genbank:XP_044403258.1;Name=XP_044403258.1;gbkey=CDS;gene=LOC123127575;product=inorganic phosphate transporter 2-1%2C chloroplastic-like;protein_id=XP_044403258.1

GO:0005887; Integral component of plasma membrane.

Kozak: TCCATGT ID=cds-XP_044403311.1;Parent=rna-XM_044547376.1;Dbxref=GeneID:123127621,Genbank:XP_044403311.1;Name=XP_044403311.1;gbkey=CDS;gene=LOC123127621;product=methionine aminopeptidase 1B%2C chloroplastic-like isoform X1;protein_id=XP_044403311.1

GO:0046872; Metal ion binding.

Kozak: TCCATGT ID=cds-XP_044403379.1;Parent=rna-XM_044547444.1;Dbxref=GeneID:123127668,Genbank:XP_044403379.1;Name=XP_044403379.1;gbkey=CDS;gene=LOC123127668;product=acetolactate synthase small subunit 2%2C chloroplastic-like isoform X1;protein_id=XP_044403379.1

GO:0005737; Cytoplasm.

Kozak: CCCATGC ID=cds-XP_044404051.1;Parent=rna-XM_044548116.1;Dbxref=GeneID:123128181,Genbank:XP_044404051.1;Name=XP_044404051.1;gbkey=CDS;gene=LOC123128181;product=ribose-phosphate pyrophosphokinase 4-like;protein_id=XP_044404051.1

GO:0005737; Cytoplasm.

Kozak: CCAATGC ID=cds-XP_044404588.1;Parent=rna-XM_044548653.1;Dbxref=GeneID:123128607,Genbank:XP_044404588.1;Name=XP_044404588.1;gbkey=CDS;gene=LOC123128607;product=LRR receptor-like serine/threonine-protein kinase ER2;protein_id=XP_044404588.1

GO:0016021; Integral component of membrane.

Kozak: CCCATGC ID=cds-XP_044404803.1;Parent=rna-XM_044548868.1;Dbxref=GeneID:123128771,Genbank:XP_044404803.1;Name=XP_044404803.1;gbkey=CDS;gene=LOC123128771;product=

Transcription factor LRL3-like.

Kozak: TACATGC ID=cds-XP_044404939.1;Parent=rna-XM_044549004.1;Dbxref=GeneID:123128890,Genbank:XP_044404939.1;Name=XP_044404939.1;gbkey=CDS;gene=LOC123128890;product=agmatine coumaroyltransferase-2-like;protein_id=XP_044404939.1

GO:0016747; Acyltransferase activity.

Kozak: CCCATGC ID=cds-XP_044405056.1;Parent=rna-XM_044549121.1;Dbxref=GeneID:123128994,Genbank:XP_044405056.1;Name=XP_044405056.1;gbkey=CDS;gene=LOC123128994;product=protein LUTEIN DEFICIENT 5%2C chloroplastic-like;protein_id=XP_044405056.1

GO:0016021; Integral component of membrane.

Kozak: TACATGC ID=cds-XP_044405139.1;Parent=rna-XM_044549204.1;Dbxref=GeneID:123129060,Genbank:XP_044405139.1;Name=XP_044405139.1;gbkey=CDS;gene=LOC123129060;product=putative disease resistance protein RGA1 isoform X1;protein_id=XP_044405139.1

GO:0043531; ADP binding.

Kozak: CCCATGT ID=cds-XP_044405256.1;Parent=rna-XM_044549321.1;Dbxref=GeneID:123129139,Genbank:XP_044405256.1;Name=XP_044405256.1;gbkey=CDS;gene=LOC123129139;product=ankyrin-1-like;protein_id=XP_044405256.1

GO:0016020; Membrane.

Kozak: TGTATGT ID=cds-XP_044405615.1;Parent=rna-XM_044549680.1;Dbxref=GeneID:123129569,Genbank:XP_044405615.1;Name=XP_044405615.1;gbkey=CDS;gene=LOC123129569;product=

Protein FAR1-RELATED SEQUENCE 6-like.

Kozak: CCAATGC ID=cds-XP_044405874.1;Parent=rna-XM_044549939.1;Dbxref=GeneID:123129983,Genbank:XP_044405874.1;Name=XP_044405874.1;gbkey=CDS;gene=LOC123129983;product=probable leucine-rich repeat receptor-like protein kinase At1g35710;protein_id=XP_044405874.1

GO:0016021; Integral component of membrane.

Kozak: CCCATGC ID=cds-XP_044406004.1;Parent=rna-XM_044550069.1;Dbxref=GeneID:123130121,Genbank:XP_044406004.1;Name=XP_044406004.1;gbkey=CDS;gene=LOC123130121;product=RING-H2 finger protein ATL52-like;protein_id=XP_044406004.1

GO:0016021; Integral component of membrane.

Kozak: CCAATGT ID=cds-XP_044406217.1;Parent=rna-XM_044550282.1;Dbxref=GeneID:123130368,Genbank:XP_044406217.1;Name=XP_044406217.1;gbkey=CDS;gene=LOC123130368;product=serrate RNA effector molecule-like;protein_id=XP_044406217.1

GO:0016604; Nuclear body.

Kozak: CAGATGC ID=cds-XP_044406226.1;Parent=rna-XM_044550291.1;Dbxref=GeneID:123130383,Genbank:XP_044406226.1;Name=XP_044406226.1;gbkey=CDS;gene=LOC123130383;product=protein DMP5-like;protein_id=XP_044406226.1

GO:0016021; Integral component of membrane.

Kozak: CCCATGT ID=cds-XP_044406628.1;Parent=rna-XM_044550693.1;Dbxref=GeneID:123130902,Genbank:XP_044406628.1;Name=XP_044406628.1;gbkey=CDS;gene=LOC123130902;product=probable LRR receptor-like serine/threonine-protein kinase At3g47570;protein_id=XP_044406628.1

GO:0016021; Integral component of membrane.

Kozak: CAGATGC ID=cds-XP_044406630.1;Parent=rna-XM_044550695.1;Dbxref=GeneID:123130903,Genbank:XP_044406630.1;Name=XP_044406630.1;gbkey=CDS;gene=LOC123130903;product=

Putative receptor-like protein kinase At3g47110.

Kozak: CCCATGT ID=cds-XP_044406724.1;Parent=rna-XM_044550789.1;Dbxref=GeneID:123131014,Genbank:XP_044406724.1;Name=XP_044406724.1;gbkey=CDS;gene=LOC123131014;product=7-deoxyloganetin glucosyltransferase-like;protein_id=XP_044406724.1

GO:0080043; Quercetin 3-O-glucosyltransferase activity.

Kozak: CCCATGC ID=cds-XP_044407415.1;Parent=rna-XM_044551480.1;Dbxref=GeneID:123131799,Genbank:XP_044407415.1;Name=XP_044407415.1;gbkey=CDS;gene=LOC123131799;product=peptidyl-prolyl cis-trans isomerase FKBP18%2C chloroplastic-like;protein_id=XP_044407415.1

GO:0003755; Peptidyl-prolyl cis-trans isomerase activity.

Kozak: CCCATGT ID=cds-XP_044407535.1;Parent=rna-XM_044551600.1;Dbxref=GeneID:123131892,Genbank:XP_044407535.1;Name=XP_044407535.1;gbkey=CDS;gene=LOC123131892;product=beta-fructofuranosidase%2C insoluble isoenzyme 4-like;protein_id=XP_044407535.1

GO:0004553; Hydrolase activity.

Kozak: CAGATGC ID=cds-XP_044407680.1;Parent=rna-XM_044551745.1;Dbxref=GeneID:123132003,Genbank:XP_044407680.1;Name=XP_044407680.1;gbkey=CDS;gene=LOC123132003;product=

Polyubiquitin.

Kozak: CCCATGC ID=cds-XP_044407698.1;Parent=rna-XM_044551763.1;Dbxref=GeneID:123132013,Genbank:XP_044407698.1;Name=XP_044407698.1;gbkey=CDS;gene=LOC123132013;product=ribose-phosphate pyrophosphokinase 1%2C chloroplastic;protein_id=XP_044407698.1

GO:0005737; Cytoplasm.

Kozak: CCCATGC ID=cds-XP_044407708.1;Parent=rna-XM_044551773.1;Dbxref=GeneID:123132026,Genbank:XP_044407708.1;Name=XP_044407708.1;gbkey=CDS;gene=LOC123132026;product=pentatricopeptide repeat-containing protein At4g01990%2C mitochondrial-like;protein_id=XP_044407708.1

GO:0005739; Mitochondrion.

Kozak: CCCATGT ID=cds-XP_044407709.1;Parent=rna-XM_044551774.1;Dbxref=GeneID:123132027,Genbank:XP_044407709.1;Name=XP_044407709.1;gbkey=CDS;gene=LOC123132027;product=pentatricopeptide repeat-containing protein At4g01990%2C mitochondrial-like;protein_id=XP_044407709.1

GO:0005739; Mitochondrion.

Kozak: CCCATGC ID=cds-XP_044407710.1;Parent=rna-XM_044551775.1;Dbxref=GeneID:123132028,Genbank:XP_044407710.1;Name=XP_044407710.1;gbkey=CDS;gene=LOC123132028;product=pentatricopeptide repeat-containing protein At1g60770-like;protein_id=XP_044407710.1

GO:0005739; Mitochondrion.

Kozak: CCCATGT ID=cds-XP_044407711.1;Parent=rna-XM_044551776.1;Dbxref=GeneID:123132029,Genbank:XP_044407711.1;Name=XP_044407711.1;gbkey=CDS;gene=LOC123132029;product=pentatricopeptide repeat-containing protein At4g01990%2C mitochondrial-like;protein_id=XP_044407711.1

GO:0005739; Mitochondrion.

Kozak: CCCATGT ID=cds-XP_044407773.1;Parent=rna-XM_044551838.1;Dbxref=GeneID:123132078,Genbank:XP_044407773.1;Name=XP_044407773.1;gbkey=CDS;gene=LOC123132078;product=

Phosphatidylinositol/phosphatidylcholine transfer protein SFH6-like isoform X1.

Kozak: CAGATGC ID=cds-XP_044407980.1;Parent=rna-XM_044552045.1;Dbxref=GeneID:123132258,Genbank:XP_044407980.1;Name=XP_044407980.1;gbkey=CDS;gene=LOC123132258;product=polyubiquitin 11;protein_id=XP_044407980.1

GO:0005737; Cytoplasm.

Kozak: CCCATGC ID=cds-XP_044408236.1;Parent=rna-XM_044552301.1;Dbxref=GeneID:123132501,Genbank:XP_044408236.1;Name=XP_044408236.1;gbkey=CDS;gene=LOC123132501;product=DNA-directed RNA polymerase III subunit 2-like;protein_id=XP_044408236.1

GO:0005666; RNA polymerase III complex.

Kozak. CCCATGT ID=cds-XP_044404354.1;Parent=rna-XM_044548419.1;Dbxref=GeneID:543429,Genbank:XP_044404354.1;Name=XP_044404354.1;gbkey=CDS;gene=LOC543429;product=dynamin-2A;protein_id=XP_044404354.1

GO:0005874; Microtubule.

Kozak: CAGATGC ID=cds-XP_044408089.1;Parent=rna-XM_044552154.1;Dbxref=GeneID:100127086,Genbank:XP_044408089.1;Name=XP_044408089.1;Note=The sequence of the model RefSeq protein was modified relative to this genomic sequence to represent the inferred CDS: deleted 1 base in 1 codon;exception=unclassified translation discrepancy;gbkey=CDS;gene=LOC100127086;product=LOW QUALITY PROTEIN: polyubiquitin;protein_id=XP_044408089.1

GO:0005737; Cytoplasm.

Kozak: CCAATGT ID=cds-XP_044402752.1;Parent=rna-XM_044546817.1;Dbxref=GeneID:123127229,Genbank:XP_044402752.1;Name=XP_044402752.1;gbkey=CDS;gene=LOC123127229;product=cyclase-like protein 1;protein_id=XP_044402752.1

GO:0004061; Arylformamidase activity.

Kozak: CCCATGT ID=cds-XP_044402908.1;Parent=rna-XM_044546973.1;Dbxref=GeneID:123127322,Genbank:XP_044402908.1;Name=XP_044402908.1;gbkey=CDS;gene=LOC123127322;product=probable serine/threonine-protein kinase SIS8;protein_id=XP_044402908.1

GO:0005737; Cytoplasm.

Kozak: TCCATGT ID=cds-XP_044403132.1;Parent=rna-XM_044547197.1;Dbxref=GeneID:123127471,Genbank:XP_044403132.1;Name=XP_044403132.1;gbkey=CDS;gene=LOC123127471;product=prohibitin-3%2C mitochondrial-like;protein_id=XP_044403132.1

GO:0005743; Mitochondrial inner membrane.

Kozak: CCCATGC ID=cds-XP_044403303.1;Parent=rna-XM_044547368.1;Dbxref=GeneID:123127614,Genbank:XP_044403303.1;Name=XP_044403303.1;gbkey=CDS;gene=LOC123127614;product=telomere length regulation protein TEL2 homolog;protein_id=XP_044403303.1

GO:0070209; ASTRA complex.

Kozak: CCAATGC ID=cds-XP_044403636.1;Parent=rna-XM_044547701.1;Dbxref=GeneID:123127844,Genbank:XP_044403636.1;Name=XP_044403636.1;gbkey=CDS;gene=LOC123127844;product=protein SHORTAGE IN CHIASMATA 1 homolog;protein_id=XP_044403636.1

GO:0000712; Resolution of meiotic recombination intermediates.

Kozak: TACATGC ID=cds-XP_044403666.1;Parent=rna-XM_044547731.1;Dbxref=GeneID:123127879,Genbank:XP_044403666.1;Name=XP_044403666.1;gbkey=CDS;gene=LOC123127879;product=probable WRKY transcription factor 12;protein_id=XP_044403666.1

GO:0005634; Nucleus.

Kozak: TCCATGT ID=cds-XP_044403682.1;Parent=rna-XM_044547747.1;Dbxref=GeneID:123127888,Genbank:XP_044403682.1;Name=XP_044403682.1;gbkey=CDS;gene=LOC123127888;product=transcription initiation factor TFIID subunit 4b-like;protein_id=XP_044403682.1

GO:0005669; Transcription factor TFIID complex.

Kozak: CCAATGC ID=cds-XP_044403812.1;Parent=rna-XM_044547877.1;Dbxref=GeneID:123127991,Genbank:XP_044403812.1;Name=XP_044403812.1;gbkey=CDS;gene=LOC123127991;product=U-box domain-containing protein 26-like;protein_id=XP_044403812.1

GO:0061630; Ubiquitin protein ligase activity.

Kozak: CCAATGC ID=cds-XP_044403825.1;Parent=rna-XM_044547890.1;Dbxref=GeneID:123128002,Genbank:XP_044403825.1;Name=XP_044403825.1;gbkey=CDS;gene=LOC123128002;product=

Pentatricopeptide repeat-containing protein At1g74900%2C mitochondrial-like.

Kozak: CCCATGC ID=cds-XP_044403987.1;Parent=rna-XM_044548052.1;Dbxref=GeneID:123128122,Genbank:XP_044403987.1;Name=XP_044403987.1;gbkey=CDS;gene=LOC123128122;product=uncharacterized protein LOC123128122;protein_id=XP_044403987.1

GO:0016021; Integral component of membrane.

Kozak: CCCATGC ID=cds-XP_044404131.1;Parent=rna-XM_044548196.1;Dbxref=GeneID:123128239,Genbank:XP_044404131.1;Name=XP_044404131.1;gbkey=CDS;gene=LOC123128239;product=enhancer of mRNA-decapping protein 4-like;protein_id=XP_044404131.1

GO:0000932; P-body.

Kozak: CCCATGC ID=cds-XP_044404265.1;Parent=rna-XM_044548330.1;Dbxref=GeneID:123128350,Genbank:XP_044404265.1;Name=XP_044404265.1;gbkey=CDS;gene=LOC123128350;product=aspartic proteinase-like protein 1 isoform X1;protein_id=XP_044404265.1

GO:0004190; Aspartic-type endopeptidase activity.

Kozak: CAGATGC ID=cds-XP_044404274.1;Parent=rna-XM_044548339.1;Dbxref=GeneID:123128355,Genbank:XP_044404274.1;Name=XP_044404274.1;gbkey=CDS;gene=LOC123128355;product=pentatricopeptide repeat-containing protein PPR5 homolog%2C chloroplastic-like;protein_id=XP_044404274.1

GO:0003729; mRNA binding.

Kozak: CCAATGC ID=cds-XP_044404458.1;Parent=rna-XM_044548523.1;Dbxref=GeneID:123128498,Genbank:XP_044404458.1;Name=XP_044404458.1;gbkey=CDS;gene=LOC123128498;product=thioredoxin-like 3-1%2C chloroplastic;protein_id=XP_044404458.1

GO:0009570; Chloroplast stroma.

Kozak: CCAATGC ID=cds-XP_044404773.1;Parent=rna-XM_044548838.1;Dbxref=GeneID:123128747,Genbank:XP_044404773.1;Name=XP_044404773.1;gbkey=CDS;gene=LOC123128747;product=uncharacterized protein LOC123128747;protein_id=XP_044404773.1

GO:0044260; Cellular macromolecule metabolic process.

Kozak: CCCATGT ID=cds-XP_044405636.1;Parent=rna-XM_044549701.1;Dbxref=GeneID:123129600,Genbank:XP_044405636.1;Name=XP_044405636.1;gbkey=CDS;gene=LOC123129600;product=

NADH-ubiquinone oxidoreductase chain 4-like.

Kozak: TACATGC ID=cds-XP_044405698.1;Parent=rna-XM_044549763.1;Dbxref=GeneID:123129704,Genbank:XP_044405698.1;Name=XP_044405698.1;gbkey=CDS;gene=LOC123129704;product=

Zinc finger MYM-type protein 1-like.

Kozak: TACATGC ID=cds-XP_044405720.1;Parent=rna-XM_044549785.1;Dbxref=GeneID:123129745,Genbank:XP_044405720.1;Name=XP_044405720.1;gbkey=CDS;gene=LOC123129745;product=

Zinc finger protein ENHYDROUS-like.

Kozak: CCAATGC ID=cds-XP_044405753.1;Parent=rna-XM_044549818.1;Dbxref=GeneID:123129798,Genbank:XP_044405753.1;Name=XP_044405753.1;gbkey=CDS;gene=LOC123129798;product=

Protein DMP3-like.

Kozak: CCCATGT ID=cds-XP_044406379.1;Parent=rna-XM_044550444.1;Dbxref=GeneID:123130575,Genbank:XP_044406379.1;Name=XP_044406379.1;gbkey=CDS;gene=LOC123130575;product=CASP-like protein 2D1;protein_id=XP_044406379.1

GO:0016021; Integral component of membrane.

Kozak: CAGATGC ID=cds-XP_044406447.1;Parent=rna-XM_044550512.1;Dbxref=GeneID:123130688,Genbank:XP_044406447.1;Name=XP_044406447.1;Note=The sequence of the model RefSeq protein was modified relative to this genomic sequence to represent the inferred CDS: added 613 bases not found in genome assembly;exception=annotated by transcript or proteomic data;gbkey=CDS;gene=LOC123130688;inference=similar to RNA sequence (same species):INSD:GIJS01149649.1;partial=true;product=probable L-ascorbate peroxidase 7%2C chloroplastic;protein_id=XP_044406447.1

GO:0005737; Cytoplasm.

Kozak: CCCATGC ID=cds-XP_044406558.1;Parent=rna-XM_044550623.1;Dbxref=GeneID:123130816,Genbank:XP_044406558.1;Name=XP_044406558.1;gbkey=CDS;gene=LOC123130816;product=

Formin-like protein 14.

Kozak: CCCATGC ID=cds-XP_044406861.1;Parent=rna-XM_044550926.1;Dbxref=GeneID:123131194,Genbank:XP_044406861.1;Name=XP_044406861.1;gbkey=CDS;gene=LOC123131194;product=ethylene-responsive transcription factor ERF003-like;protein_id=XP_044406861.1

GO:0005634; Nucleus.

Kozak: CCAATGT ID=cds-XP_044406889.1;Parent=rna-XM_044550954.1;Dbxref=GeneID:123131232,Genbank:XP_044406889.1;Name=XP_044406889.1;gbkey=CDS;gene=LOC123131232;product=

BTB/POZ and MATH domain-containing protein 1-like.

Kozak: CGAATGT ID=cds-XP_044406951.1;Parent=rna-XM_044551016.1;Dbxref=GeneID:123131318,Genbank:XP_044406951.1;Name=XP_044406951.1;gbkey=CDS;gene=LOC123131318;product=

Putative F-box protein PP2-B12.

Kozak: TCCATGT ID=cds-XP_044407267.1;Parent=rna-XM_044551332.1;Dbxref=GeneID:123131662,Genbank:XP_044407267.1;Name=XP_044407267.1;gbkey=CDS;gene=LOC123131662;product=sucrose:sucrose 1-fructosyltransferase-like;protein_id=XP_044407267.1

GO:0016021; Integral component of membrane.

Kozak: TTCATGT ID=cds-XP_044407504.1;Parent=rna-XM_044551569.1;Dbxref=GeneID:123131869,Genbank:XP_044407504.1;Name=XP_044407504.1;gbkey=CDS;gene=LOC123131869;product=uncharacterized protein LOC123131869;protein_id=XP_044407504.1

GO:0048364; Root development.

Kozak: TACATGC ID=cds-XP_044407622.1;Parent=rna-XM_044551687.1;Dbxref=GeneID:123131956,Genbank:XP_044407622.1;Name=XP_044407622.1;gbkey=CDS;gene=LOC123131956;product=

Putative pentatricopeptide repeat-containing protein At1g19290.

Kozak: CCCATGC ID=cds-XP_044407627.1;Parent=rna-XM_044551692.1;Dbxref=GeneID:123131964,Genbank:XP_044407627.1;Name=XP_044407627.1;gbkey=CDS;gene=LOC123131964;product=vacuolar protein sorting-associated protein 8 homolog isoform X1;protein_id=XP_044407627.1

GO:0030897; HOPS complex

Kozak: CAGATGC ID=cds-XP_044407690.1;Parent=rna-XM_044551755.1;Dbxref=GeneID:123132008,Genbank:XP_044407690.1;Name=XP_044407690.1;gbkey=CDS;gene=LOC123132008;product=polyubiquitin;protein_id=XP_044407690.1

GO:0005737; Cytoplasm.

Kozak. CCCATGT ID=cds-XP_044407742.1;Parent=rna-XM_044551807.1;Dbxref=GeneID:123132061,Genbank:XP_044407742.1;Name=XP_044407742.1;gbkey=CDS;gene=LOC123132061;product=

Pentatricopeptide repeat-containing protein At2g17140-like.

Kozak: CCCATGT ID=cds-XP_044407769.1;Parent=rna-XM_044551834.1;Dbxref=GeneID:123132077,Genbank:XP_044407769.1;Name=XP_044407769.1;gbkey=CDS;gene=LOC123132077;product=

Phosphatidylinositol/phosphatidylcholine transfer protein SFH12-like isoform X1.

Kozak: CCAATGC ID=cds-XP_044408045.1;Parent=rna-XM_044552110.1;Dbxref=GeneID:123132328,Genbank:XP_044408045.1;Name=XP_044408045.1;gbkey=CDS;gene=LOC123132328;product=receptor-like protein 3;protein_id=XP_044408045.1

GO:0016021; Integral component of membrane.

Kozak: CCAATGC ID=cds-XP_044408047.1;Parent=rna-XM_044552112.1;Dbxref=GeneID:123132333,Genbank:XP_044408047.1;Name=XP_044408047.1;gbkey=CDS;gene=LOC123132333;product=tyrosine-sulfated glycopeptide receptor 1-like;protein_id=XP_044408047.1

GO:0016021; Integral component of membrane.

Kozak: CCAATGT ID=cds-XP_044408048.1;Parent=rna-XM_044552113.1;Dbxref=GeneID:123132337,Genbank:XP_044408048.1;Name=XP_044408048.1;gbkey=CDS;gene=LOC123132337;product=receptor-like protein 2;protein_id=XP_044408048.1

GO:0016021; Integral component of membrane.

Kozak: CAGATGC ID=cds-XP_044408096.1;Parent=rna-XM_044552161.1;Dbxref=GeneID:123132383,Genbank:XP_044408096.1;Name=XP_044408096.1;gbkey=CDS;gene=LOC123132383;product=polyubiquitin;protein_id=XP_044408096.1

GO:0005737; Ccytoplasm.

Kozak: CAGATGC ID=cds-XP_044408098.1;Parent=rna-XM_044552163.1;Dbxref=GeneID:123132384,Genbank:XP_044408098.1;Name=XP_044408098.1;gbkey=CDS;gene=LOC123132384;product=polyubiquitin;protein_id=XP_044408098.1

GO:0005737; Cytoplasm.

Kozak: CCCATGC ID=cds-XP_044408279.1;Parent=rna-XM_044552344.1;Dbxref=GeneID:123132524,Genbank:XP_044408279.1;Name=XP_044408279.1;gbkey=CDS;gene=LOC123132524;product=ultraviolet-B receptor UVR8-like;protein_id=XP_044408279.1

GO:0009881; Photoreceptor activity.

Kozak: CAGATGC ID=cds-XP_044408357.1;Parent=rna-XM_044552422.1;Dbxref=GeneID:123132592,Genbank:XP_044408357.1;Name=XP_044408357.1;gbkey=CDS;gene=LOC123132592;product=

Ultraviolet-B receptor UVR8-like.

Kozak: CCCATGC ID=cds-XP_044404153.1;Parent=rna-XM_044548218.1;Dbxref=GeneID:606390,Genbank:XP_044404153.1;Name=XP_044404153.1;gbkey=CDS;gene=LOC606390;product=dof zinc finger protein 4;protein_id=XP_044404153.1

GO:0005634; Nucleus.

Kozak: TTCATGT ID=cds-XP_044445546.1;Parent=rna-XM_044589611.1;Dbxref=GeneID:123172668,Genbank:XP_044445546.1;Name=XP_044445546.1;gbkey=CDS;gene=LOC123172668;product=

Pentatricopeptide repeat-containing protein At1g18900-like.

Kozak. CCCATGT ID=cds-XP_044446580.1;Parent=rna-XM_044590645.1;Dbxref=GeneID:123176446,Genbank:XP_044446580.1;Name=XP_044446580.1;gbkey=CDS;gene=LOC123176446;product=sugar transporter ERD6-like 5 isoform X1;protein_id=XP_044446580.1

GO:0016021; Integral component of membrane.

Kozak: TTAATGC ID=cds-XP_044445200.1;Parent=rna-XM_044589265.1;Dbxref=GeneID:123172265,Genbank:XP_044445200.1;Name=XP_044445200.1;gbkey=CDS;gene=LOC123172265;product=DIBOA-glucoside dioxygenase BX6-like;protein_id=XP_044445200.1

GO:0051213; Dioxygenase activity.

Kozak: CCAATGT ID=cds-XP_044446433.1;Parent=rna-XM_044590498.1;Dbxref=GeneID:123176121,Genbank:XP_044446433.1;Name=XP_044446433.1;gbkey=CDS;gene=LOC123176121;product=

Methionine S-methyltransferase-like.

Kozak: CCCATGT ID=cds-XP_044445816.1;Parent=rna-XM_044589881.1;Dbxref=GeneID:123173015,Genbank:XP_044445816.1;Name=XP_044445816.1;gbkey=CDS;gene=LOC123173015;product=

TATA-box-binding protein 1-like isoform X1.

Kozak: TACATGC ID=cds-XP_044446446.1;Parent=rna-XM_044590511.1;Dbxref=GeneID:123176159,Genbank:XP_044446446.1;Name=XP_044446446.1;gbkey=CDS;gene=LOC123176159;product=pre-mRNA-splicing factor ATP-dependent RNA helicase DEAH1-like;protein_id=XP_044446446.1

GO:0005524; ATP binding.

Kozak. TTCATGT ID=cds-XP_044445976.1;Parent=rna-XM_044590041.1;Dbxref=GeneID:123174365,Genbank:XP_044445976.1;Name=XP_044445976.1;gbkey=CDS;gene=LOC123174365;product=

Putative F-box protein At2g02030 isoform X1.

Kozak: CCAATGC ID=cds-XP_044447137.1;Parent=rna-XM_044591202.1;Dbxref=GeneID:123177477,Genbank:XP_044447137.1;Name=XP_044447137.1;end_range=1980,.;gbkey=CDS;gene=LOC123177477;partial=true;product=uncharacterized protein LOC123177477;protein_id=XP_044447137.1

GO:0005737; Cytoplasm.

Kozak: TACATGC ID=cds-XP_044446504.1;Parent=rna-XM_044590569.1;Dbxref=GeneID:123176281,Genbank:XP_044446504.1;Name=XP_044446504.1;gbkey=CDS;gene=LOC123176281;product=probable LRR receptor-like serine/threonine-protein kinase At3g47570;protein_id=XP_044446504.1

GO:0016021; Integral component of membrane.

Kozak: CCAATGT ID=cds-XP_044446793.1;Parent=rna-XM_044590858.1;Dbxref=GeneID:123176812,Genbank:XP_044446793.1;Name=XP_044446793.1;gbkey=CDS;gene=LOC123176812;product=

Tricetin 3'%2C4'%2C5'-O-trimethyltransferase-like.

Kozak: CCCATGT ID=cds-XP_044446849.1;Parent=rna-XM_044590914.1;Dbxref=GeneID:123176888,Genbank:XP_044446849.1;Name=XP_044446849.1;Note=The sequence of the model RefSeq protein was modified relative to this genomic sequence to represent the inferred CDS: added 405 bases not found in genome assembly;exception=annotated by transcript or proteomic data;gbkey=CDS;gene=LOC123176888;inference=similar to RNA sequence (same species):INSD:HAAB01040796.1;partial=true;product=mitogen-activated protein kinase kinase 3-like;protein_id=XP_044446849.1;start_range=.,796

GO:0005524; ATP binding.

Kozak: TACATGC ID=cds-XP_044447359.1;Parent=rna-XM_044591424.1;Dbxref=GeneID:123178252,Genbank:XP_044447359.1;Name=XP_044447359.1;gbkey=CDS;gene=LOC123178252;product=probable LRR receptor-like serine/threonine-protein kinase At3g47570;protein_id=XP_044447359.1

GO:0016021; Integral component of membrane.

Kozak: CCCATGT ID=cds-XP_044446178.1;Parent=rna-XM_044590243.1;Dbxref=GeneID:123175596,Genbank:XP_044446178.1;Name=XP_044446178.1;gbkey=CDS;gene=LOC123175596;product=3-ketoacyl-CoA synthase 5-like;protein_id=XP_044446178.1

GO:0016021; Integral component of membrane.

Kozak: TCCATGT ID=cds-XP_044446585.1;Parent=rna-XM_044590650.1;Dbxref=GeneID:123176454,Genbank:XP_044446585.1;Name=XP_044446585.1;gbkey=CDS;gene=LOC123176454;product=disease resistance protein RGA5-like;protein_id=XP_044446585.1

GO:0043531; ADP binding.
