# Supplementary material for: Functional analysis of the AUG initiator codon context reveals novel conserved sequences that disfavor mRNA translation in eukaryotes
Source: Nucleic Acids Res. 2023 Dec 1;52(3):1064–79. doi: 10.1093/nar/gkad1152 (PMC10853783; doi:10.1093/nar/gkad1152)
Supplement: gkad1152_supplemental_files [file gkad1152_supplemental_files.zip › Supplementary Material.pdf]

Supplementary Material of:

Functional analysis of the AUG initiator codon context reveals novel conserved sequences that disfavor mRNA translation in eukaryotes

Greco Hernández, Alejandra García, Shira Weingarten-Gabbay, Rishi Kumar Mishra, Tanweer Hussain, Mehdi Amiri, Gabriel Moreno-Hagelsieb, Angélica Montiel-Dávalos, Paul Lasko, and Nahum Sonenberg

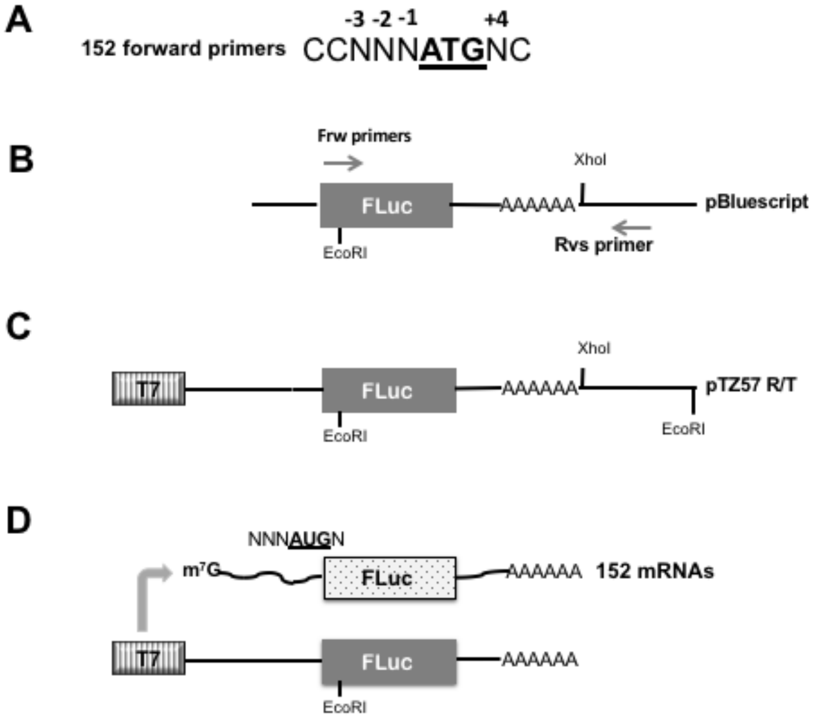

Suppl. Fig. 1 Cloning and synthesis of 152 reporter mRNAs with different TIS context. ***A)*** 152 different forward (*Frw*) primers with mutations at the *-3*, *-2*, *-1*, and *+4* positions surrounding the *AUG* TIS (*underlined*). ***B)*** Constructs were PCR-amplified using pLUC-cassette (29) in pBluescript that contains the firefly (*FLuc*) cistron and a poly$(A)_{71}$ tail as a template, the forward (*Frw*) primers and a unique reverse (*Rvs*) primer. ***C)*** Fragments were cloned onto the pTZ57 R/T downstream of the T7 promoter. ***D)*** *In vitro* transcription of capped and polyadenylated mRNA driven by the T7 promoter. *N*, any nucleotide.
